# Turn-Mimic Hydantoin-Based Loops Constructed by a Sequential Multicomponent Reaction

**DOI:** 10.1021/acs.joc.3c01861

**Published:** 2023-11-06

**Authors:** Alessio Maria Caramiello, Maria Cristina Bellucci, Javier Marti-Rujas, Alessandro Sacchetti, Alessandro Volonterio

**Affiliations:** † Department of Chemistry, Material and Chemical Engineering “Giulio Natta”, Politecnico di Milano, via Mancinelli 7, Milano 20131, Italy; ‡ Department of Food, Environmental and Nutritional Sciences, Università degli Studi di Milano, via Celoria 2, Milano 20133, Italy

## Abstract

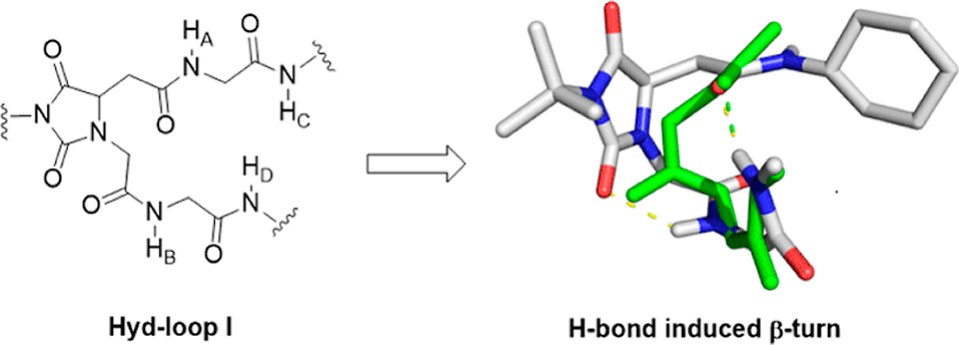

A collection of peptidomimetics characterized by having an aspartic acid motif embedded in a rigid hydantoin heterocycle are synthesized through a sequential multicomponent domino process followed by standard regioselective deprotection/coupling reactions based on acid–base liquid/liquid purification protocols. ^1^H nuclear magnetic resonance experiments, molecular modeling, and X-ray analysis showed that the resulting hydantoin-based loops I (in particular) and II (to a lesser extent) can be considered novel β-turn inducer motifs being able to project two peptide-like strands in a U-shaped conformation driven by the formation of intermolecular hydrogen bonds.

## Introduction

**Figure 1:**
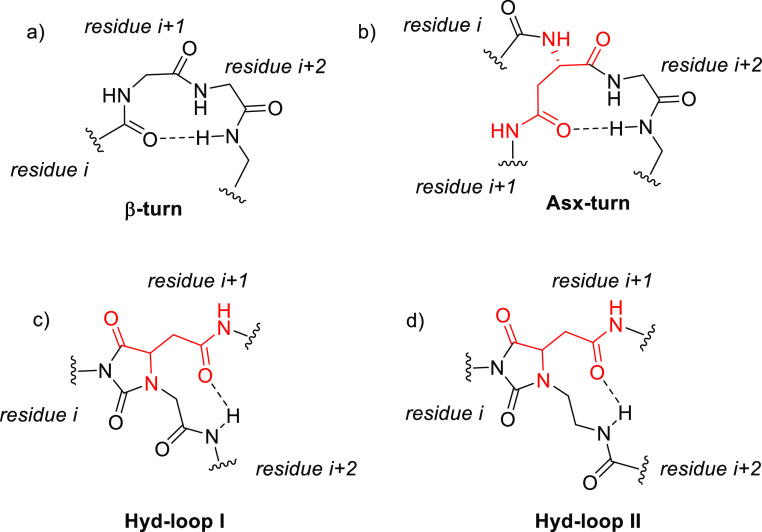
Geometrical representations of (a) classical β-turn conformation, (b) Asn–Gly-induced turn conformation, (c) predicted **Hyd-loop I**-induced turn conformation, and (d) predicted **Hyd-loop II**-induced turn conformation.

One of the drawbacks of the use of short peptides as drugs is their inability to retain organized secondary structures essential for the biological activity.^1^ For this reason, different motifs have been developed to stabilize protein secondary structures, thus improving peptide leads. In particular, the quest for scaffolds capable of mimicking turn motifs has garnered significant attention, as turns play a crucial role in stabilizing conformations involved in many biological molecular recognition events.^2^ With the turn motif being characterized by the dihedral angles of the amide backbone, the typical strategy to induce a turn conformation in peptidomimetics is to design scaffolds that favor the intramolecular formation of hydrogen bonds (H-bonds) involving the C=O belonging to the peptide bond (Figure 1, top left).^3^ Turn conformations have also been observed to be triggered by intramolecular H-bonds involving the C=O groups of aspartic acid (Asp) or asparagine (Asn) side chains (Figure 1, top right).^4^ However, Asx-turns are very often formed along a second secondary structure, mainly β-turn, providing a well-defined H-bond-driven intersected conformation referred to as Asx-β-turn.^5^ To date, the debate on which turn pre-exists, thus fostering the formation of the second, is still open.^6^ Nevertheless, recent in silico research has shown that Asx–Gly sequence in a small dipeptide model is able to induce type II′ β-turns independently.^7^ Since an effective approach to stabilize turn conformation is the synthesis of rigid peptide turn surrogates, we present herein the design, synthesis, and conformational analysis of novel hydantoin-based loops, where (1) the structure of Asn is embedded into a more rigid hydantoin five-membered ring, and (2) a flexible glycine or an even more flexible ethylenediamino residue is tethered to the Nα of Asn (or N1 of the hydantoin ring), respectively, in **Hyd-loop I** and **Hyd-loop II** (Figure 1, bottom). We reasoned that if a turn structure can exist in a flexible Asp–Gly sequence, even if triggered by a pre-existing β-turn, the designed hydantoin loops could possibly induce in itself a defined turn conformation with a 9-membered ring intramolecular H-bond. The synthesis of such peptidomimetics is achieved by exploiting a sequential multicomponent (MC) process followed by standard hydrolysis/coupling reactions using liquid–liquid acid/base extraction protocols for the purification of the intermediates.^8^ The strategy described herein is suitable for the combinatorial synthesis of wide libraries of such compounds, thus overcoming the drawbacks of tedious multistep, time-consuming synthetic pathways often exploited for the synthesis of turn motifs.

## Results and Discussion

### Synthesis

In medicinal chemistry, hydantoin heterocycle is considered to be a privileged scaffold from which it is possible to prepare compounds with a wide spectrum of biological activities.^9^ Moreover, thanks to its planar conformation with two carbonyl groups that could be involved in hydrogen bonding, it has been exploited as a building block for the synthesis of peptidomimetics with well-defined secondary structures.^10^ Due to the wide interest raised by this heterocycle during the time, in the past decade, we have developed a MC process for the synthesis of differently substituted hydantoin derivatives with biological interest.^11^ Taking into account all these features, we have designed **Hyd-loop I** and **Hyd-loop II** frameworks as possible inducers of turn structures, which would be accessible with the MC process developed by us. Compared to the structure of the hydantoin-based universal peptidomimetic previously reported^8,10c^ where the extended strands are attached to the N3 nitrogen of the hydantoin ring and to the C5 hydantoin carbon, the **Hyd-loop I** and **Hyd-loop II** scaffolds bear the extended strands in the vicinal N1 hydantoin nitrogen and C5 hydantoin carbon. The synthetic pathway foresees the in situ formation of carbodiimides **3** by Staudinger/aza-Wittig reaction between primary azides **1** and commercially available tertiary *tert*-butyl or adamantyl (Ad) isocyanates **2** (Table 1). After completion of the reaction, fumaric acid mono-*p*-nitrophenylester **4** was added to the reaction mixture providing the formation of hydantoin intermediates **5** through a domino process involving a regiospecific condensation/intramolecular aza-Michael/O–N acyl migration domino process. The regiocontrol of the reaction is due to the preferential nucleophilic attack of the primary amine moiety compared to the sterically hindered tertiary amine moiety during the intramolecular aza-Michael step.^12^ Finally, the addition in situ of amines, either alkyl, aryl, or glycine derivatives, yields hydantoin-based peptidomimetics **6** by nucleophilic displacement of *p*-nitrophenol at room temperature.

**Table 1:**
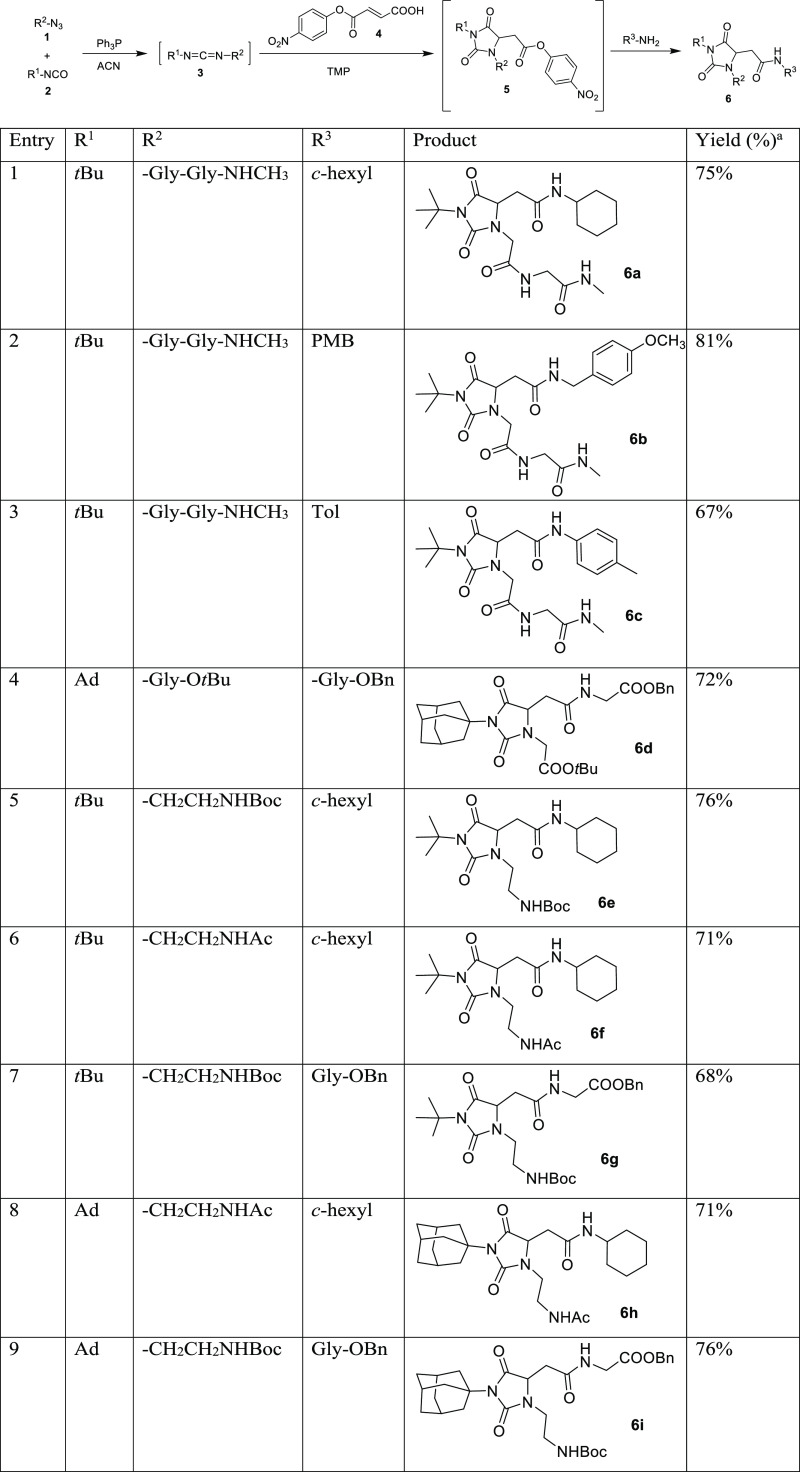
MC Sequential Synthesis of Hydantoin-Based Peptidomimetics **6**

a Overall yields.

**Scheme 1:**
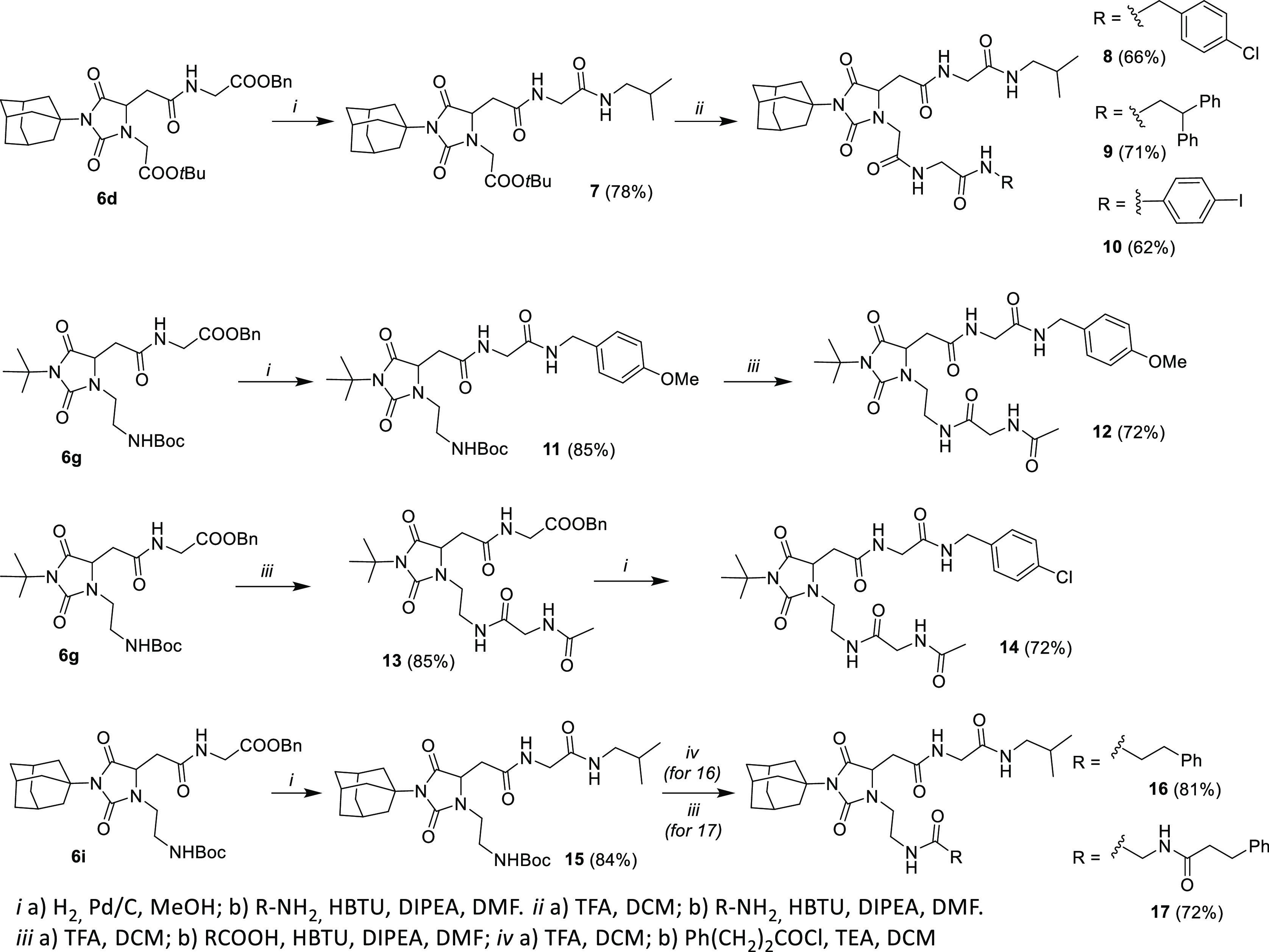
Synthesis of Elongated Hydantoin-Based Peptidomimetics **8–10**, **12**, **14**, **16**, **17**

The process worked efficiently starting from glycine azide *tert*-butylester (entry 4, Table 1) and diglycine azide methylamide (entries 1–3, Table 1) providing the formation of four peptidomimetics based on **Hyd-loop I** (compounds **6a–d**, Table 1) and with *N*-Boc ethylenediamine mono azide (entries 5, 7, 9, Table 1) or *N*-Ac-ethylenediamine mono azide (entries 6, 8, Table 1) yielding other five peptidomimetics based on **Hyd-loop II** (compounds **6e–i**, Table 1). It is worth noting that peptidomimetics **6d, g, i**, having terminal functional groups orthogonally protected, are prone to further elongation. Indeed, we synthesized 6 new peptidomimetics **8–10, 12, 14–15** bearing two artificial dipeptide strands through classical deprotection/condensation procedures using liquid–liquid acid/base extraction protocols for the purification of the intermediates in high yields (Scheme 1). More specifically, the benzyl ester of hydantoin **6d** was selectively hydrolyzed by treatment with hydrogen in the presence of a catalytic amount of Pd/C, and the resulting carboxylic acid coupled with H–Gly–CONH–*i*Bu providing intermediate **7**. Thus, the *tert*-butyl ester was cleaved upon treatment with TFA in DCM, and the resulting carboxylic acid coupled with *p*-Cl-benzyl amine, 2,2-diphenyl-ethylamine, and *p*-I-phenylamine affording peptidomimetics **8–10**, respectively, in good yields. Analogously, after selective hydrogenolysis of the benzyl ester, the upper strand of hydantoin **6g** was elongated with H–Gly–CONH–PMB yielding intermediate **11,** which was Boc deprotected with TFA and coupled with AcNH–Gly–OH producing peptidomimetic **12**. We could also first elongate the lower arm of **6g** by *tert*-butyloxycarbonyl (Boc)-deprotection of the amino function (TFA/DCM) and coupling with AcNH–Gly–OH yielding **13**, followed by hydrogenolysis of the benzyl ester and coupling with *p*-Cl-benzyl amine leading to peptidomimetic **14**. Lastly, the corresponding adamantyl derivative **6i**, having a benzyl ester on the upper strand and an amino group protected as Boc in the lower strand, was first submitted to hydrogenolysis and coupled with iso-propyl amine leading to the formation of **15** and then Boc deprotected and coupled with 3-phenyl-propanoyl chloride or Ph–CH_2_–CH_2_–CONH–Gly–OH affording **16** and **17**, respectively.

### Conformational Analysis: NMR

To get insights on the possible conformations that **Hyd-loop I** and **Hyd-loop II** could trigger in solution, we sought to identify the presence of possible intramolecular H-bonds through proton nuclear magnetic resonance (^1^H NMR) experiments. The first feature to take in consideration is the chemical shift of the amidic protons –CONH– in relatively nonpolar solvents such as CDCl_3_. Indeed, it is well documented that the amidic protons resonating at lower fields, typically around 8.0 ppm, are likely to be involved in intramolecular hydrogen bonding, whereas hydrogens resonating at higher fields are not.^13^ The chemical shifts of the amidic protons NH_A_–NH_D_ of **Hyd-loop I** peptidomimetics and NH_A_, NH_B′_, NH_C_, NH_D′_ belonging to **Hyd-loop II** peptidomimetics for which it was possible to recover the ^1^H NMR spectra in CDCl_3_ were assigned through correlation spectroscopy (COSY) experiments (see the Supporting Information) and are reported in Table 2.^14^ Interestingly, in short, peptidomimetics **6**, having only one amidic proton in the upper strand, namely NH_A_, and one or two amidic protons in the lower arm (NH_B/B′_ and NH_D/D′_), only the **Hyd-loop I** seems to trigger the formation of an intramolecular H-bond involving NH_B_, which resonates at 8.02 and 7.78 ppm for compounds **6a,b**, respectively (entries 1 and 2, Table 2), whereas all the amidic protons of peptidomimetics of type **6** built on the **Hyd-loop II** resonate at ppm lower than 7.5 (entries 3–8, Table 2). These results strongly suggest that the **Hyd-loop I** likely induces the formation of a H-bond-driven β-turn conformation in solution, while the presence of a more flexible ethylenediamino residue in **Hyd-loop II** hampers the formation of strong intramolecular H-bonds, at least in short sequences. The capability of the **Hyd-loop I** to induce turn conformations in solution is maintained also in peptidomimetics having two artificial dipeptide arms, such as **8** (entry 9, Table 2). Indeed, amidic protons NH_A_ and NH_B_ are likely involved in intramolecular H-bond, whereas proton NH_C_, resonating at 6.53 ppm, is clearly free in solution. It is noteworthy that also proton NH_D_ (7.66 ppm) seems involved to some extent in the formation of an intramolecular H-bond, corroborating the results obtained from X-ray analysis (see below). Very interestingly, also **Hyd-loop II** seems to be able to induce the formation of a defined intramolecular hydrogen bonding network when the lower arm is elongated by a glycinamide residue. Indeed, the presence of a further amide functional group in the lower arm seems to trigger the formation of two intramolecular H-bonds involving protons NH_A_ and NH_B′_, which resonate at ppm higher than 8.0 in peptidomimetics **12**, **14**, and **16** (entries 10, 12, 15, respectively, Table 2). On the contrary, when **Hyd-loop II** is elongated only in the upper arm, such as in peptidomimetics **15** and **16** (entries 13 and 14, respectively, Table 2), there is no evidence of the presence of any H-bonds being all the amidic protons resonating at ppm lower than 7.0, whereas weaker intramolecular H-bonds involving proton NH_A_ and NH_B′_ seem to be present in peptidomimetic **13** having an extended lower arm (entry 11, Table 2).

**Table 2:**
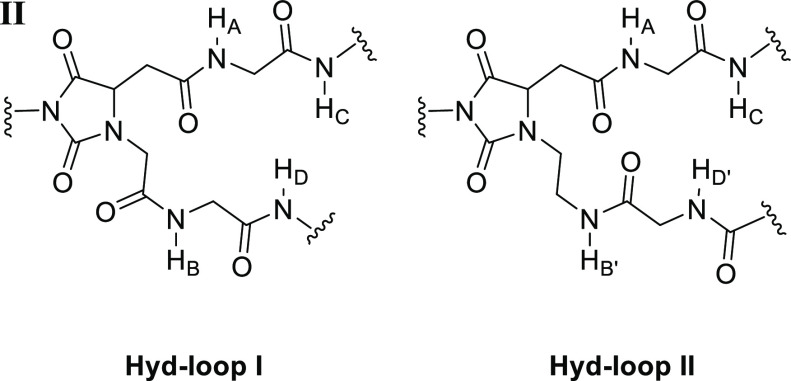
Chemical Shifts of Amidic Protons CONH in Peptidomimetics Built on **Hyd-Loops I** and **II**^a^

entry	compound	δ NH_A_	δ NH_B_	δ NH_B′_	δ NH_C_	δ NH_D_	δ NH_D′_
1	**6a**	5.74	**8.02**			6.80	
2	**6b**	6.79	**7.78**			6.90	
3	**6d**	6.70					
4	**6e**	5.82		5.07			
5	**6f**	5.91		6.84			
6	**6g**	6.79		5.34			
7	**6h**	6.12		7.09			
8	**6i**	6.48		5.22			
9	**8**	**7.61**	**8.07**		6.53	**7.66**	
10	**12**	**8.21**		**8.19**	7.01		6.83
11	**13**	**7.90**		**7.61**			7.10
12	**14**	**8.20**		**8.11**	7.06		6.97
13	**15**	6.56		6.94	6.62		
14	**16**	6.94		6.61	6.62		
15	**17**	**8.23**		**8.04**	7.20		6.62

a NMR experiments performed in 2.5 nM CDCl_3_ solutions.

**Figure 2:**
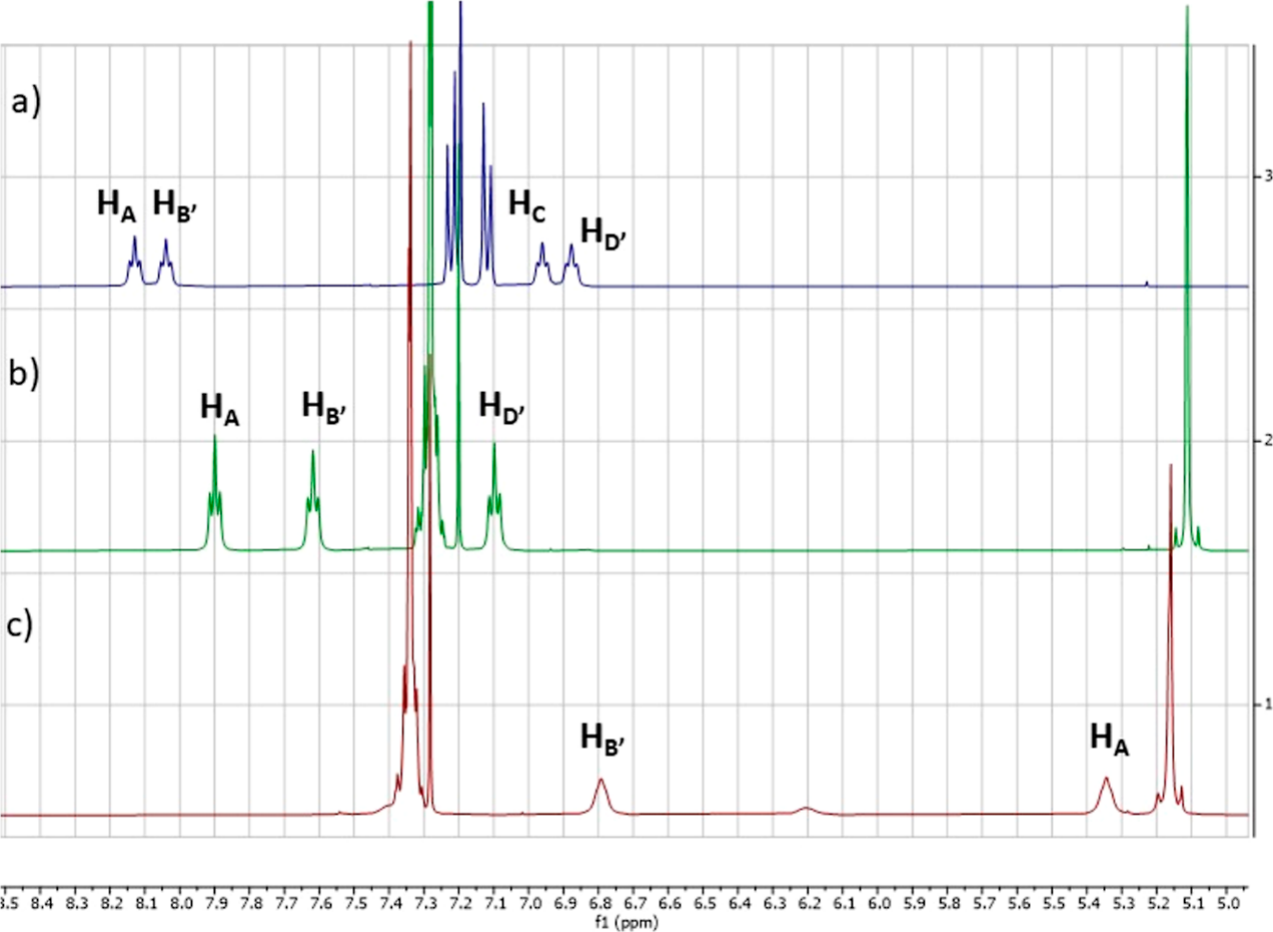
Partial ^1^H NMR spectra of (a) **14**, (b) **13**, and (c) **6g**.

**Figure 3:**
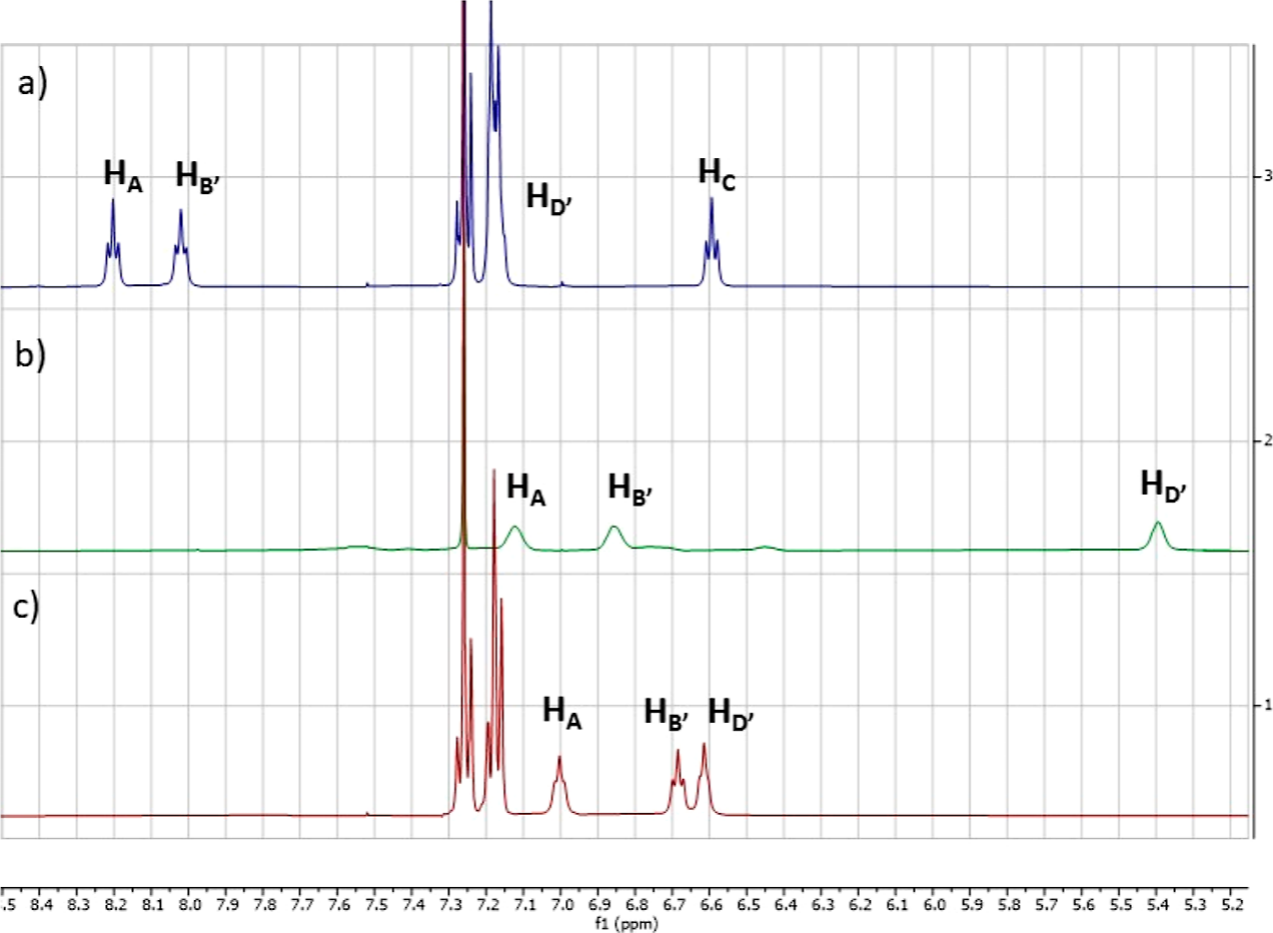
Partial ^1^H NMR spectra of (a) **17**, (b) **15**, and (c) **16**.

Notably, compared to that of the precursors **6g** and **13**, the chemical shifts of amidic protons NH_A_ and NH_B′_ in peptidomimetic **14** were shifted downfield pronouncedly by, respectively, 2.8 and 0.3 ppm for H_A_ and 1.3 and 1.0 ppm for NH_B′_, suggesting that both protons are hydrogen-bonded (Figure 2). Likewise, if we compare the chemical shifts of the same protons H_A_ and H_B′_ of intermediate **15** with those of peptidomimetics **16** and **17**, we measured downfield shifts only in compound **17**, namely, 1.0 ppm for H_A_ and 1.2 ppm for H_B′_, whereas analogous shifts were not observed for compound **16** having only one amidic group in the lower strand (Figure 3).

Next, we performed variable temperature (VT) ^1^H NMR experiments on model peptidomimetics **6a**, **8**, based on **Hyd-loop I** and **14** based on **Hyd-loop II** (Table 3). We measured the variation of the chemical shifts of the amidic protons in 2.0 mM CDCl_3_ solution of **6a**, **8**, and **14**, and in 2.0 mM dimethyl sulfoxide (DMSO)-*d*_6_ solution of **6a** and **8**. Indeed, the variation of the chemical shifts of amidic protons within the rising of the temperature is a parameter often considered to get insights on the presence/strength of intramolecular H-bonds. More in detail, in low-polarity solvents, temperature coefficient significantly larger than 2.4 ppb/K can be assigned to protons engaged in intramolecular H-bonds, which are weakened upon increasing the temperature, whereas values lower than 2.4 ppb/K are not really indicative because they can be assigned to protons either accessible or not accessible to the solvent.^15^ On the other hand, in polar DMSO-*d*_6_ values of δΔ/δ*T* < 5 ppb/K denote not accessible protons, thus already involved in H-bond.^16^ The temperature coefficient measured in CDCl_3_ solutions were not very useful to clarify the presence of intramolecular H-bonds. Actually, the value of δΔ/δ*T* obtained for NH_B_ of peptidomimetic **6a** based on **Hyd-loop I**, i.e., the proton that seemed to be involved in intramolecular hydrogen bond considering its chemical shift (Table 3), was equal to 11.4 ppb/K, higher than the reference of 2.4 ppb/K but not as high as those we observed in the previous study.^10c^ Moreover, the values obtained for all the amidic protons in peptidomimetic **8** having an extended upper strand were even lower. However, the temperature coefficient measured for the same peptidomimetics in DMSO-*d*_6_ solutions were smaller than 5.0 ppb/K, suggesting the possibility for these peptidomimetics to adopt different conformations where the amidic NH could be involved in hydrogen bonding. The results obtained for peptidomimetic **14** based on **Hyd-loop II** demonstrate a certain tendency of proton NH_A_, NH_B′_, and NH_D′_ to be involved in H-bonding, having δΔ/δ*T* values respectively of 18.1, 13.4, and 13.2 ppb/K.

**Table 3:** Δδ/Δ*T* Values for the Amidic Protons of Some Hydantoin-Based Peptidomimetics^a^

entry	compound	solvent	Δδ/Δ*T*-H_A_ (ppb/K)^b^	Δδ/Δ*T*-H_B_ (ppb/K)^b^	Δδ/Δ*T*-H_B′_ (ppb/K)^b^	Δδ/Δ*T*-H_C_ (ppb/K)^b^	Δδ/Δ*T*-H_D_ (ppb/K)^b^	Δδ/Δ*T*-H_D′_ (ppb/K)^b^
1	**6a**	CDCl_3_	1.1	11.4			5.7	
2	**6a**	DMSO-*d*_6_	4.4	4.2			4.5	
3	**8**	CDCl_3_	5.9	6.6		0.7	7.0	
4	**8**	DMSO-*d*_6_	4.5	4.4		4.8	4.4	
5	**14**	CDCl_3_	18.1		13.4	5.3		13.2

a NMR experiments performed in 2.5 mM solutions.

b Absolute values.

**Figure 4:**
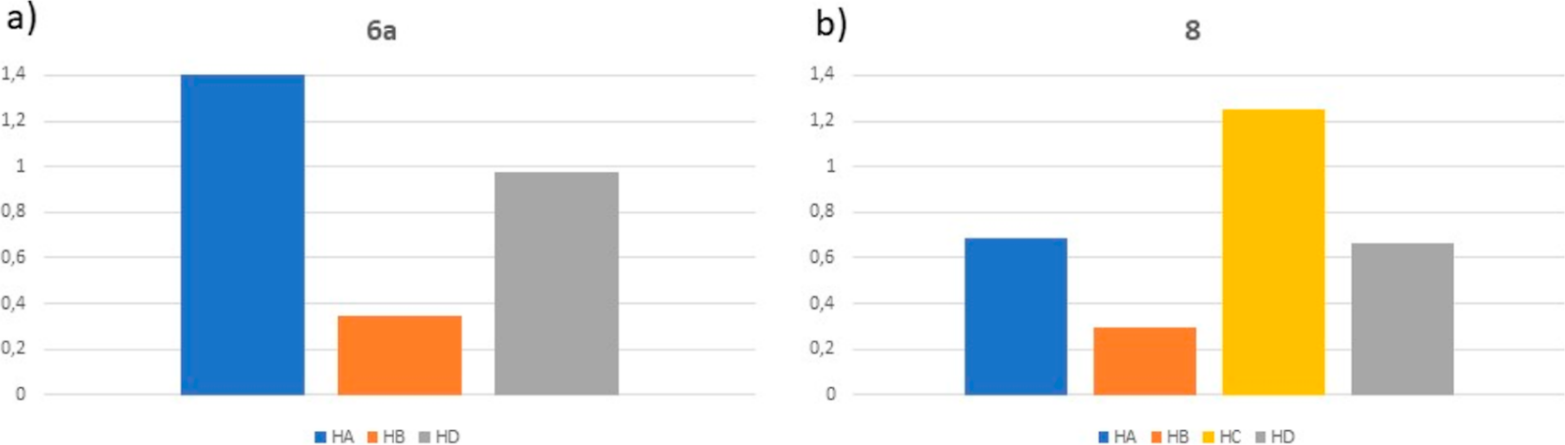
(δ_CDCl_3__ – δ_DMSO-*d*_6__) values as a measure of the solvent accessibility of amidic –CONH– in (a) **6a** and (b) **8**.

Given that we have recorded the spectra of **Hyd-loop I** peptidomimetics **6a** and **8** in both 2.5 mM CDCl_3_ and DMSO-*d*_6_ solutions, we calculated the difference in the chemical shifts of the amidic proton as a measure of their accessibility (Figure 4).^17^ In both cases, hydrogen H_B_ showed the smallest difference, suggesting its engagement in intramolecular hydrogen bonding.

**Figure 5:**
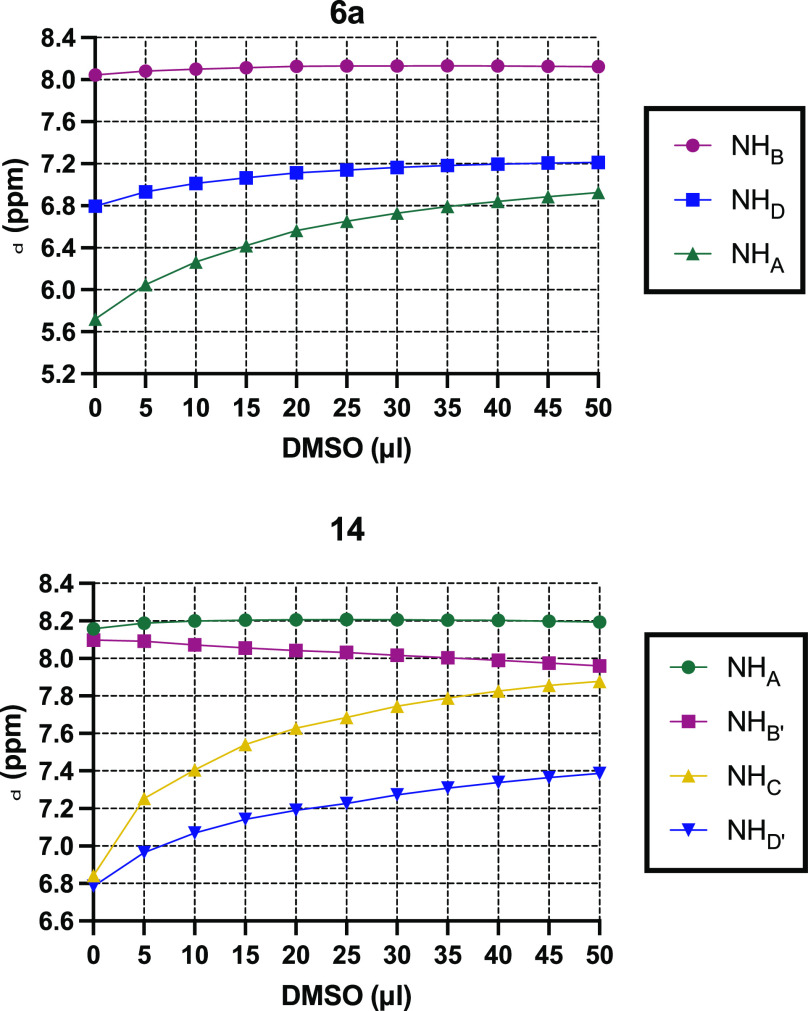
DMSO titration experiments on substrates **6a** and **14**.

Since the VT experiments did not clearly demonstrate the presence of strong intramolecular H-bonds, we performed DMSO titration experiments on peptidomimetics **6a** and **14** by recording the chemical shifts of the amidic protons in CDCl_3_ solutions (2.0 mM) upon addition of small aliquots (5 μL) of DMSO. In this experiment, the protons engaged in hydrogen bonding did not experience an evident chemical shift change after dilution with a coordinating solvent like DMSO, whereas sizable downfield shifts are recorded for protons free in solution, as a result of increasing H-bond with DMSO.^18^ As evidenced in Figure 5, in compound **6a**, proton H_B_ essentially did not shift upon dilution, whereas proton H_D_ and to a larger extent proton H_A_ showed a significant downfield shift. This result further corroborates the presence of a secondary structure triggered by an intramolecular H-bond involving H_B_ for peptidomimetics built on **Hyd-loop I**. Concerning **Hyd-loop II** peptidomimetic **14**, we did not record evident downfield shifted proton resonances for H_A_ and H_B′_, indicating their involvement in H-bonds, whereas for protons H_C_ and H_D′_, we observed a clear increase of chemical shifts due to their increased interaction with the titrating solvent. However, nuclear Overhauser enhancement spectroscopy (NOESY) experiment performed on compound **14** did not reveal clear long-range contacts supporting the possibility that the β-turn conformation promoted by **Hyd-loop II** could not be very robust.

### Conformational Analysis: Computation

Computational tools were employed to gather more information on the conformational behavior of the proposed turn mimics. In accordance with the commonly accepted definition of β-turn conformation, the presence of both an interatomic distance *d*α < 7 Å and the absolute value of the dihedral angle C1–C2–C3–N4 |β| < 60° were strictly considered as a necessary condition for the presence of this specific turn. Different techniques were used at different levels as molecular mechanics (MM), ab initio density functional theory (DFT), and molecular dynamics (MD). First, a conformational search by a combined MM–Monte Carlo (MM–MC) approach was performed. Structures **6a–c** (**Hyd-loop I**) and **6e–i** (**Hyd-loop II**) were submitted to the MM–MC analysis using both MMFF94 and MMFFaq force fields; the latter was chosen to evaluate the presence of an aqueous environment on the formation of the turn. For each structure, the conformers within 10 kcal/mol from the minimum were considered.

**Figure 6:**
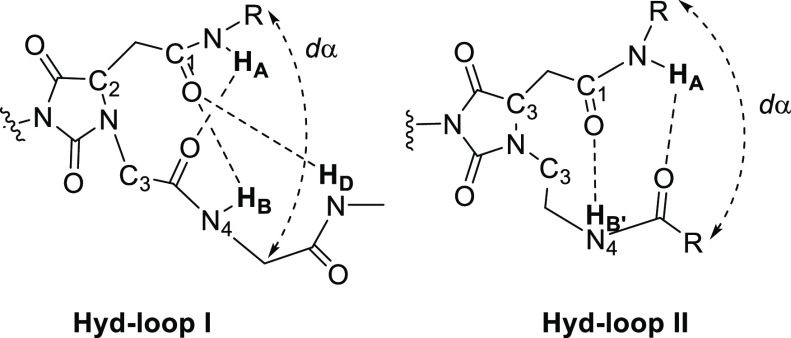
Geometrical parameters for the β-turn conformation for model compounds.

In addition to the *d*α and β values, the formation of an intramolecular H-bond is also indicative of the presence of a β-turn conformation; in a classical β-turn, this H-bond results in the formation of an 11-membered ring. In our case, two possible intramolecular H-bonds could be identified for the two Hyd-loop types. For **Hyd-loop I**, two 9-membered intramolecular hydrogen bond rings involving NH_A_ or NH_B_ could be formed, whereas for **Hyd-loop II**, a 9-membered (involving NH_B′_) and a 11-membered (involving H_A_) hydrogen bond rings can be defined (Figure 6). Due to the important contribution of these H-bonds in stabilizing the turn conformation, their presence was also evaluated.

Results from this study are reported in Table 4 as percentage of conformers that meet the requirements.

**Table 4:** Results from MM–MC Conformational Analysis: (a) In Vacuo and (b) in Water Results as Percentage of Conformers Meeting the Requirements

	*d*α < 7 Å		|β| < 60°		presence of β-turn		NH_B/B′···_O		NH_A···_O	
	(a)	(b)	(a)	(b)	(a)	(b)	(a)	(b)	(b)	(a)
**6a**	59	51	40	32	38	28	13	12	23	8
**6b**	69	63	41	35	38	31	15	8	14	8
**6c**	39	38	43	39	25	22	7	2	13	3
**6e**	60	59	34	33	34	33	17	17	15	7
**6f**	52	53	31	31	30	31	18	13	20	18
**6g**	66	58	46	29	34	23	10	5	31	22
**6h**	48	53	29	38	29	36	18	21	19	16
**6i**	63	63	45	37	31	36	7	11	27	24

All the structures showed a good propensity toward the adoption of a β-turn conformation, with about 30% of all conformers being within the desired values. The effect of water seemed to be insignificant, with only a small deviation from the values of the in vacuo calculations. As expected, a significant percentage of the conformers also have the considered intramolecular H-bonds, thus confirming the importance of this parameter. It must be noticed that for compounds **6a,b**, an extra H-bond involving the H_D_ hydrogen, resulting in a 12-membered ring, is possible. Indeed, this H-bond could be found in 13, 11, and 3% of conformers for **6a**, **6b**, and **6c** respectively. Anyway, a closer inspection of the results showed some differences in the global minimum conformers between **Hyd-loop I** and **Hyd-loop II**. In Table 5, the relative energies of the first β-turn conformers are reported. As can be seen, the only compounds for which the global minimum structures are a β-turn both in vacuo and in water are **Hyd-loop I 6a,b** (in **6c**, the presence of a terminal arylamide negatively affects the formation of the H-bond). In the case of **Hyd-loop II**, a less defined situation is present, with most of the β-turn conformations not being the global minimum. Even if in some cases the Δ*E* are not so high, this result points out the importance of the C_3_–C=O–N_4_ carbonyl moiety in favoring the stabilization of the turn.

**Table 5:** Relative Energies of the First β-Turn Conformer from MM–MC Analysis: (a) In Vacuo and (b) in Water Results^a^

	*d*α (Å)		β (deg)		rel *E* (kcal/mol)	
	(a)	(b)	(a)	(b)	(a)	(b)
**6a**	5.05	5.10	36.6	36.5	0.00	0.00
**6b**	5.80	4.62	29.9	35.0	0.00	0.00
**6c**	6.24	5.00	–58.4	–34.4	2.22	0.73
**6e**	5.53	5.51	24.2	24.1	0.00	0.55
**6f**	5.66	5.61	21.9	21.2	0.00	0.12
**6g**	5.66	5.65	20.3	20.2	2.11	1.66
**6h**	6.15	5.67	6.2	21.9	0.71	1.19
**6i**	5.65	5.66	20.65	20.66	1.94	1.47

a Values for *d*α (Å) and β (deg) are also reported.

To better evaluate this effect, selected compounds **6a** and **6f,g** were investigated by DFT (Table 6). The energy minima of both the first β-turn and open (not β-turn) conformations, as obtained from MM–MC, were submitted to a full energy optimization (with the calculation of vibrational energies to add for zero point energy correction) with DFT at the B3LYP 6-31+G(d,p) level. Energies were calculated in vacuo and with the contribution of water solvation. For compound **6a**, the β-turn is favored both in water and in vacuo, and the first open conformation is found at a substantially higher energy (5.33 and 6.22 kcal/mol for in vacuo and in water geometries ,respectively); these Δ*E* values virtually ensure a total preference for the turn conformation at room temperature. In this conformation, H_D_ is involved in the H-bond; this is agreement in with what was observed by solid-state X-ray analysis (vide infra). For compounds **6f,g**, the situation is less defined. The Δ*E* are quite small (0.60 to 1.46 kcal/mol), thus supporting the concurrent presence of both conformations at room temperature. For **6g**, the β-turn is preferred in water, whereas in vacuo, the open conformation is more stable. In this case, it can be noticed that for the open conformation, an acceptable *d*α = 5.95 Å value is found, but the high β angle value and lack of the H-bond gave this compound an open conformation. In conclusion, we can suggest that both the **Hyd-loop I** and **Hyd-loop II** are able to stabilize a β-turn conformation, but the presence of a carbonyl within the turn in compounds **6a,b** seems to have a great positive effect in stabilizing the turn.

**Table 6:** Results from DFT Calculations^a^

	rel. *E* (kcal/mol)		*d*α (Å)	β (deg)	H-bond type
	in vacuo	in water			
**6a**—β-turn	0.00	0.00	4.80	38.0	NH_D···_O
**6a′**—open	5.33	6.22	9.36	100.5	n.d^b^
**6f**—β-turn	0.00	0.00	5.29	14.4	NH_B′···_O
**6f’**—open	1.46	0.76	11.09	112.8	n.d^b^
**6g**—β-turn	0.00	0.60	5.54	16.3	NH_B′···_O
**6g′**—open	1.22	0.00	5.95	76.1	n.d^b^

a The values of *d*α (Å) and β (deg) are also reported.

b Not determined.

The role of a β-turn is to generate an inversion of the protein chain direction of about 180° and to stabilize the two parallel chains. For this reason, it was also important to investigate with similar tools the effect of the chain elongation on **Hyd-loop I** and **Hyd-loop II**. Two representative compounds were selected for the two different loops, namely compounds **8** and **14**. Briefly, as predicted, both structures could generate the turn in the 41–53% of cases with little differences between **Hyd-loop I** and **Hyd-loop II** and between in vacuo an in-water environments. Similarly, from DFT calculations, both compounds showed a great preference for the β-turn conformation (see the Supporting Information for details).

**Figure 7:**
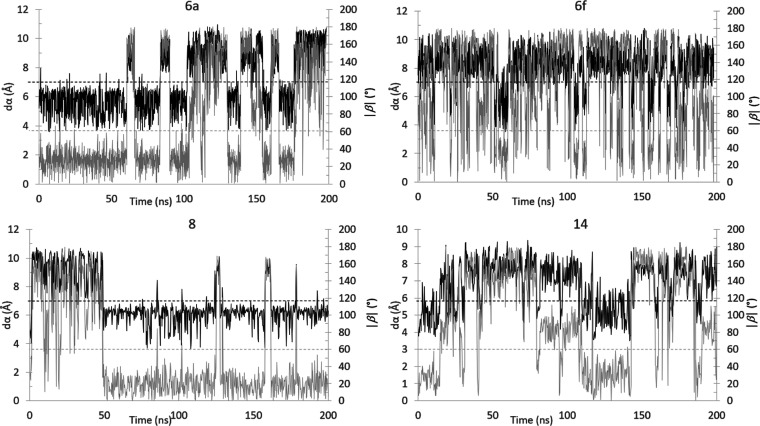
Plots of the *d*α (black line) and |β| (gray line) values vs time. The accepted limit values are also indicated as dotted black (*d*α < 7 Å) and gray (|β| < 60°) lines.

To further assess the stability of the turn conformation, we decided to further investigate the **Hyd-loop I** and **Hyd-loop II** by MD (Figure 7). Compounds **6a,f**, **8**, and **14** were selected as reference for the two Hyd-loop types both in the minimal and the peptidomimetic forms. The most stable β-turn conformations, as obtained from MM–MC, were therefore submitted to 200 ns of MD simulation in water. The value of *d*α and β and the presence of the intramolecular H-bond were analyzed through the simulation. In Figure 7, the plots of *d*α and |β| vs time are reported. As predicted, there is a strong correlation between these two parameters: a change in the *d*α value beyond the limit value of 7 Å is associated with an increase of the |β| over the 60° limit. Remarkably, from this analysis, a great difference between **Hyd-loop I** and **Hyd-loop II** was revealed. Compounds **6a** and **8** (**Hyd-loop I**) showed a greater tendency toward the β-turn conformation, which was rather stable during the dynamic; moreover, a sharp switch to the less favored open conformation is found. For compounds **6f** and **14**, the β turn is very unstable, and once this conformation is lost, its recovery is not easy. These findings are in good agreement with the results from MM–MC and DFT, according to which **Hyd-loop II** is less disposed to stabilize the β-turn conformation, especially in a water environment.

Finally, the comparison to classical β-turn types (I, I′, II, and II′) has been made by superimposition of the amide backbones with compound **6a**. The best pairing was found with a type I turn with an acceptable root-mean-square deviation (rmsd) = 0.74 Å (type II rmsd = 1.04 Å; type I′ rmsd = 1.25 Å; type II′ rmsd = 1.09 Å)

In conclusion, from computational investigation, it can be assumed that both the **Hyd-loop I** and **Hyd-loop II** scaffolds are able to achieve the desired β-turn conformation, but this tendency is more significant for **Hyd-loop I**, especially when water is considered as the solvent.

### Conformational Analysis: X-ray

#### Single-Crystal X-ray Structure of **6a**

**Figure 8:**
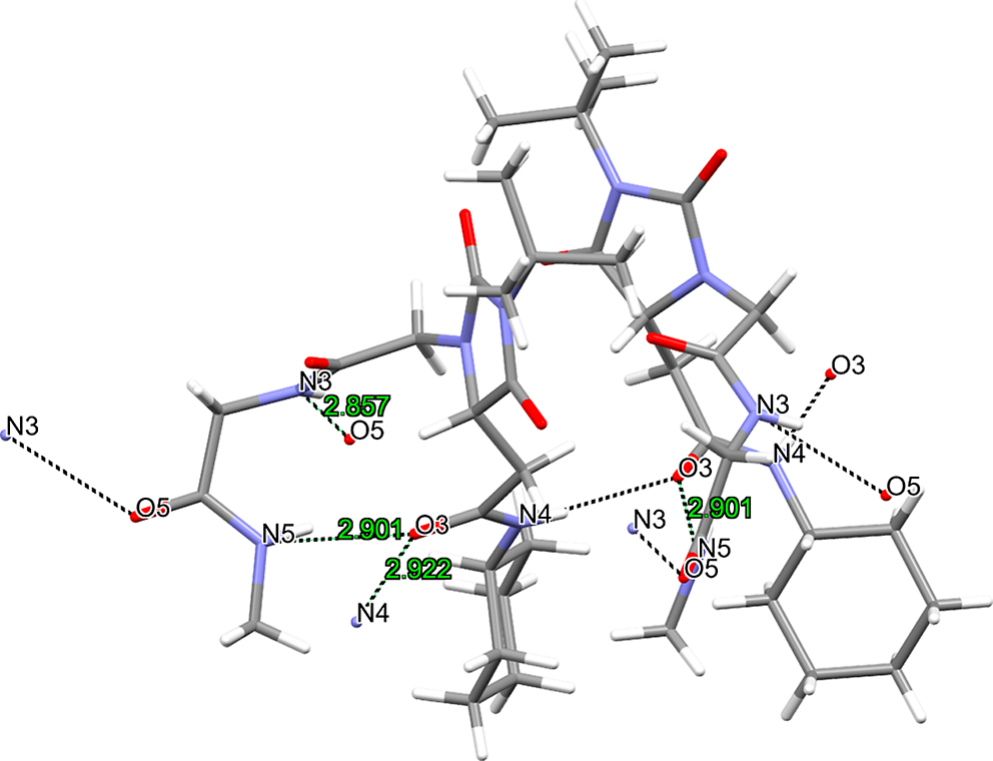
Intermolecular hydrogen bonds (black dashed lines) among two **6a** molecules. Distances in Å.

A colorless single crystal of **6a** suitable for single-crystal X-ray diffraction (SC-XRD) was obtained by evaporation from AcOEt/MeOH at room temperature. During the crystal handling, no crystal degradation was noticed. The diffraction data was recorded at a synchrotron facility (ALBA, Barcelona) at 100 K. Compound **6a** crystallizes in the orthorhombic noncentrosymmetric space group *Pna*2_1_ and contains one **6a** molecule in the asymmetric unit. In the solid-state, **6a** establishes both intermolecular and intramolecular H-bonds. The intramolecular interaction is among the N5 and O3 atoms with the distance and angle: O3···N5: 2.901(1) Å and 152(4)°. The two intermolecular distances are present in the solid-state, which involve the N and O atoms in the O3···N5: 2.857(4) Å, 158(5)° and O3···N4: 2.922(4) Å, 175(7)°. The described H-bonds are shown below in Figure 8.

Notably, also in the solid-state, compound **6a** possessing **Hyd-loop I** exists in a well-defined, H-bond-triggered β-turn conformation, even if the amidic NH involved in the intramolecular H-bond is not NH_B_ as foreseen by ^1^H NMR experiments and computation analysis but NH_D_ generating a 12-membered ring.

**Figure 9:**
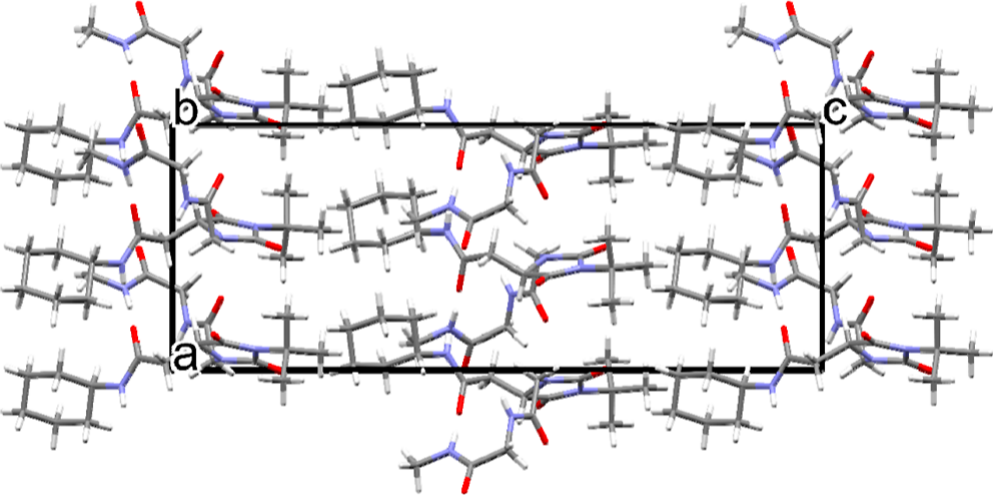
Crystal packing in **6a** viewed along the *b*-axis.

The crystal packing viewed along the *b*-axis is shown below in Figure 9. In the structure, no solvent of crystallization is observed, indicating that the lattice energy of **6a** is quite important, and the mutual fit of **6a** molecules is optimal, not leaving space for solvent to be included.

#### Single-Crystal X-ray Structure of **6f**

**Figure 10:**
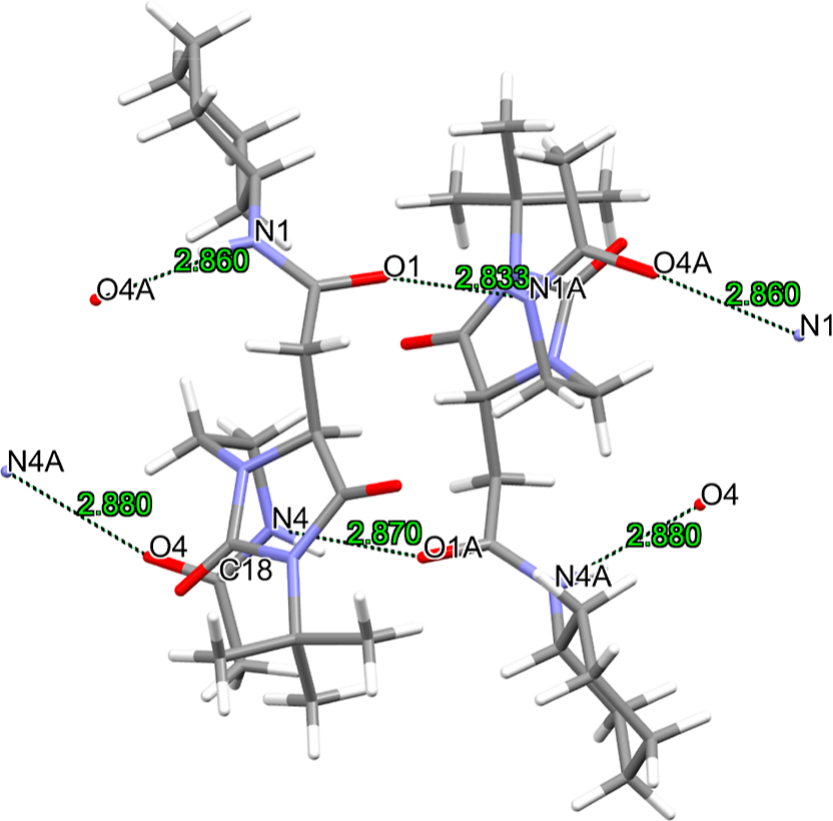
Intermolecular H-bonds (black dashed lines) among two **6f** molecules. Chloroform molecules have been removed for the sake of clarity. Distances in Å.

Single crystals of **6f** suitable for SC-XRD experiments were obtained by slow evaporation from chloroform at room temperature conditions. The SC-XRD data was recorded at the synchrotron (Elettra, Trieste) at 100 K. Compound **6f** crystallizes in the monoclinic space group *P*2_1_/*n* and contains two **6f** molecules and two chloroform solvent molecules in the asymmetric unit. One molecule of **6f** interacts with adjacent **6f** molecules via four intermolecular H-bonds involving the C=O and N–H groups (Figure 10) with the distances and angles: O1A···N4: 2.870(1) Å and 151(6)°; O4···N4A: 2.880(1) Å and 169(4)°; O1···N1A: 2.833(1) Å and 155(4)°; N1···O4A: 2.860(1) Å and 164(4)°. The H-bonds create ribbons of **6f** molecules.

No intramolecular H-bond is observed in the solid state, confirming the minor tendency of **Hyd-loop II** to adopt H-bond-triggered β-turn conformation.

**Figure 11:**
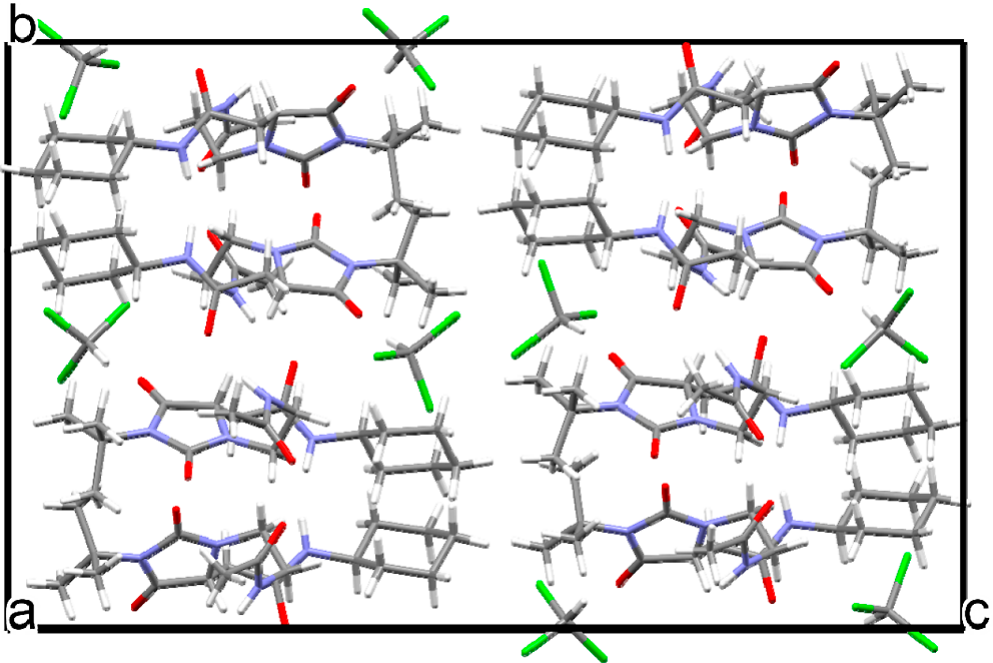
Crystal packing of **6f** viewed along the *a*-axis including solvent CHCl_3_.

Chloroform molecules are stabilized in the crystal lattice via weak electrostatic interactions O2···H1S–C1S: 3.086(1) Å. The packing of **6f** is shown in Figure 11, where the CHCl_3_ solvent molecules form layers separating the layers formed of two **6f** molecules. The **6f** crystals including chloroform are rather stable at room temperature conditions since we did not observe any crystal decay after the chloroform solution of **6f** was evaporated completely in the crystallization process.

## Conclusions

In conclusion, we synthesized a collection of racemic peptidomimetics based on two different loops, namely **Hyd-loop I** and **Hy-loop II**, having the structure of Asn embedded into a rigid hydantoin five-membered ring, a flexible glycine or an even more flexible ethylenediamine residue tethered to the Nα of Asn (or N1 of the hydantoin ring), respectively, and two strands of different lengths. The synthesis is straightforward, exploiting a sequential MC domino process followed by deprotection/coupling strategy based on acid/base liquid–liquid purification procedures, thus not requiring further chromatographic purification steps of the intermediates, which is suitable for the possible synthesis of large libraries of such compounds. The tendency to trigger β-turn conformation in vacuo, through MC–MM and Ab initio computations, in solution, through ^1^H NMR experiments, and in solid-state, through X-ray analysis, have been performed. ^1^H NMR experiments showed that **Hyd-loop I** can trigger a β-turn conformation in solution due to the presence of a quite strong intramolecular hydrogen bond involving amidic NH_B_, regardless of the length of the upper and lower strands. This result is corroborated by molecular modeling that showed that this framework can adopt favored intramolecular H-bond-driven conformations mimicking the β-turn. Quite surprisingly, in solid-state, the β-turn conformation resists even if triggered by a different intramolecular H-bond involving the amidic NH_D_. Also, more flexible **Hyd-loop II** can induce the formation of β-turn conformation even if to a lesser extent. Indeed, from molecular modeling, ^1^H NMR analysis, and X-ray, it seems that the β-turn conformation is favored only when the two peptide-like strands are elongated, in which case, amidic hydrogens NH_A_ and NH_B′_ could be involved in the formation of intramolecular H-bond favoring the arrangement of the two strands in parallel chains. All these features demonstrate that **Hyd-loop I** and **Hyd-loop II**, designed in this work to embed Asp motif in a rigid hydantoin scaffold, could be considered as novel frameworks to promote a more (**Hyd-loop I**) or less (**Hyd-loop II**) robust folded β-turn conformation. The synthesis of nonracemic, enantiomerically pure **Hyd-loop I-** and **Hyd-loop II**-based peptidomimetics and their biological application are ongoing in our laboratories.

## Materials and Methods

### Materials

Commercially available reagent-grade solvents were employed without purification. Compound **4** was synthesized as described in ref (8). Azides **1** and isocyanates **2** are commercially available. Thin-layer chromatography (TLC) was run on silica gel 60 F254 Merck. Visualization of the developed chromatogram was achieved with UV light and ceric ammonium molybdate (CAM) or ninhydrin stains. Flash chromatography (FC) was performed with silica gel 60 (60–200 μm, Merck). ^1^H-, and ^13^C NMR spectra were run at 400 MHz. Chemical shifts are expressed in ppm (δ), using tetramethylsilane (TMS) as the internal standard for ^1^H and ^13^C nuclei (δH and δC = 0.00). Structural assignments were made with additional information from gradient COSY (gCOSY) experiments. Electrospray ionization (ESI) mass spectroscopy was performed by a Bruker Esquire 3000+ instrument equipped with an MS detector composed by an ESI ionization source and a single quadrupole mass selective detector or by an Agilent Technologies 1200 Series HPLC system equipped with a diode array detector (DAD) and a 6120 MS detector composed by an ESI ionization source and a single quadrupole mass selective detector.

### Computational Details

Conformational analysis was performed with the software Spartan’08 [Y. Shao, L. F. Molnar, Y. Jung, J. Kussmann, C. Ochsenfeld, S. T. Brown, Jr. R. A. DiStasio, Phys. Chem. Chem. Phys., 8, 3172–3191 (2006)] by means of the “conformer distribution” function, using the MC search method. The Merck molecular force field (MMFF) in vacuo and MMFaq force field were used for the energy minimization of the found structures. The structures were then clustered according to the default setting of the software (which prunes out higher-energy conformers, keeping a diverse set of the low-energy conformers using the rms-torsion definition of nearness). Full geometry optimizations were performed with DFT at the B3LYP 6-31+G (d,p) level in vacuo. All energies were corrected by adding the ZPE as obtained by frequency calculation at the same level. MD simulations were realized with the Yasara program using the AMBER14 force field. The structures were analyzed through a 200 ns simulation in water at pH 7.4 and in physiological ion concentration (0.9% NaCl).

### X-ray Analysis

**6a** was recorded using high-resolution synchrotron X-ray radiation at the Alba-Cells synchrotron (Barcelona) center. The wavelength used in the experiments was λ = 0.82656 Å. Data were indexed, integrated, and scaled using the xia2 program^19^ with the DIALS pipeline for small molecule^20^ at the B13-XALOC Macromolceular Crystallography beamline. The structure was determined using direct methods (SHELXTL 97) and refined (based on F2 using all independent data) by full-matrix least-squares methods (SHELX 2014).^21^ All non-hydrogen atoms were located from different Fourier maps and refined with anisotropic displacement parameters. Hydrogen atoms were added in riding positions.

The single-crystal X-ray data for **6f** have been recorded at the Elettra Synchrotron facility (Italy). Single crystals of **6f** dipped in NVH oil (Jena Bioscience, Jena, Germany), mounted at room temperature on Kapton loops (MiTeGen, Ithaca, USA) and flash-frozen in liquid nitrogen. Two data sets were recorded, and only the best data quality sample was used for refinement and structural analysis.

**6f** has been characterized at the XRD1 beamline^22^ of the Elettra synchrotron, Trieste (Italy), through X-ray diffraction and the rotating crystal method. During data collection, the sample has been kept at 100 K via a gas nitrogen stream (Oxford Cryostream 700—Oxford Cryosystems Ltd., Oxford, United Kingdom). The diffraction images were acquired using a monochromatic wavelength of 0.700 Å and a Pilatus 2 M area detector (DECTRIS Ltd., Baden-Daettwil, Switzerland) for a total rotation of 180°.

The raw diffraction data were indexed and integrated using XDS.^23^ The structure was solved by the dual space algorithm implemented in SHELXT.^24^ Fourier analysis and refinement were performed by the full-matrix least-squares methods implemented in SHELXL,^21^ based on F2.

### General Procedure for the MC Synthesis of Intermediates **6**

To a stirred solution of azide **1** (2.5 mmol, 1.0 equiv) and isocyanate **2** (2.5 mmol, 1.0 equiv) in CH_3_CN (0.1 M), solid Ph_3_P (2.7 mmol, 1.1 equiv) was added, and the resulting solution was stirred overnight. 2,4,6 Trimethylpyridine (TMP, 2.7 mmol, 1.1 equiv) followed by acid **3** (2.7 mmol, 1.1 equiv) were added at room temperature. Once intermediate **4** is formed (3–4 h, TLC monitoring), the amine component (2.7 mmol, 1.1 equiv) is added, and the mixture stirred at room temperature overnight. The solution was diluted with AcOEt and washed with a 1 M aqueous HCl solution, brine, a saturated aqueous solution of NaHCO_3_, and brine once again. The combined organic layers were dried on Na_2_SO_4_, filtered, and the organic solvent evaporated. The crude was purified by FC affording compounds **7** in 67–81% yields.

#### 2-(1-(*tert*-Butyl)-3-(2-((2-(methylamino)-2-oxoethyl)amino)-2-oxoethyl)-2,5-dioxoimidazolidin-4-yl)-*N*-cyclohexylacetamide, **6a**

White solid. Yields: 75% (428 mg); *R*_f_ (AcOEt/MeOH, 95:5) = 0.46; ^1^H NMR (400 MHz, CDCl_3_): δ 8.02 (t, *J* = 6.0 Hz, 1H), 6.80 (br s, 1H), 5.74 (d, *J* = 8.0 Hz, 1H), 4.19 (d, *J* = 16.8 Hz, 1H), 4.14–4.12 (m, 1H), 4.08 (dd, *J* = 16.8 and 6.0 Hz, 1H), 3.85 (dd, *J* = 16.8 and 6.0 Hz, 1H), 3.80 (d, *J* = 16.8 Hz, 1H), 3.69–3.66 (m, 1H), 2.83 (d, *J* = 4.8 Hz, 3H), 2.79 (dd, *J* = 15.6 and, 3.2 Hz, 1H), 2.66 (dd, *J* = 15.6 and 5.6 Hz, 1H), 1.86–1.84 (m, 2H), 1.74–1.71 (m, 2H), 1.62–1.60 (m, 2H), 1.60 (s, 9H), 1.34–1.32 (m, 2H), 1.17–1.14 (m, 2H); ^13^C{^1^H} NMR (101 MHz, DMSO-*d*_6_): δ 173.8, 169.4, 168.5, 167.9, 157.9, 57.1, 57.0, 48.0, 44.2, 42.4, 35.6, 32.7, 32.6, 28.7, 25.9, 25.7, 24.9; ESI *m*/*z*: 424.5 [M + H, (21)]^+^, 446.6 [M + Na, (100)]^+^; Anal. Calcd for C_20_H_33_N_5_O_5_: C, 56.72; H, 7.85; N, 16.54. Found: C, 56.71; H, 7.85; N, 16.53.

#### 2-(1-(*tert*-Butyl)-3-(2-((2-(methylamino)-2-oxoethyl)amino)-2-oxoethyl)-2,5-dioxoimidazolidin-4-yl)-*N*-(4-methoxybenzyl)acetamide, **6b**

Gray solid. Yields: 81% (367 mg); *R*_f_ (AcOEt/MeOH, 95:5) = 0.52; ^1^H NMR (400 MHz, CDCl_3_): δ 7.78 (t, *J* = 6.4 Hz, 1H), 7.16 (d, *J* = 8.4 Hz, 2H), 6.90 (br q, *J* = 4.4 Hz, 1H), 6.85 (d, *J* = 8.4 Hz, 2H), 6.79 (t, *J* = 6.0 Hz, 1H), 4.29 (d, *J* = 5.6 Hz, 2H), 4.15 (m, 1H), 4.05 (d, *J* = 17.2, 1H), 3.86–3.80 (m, 2H), 3.79 (s, 3H), 2.86 (dd, *J* = 16.0 and 3.2 Hz, 1H), 2.75 (d, *J* = 4.4 Hz, 3H), 2.70 (dd, *J* = 16.0 and 6.0 Hz, 1H), 1.59 (s, 9H); ^13^C{^1^H} NMR (101 MHz, CDCl_3_): δ 172.7, 169.7, 169.0, 168.7, 159.2, 158.4, 129.6, 129.1, 114.1, 58.5, 57.7, 55.3, 46.1, 43.3, 42.8, 35.5, 28.5, 26.1; ESI *m*/*z*: 462.3 [M + H, (3)]^+^, 484.3 [M + Na, (100)]^+^; Anal. Calcd for C_22_H_31_N_5_O_6_: C, 57.25; H, 6.77; N, 15.17. Found: C, 57.24; H, 6.75; N, 15.16.

#### 2-(3-(*tert*-Butyl)-2,4-dioxo-5-(2-oxo-2-(*p*-tolylamino)ethyl)imidazolidin-1-yl)-*N*-(2-(methylamino)-2-oxoethyl)acetamide, **6c**

Gray solid. Yields: 67% (412 mg); *R*_f_ (AcOEt/MeOH, 95:5) = 0.25; ^1^H NMR (400 MHz, CD_3_OD): δ 7.38 (d, *J* = 8.4 Hz, 2H), 7.11 (d, *J* = 8.4 Hz, 2H), 4.34 (dd, *J* = 6.4 and 3.6 Hz, 1H), 4.22 (d, *J* = 17.2 Hz, 1H), 3.97 (d, *J* = 17.2 Hz, 1H), 3.88 (d, *J* = 16.8 Hz, 1H), 3.77 (d, *J* = 16.8 Hz, 1H), 3.03 (dd, *J* = 16.4 and 3.6 Hz, 1H), 2.84 (dd, *J* = 16.4 and 6.8 Hz, 1H), 2.71 (s, 3H), 2.29 (s, 3H), 1.60 (s, 9H); ^13^C{^1^H} NMR (101 MHz, CD_3_OD): δ 173.6, 170.6, 169.9, 168.3, 158.6, 135.5, 128.9, 120.1, 57.8, 57.1, 44.4, 42.1, 36.0, 27.5, 24.9, 19.5; ESI *m*/*z*: 454.1 [M + Na, (100)]^+^; Anal. Calcd for C_21_H_29_N_5_O_5_: C, 58.46; H, 6.77; N, 16.23. Found: C, 58.47; H, 6.77; N, 16.22.

#### 2-(3-Benzyl (2-(1-((3s,5s,7s)-adamantan-1-yl)-3-(2-(*tert*-butoxy)-2-oxoethyl)-2,5-dioxoimidazolidin-4-yl)acetyl)glycinate), **6d**

Gummy liquid. Yields: 72% (378 mg); *R*_f_ (hexane/AcOEt, 50:50) = 0.26; ^1^H NMR (400 MHz, CDCl_3_): δ 7.33–7.28 (m, 5H), 6.70 (t, *J* = 5.6 Hz, 1H), 5.16 (s, 2H), 4.29 (dd, *J* = 7.2 and 4.0 Hz, 1H), 4.18 (d, *J* = 18.0 Hz, 1H), 4.03 (d, *J* = 5.6 Hz, 2H), 3.81 (d, *J* = 18.0 Hz, 1H), 2.85 (dd, *J* = 16.0 and 4.0 Hz, 1H), 2.63 (dd, *J* = 16.0 and 7.2 Hz, 1H), 2.41–2.38 (m, 6H), 2.11–2.08 (m, 3H), 1.73–1.63 (m, 6H), 1.43 (s, 9H); ^13^C{^1^H} NMR (101 MHz, CDCl_3_): δ 173.5, 169.5, 169.3, 168.1, 157.6, 135.1, 128.6, 128.5, 128.3, 82.3, 67.2, 60.6, 56.3, 43.9, 41.5, 39.7, 36.9, 36.1, 29.7, 28.0; ESI *m*/*z*: 576.4 [M + Na, (100)]^+^; Anal. Calcd for C_30_H_39_N_3_O_7_: C, 65.08; H, 7.10; N, 7.59. Found: C, 65.08; H, 7.11; N, 7.60.

#### *tert*-Butyl (2-(3-(*tert*-Butyl)-5-(2-(cyclohexylamino)-2-oxoethyl)-2,4-dioxoimidazolidin-1-yl)ethyl)carbamate, **6e**

White solid. Yields: 76% (456 mg); *R*_f_ (AcOEt/MeOH, 98:2) = 0.21; ^1^H NMR (400 MHz, CD_3_Cl): δ 5.82 (d, *J* = 8.0 Hz, 1H), 5.06 (br s, 1H), 4.14 (dd, *J* = 6.0 and 4.0 Hz, 1H), 3.68–3.65 (m, 1H), 3.41–3.39 (m, 1H), 3.33–3.31 (m, 1H), 3.24–3.21 (m, 2H), 2.70 (dd, *J* = 15.2 and 4.0 Hz, 1H), 2.50 (dd, *J* = 15.2 and 6.0 Hz, 1H), 1.84–1.79 (m, 2H), 1.68–1.66 (m, 2H), 1.54–1.52 (m, 10H), 1.35 (s, 9H), 1.30–1.26 (m, 2H), 1.10–1.04 (m, 3H); ^13^C{^1^H} NMR (101 MHz, CDCl_3_): δ 173.4, 167.4, 157.8, 156.2, 79.4, 58.0, 57.0, 48.6, 41.7, 37.0, 33.3, 32.9, 28.6, 28.4, 25.4, 24.8; ESI *m*/*z*: 461.2 [M + Na, (100)]^+^; Anal. Calcd for C_22_H_38_N_4_O_5_: C, 60.25; H, 8.73; N, 12.78. Found: C, 60.27; H, 8.74; N, 12.77.

#### 2-(3-(2-Acetamidoethyl)-1-(*tert*-butyl)-2,5-dioxoimidazolidin-4-yl)-*N*-cyclohexylacetamide, **6f**

White solid. Yields: 71% (236 mg); *R*_f_ (AcOEt/MeOH, 98:2) = 0.15; ^1^H NMR (400 MHz, CD_3_Cl): δ 6.75 (bs s, 1H), 5.77 (br d, *J* = 8.0 Hz, 1H), 4.12 (dd, *J* = 6.4 and 4.0 Hz, 1H), 3.68–3.65 (m, 1H), 3.47–3.45 (m, 2H), 3.36–3.32 (m, 2H), 2.70 (dd, *J* = 15.2 and 3.6 Hz, 1H), 2.50 (dd, *J* = 15.2 and 6.4 Hz, 1H), 1.89 (s, 3H), 1.84–1.82 (m, 2H), 1.67–1.63 (m, 2H), 1.52 (s, 9H), 1.32–1.25 (m, 3H), 1.08–1.04 (m, 3H); ^13^C{^1^H} NMR (101 MHz, CDCl_3_): δ 172.1, 169.7, 166.6, 157.2, 57.2, 56.7, 47.7, 41.2, 37.9, 36.1, 32.0, 31.9, 28.7, 27.6, 24.4, 23.8, 22.2,; ESI *m*/*z*: 380.4 [M + H, (100)]+; Anal. Calcd for C_19_H_32_N_4_O_4_: C, 59.98; H, 8.48; N, 14.73. Found: C, 60.00; H, 8.48; N, 14.72.

#### Benzyl (2-(3-(2-((*tert*-butoxycarbonyl)amino)ethyl)-1-(*tert*-butyl)-2,5-dioxoimidazolidin-4-yl)acetyl)glycinate, **6g**

Gummy liquid. Yields: 68% (301 mg); *R*_f_ (AcOEt/MeOH, 98:2) = 0.33; ^1^H NMR (400 MHz, CDCl_3_): δ 7.34–7.32 (m, 5H), 6.79 (br s, 1H), 5.34 (br s, 1H), 5.17 (d, *J* = 12.4 Hz, 1H), 5.13 (d, *J* = 12.4 Hz, 1H), 4.22 (dd, *J* = 6.4 and 3.2 Hz, 1H), 4.06–4.04 (m, 2H), 3.47–3.45 (m, 1H), 3.33–3.30 (m, 3H), 2.85 (dd, *J* = 16.0 and 3.6 Hz, 1H), 2.69 (dd, *J* = 16.0 and 6.0 Hz, 1H), 1.58 (s, 9H), 1.41 (s, 9H); ^13^C{^1^H} NMR (101 MHz, CDCl_3_): δ 173.9, 173.3, 169.7, 169.1, 168.0, 157.8, 156.2, 135.1, 128.6, 128.5, 128.3, 79.4, 67.2, 58.0, 56.7, 42.1, 41.7, 41.5, 40.6, 39.2, 36.4, 28.6, 28.5, 28.4, 28.3, 14.2, 13.0; ESI *m*/*z*: 527.4 [M + Na, (100)]^+^; Anal. Calcd for C_25_H_36_N_4_O_7_: C, 59.51; H, 7.19; N, 11.10. Found: C, 59.50; H, 7.20; N, 11.11.

#### 2-(3-(2-Acetamidoethyl)-1-((3s,5s,7s)-adamantan-1-yl)-2,5-dioxoimidazolidin-4-yl)-*N*-cyclohexylacetamide, **6h**

Gray solid. Yields: 71% (515 mg); *R*_f_ (AcOEt/MeOH, 98:2) = 0.36; ^1^H NMR (400 MHz, CDCl_3_): δ 7.09 (br s, 1H), 6.12 (d, *J* = 8.0 Hz, 1H), 4.15–4.13 (m, 1H), 3.73–3.70 (m, 1H), 3.46–3.39 (m, 4H), 2.75 (dd, *J* = 15.6 and 3.2 Hz, 1H), 2.55 (dd, *J* = 15.6 and 5.8 Hz, 1H), 2.39–2.37 (m, 6H), 2.11–2.09 (m, 3H), 1.97 (s, 3H), 1.89–1.87 (m, 2H), 1.72–1.68 (m, 9H), 1.36–1.31 (m, 2H), 1.17–1.14 (m, 3H); ^13^C{^1^H} NMR (101 MHz, CDCl_3_): δ 173.2, 171.0, 167.7, 158.0, 60.5, 57.4, 48.7, 42.1, 39.8, 38.8, 37.0, 36.1, 33.0, 32.8, 29.7, 25.4, 24.8, 23.1; ESI *m*/*z*: 481.3 [M + Na, (100)]^+^; Anal. Calcd for C_25_H_38_N_4_O_4_: C, 65.48; H, 8.35; N, 12.22. Found: C, 65.50; H, 8.35; N, 12.23.

#### Benzyl (2-(1-((3s,5s,7s)-adamantan-1-yl)-3-(2-((*tert*-butoxycarbonyl)amino)ethyl)-2,5-dioxoimidazolidin-4-yl)acetyl)glycinate, **6i**

Gummy liquid. Yields: 76% (491 mg); *R*_f_ (AcOEt/MeOH, 98:2) = 0.41; ^1^H NMR (400 MHz, CDCl_3_): δ 7.28–7.27 (m, 5H), 6.48 (br s, 1H), 5.23 (br s, 1H), 5.13 (d, *J* = 12.4 Hz, 1H), 5.09 (d, *J* = 12.4 Hz, 1H), 4.11–4.09 (m, 1H), 4.00–3.98 (m, 2H), 3.58–3.32 (br m, 1H), 3.30–3.28 (m, 1H), 3.23–3.21 (m, 1H), 3.01–2.84 (br m, 1H), 2.75 (dd, *J* = 16.0 and 3.6 Hz, 1H), 2.61 (dd, *J* = 16.0 and 5.6 Hz, 1H), 2.36–2.34 (m, 6H), 2.06–2.04 (m, 3H), 1.69–1.61 (m, 7H), 1.35 (s, 9H); ^13^C{^1^H} NMR (101 MHz, CDCl_3_): δ 173.4, 169.7, 169.0, 157.8, 156.2, 128.6, 128.5, 128.4, 127.0, 79.5, 67.3, 65.3, 60.4, 56.8, 42.0, 41.5, 39.8, 39.3, 38.6, 36.7, 36.2, 29.8, 28.4; ESI *m*/*z*: 583.4 [M + H, (35)]^+^, 605.5 [M + Na, (100)]^+^; Anal. Calcd for C_31_H_42_N_4_O_7_: C, 63.90; H, 7.27; N, 9.62. Found: C, 63.91; H, 7.28; N, 9.60.

### General Procedure for the Synthesis of Hyd-Loop I Peptidomimetics **8–10**

To a solution of compound **6d** (0.45 mmol, 1.0 equiv) in a 1:1 mixture of AcOEt/MeOH (0.1 M), a catalytic amount of Pd/C (0.09 mmol, 0.2 equiv) was added. The mixture was stirred under a H_2_ atmosphere until completion of the reaction (TLC monitoring). The mixture was filtered over a Celite pad, the solvent evaporated and the crude submitted to the next step without further purification. The acid obtained as described above was dissolved in DMF (0.1 M solution), the temperature cooled to 0 °C and hexafluorophosphate benzotriazole tetramethyl uronium (HBTU) (0.47 mmol, 1.05 equiv) was added. After 10 min, H–Gly–CONH–*i*Bu (0.47 mmol, 1.05 equiv) followed by diisopropylethylamine (DIPEA) (0.47 mmol, 1.05 equiv) were added, and the solution stirred at room temperature overnight. The solution was diluted with a 1 M aqueous HCl solution, and AcOEt and was extracted three times with AcOEt. The collected organic layers were washed with brine (once), a saturated aqueous solution of NaHCO_3_ (twice), and brine (twice). The combined organic layers were dried on Na_2_SO_4_, filtered, and the organic solvent evaporated affording intermediate **7**, which was used in the following step without any further purification. Compound **7** was dissolved in DCM (0.1 M solution), and TFA (30% in volume) was added. The solution was stirred until completion of the reaction (ca. 3 h, TLC monitoring). The solvents were evaporated and coevaporated twice with cyclo-hexane. The crude was dissolved in DMF (0.1 M solution), the temperature cooled to 0 °C, and HBTU (0.47 mmol, 1.05 equiv) was added. After 10 min, a solution of the amine (0.47 mmol, 1.05 equiv) dissolved in a minimum amount of DMF followed by DIPEA (0.47 mmol, 1.05 equiv) were added, and the resulting solution was stirred at room temperature overnight. The solution was diluted with a 1 M aqueous HCl solution, and AcOEt and was extracted three times with AcOEt. The collected organic layers were washed with brine (once), a saturated aqueous solution of NaHCO_3_ (twice), and brine (twice). The combined organic layers were dried on Na_2_SO_4_, filtered, and the organic solvent evaporated. The crude was purified by FC affording peptidomimetics **8–10**.

#### *tert*-Butyl 2-(3-((3s,5s,7s)-adamantan-1-yl)-5-(2-((2-(isobutylamino)-2-oxoethyl)amino)-2-oxoethyl)-2,4-dioxoimidazolidin-1-yl)acetate, **7**

Gummy liquid. Yields: 78% (234 mg); *R*_f_ (AcOEt/MeOH, 98:2) = 0.56; ^1^H NMR (400 MHz, CDCl_3_): δ 6.99 (t, *J* = 6.0 Hz, 1H), 4.25 (t, *J* = 6.0 Hz, 1H), 4.12 (d, *J* = 18.0 Hz, 1H), 3.98 (dd, *J* = 16.8 and 6.0 Hz, 1H), 3.76–71 (m, 2H), 3.02 (t, *J* = 6.4 Hz, 2H), 2.66 (d, *J* = 6.0 Hz, 2H), 2.38–2.36 (m, 6H), 2.08–2.06 (m, 3H), 1.68–1.63 (m, 8H), 1.41 (s, 9H), 0.85 (d, *J* = 6.8 Hz, 6H); ^13^C{^1^H} NMR (101 MHz, CDCl_3_): δ 173.6, 169.4, 169.0, 167.8, 157.5, 82.5, 60.5, 56.5, 47.0, 43.6, 43.4, 39.6, 36.5, 36.1, 29.7, 28.3, 28.0, 20.1, 20.0; ESI *m*/*z*: 541.4 [M + Na, (100)]^+^; Anal. Calcd for C_27_H_42_N_4_O_6_: C, 62.53; H, 8.16; N, 10.80. Found: C, 62.53; H, 8.15; N, 10.79.

#### 2-(1-((3s,5s,7s)-Adamantan-1-yl)-3-(2-((2-((4-chlorobenzyl)amino)-2-oxoethyl)amino)-2-oxoethyl)-2,5-dioxoimidazolidin-4-yl)-*N*-(2-(isobutylamino)-2-oxoethyl)acetamide, **8**

Yellowish solid. Yields: 66% (216 mg); *R*_f_ (AcOEt/MeOH, 94:6) = 0.19; ^1^H NMR (400 MHz, CDCl_3_): δ 8.07 (br t, *J* = 6.0 Hz, 1H), 7.66 (br t, *J* = 6.4 Hz, 1H), 7.61 (br t, *J* = 5.6 Hz, 1H), 5.25 (d, *J* = 8.4 Hz, 2H), 7.20 (d, *J* = 8.4 Hz, 2H), 6.53 (br t, *J* = 5.6 Hz, 1H), 4.42 (dd, *J* = 15.2 and 6.0 Hz, 1H), 4.33 (dd, *J* = 15.2 and 6.0 Hz, 1H), 4.17 (dd, *J* = 6.8 and 3.2 Hz, 1H), 4.05 (d, *J* = 16.8 Hz, 1H), 3.96 (d, *J* = 16.8 Hz, 1H), 3.93 (dd, *J* = 10.4 and 6.4 Hz, 1H), 3.86–3.72 (m, 3H), 3.03–2.98 (m, 2H), 2.83 (dd, *J* = 15.6 and 3.2 Hz, 1H), 2.63 (dd, *J* = 15.6 and 7.2 Hz, 1H), 2.36–2.34 (m, 6H), 2.09–2.07 (m, 3H), 1.99–1.67 (m, 1H), 1.67 (s, 6H), 0.88 (d, *J* = 6.8 Hz, 6H); ^13^C{^1^H} NMR (101 MHz, CDCl_3_): δ 172.9, 170.2, 169.6, 169.1, 169.0, 158.1, 136.9, 133.0, 129.0, 128.6, 60.8, 57.1, 47.1, 45.2, 43.1, 42.6, 39.7, 36.1, 29.7, 28.4, 20.1; ESI *m*/*z*: 643.5 [M + H, (87)]^+^, 665.5 [M + Na, (100)]^+^; Anal. Calcd for C_32_H_43_ClN_6_O_6_: C, 59.76; H, 6.74; N, 13.07. Found: C, 59.78; H, 6.74; N, 13.07.

#### 2-(1-((3s,5s,7s)-Adamantan-1-yl)-3-(2-((2-((2,2-diphenylethyl)amino)-2-oxoethyl)amino)-2-oxoethyl)-2,5-dioxoimidazolidin-4-yl)-*N*-(2-(isobutylamino)-2-oxoethyl)acetamide, **9**

White solid. Yields: 71% (251 mg); *R*_f_ (AcOEt/MeOH, 94:6) = 0.29; ^1^H NMR (400 MHz, DMSO-*d*_6_): δ 8.28 (br t, *J* = 5.6 Hz, 1H), 8.24 (br t, *J* = 5.6 Hz, 1H), 7.89 (br t, *J* = 5.6 Hz, 1H), 7.80 (d, *J* = 5.6 Hz, 1H), 7.28–7.26 (m, 8H), 7.19–7.17 (m, 2H), 4.19 (t, *J* = 8.0 Hz, 1H), 4.09 (t, *J* = 5.2 Hz, 1H), 4.05 (d, *J* = 17.2 Hz, 1H), 3.73–3.70 (m, 4H), 3.65–3.63 (m, 1H), 3.60–3.57 (m, 2H), 2.89 (t, *J* = 6.4 Hz, 2H), 2.72 (dd, *J* = 15.6 and 4.4 Hz, 1H), 2.61 (dd, *J* = 15.6 and 6.0 Hz, 1H), 2.34–2.32 (m, 6H), 2.07–2.05 (m, 3H), 1.67–1.64 (m, 1H), 1.64 (s, 6H), 0.82 (d, *J* = 6.8 Hz, 6H); ^13^C{^1^H} NMR (101 MHz, CDCl_3_): δ 174.0, 169.3, 169.1, 169.0, 168.5, 157.7, 143.3, 143.2, 128.9, 128.8, 128.3, 128.2, 126.8, 59.3, 56.7, 50.5, 46.5, 44.1, 43.7, 42.7, 42.3, 36.2, 35.5, 29.6, 28.5, 20.5; ESI *m*/*z*: 721.5 [M + Na, (100)]^+^; Anal. Calcd for C_39_H_50_N_6_O_6_: C, 67.03; H, 7.21; N, 12.03. Found: C, 67.04; H, 7.20; N, 12.00.

#### 2-(1-((3s,5s,7s)-Adamantan-1-yl)-3-(2-((2-((4-iodophenyl)amino)-2-oxoethyl)amino)-2-oxoethyl)-2,5-dioxoimidazolidin-4-yl)-*N*-(2-(isobutylamino)-2-oxoethyl)acetamide, **10**

Yellowish solid. Yields: 62% (186 mg); *R*_f_ (AcOEt/MeOH, 90:10) = 0.25; ^1^H NMR (400 MHz, CD_3_OD): δ 7.64 (d, *J* = 8.8 Hz, 2H), 7.45 (d, *J* = 8.8 Hz, 2H), 4.26 (dd, *J* = 5.6 and 4.8 Hz, 1H), 4.31–4.01 (m, 4H), 3.90 (d, *J* = 16.8 Hz, 1H), 3.73 (d, *J* = 16.8 Hz, 1H), 3.00 (d, *J* = 6.8 Hz, 2H), 2.88 (dd, *J* = 15.6 and 4.8 Hz, 1H), 2.79 (dd, *J* = 15.6 and 5.6 Hz, 1H), 2.46–2.45 (m, 6H), 2.11–2.09 (m, 3H), 1.87–1.85 (m, 1H), 1.76–1.74 (m, 6H), 0.89 (d, *J* = 1.6 Hz, 3H), 0.87 (d, *J* = 1.6 Hz, 3H); ^13^C{^1^H} NMR (101 MHz, CD_3_OD): δ 173.3, 170.3, 169.9, 169.3, 169.1, 157.9, 135.1, 128.6, 128.2, 127.0, 67.7, 67.3, 65.1, 60.7, 57.6, 47.0, 45.4, 43.3, 41.3, 41.2, 39.6, 36.1, 35.7, 29.7, 28.4, 20.1; ESI *m*/*z*: 721.2 [M + H, (100)]^+^; Anal. Calcd for C_31_H_41_IN_6_O_6_: C, 51.67; H, 5.74; N, 11.66. Found: C, 51.69; H, 5.72; N, 11.68.

### General Procedure for the Synthesis of Hyd-Loop II Peptidomimetic **12**, **17**

To a solution of compound **6g** (or **6i**) (0.50 mmol, 1.0 equiv) in a 1:1 mixture of AcOEt/MeOH (0.1 M), a catalytic amount of Pd/C (0.1 mmol, 0.2 equiv) was added. The mixture was stirred under a H_2_ atmosphere until completion of the reaction (TLC monitoring). The mixture was filtered over a Celite pad, the solvent evaporated, and the crude submitted to the next step without further purification. The acid obtained as described above was dissolved in DMF (0.1 M solution), the temperature cooled to 0 °C, and HBTU (0.52 mmol, 1.05 equiv) was added. After 10 min, H–Gly–CONH–PMB (0.52 mmol, 1.05 equiv) followed by DIPEA (0.52 mmol, 1.05 equiv) were added, and the solution was stirred at room temperature overnight. The solution was diluted with a 1 M aqueous HCl solution and AcOEt and was extracted three times with AcOEt. The collected organic layers were washed with brine (once), a saturated aqueous solution of NaHCO_3_ (twice), and brine (twice). The combined organic layers were dried on Na_2_SO_4_, filtered, and the organic solvent evaporated affording intermediate **11** (or **15**), which was used in the following step without any further purification. Compound **11** (or **15**) was dissolved in DCM (0.1 M solution), and TFA (10% in volume) was added. The solution was stirred until completion of the reaction (ca. 1 h, TLC monitoring). The solvents were evaporated and coevaporated twice with cyclo-hexane. The crude was dissolved in DMF (0.1 M solution), and HBTU (0.52 mmol, 1.05 equiv) followed by a solution of the amine (0.52 mmol, 1.05 equiv) dissolved in a minimum amount of DMF and DIPEA (0.52 mmol, 1.05 equiv) were added, and the resulting solution was stirred at room temperature overnight. The solution was diluted with a 1 M aqueous HCl solution and AcOEt and was extracted three times with AcOEt. The collected organic layers were washed with brine (once), a saturated aqueous solution of NaHCO_3_ (twice), and brine (twice). The combined organic layers were dried on Na_2_SO_4_, filtered, and the organic solvent evaporated. The crude was purified by FC affording peptidomimetic **12** (or **17**).

#### *tert*-Butyl (2-(3-(*tert*-Butyl)-5-(2-((2-((4-methoxybenzyl)amino)-2-oxoethyl)amino)-2-oxoethyl)-2,4-dioxoimidazolidin-1-yl)ethyl)carbamate, **11**

Gray solid. Yields: 85% (276 mg); *R*_f_ (AcOEt) = 0.26; ^1^H NMR (400 MHz, CD_3_OD): δ 7.21 (d, *J* = 8.8 Hz, 2H), 6.86 (d, *J* = 8.8 Hz, 2H), 4.35–4.29 (m, 3H), 3.94 (d, *J* = 16.8 Hz, 1H), 3.82 (d, *J* = 16.8 Hz, 1H), 3.78 (s, 3H), 3.65–3.61 (m, 1H), 3.21–3.20 (m, 3H), 2.79–2.75 (m, 2H), 1.58 (s, 9H), 1.43 (s, 9H); ^13^C{^1^H} NMR (101 MHz, CD_3_OD): δ 174.2, 170.2, 169.8, 159.0, 157.6, 157.0, 130.4, 128.6, 113.5, 78.8, 57.5, 56.1, 54.3, 42.3, 42.2, 40.4, 37.8, 35.2, 27.7, 27.4; ESI *m*/*z*: 556.5 [M + Na, (100)]^+^; Anal. Calcd for C_26_H_39_N_5_O_7_: C, 58.52; H, 7.37; N, 13.12. Found: C, 58.51; H, 7.39; N, 13.11.

#### 2-Acetamido-*N*-(2-(3-(*tert*-butyl)-5-(2-((2-((4-methoxybenzyl)amino)-2-oxoethyl)amino)-2-oxoethyl)-2,4-dioxoimidazolidin-1-yl)ethyl)acetamide, **12**

Brown solid. Yields: 72% (251 mg); *R*_f_ (AcOEt/MeOH, 90:10) = 0.28; ^1^H NMR (400 MHz, CDCl_3_): δ 8.21 (br t, *J* = 5.6 Hz, 1H), 8.19 (br t, *J* = 5.6 Hz, 1H), 7.19 (d, *J* = 8.8 Hz, 2H), 7.01 (br t, *J* = 6.4 Hz, 1H), 6.87 (d, *J* = 8.8 Hz, 2H), 6.83 (br t, *J* = 5.6 Hz, 1H), 4.44 (dd, *J* = 14.8 and 6.0 Hz, 1H), 4.30 (dd, *J* = 14.8 and 5.6 Hz, 1H), 4.05 (dd, *J* = 16.4 and 6.8 Hz, 1H), 4.00 (dd, *J* = 16.4 and 6.0 Hz, 1H), 3.93 (t, *J* = 4.4 Hz, 1H), 3.81 (s, 3H), 3.74–3.70 (m, 2H), 3.59–3.54 (m, 1H), 3.46–3.37 (m, 2H), 3.22–3.16 (m, 1H), 2.74 (d, *J* = 4.4 Hz, 2H), 2.03 (s, 3H), 1.61 (s, 9H); ^13^C{^1^H} NMR (101 MHz, CDCl_3_): δ 173.5, 171.7, 170.5, 169.8, 159.1, 158.7, 129.7, 129.0, 114.1, 57.9, 57.8, 55.3, 43.8, 43.2, 42.9, 39.8, 35.9, 28.6, 22.9; ESI *m*/*z*: 533.4 [M + H, (63)]^+^, 555.4 [M + Na, (100)]^+^; Anal. Calcd for C_25_H_36_N_6_O_7_: C, 56.38; H, 6.81; N, 15.78. Found: C, 56.40; H, 6.80; N, 15.79.

#### *N*-(2-((2-(3-((3s,5s,7s)-Adamantan-1-yl)-5-(2-((2-(isobutylamino)-2-oxoethyl)amino)-2-oxoethyl)-2,4-dioxoimidazolidin-1-yl)ethyl)amino)-2-oxoethyl)-3-phenylpropanamide, **17**

Gray solid. Yields: 72% (281 mg); *R*_f_ (AcOEt/MeOH, 80:20) = 0.15; ^1^H NMR (400 MHz, CDCl_3_): δ 8.23 (t, *J* = 5.6 Hz, 1H), 8.04 (t, *J* = 5.6 Hz, 1H), 7.27–7.24 (m, 3H), 7.21–7.19 (m, 3H), 6.62 (t, *J* = 6.0 Ha, 1H), 4.07 (t, *J* = 5.6 Hz, 1H), 3.99 (t, *J* = 5.6 Hz, 1H), 3.87 (t, *J* = 4.8 Hz, 1H), 3.76–3.72 (m, 2H), 3.48–3.40 (m, 3H), 3.18–3.12 (m, 2H), 3.06–3.04 (m, 1H), 2.98–2.94 (m, 2H), 2.67 (t, *J* = 5.6 Hz, 2H), 2.61–2.58 (m, 2H), 2.43–2.41 (m, 6H), 2.17–2.10 (m, 3H), 1.79 (septet, *J* = 6.8 Hz, 1H), 1.72–1.67 (m, 6H), 0.92 (d, *J* = 6.8 Hz, 6H); ^13^C{^1^H} NMR (101 MHz, CDCl_3_): δ 173.6, 170.4, 169.9, 169.7, 158.4, 140.5, 128.5, 128.3, 126.3, 60.2, 57.5, 47.1, 43.4, 43.3, 42.9, 39.7, 37.7, 36.2, 35.9, 31.5, 29.7, 28.5, 20.1, 20.0; ESI *m*/*z*: 636.2 [M + H, (100)]^+^; Anal. Calcd for C_34_H_48_N_6_O_6_: C, 64.13; H, 7.60; N, 13.20. Found: C, 64.13; H, 7.60; N, 13.19.

### General Procedure for the Synthesis of Hyd-Loop II Peptidomimetic **14**

Compound **6g** (0.50 mmol, 1.0 equiv) was dissolved in DCM (0.1 M solution), and TFA (10% in volume) was added. The solution was stirred until completion of the reaction (ca. 1 h, TLC monitoring). The solvents were evaporated and coevaporated twice with cyclo-hexane. The crude was dissolved in DMF (0.1 M solution), and HBTU (0.52 mmol, 1.05 equiv) followed by a solution of the amine (0.52 mmol, 1.05 equiv) dissolved in a minimum amount of DMF and DIPEA (0.52 mmol, 1.05 equiv) were added, and the resulting solution was stirred at room temperature overnight. The solution was diluted with a 1 M aqueous HCl solution and AcOEt and was extracted three times with AcOEt. The collected organic layers were washed with brine (once), a saturated aqueous solution of NaHCO_3_ (twice), and brine (twice). The combined organic layers were dried on Na_2_SO_4_, filtered, and the organic solvent evaporated affording intermediate **13**, which was used in the following step without any further purification. Compound **13** was dissolved in a 1:1 mixture of AcOEt/MeOH (0.1 M), and a catalytic amount of Pd/C (0.1 mmol, 0.2 equiv) was added. The mixture was stirred under a H_2_ atmosphere until completion of the reaction (TLC monitoring). The mixture was filtered over a Celite pad, the solvent evaporated, and the crude submitted to the next step without further purification. The acid obtained as described above was dissolved in DMF (0.1 M solution), the temperature cooled to 0 °C, and HBTU (0.52 mmol, 1.05 equiv) was added. After 10 min, a solution of the amine (0.52 mmol, 1.05 equiv) dissolved in a minimum amount of DMF followed by DIPEA (0.52 mmol, 1.05 equiv) were added, and the solution was stirred at room temperature overnight. The solution was diluted with a 1 M aqueous HCl solution and AcOEt and was extracted three times with AcOEt. The collected organic layers were washed with brine (once), a saturated aqueous solution of NaHCO_3_ (twice), and brine (twice). The combined organic layers were dried on Na_2_SO_4_, filtered, and the organic solvent evaporated. The crude was purified by FC affording **14**.

#### Benzyl (2-(3-(2-(2-Acetamidoacetamido)ethyl)-1-(*tert*-butyl)-2,5-dioxoimidazolidin-4-yl)acetyl)glycinate, **13**

White solid. Yields: 85% (301 mg); *R*_f_ (AcOEt/MeOH, 90:10) = 0.33; ^1^H NMR (400 MHz, CDCl_3_): δ 7.80 (br t, *J* = 6.0 Hz, 1H), 7.62 (br t, *J* = 6.0 Hz, 1H), 7.29–7.25 (m, 5H), 7.10 (br t, *J* = 6.0 Hz, 1H), 5.11 (d, *J* = 12.0 Hz, 1H), 5.09 (d, *J* = 12.0 Hz, 1H), 4.07 (dd, *J* = 18.0 and 6.0 Hz, 1H), 3.93 (dd, *J* = 5.6 and 3.6 Hz, 1H), 3.90–3.86 (m, 2H), 3.64 (dd, *J* = 16.4 and 6.0 Hz, 1H), 3.49–3.46 (m, 2H), 3.33–3.31 (m, 1H), 3.19–3.14 (m, 1H), 2.75 (dd, *J* = 16.4 and 3.6 Hz, 1H), 2.65 (dd, *J* = 16.4 and 5.6 Hz, 1H), 1.95 (s, 3H), 1.52 (s, 9H); ^13^C NMR (101 MHz, CDCl_3_): δ 173.4, 171.6, 171.3, 170.3, 169.6, 158.7, 134.9, 128.7, 128.6, 128.3, 67.5, 57.9, 57.6, 43.7, 42.9, 41.4, 39.6, 36.1, 28.6, 22.9; ESI *m*/*z*: 526.4 [M + Na (100)]^+^; Anal. Calcd for C_24_H_33_N_5_O_7_: C, 57.25; H, 6.61; N, 13.91. Found: C, 57.26; H, 6.61; N, 13.89.

#### 2-Acetamido-*N*-(2-(3-(*tert*-butyl)-5-(2-((2-((4-chlorobenzyl)amino)-2-oxoethyl)amino)-2-oxoethyl)-2,4-dioxoimidazolidin-1-yl)ethyl)acetamide, **14**

White solid. Yields: 72% (273 mg); *R*_f_ (AcOEt/MeOH, 80:20) = 0.24; ^1^H NMR (400 MHz, CDCl_3_): δ 8.20 (br t, *J* = 5.6 Hz, 1H), 8.11 (br t, *J* = 5.6 Hz, 1H), 7.28 (d, *J* = 8.4 Hz, 2H), 7.18 (d, *J* = 8.4 Hz, 2H), 7.06 (br t, *J* = 5.6 Hz, 1H), 6.97 (br t, *J* = 5.6 Hz, 1H), 4.46 (dd, *J* = 14.8 and 5.6 Hz, 1H), 4.30 (dd, *J* = 14.8 and 5.6 Hz, 1H), 4.05–4.97 (m, 2H), 3.91 (t, *J* = 4.8 Hz, 1H), 3.72–3.62 (m, 2H), 3.54–3.51 (m, 1H), 3.40–3.35 (m, 2H), 3.20–3.15 (m, 1H), 2.73 (dd, *J* = 16.8 and 4.8 Hz, 1H), 2.68 (dd, *J* = 16.8 and 4.8 Hz, 1H), 2.01 (s, 3H), 1.58 (s, 9H); ^13^C{^1^H} NMR (101 MHz, CDCl_3_): δ 173.5, 171.8, 170.4, 170.1, 169.9, 158.7, 136.4, 133.3, 129.0, 128.8, 57.9, 57.8, 43.8, 43.3, 42.9, 39.8, 35.8, 28.6, 22.9; ESI *m*/*z*: 560.4 [M + Na, (100)]^+^; Anal. Calcd for C_24_H_33_ClN_6_O_6_: C, 53.68; H, 6.19; N, 15.65. Found: C, 53.68; H, 6.19; N, 15.66.

### General Procedure for the Synthesis of Hyd-Loop II Peptidomimetic **16**

To a solution of compound **6i** (0.34 mmol, 0.1 equiv) in a 1:1 mixture of AcOEt/MeOH (0.1 M), a catalytic amount of Pd/C (0.07 mmol, 0.2 equiv) was added. The mixture was stirred under a H_2_ atmosphere until completion of the reaction (TLC monitoring). The mixture was filtered over a Celite pad, the solvent evaporated, and the crude submitted to the next step without further purification. The acid obtained as described above was dissolved in DMF (0.1 M solution), the temperature cooled to 0 °C, and HBTU (0.36 mmol, 1.05 equiv) was added. After 10 min, H–Gly–CONH–PMB (0.36 mmol, 1.05 equiv) followed by DIPEA (0.36 mmol, 1.05 equiv) were added, and the solution was stirred at room temperature overnight. The solution was diluted with a 1 M aqueous HCl solution and AcOEt and was extracted three times with AcOEt. The collected organic layers were washed with brine (once), a saturated aqueous solution of NaHCO_3_ (twice), and brine (twice). The combined organic layers were dried on Na_2_SO_4_, filtered, and the organic solvent evaporated affording intermediate **15**, which was used in the following step without any further purification. Compound **15** was dissolved in DCM (0.1 M solution), and TFA (10% in volume) was added. The solution was stirred until completion of the reaction (ca. 1 h, TLC monitoring). The solvents were evaporated and coevaporated twice with cyclo-hexane. The crude was dissolved in DCM (0.1 M solution) and TEA (0.41 mmol, 1.2 equiv) followed by 3-phenyl-propanoyl chloride (0.36 mmol, 1.05 equiv) were added at 0 °C, and the resulting solution was stirred at room temperature overnight. The solution was diluted with a saturated aqueous solution of NaHCO_3_ and extracted with DCM three times. The collected organic layers were washed with brine (once), 1 M aqueous HCl solution (once), and brine (twice). The combined organic layers were dried on Na_2_SO_4_, filtered, and the organic solvent evaporated. The crude was purified by FC affording peptidomimetic **16**.

#### *tert*-Butyl (2-(3-((3s,5s,7s)-adamantan-1-yl)-5-(2-((2-(isobutylamino)-2-oxoethyl)amino)-2-oxoethyl)-2,4-dioxoimidazolidin-1-yl)ethyl)carbamate, **15**

Gummy oil. Yields: 84% (216 mg); *R*_f_ (AcOEt) = 0.15; ^1^H NMR (400 MHz, CDCl_3_): δ 7.08 (br s, 1H), 6.82 (br s, 1H), 5.36 (br s, 1H), 4.16–4.14 (m, 1H), 3.89–3.79 (m, 2H), 3.42–3.40 (m, 1H), 3.22–3.20 (m, 3H), 2.99–2.98 (m, 2H). 2.66–2.64 (m, 2H), 2.33–2.31 (m, 6H), 2.04–2.02 (m, 3H), 1.68–1.66 (m, 1H), 1.62–1.60 (m, 6H), 1.36 (s, 9H), 0.81 (d, *J* = 6.8 Hz, 6H); ^13^C{^1^H} NMR (101 MHz, CDCl_3_): δ 173.7, 169.5, 169.1, 157.6, 156.3, 100.0, 79.6, 60.4, 56.5, 47.0, 43.4, 41.5, 39.7, 39.1, 38.6, 36.5, 36.1, 29.7, 29.6, 28.4, 28.3, 20.1; ESI *m*/*z*: 570.5 [M + Na, (100)]^+^; Anal. Calcd for C_28_H_45_N_5_O_6_: C, 61.40; H, 8.28; N, 12.79. Found: C, 61.42; H, 8.29; N, 12.80.

#### *N*-(2-(3-((3s,5s,7s)-Adamantan-1-yl)-5-(2-((2-(isobutylamino)-2-oxoethyl)amino)-2-oxoethyl)-2,4-dioxoimidazolidin-1-yl)ethyl)-3-phenylpropanamide, **16**

Yellowish solid. Yields: 81% (197 mg); *R*_f_ (AcOEt/MeOH, 80:22) = 0.26; ^1^H NMR (400 MHz, CDCl_3_): δ 7.20–7.15 (m, 2H), 7.11–7.09 (m, 3H), 6.94 (br s, 1H), 6.62 (br t, *J* = 6.0 Hz, 1H), 6.56 (br s, 1H), 4.08 (t, *J* = 5.2 Hz, 1H), 3.85 (dd, *J* = 16.4 and 5.6 Hz, 1H), 3.78 (dd, *J* = 16.4 and 5.6 Hz, 1H), 3.31 (br s, 4H), 2.98 (t, *J* = 6.8 Hz, 2H), 2.86–2.84 (m, 2H), 2.61 (dd, *J* = 16.0 and 5.2 Hz, 1H), 2.54 (dd, *J* = 16.0 and 6.0 Hz, 1H), 2.42–2.37 (m, 2H), 2.31–2.29 (m, 6H), 2.03–2.01 (m, 3H), 1.70 (septet, *J* = 6.8 Hz, 1H), 1.63–1.60 (m, 6H), 0.81 (d, *J* = 6.8 Hz, 6H); ^13^C{^1^H} NMR (101 MHz, CDCl_3_): δ 173.3, 172.9, 169.3, 168.7, 158.1, 140.9, 128.5, 128.3, 126.3, 60.6, 56.8, 47.0, 43.4, 41.5, 39.8, 38.8, 38.2, 36.5, 36.1, 31.5, 29.7, 28.4, 20.1; ESI *m*/*z*: 602.5 [M + Na, (100)]^+^; Anal. Calcd for C_32_H_45_N_5_O_5_: C, 66.30; H, 7.82; N, 12.08. Found: C, 66.29; H, 7.83; N, 12.09.

#### *N*-(2-((2-(3-((3s,5s,7s)-Adamantan-1-yl)-5-(2-((2-(isobutylamino)-2-oxoethyl)amino)-2-oxoethyl)-2,4-dioxoimidazolidin-1-yl)ethyl)amino)-2-oxoethyl)-3-phenylpropanamide, **17**

Yellowish solid. Yields: 72% (169 mg); *R*_f_ (AcOEt/MeOH, 80:22) = 0.29; ^1^H NMR (400 MHz, CDCl_3_): δ 8.23 (t, *J* = 5.6 Hz, 1H), 8.04 (t, *J* = 5.6 Hz, 1H), 7.27–7.25 (m, 2H), 7.21–7.19 (m, 3H), 6.62 (t, *J* = 6.0 Hz, 1H), 4.04–3.98 (m, 2H), 3.87 (t, *J* = 4.8 Hz, 1H), 3.76–3.72 (m, 2H), 3.48–3.33 (m, 3H), 3.18–3.12 (m, 2H), 3.06–2.94 (m, 3H), 2.67 (t, *J* = 5.6 Hz, 2H), 2.61–2.58 (m, 2H), 2.43–2.41 (m, 6H), 2.10 (br s, 4H), 1.79 (septet, *J* = 6.8 Hz, 1H), 1.71–1.67 (m, 6H), 0.91 (d, *J* = 6.8 Hz, 6H); ^13^C{^1^H} NMR (101 MHz, CDCl_3_): δ 173.6, 170.4, 169.9, 169.7, 158.4, 140.5, 128.5, 128.3, 126.3, 60.2, 57.5, 47.1, 43.4, 43.3, 42.9, 39.7, 37.7, 36.2, 35.9, 31.5, 29.7, 28.5, 20.1, 20.0; ESI *m*/*z*: 636.2 [M + H, (100)]^+^; Anal. Calcd for C_34_H_48_N_6_O_6_: C, 64.13; H, 7.60; N, 13.20. Found: C, 64.14; H, 7.60; N, 13.21.

## Data Availability

The data underlying this study are available in the published article and its Supporting Information.

## Supporting Information Available

Copies of the ^1^H, ^13^C NMR, and MS spectra for all new compounds, copies of the VT ^1^H NMR experiments, and data of the molecular modeling computations (PDF)
