# Supplementary material for: Turn-Mimic Hydantoin-Based Loops Constructed by a Sequential Multicomponent Reaction
Source: J Org Chem. 2023 Nov 6;88(22):15790–804. doi: 10.1021/acs.joc.3c01861 (PMC10661056; doi:10.1021/acs.joc.3c01861)
Supplement: Supplementary file 1 — jo3c01861_si_001.pdf [file jo3c01861_si_001.pdf]

SUPPORTING INFORMATION

Turn-Mimic Hydantoin-Based Loops Constructed by a Sequential Multicomponent Reaction

Alessio Maria Caramiello,[†] Maria Cristina Bellucci,[§] Javier Marti-Rujas,[†] Alessandro Sacchetti,[†,*] Alessandro Volonterio[†,*]

[†] Department of Chemistry, Material and Chemical Engineering "Giulio Natta", Politecnico di Milano, via Mancinelli 7, 20131 Milano, Italy

[§] Department of Food, Environmental and Nutritional Sciences, Università degli Studi di Milano, via Celoria 2, 20133 Milano, Italy

alessandro.volonterio@polimi.it; *alessandro.sacchetti@polimi.it*

TABLE OF CONTENTS:

S2-S51 Copies of ^{1}H NMR, ^{13}C NMR, COSY and MS spectra of all new compounds

S52-S56 VT ^{1}H NMR spectra

S57-S66 Molecular modeling computations

S66-S69 Crystal packing, crystal data and structure refinement for **6a**

S70-S74 Crystal packing, crystal data and structure refinement for **6f**

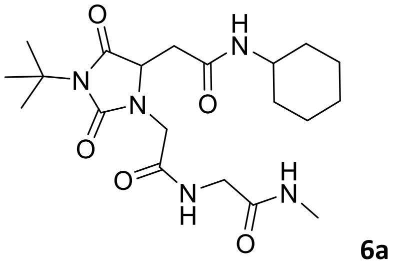

6a

^{1}H NMR (400 MHZ, $CDCl_3$)

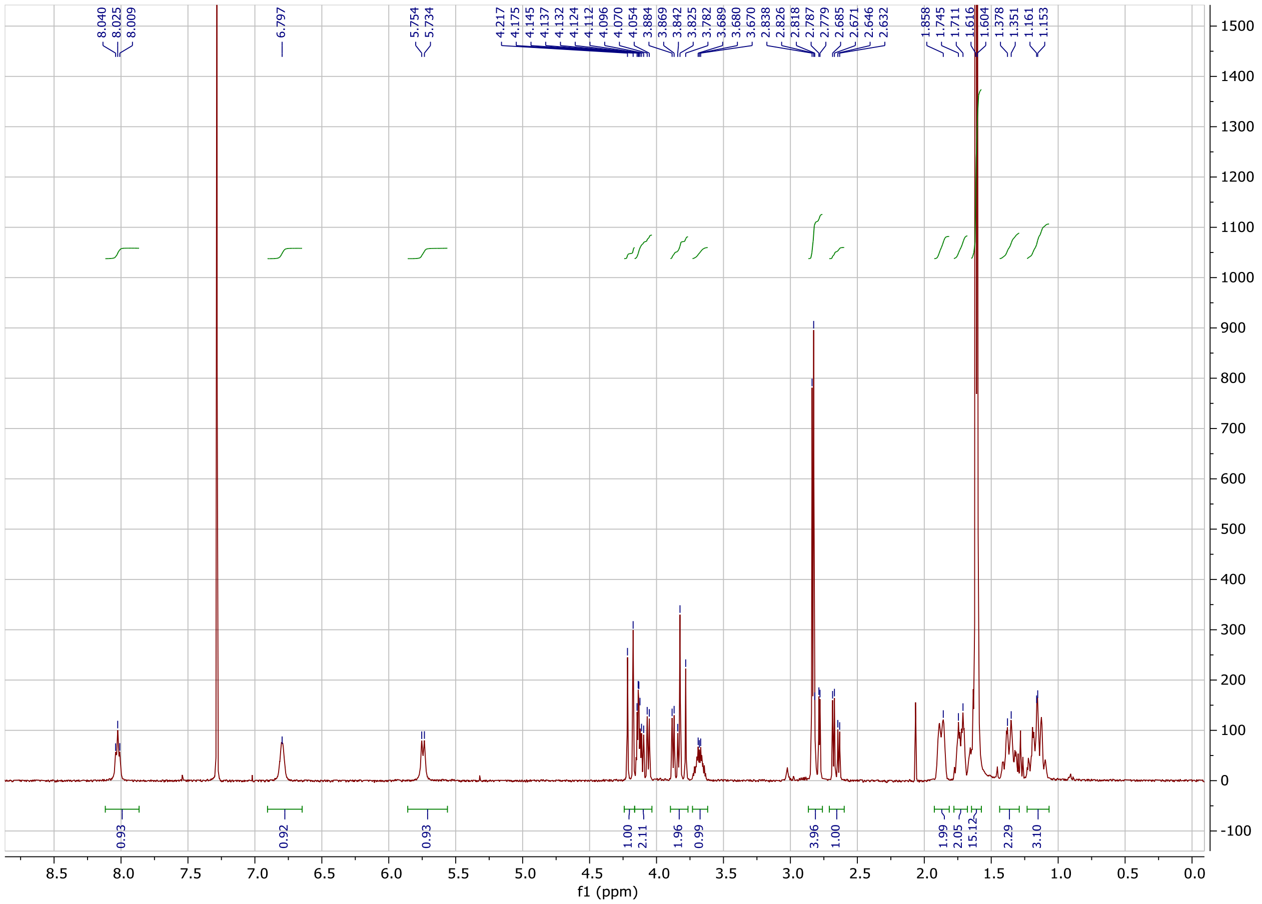

gCOSY NMR (400 MHZ, $CDCl_3$)

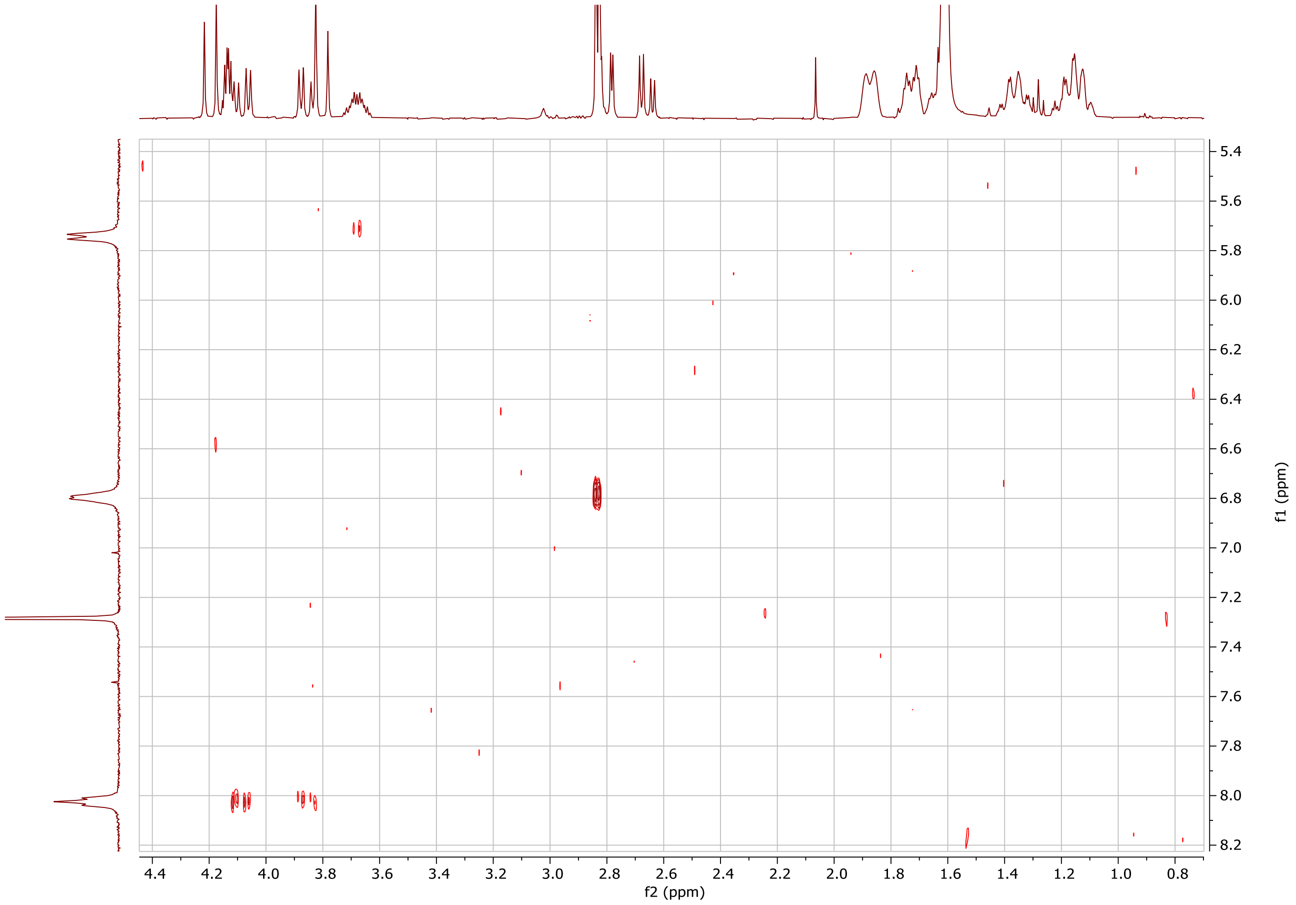

$^{13}C\{^{1}H\}$ NMR (101 MHz, dmso-d_6)

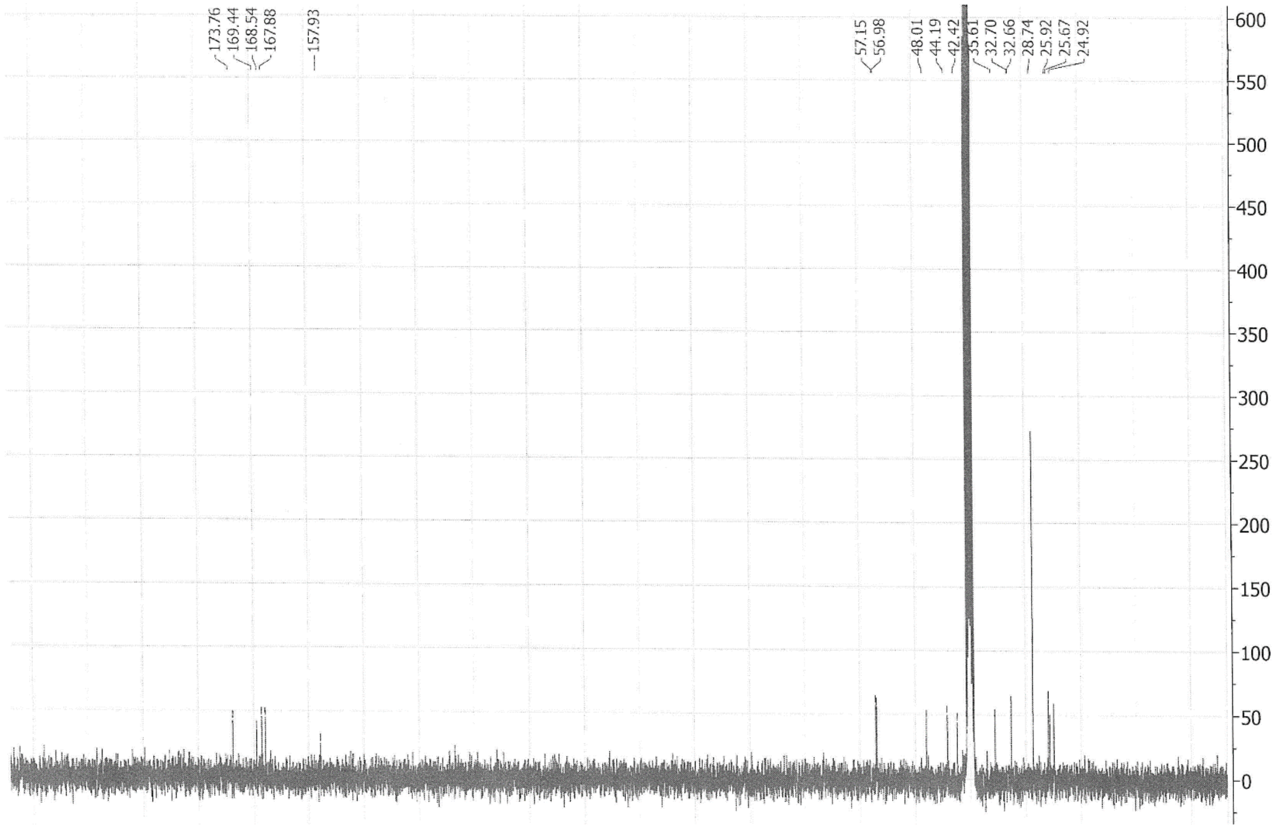

-L.G.S. - Laboratorio Grandi Strumenti - Display Report

Analysis Name	av ld38.d	Acquisition Date	02/27/13 11:51:40	Operator	Walter Panzeri
Sample Name		Method	Copy of _01tmix_posneg lm.MS	Instrument	esquire3000plus
Comment	1 mg/mL dil 1:100 MeOH<br>Richiedente: Volonterio				

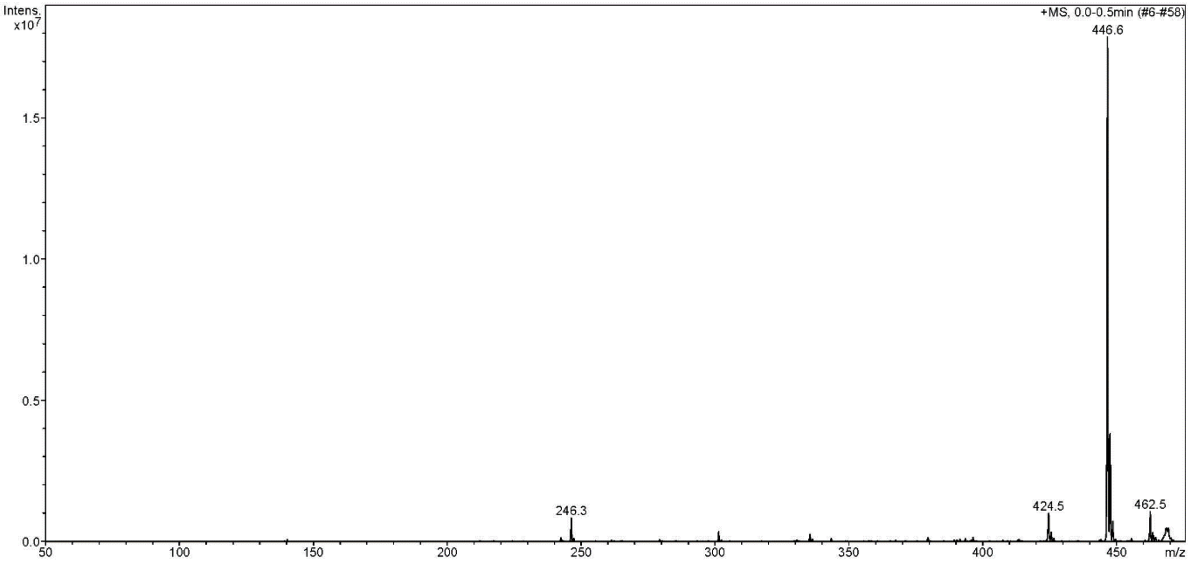

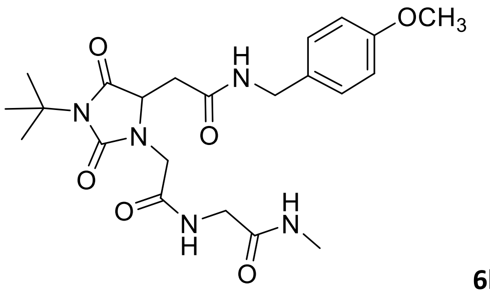

6b

^{1}H NMR (400 MHZ, $CDCl_3$)

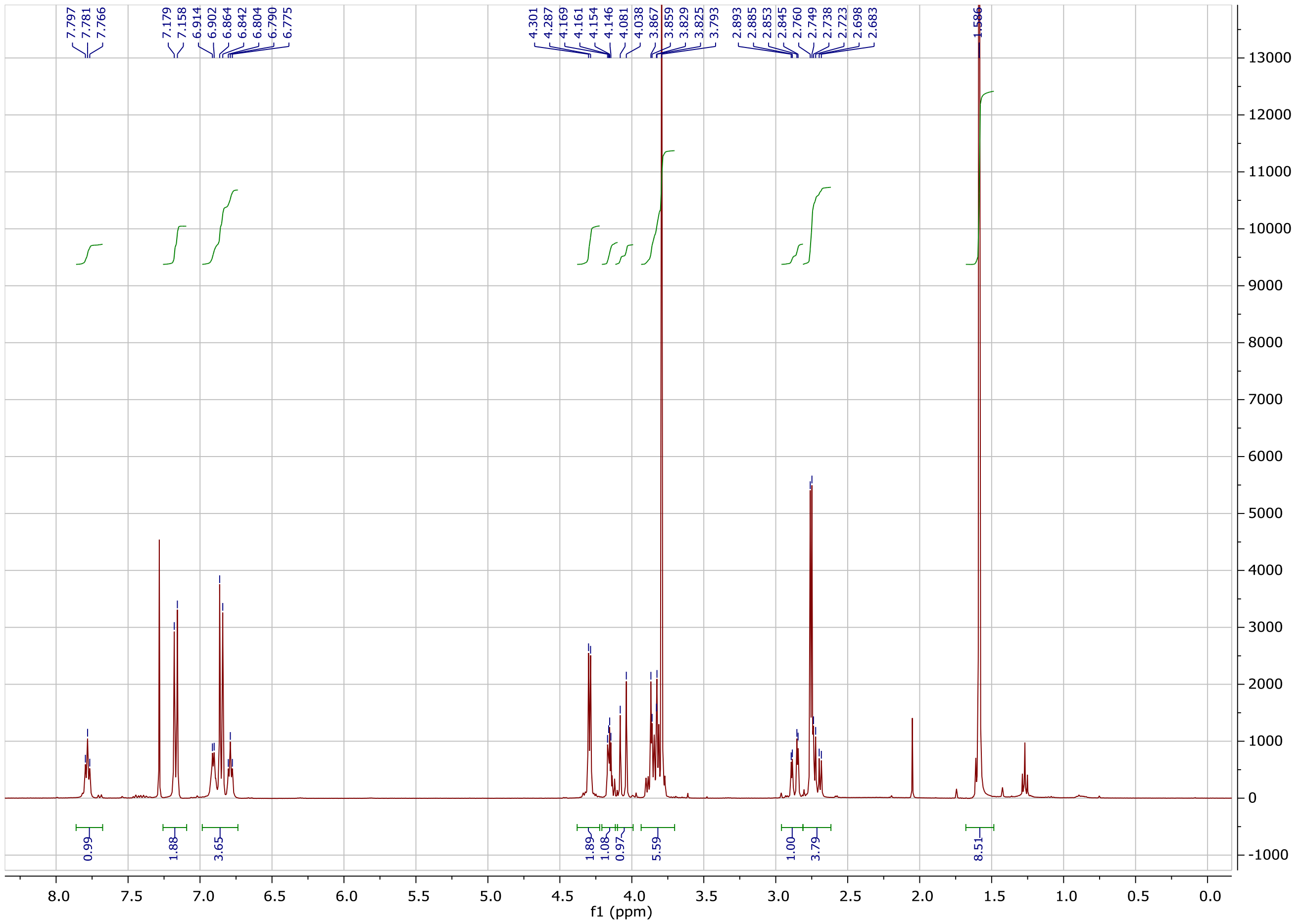

gCOSY NMR (400 MHZ, $CDCl_3$)

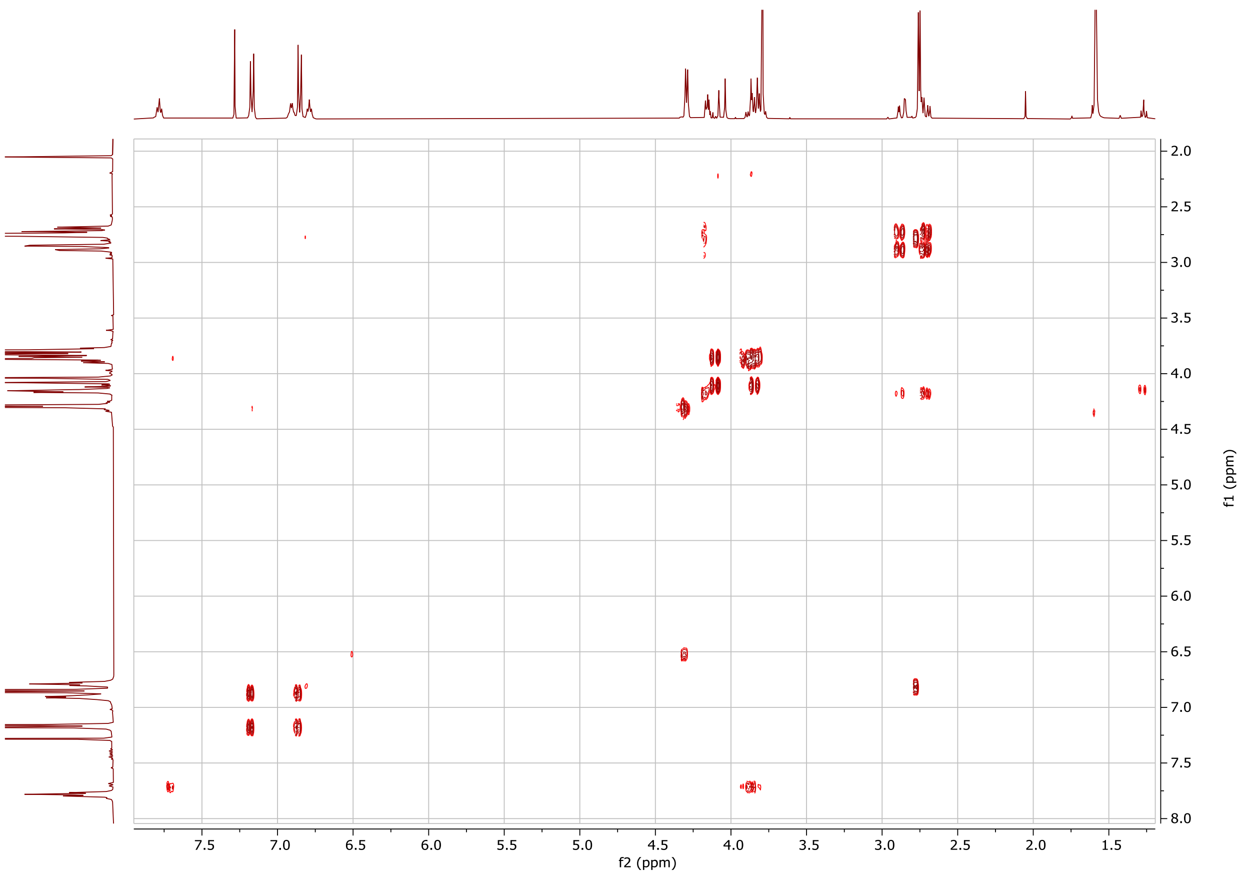

$^{13}C\{^1H\}$ NMR (101 MHz, $CDCl_3$)

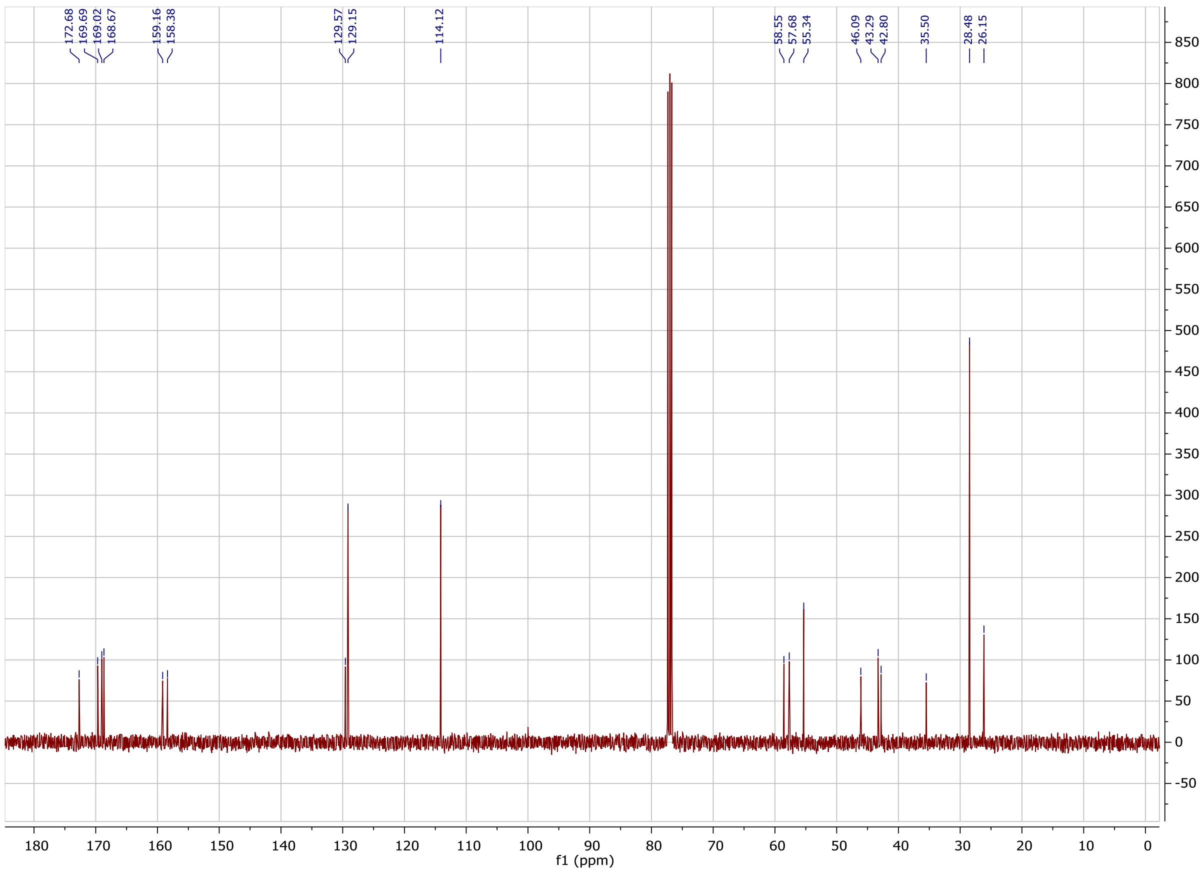

-L.G.S. - Laboratorio Grandi Strumenti - Display Report

Analysis Name	av 2905.d	Acquisition Date	05/19/21 13:26:29	Operator	Walter Panzeri
Sample Name		Method	Copy of $wp_lm.MS	Instrument	esquire3000plus
Comment	1 mg/ml dil 1:100 MeOH<br>Richiedente: Volonterio				

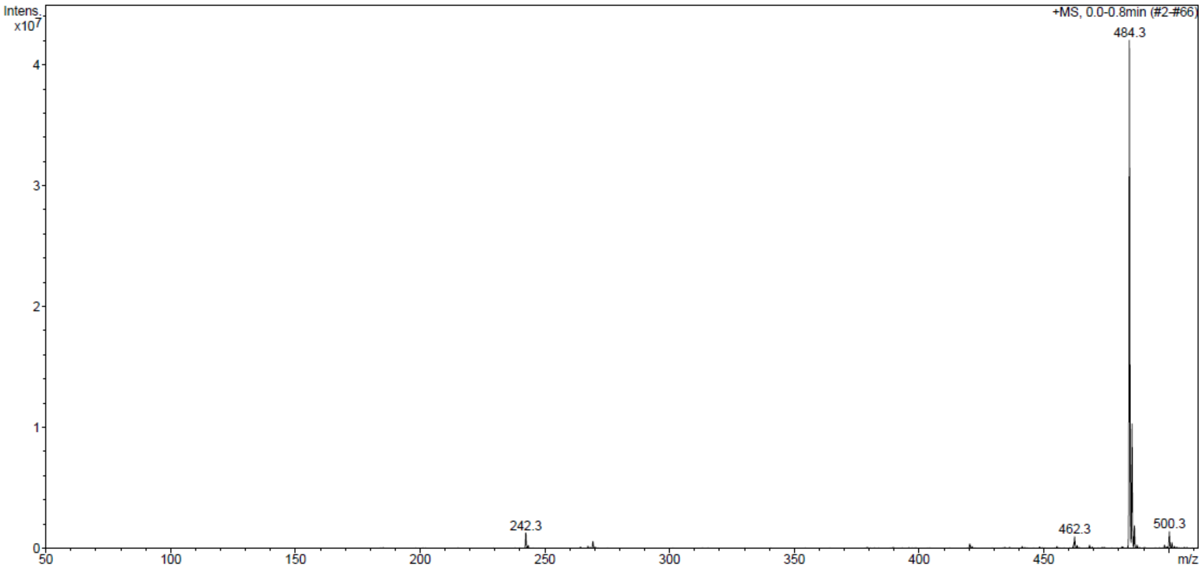

Bruker Daltonics DataAnalysis 3.1 printed: 05/19/21 13:32:57 Page 1 of 1

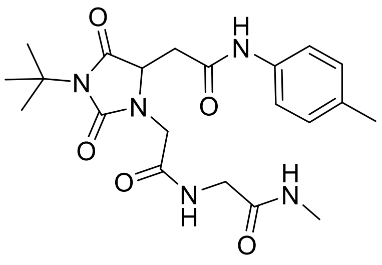

6c

^{1}H NMR (400 MHZ, CD_3OD)

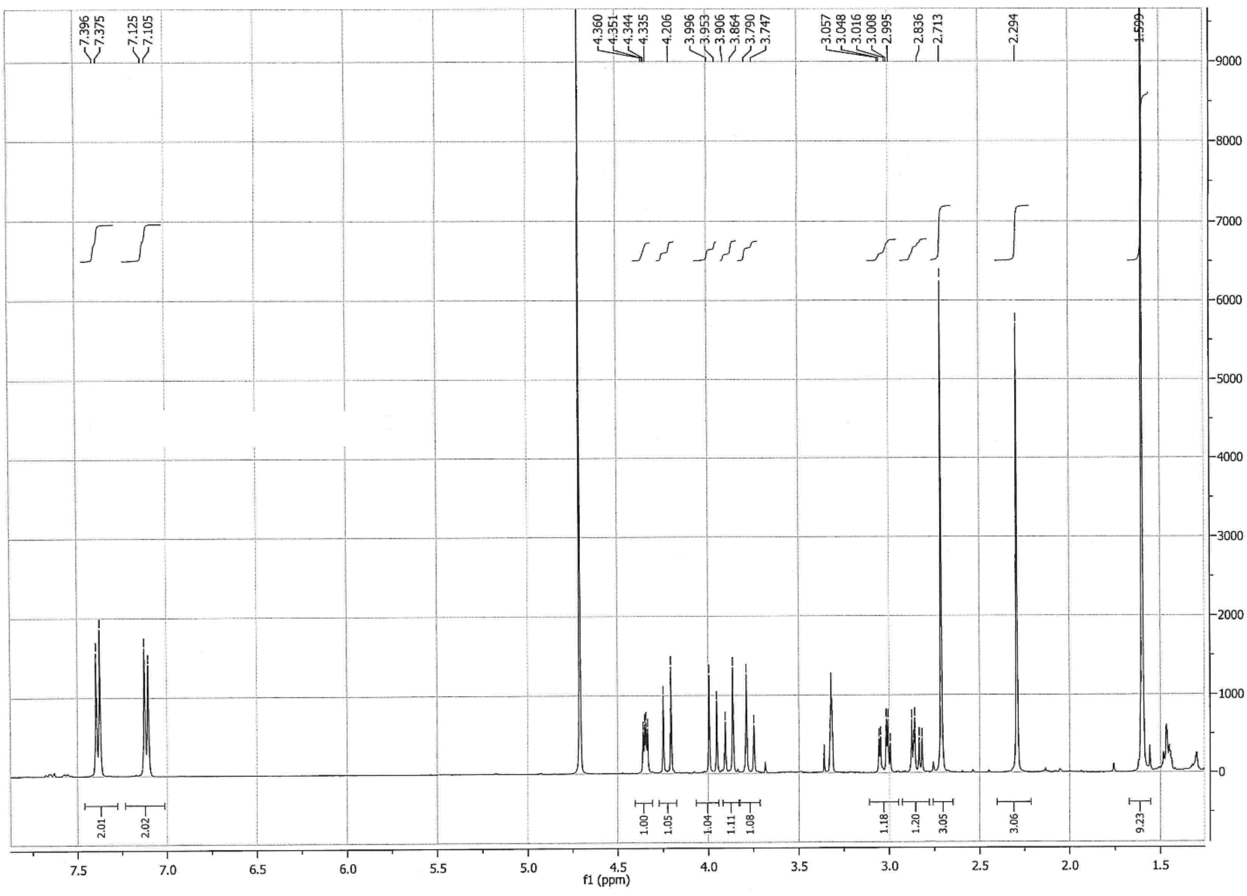

$^{13}C\{^1H\}$ NMR (101 MHz, CD_3OD)

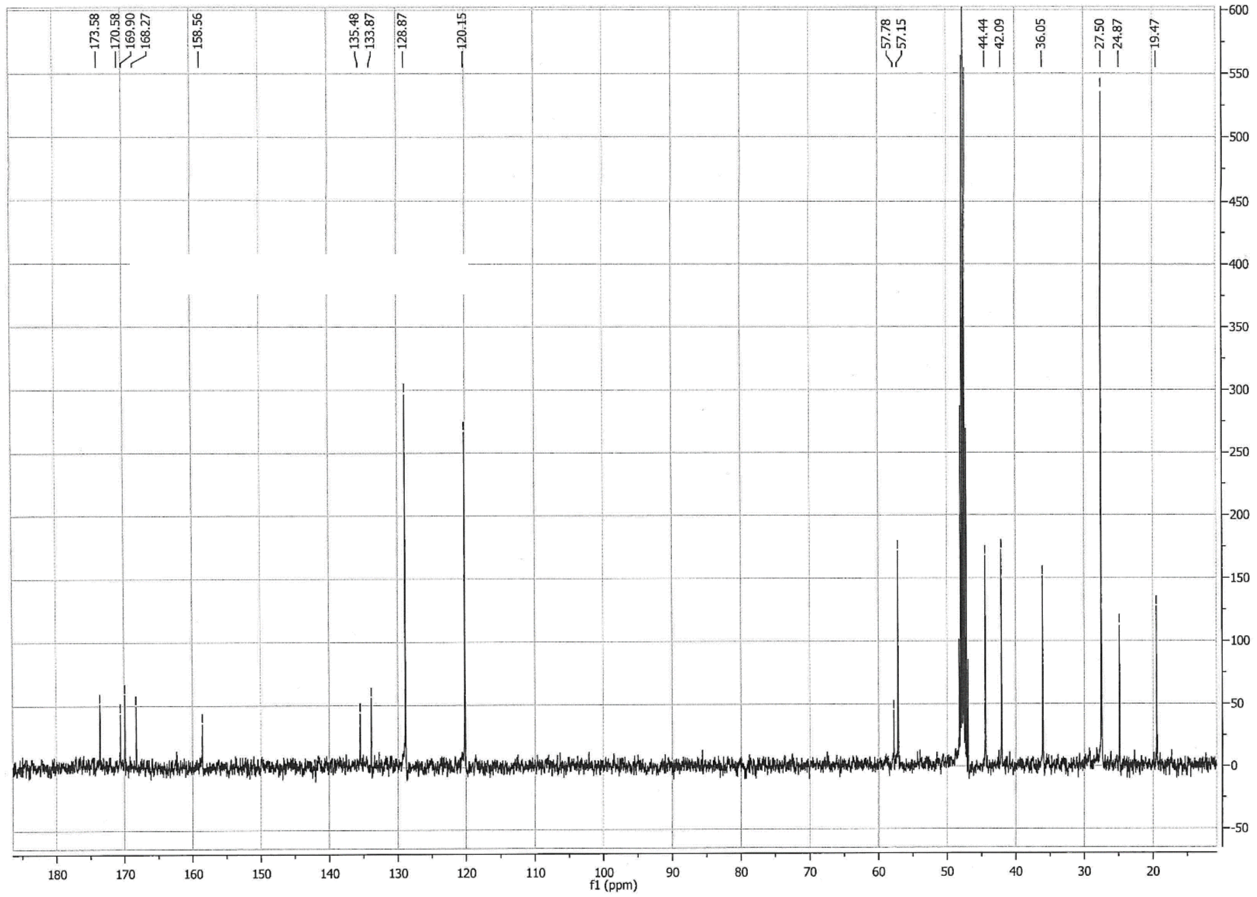

-L.G.S. - Laboratorio Grandi Strumenti - Display Report

Analysis Name	av pc41.d	Acquisition Date	07/24/14 14:38:56	Operator	Walter Panzeri
Sample Name		Method	Copy of _01esquirew t 1500.MS	Instrument	esquire3000plus
Comment	1 mg/ml dil 1:100 MeOH<br>Richiedente: Volonterio				

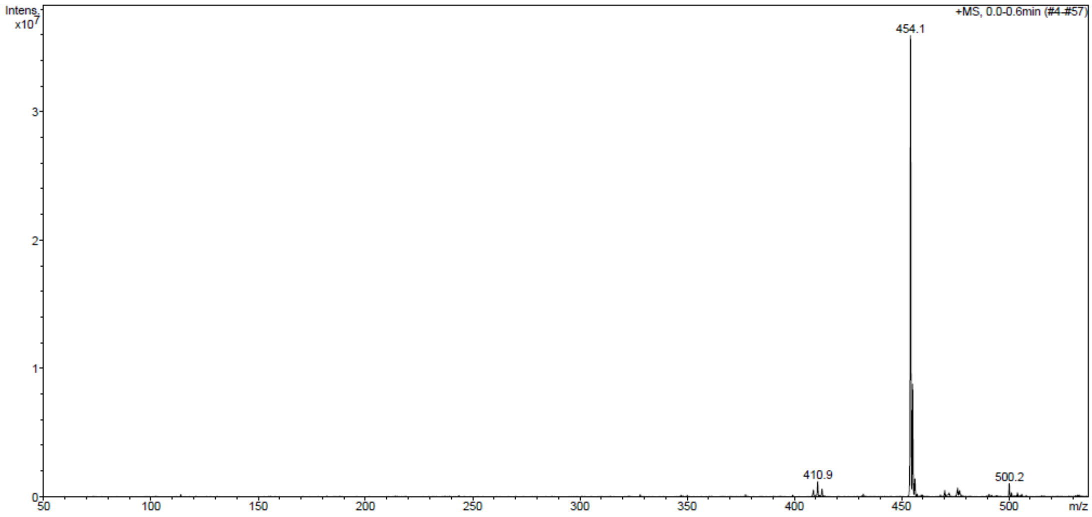

Bruker Daltonics DataAnalysis 3.1 printed: 07/24/14 14:40:04 Page 1 of 1

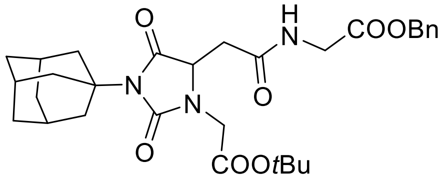

6d

^{1}H NMR (400 MHZ, $CDCl_3$)

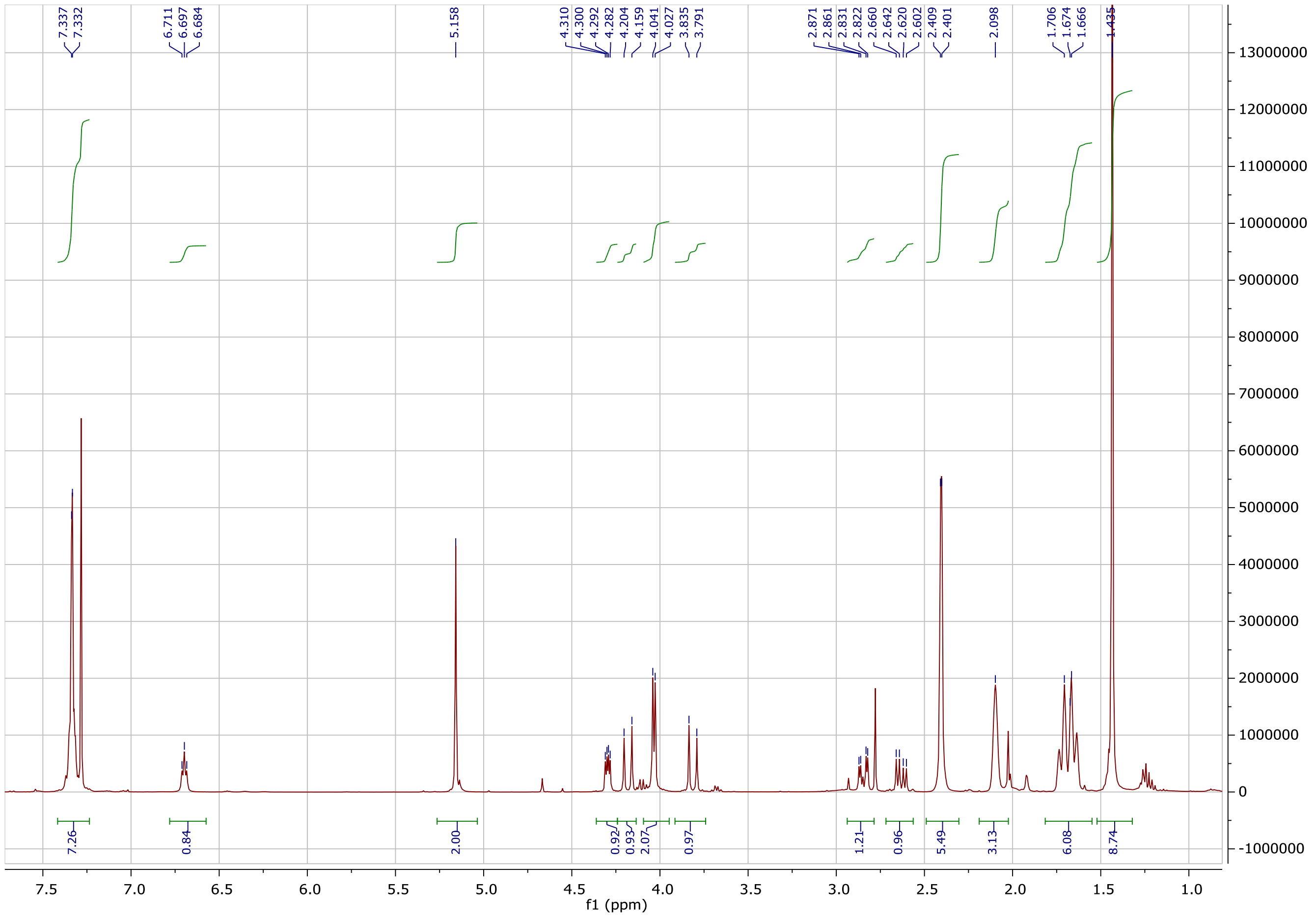

$^{13}C\{^{1}H\}$ NMR (101 MHz, $CDCl_3$)

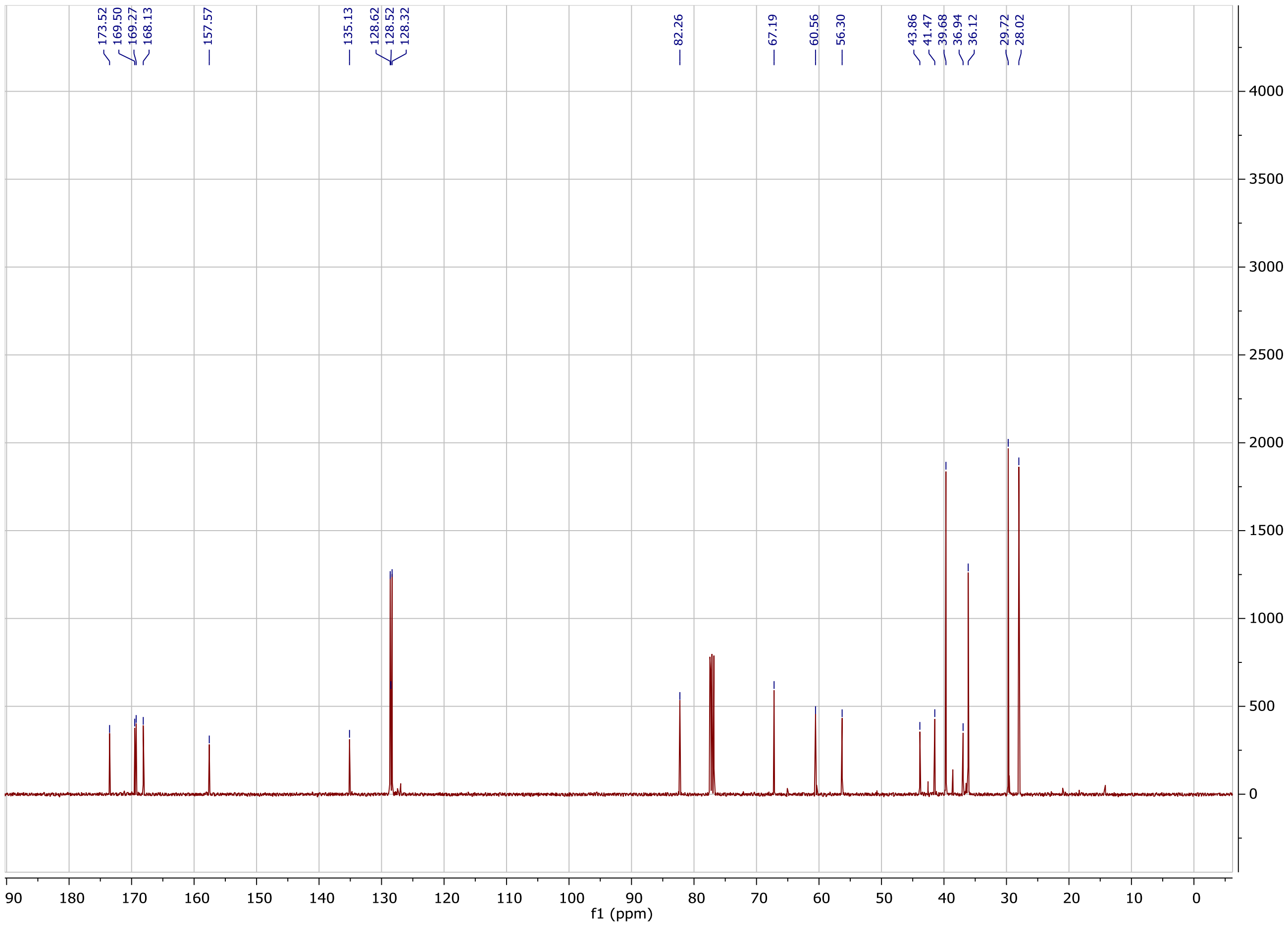

-L.G.S. - Laboratorio Grandi Strumenti - Display Report

Analysis Name	av 2915.d	Acquisition Date	06/28/21 08:18:25	Operator	Administrator
Sample Name		Method	Copy of $wp_lm.MS	Instrument	esquire3000plus
Comment	1 mg/mL dil 1:100 MeOH<br>Richiedente: Volonteriop				

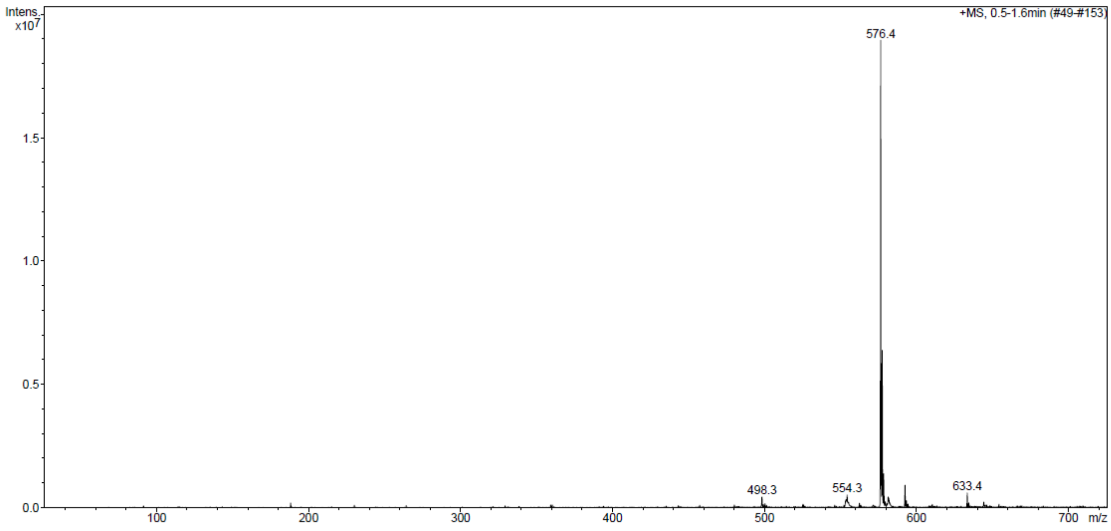

Bruker Daltonics DataAnalysis 3.1 printed: 06/28/21 08:20:31 Page 1 of 1

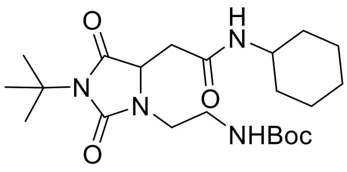

6e

^{1}H NMR (400 MHZ, $CDCl_3$)

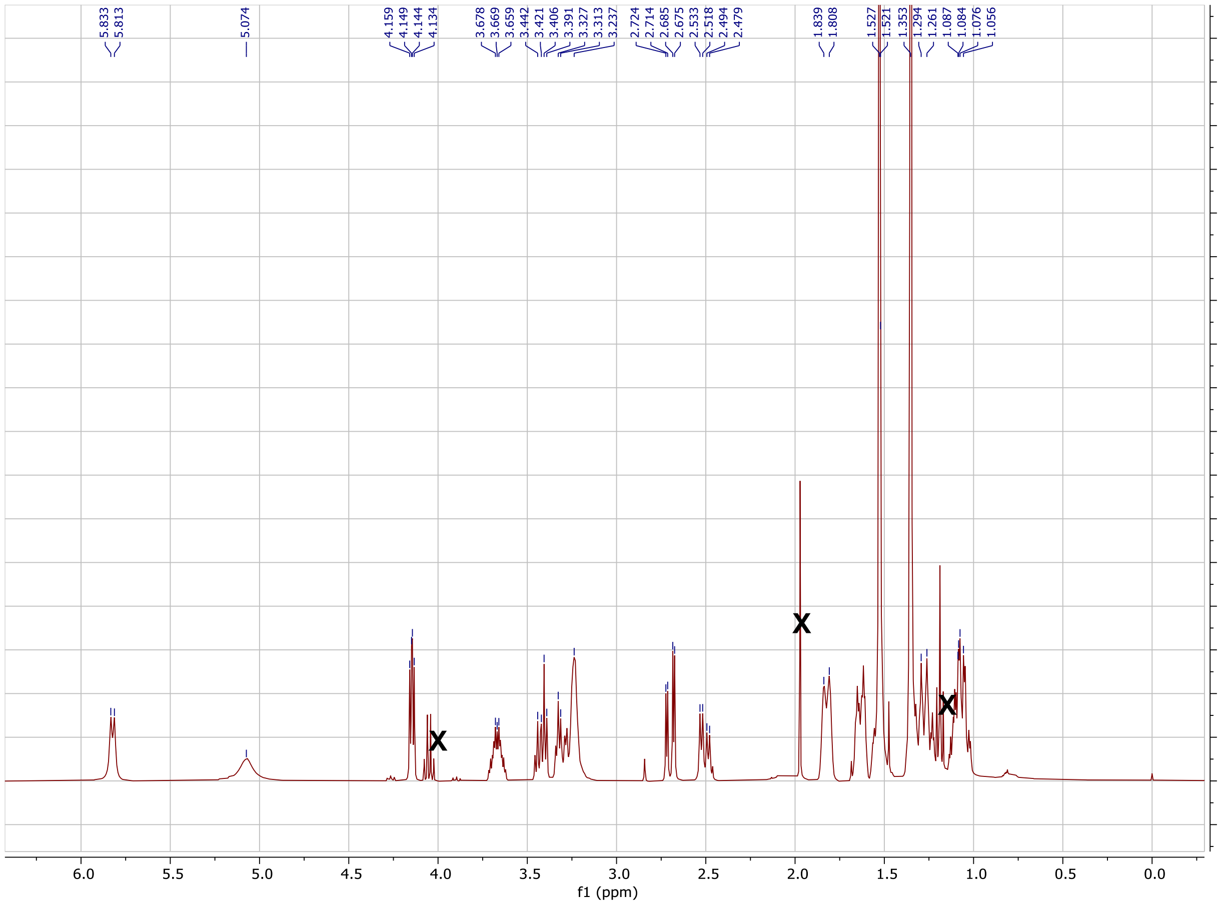

$^{13}C\{^{1}H\}$ NMR (101 MHz, $CDCl_3$)

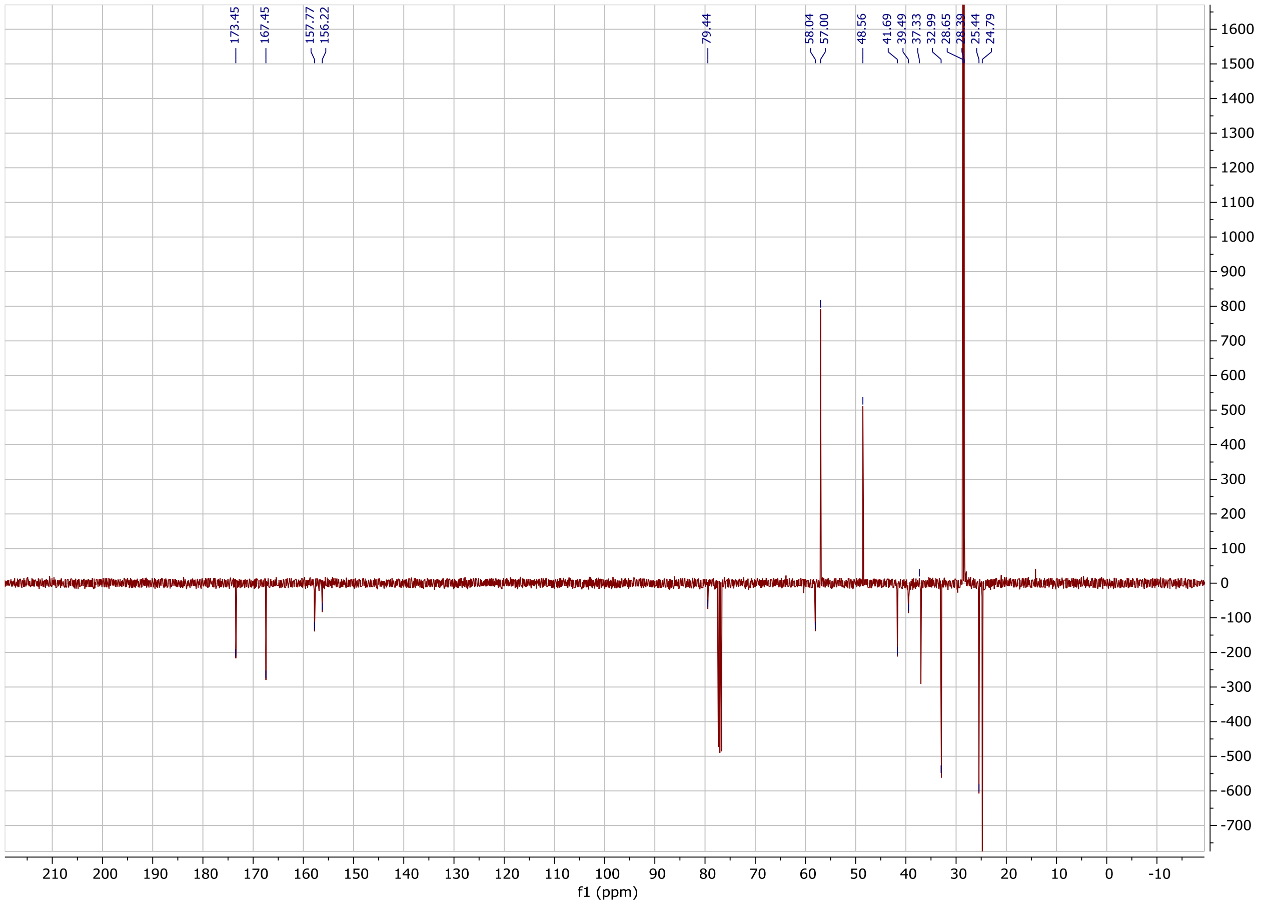

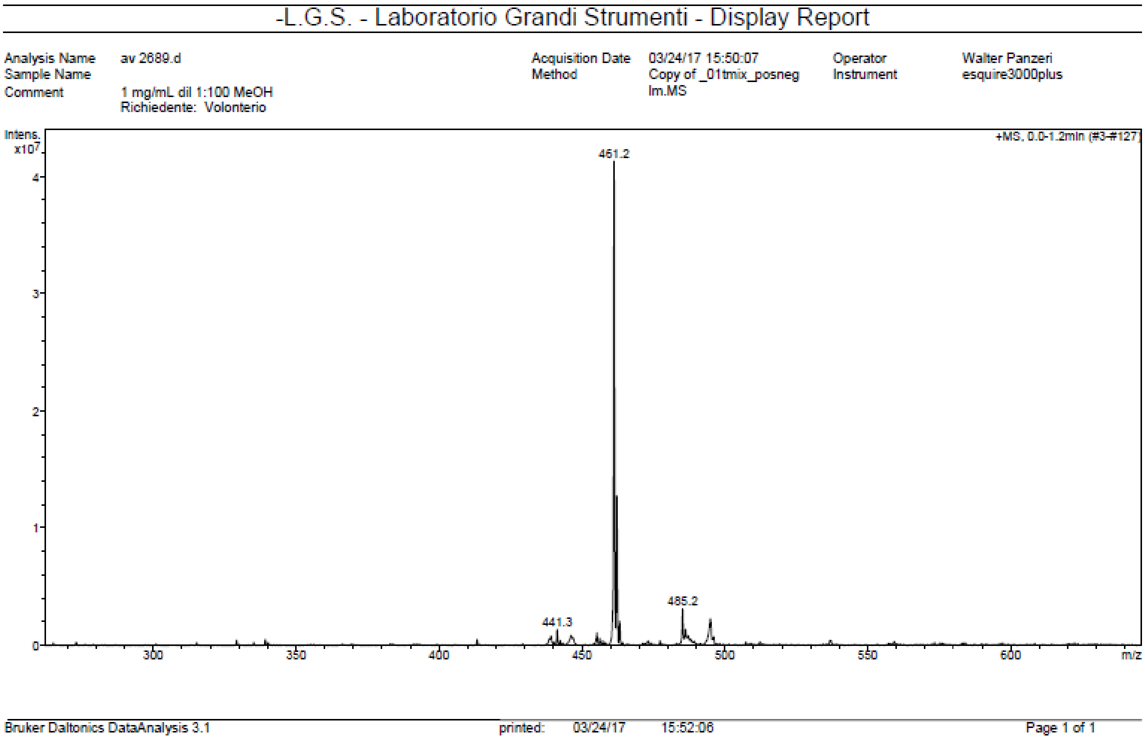

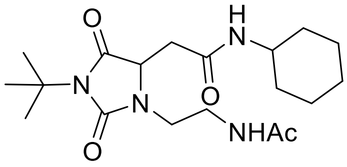

6f

^{1}H NMR (400 MHZ, $CDCl_3$)

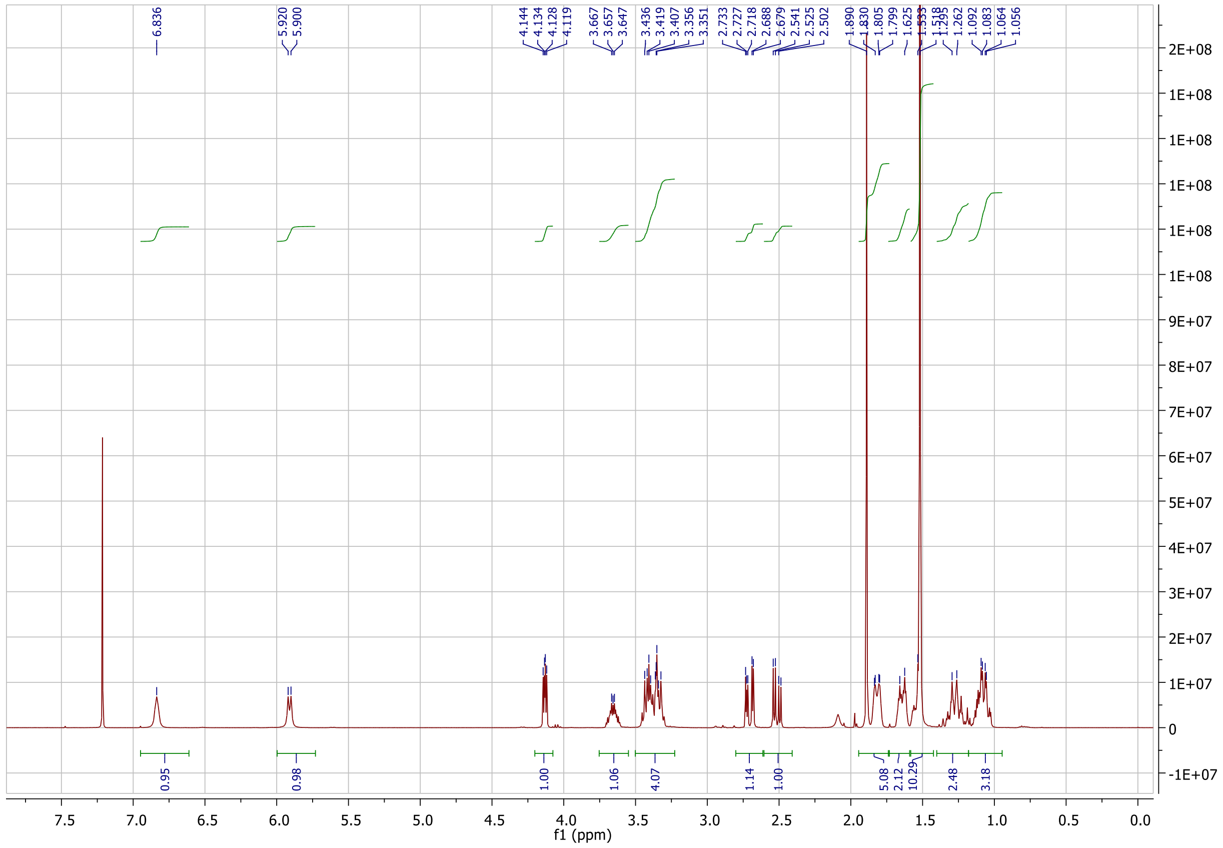

gCOSY NMR (400 MHZ, $CDCl_3$)

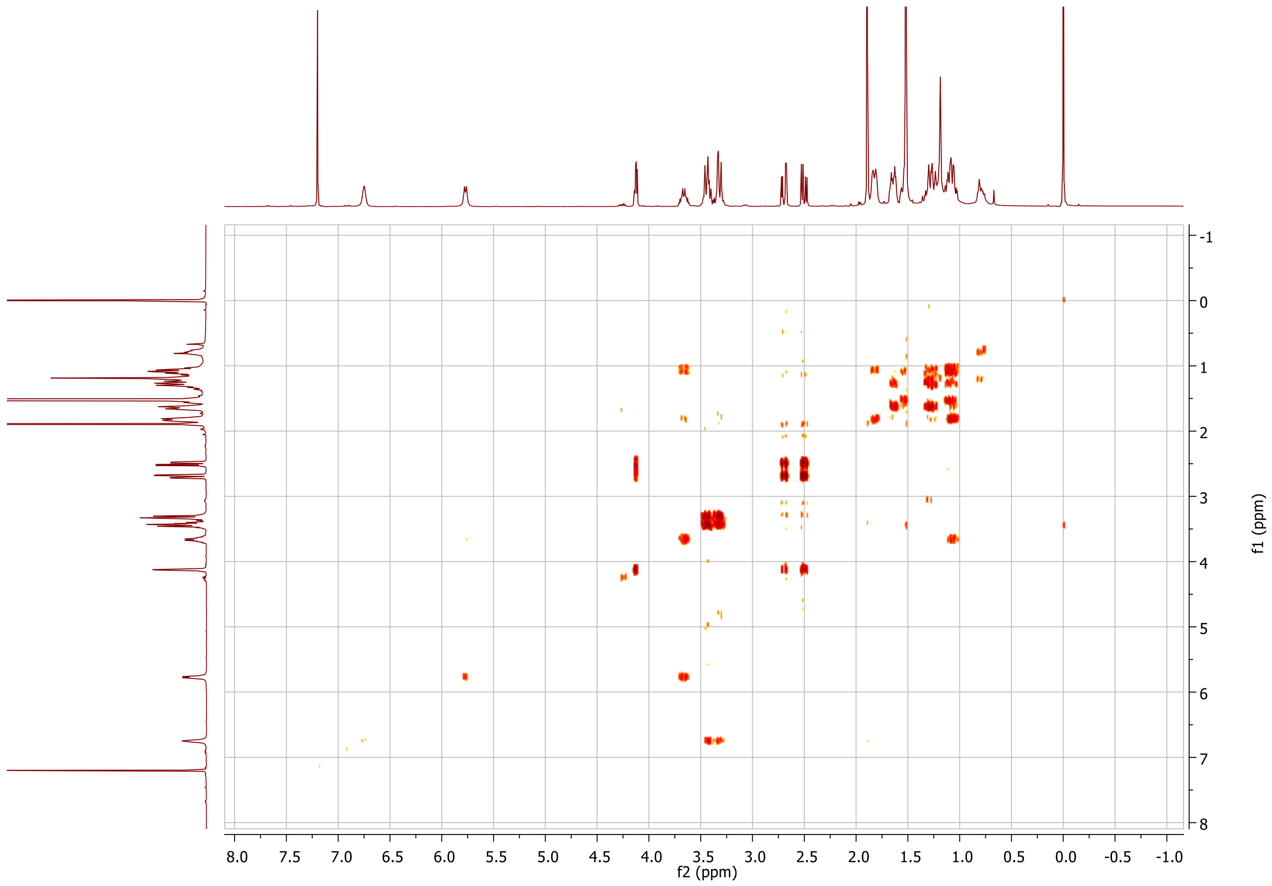

$^{13}C\{^1H\}$ NMR (101 MHz, $CDCl_3$)

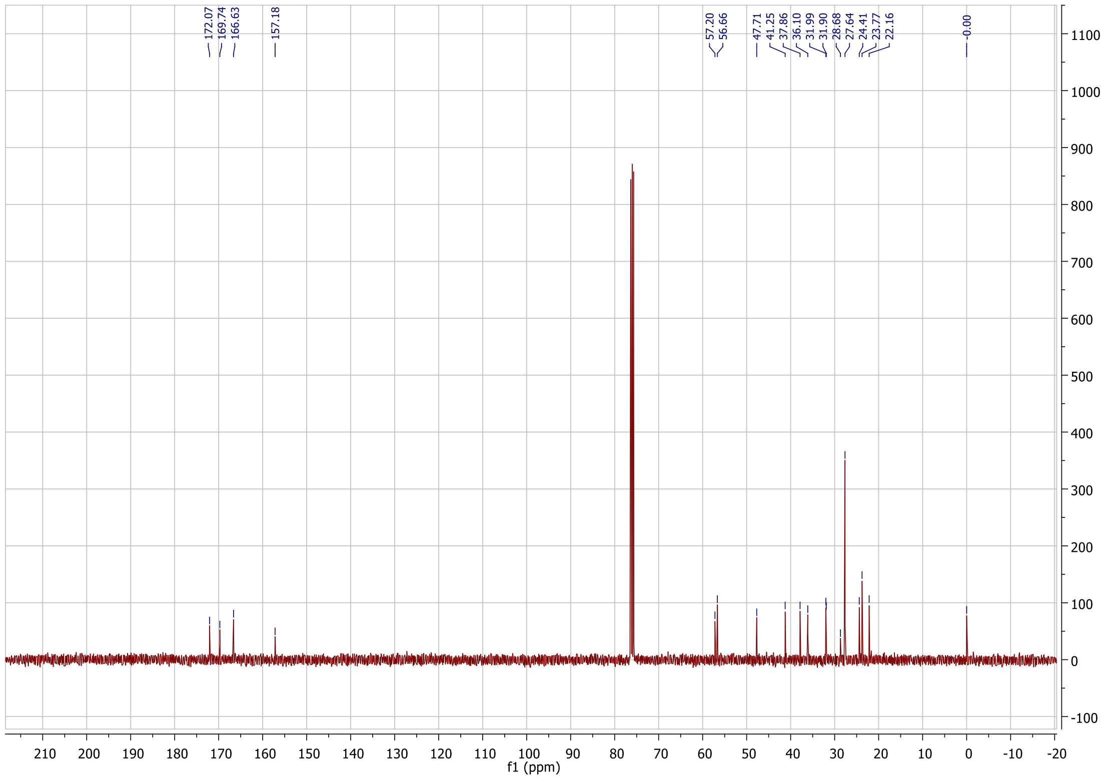

-L.G.S. - Laboratorio Grandi Strumenti - Display Report

Analysis Name	av gc20_c.d	Acquisition Date	01/26/18 14:43:10	Operator	Walter Panzeri
Sample Name		Method	Copy of _01tmix_posneg lm.MS	Instrument	esquire3000plus
Comment	1 mg/ml H2O Richiedente: Cristina				

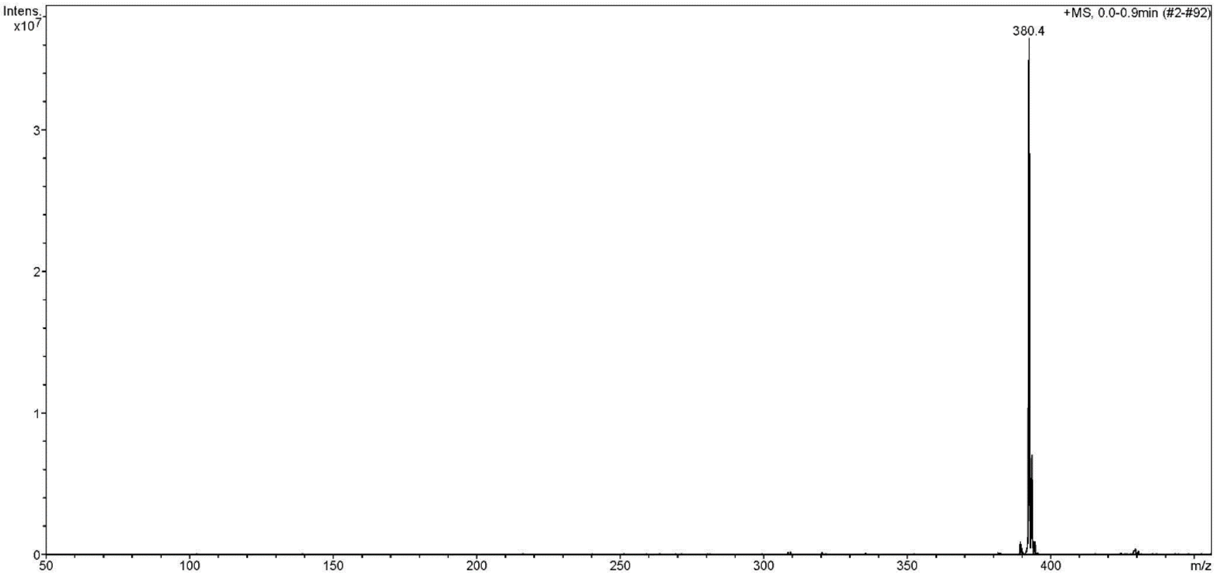

Bruker Daltonics DataAnalysis 3.1 printed: 01/26/18 14:44:33 Page 1 of 1

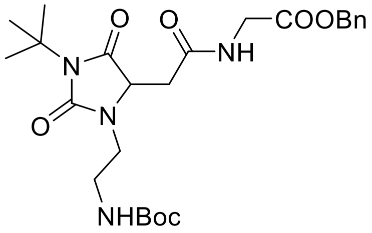

6g

^{1}H NMR (400 MHZ, $CDCl_3$)

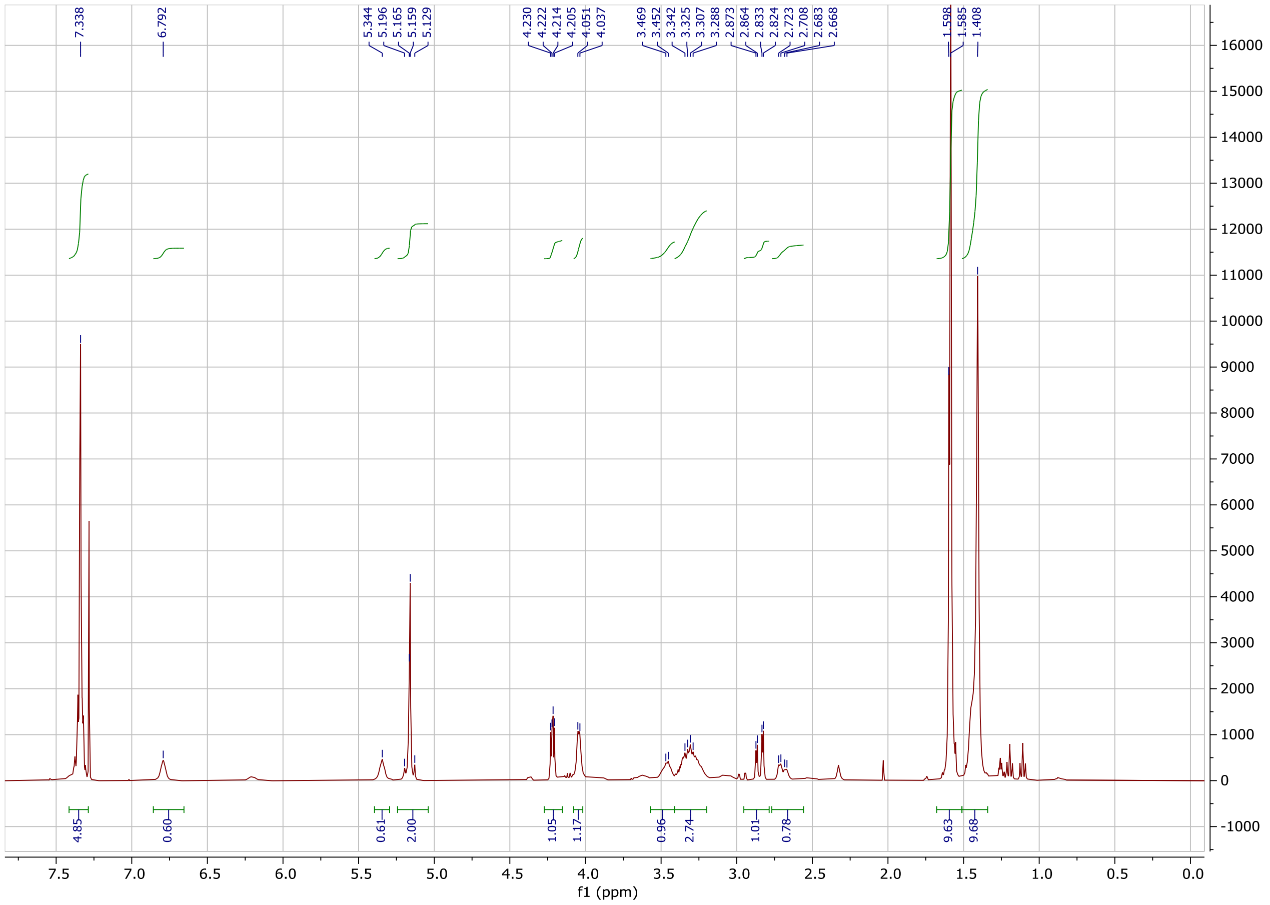

$^{13}C\{^1H\}$ NMR (101 MHz, $CDCl_3$)

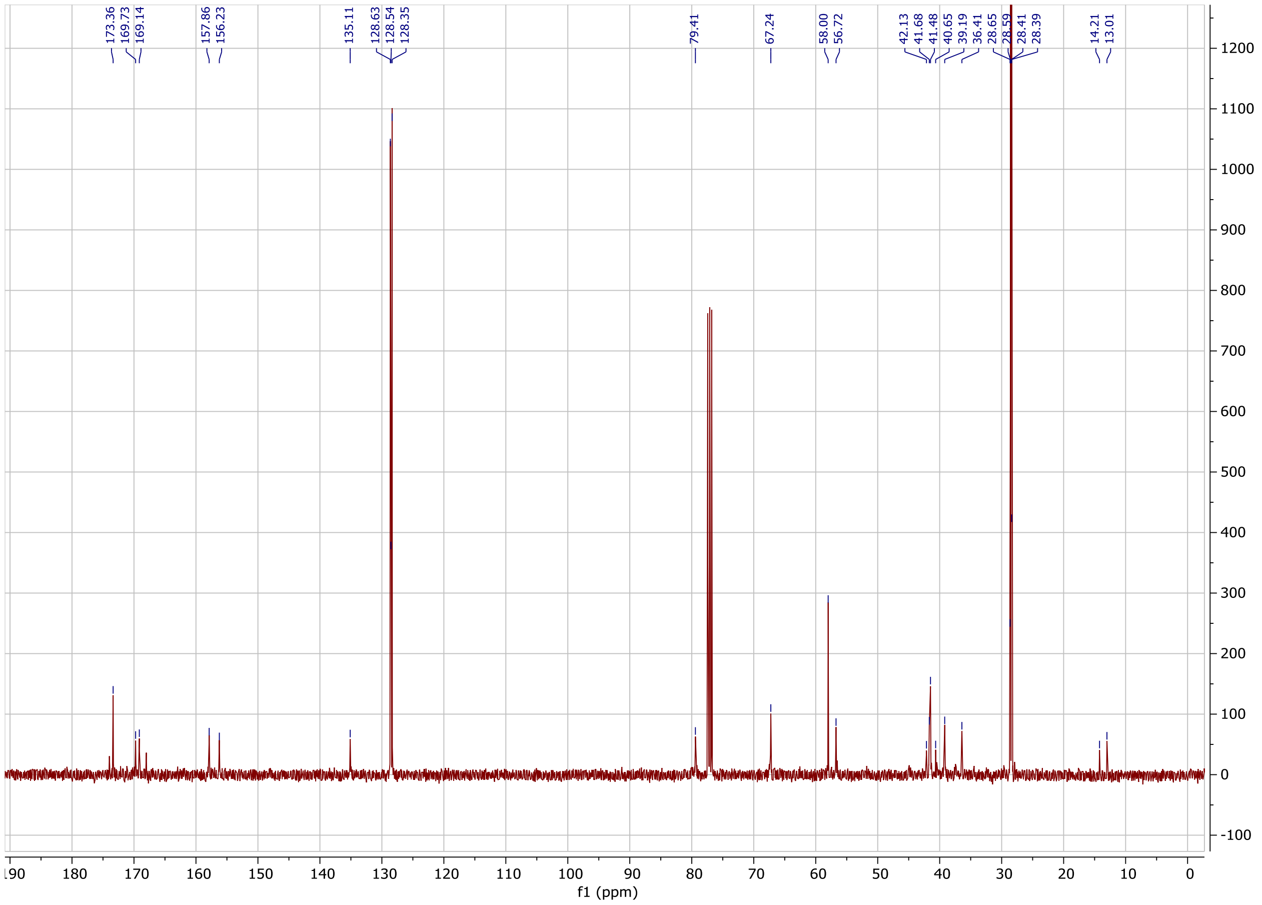

-L.G.S. - Laboratorio Grandi Strumenti - Display Report

Analysis Name	av 2911.d	Acquisition Date	06/04/21 09:25:49	Operator	Walter Panzeri
Sample Name		Method	Copy of $wp_lm.MS	Instrument	esquire3000plus
Comment	1 mg/mL dil 1:100 MeOH<br>Richiedente: Volonterio				

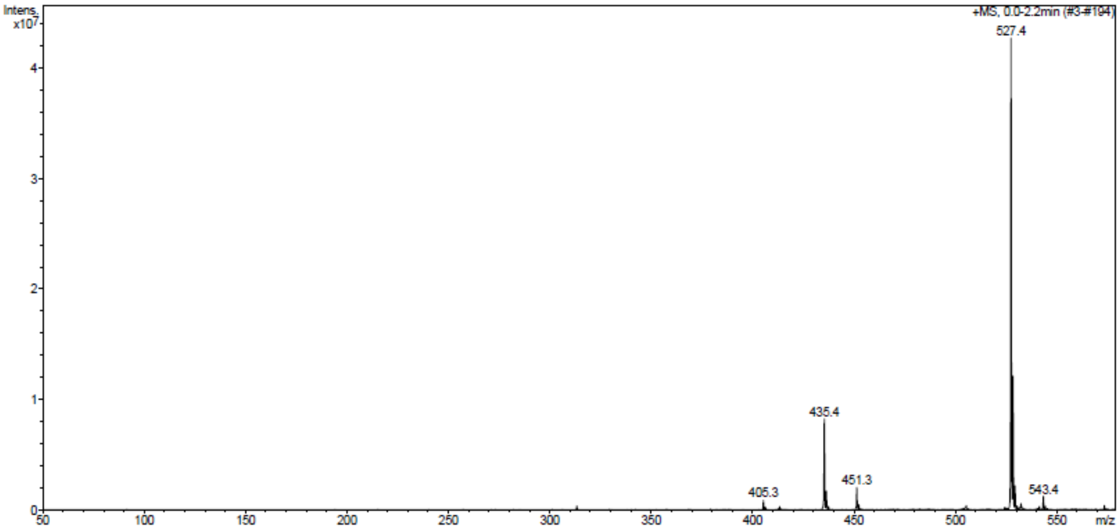

Bruker Daltonics DataAnalysis 3.1 printed: 06/04/21 09:28:49 Page 1 of 1

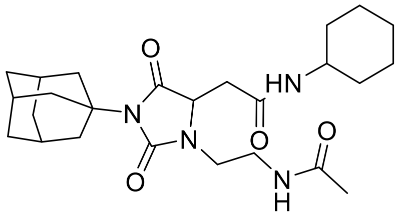

6h

^{1}H NMR (400 MHZ, $CDCl_3$)

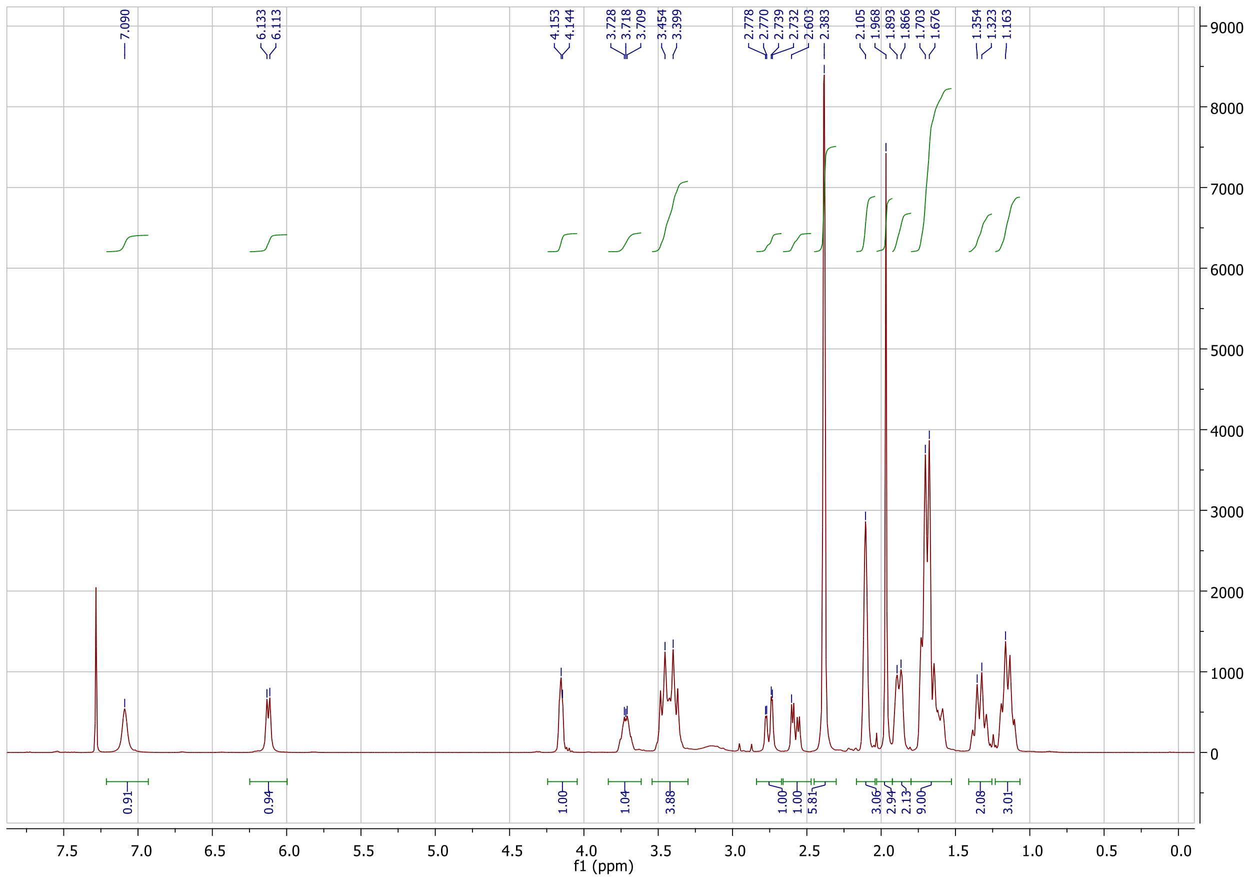

gCOSY NMR (400 MHZ, $CDCl_3$)

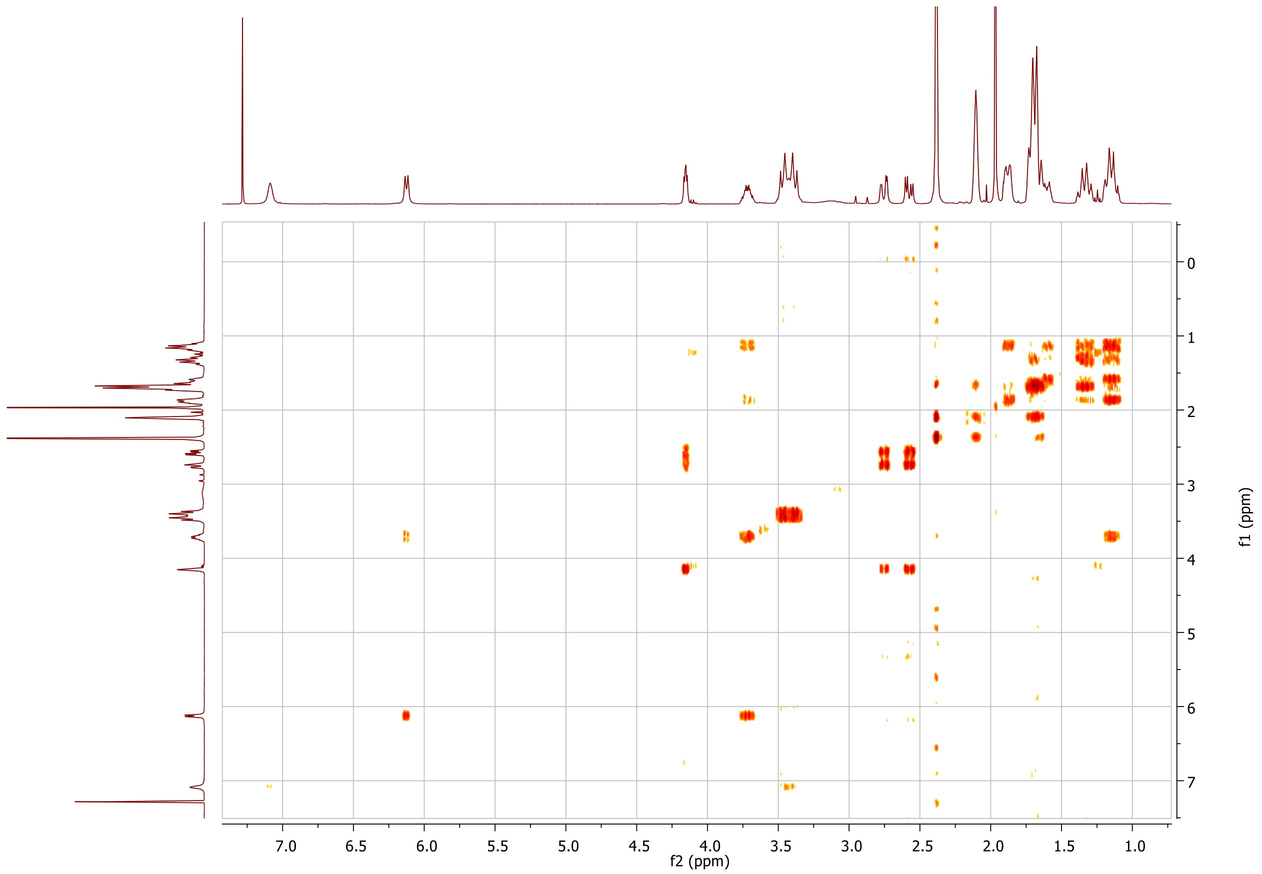

$^{13}C\{^1H\}$ NMR (101 MHz, $CDCl_3$)

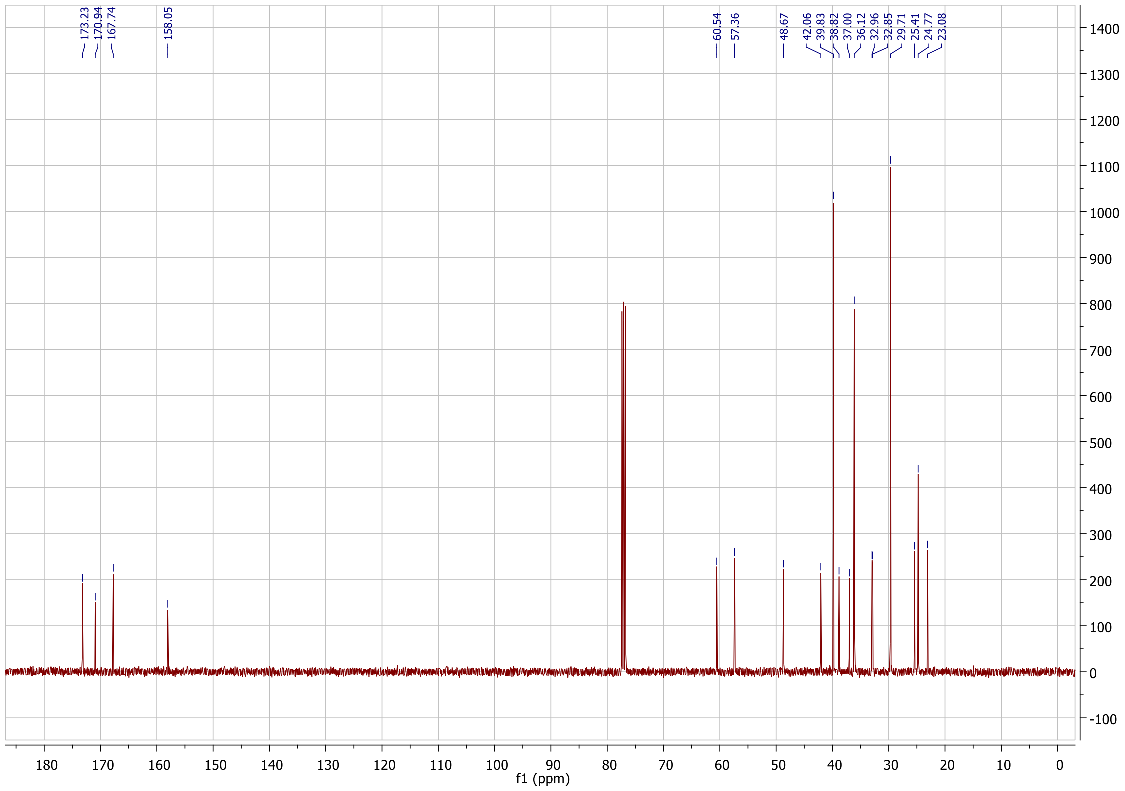

-L.G.S. - Laboratorio Grandi Strumenti - Display Report

Analysis Name	av gc118.d	Acquisition Date	03/26/19 13:57:44	Operator	Walter Panzeri
Sample Name		Method	Copy of _01tmix_posneg lm.MS	Instrument	esquire3000plus
Comment	1 mg/ml dil 1:100 MeOH<br>Richiedente: Cristina				

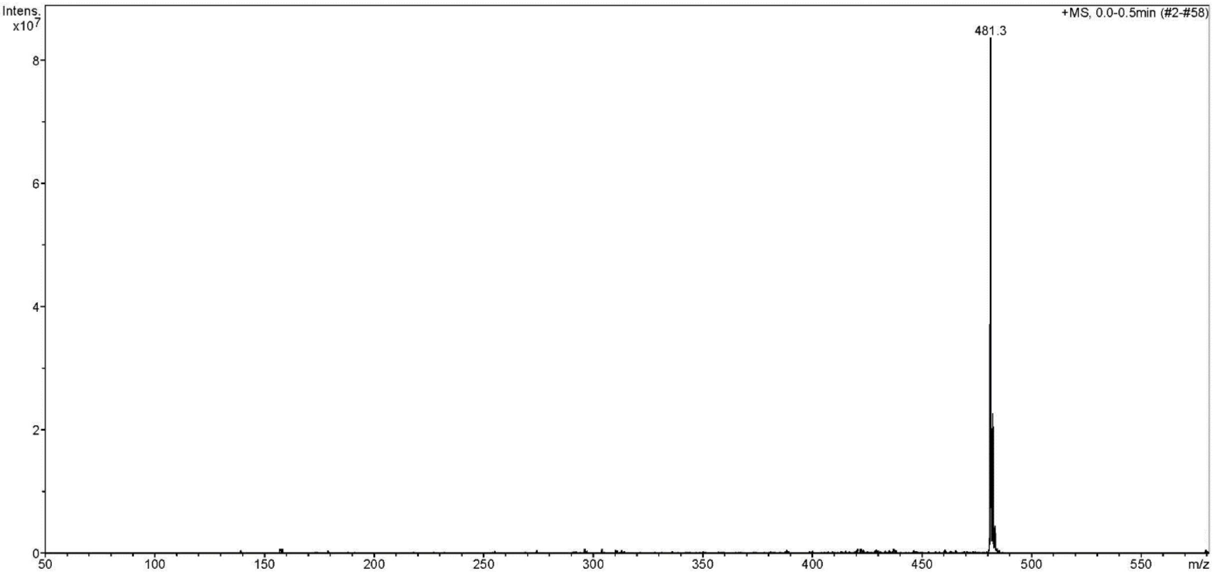

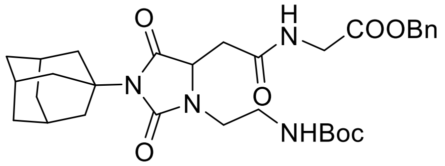

6i

^{1}H NMR (400 MHZ, $CDCl_3$)

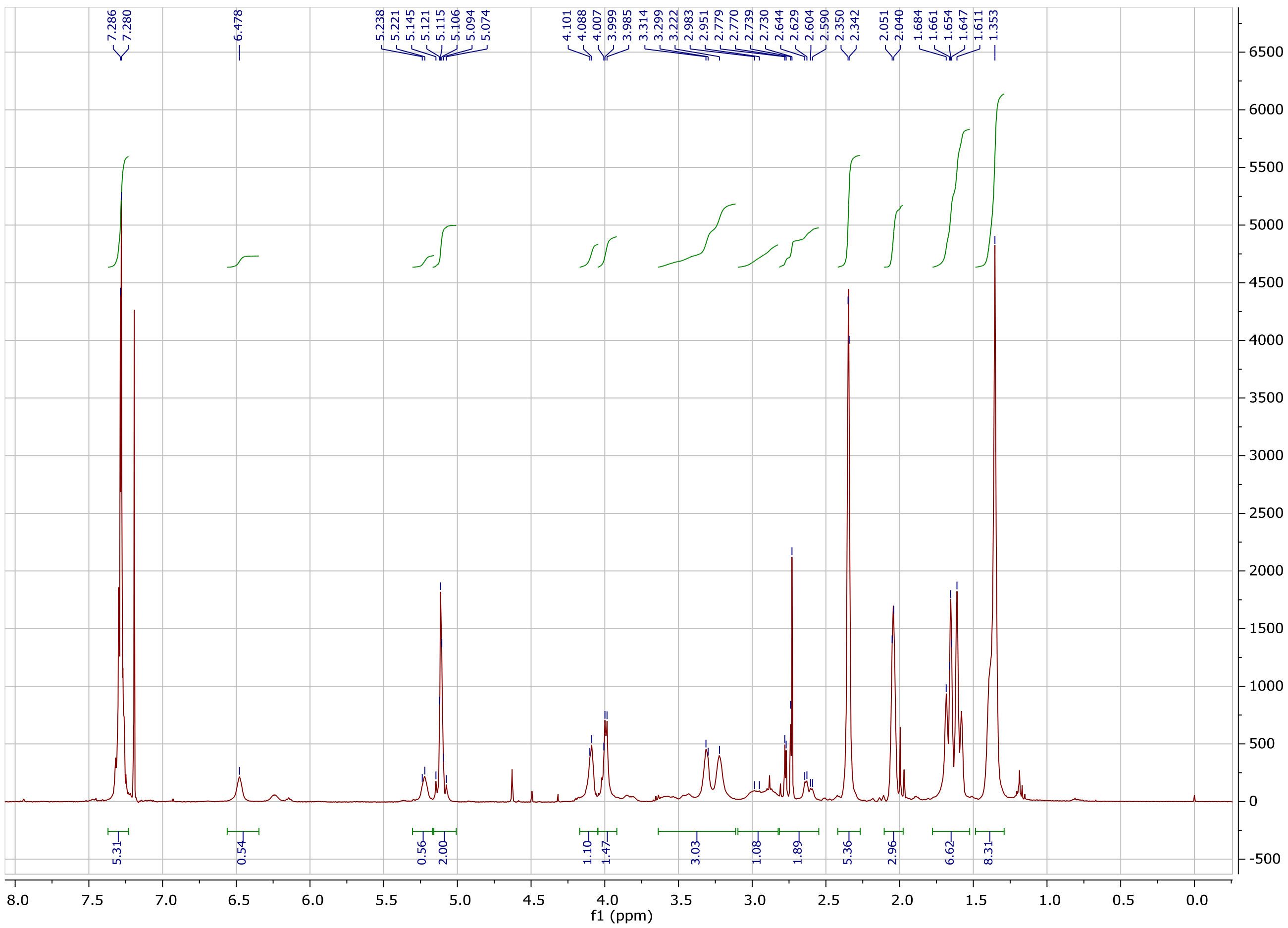

$^{13}C\{^{1}H\}$ NMR (101 MHz, $CDCl_3$)

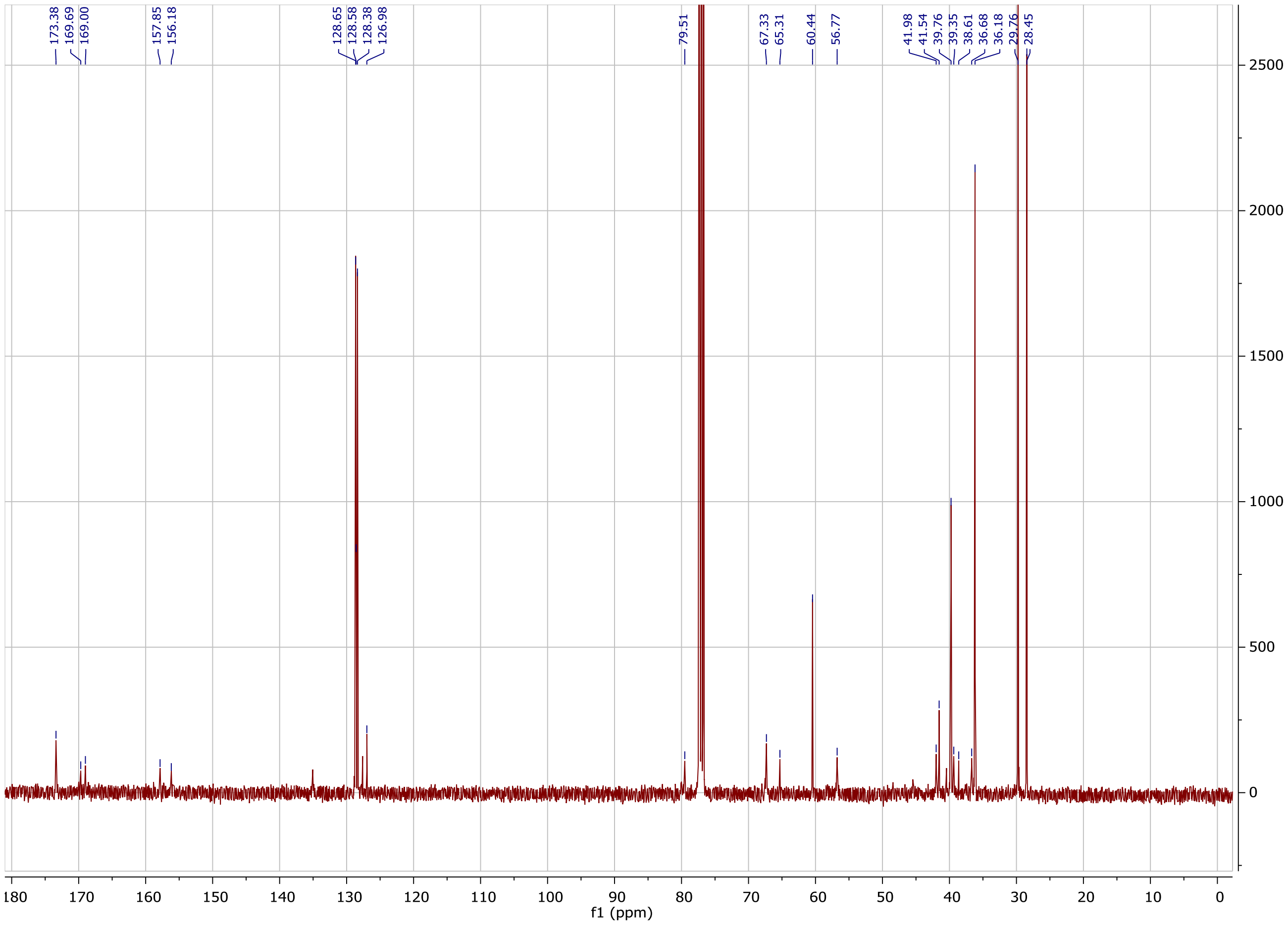

-L.G.S. - Laboratorio Grandi Strumenti - Display Report

Analysis Name	av 2925.d	Acquisition Date	07/16/21 08:31:05	Operator	Walter Panzeri
Sample Name		Method	Copy of _giov 1620 24713.MS	Instrument	esquire3000plus
Comment	1 mg/mL dil 1:100 MeOH Richiedente: Volonterio				

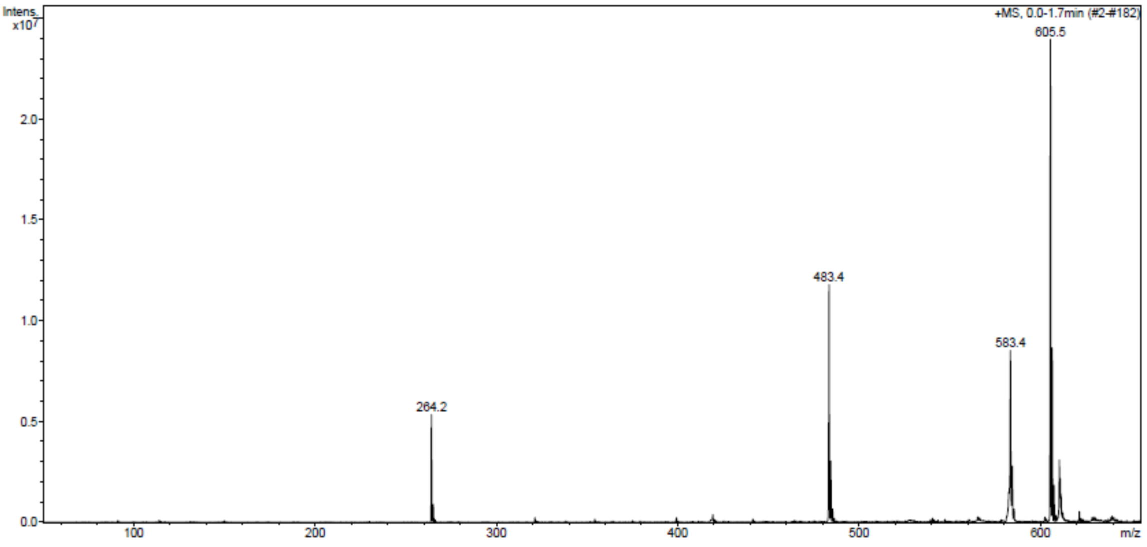

Bruker Daltonics DataAnalysis 3.1 printed: 07/16/21 08:33:40 Page 1 of 1

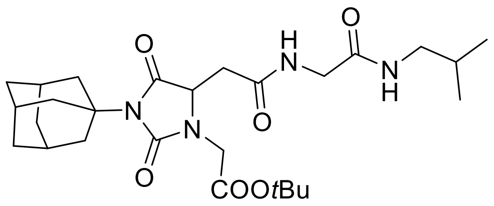

7

^{1}H NMR (400 MHZ, $CDCl_3$)

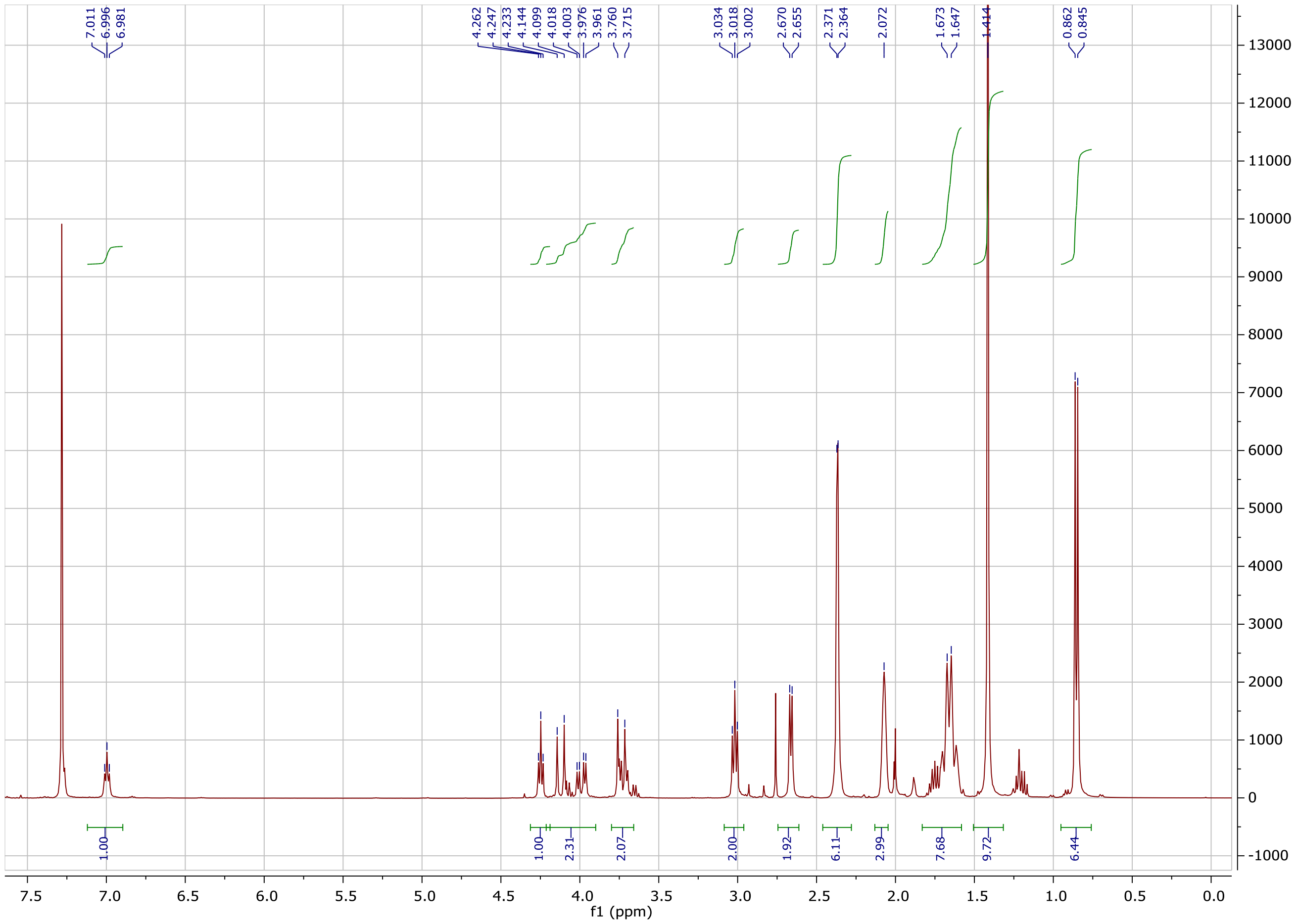

$^{13}C\{^{1}H\}$ NMR (101 MHz, $CDCl_3$)

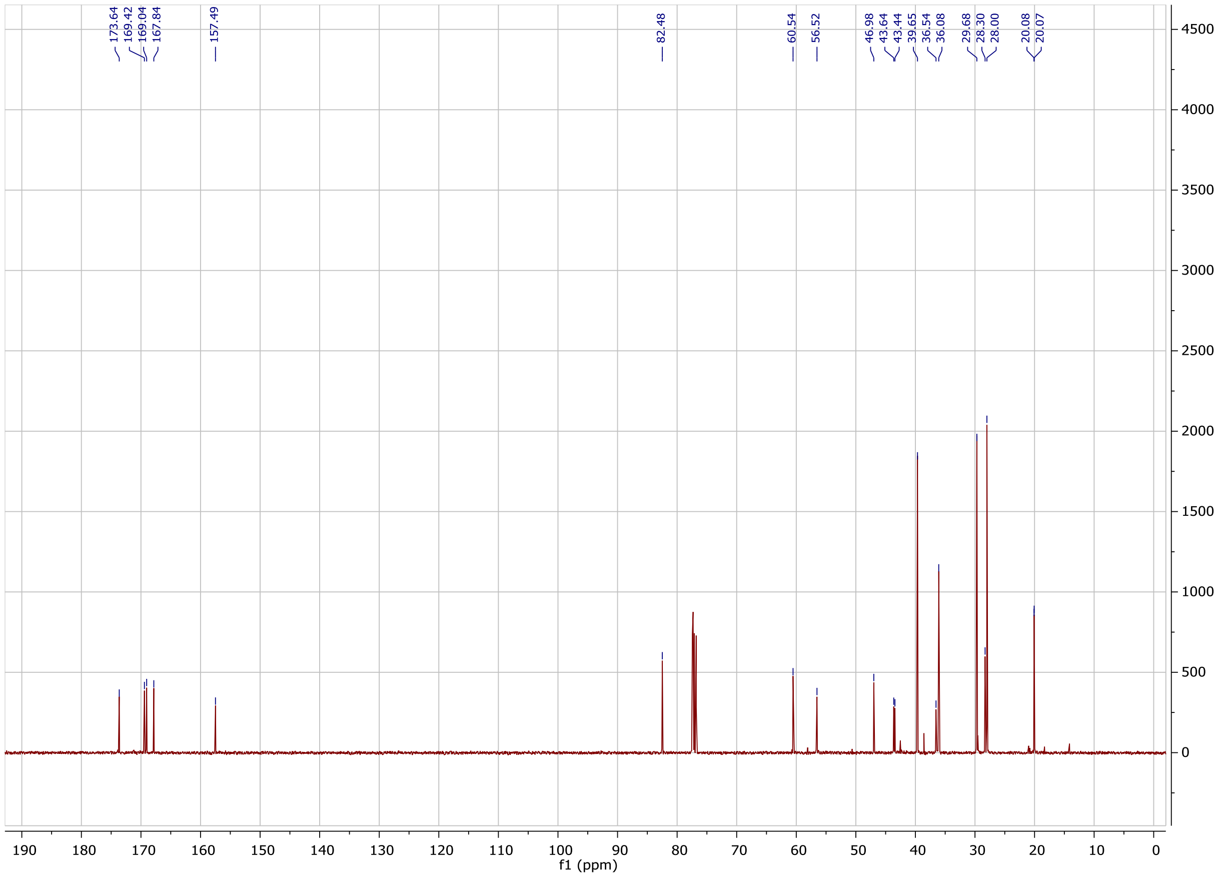

-L.G.S. - Laboratorio Grandi Strumenti - Display Report

Analysis Name	av 2916.d	Acquisition Date	06/30/21 09:11:35	Operator	Walter Panzeri
Sample Name		Method	Copy of $wp_lm.MS	Instrument	esquire3000plus
Comment	1 mg/mL dil 1:100 MeOH<br>Richiedente: Volonterio				

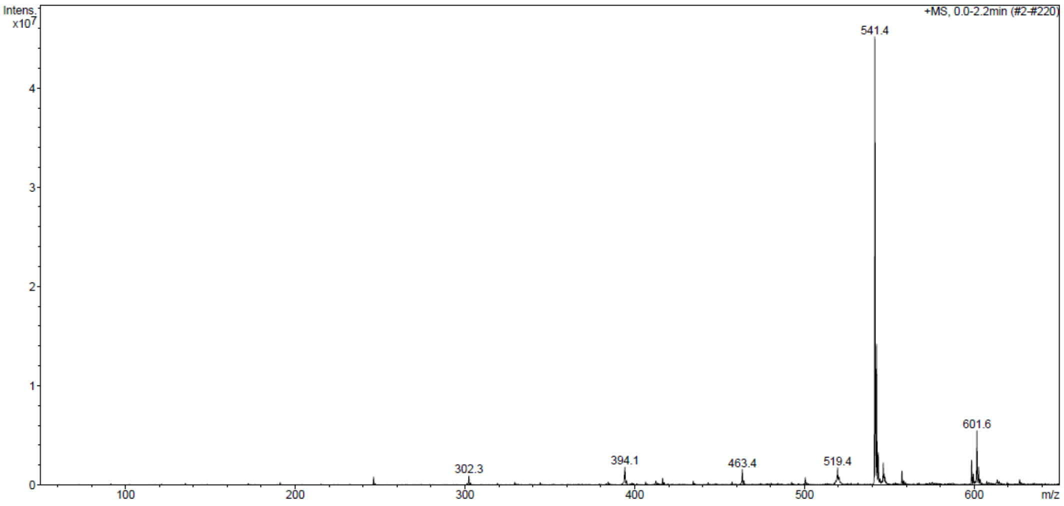

Bruker Daltonics DataAnalysis 3.1 printed: 06/30/21 09:22:05 Page 1 of 1

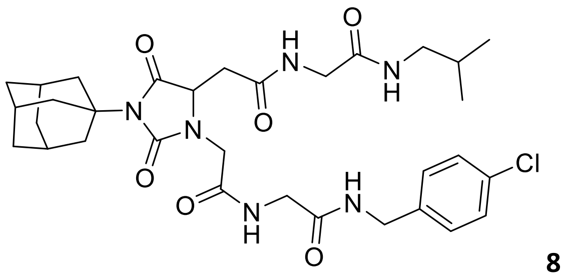

8

^{1}H NMR (400 MHZ, $CDCl_3$)

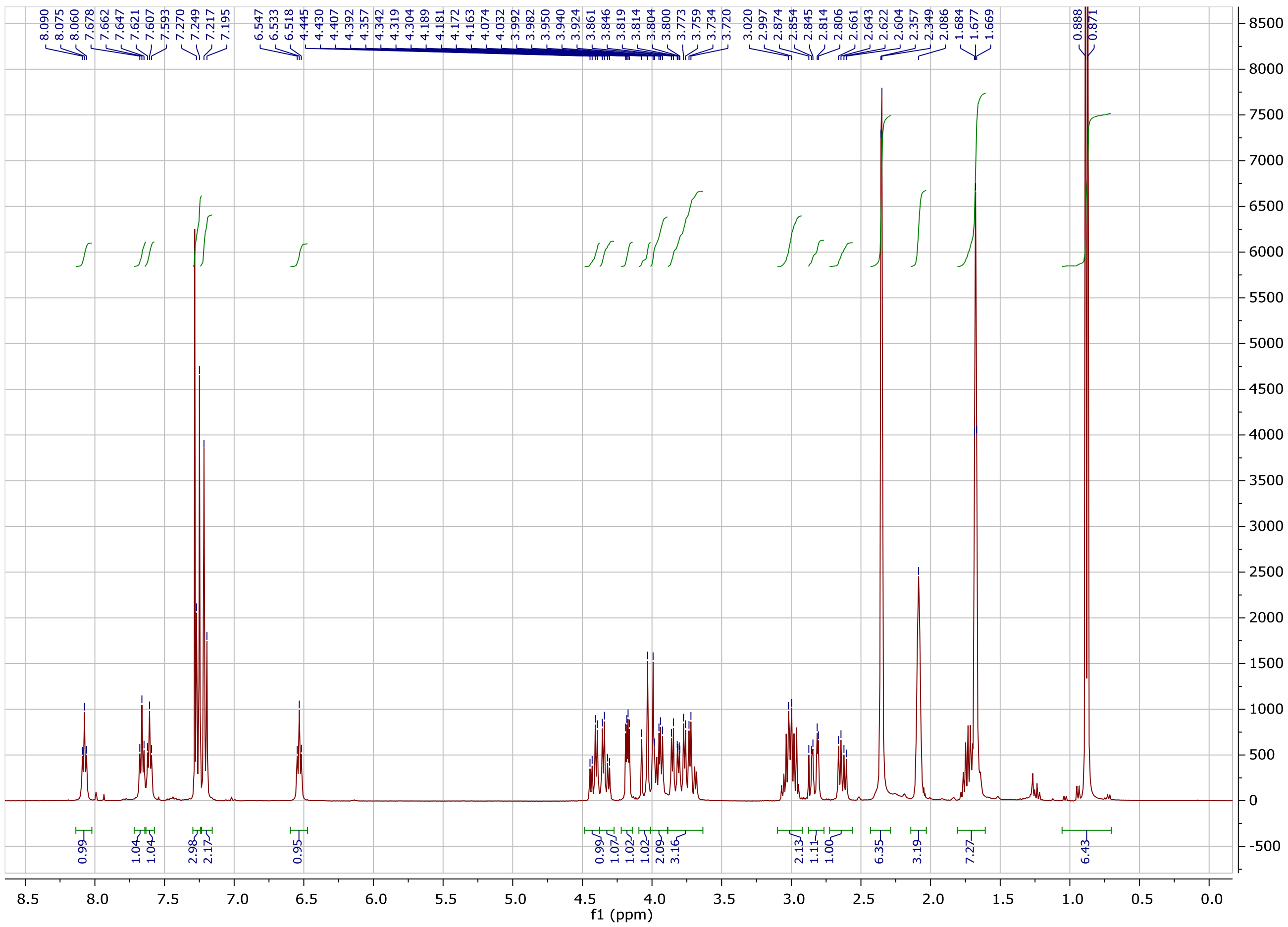

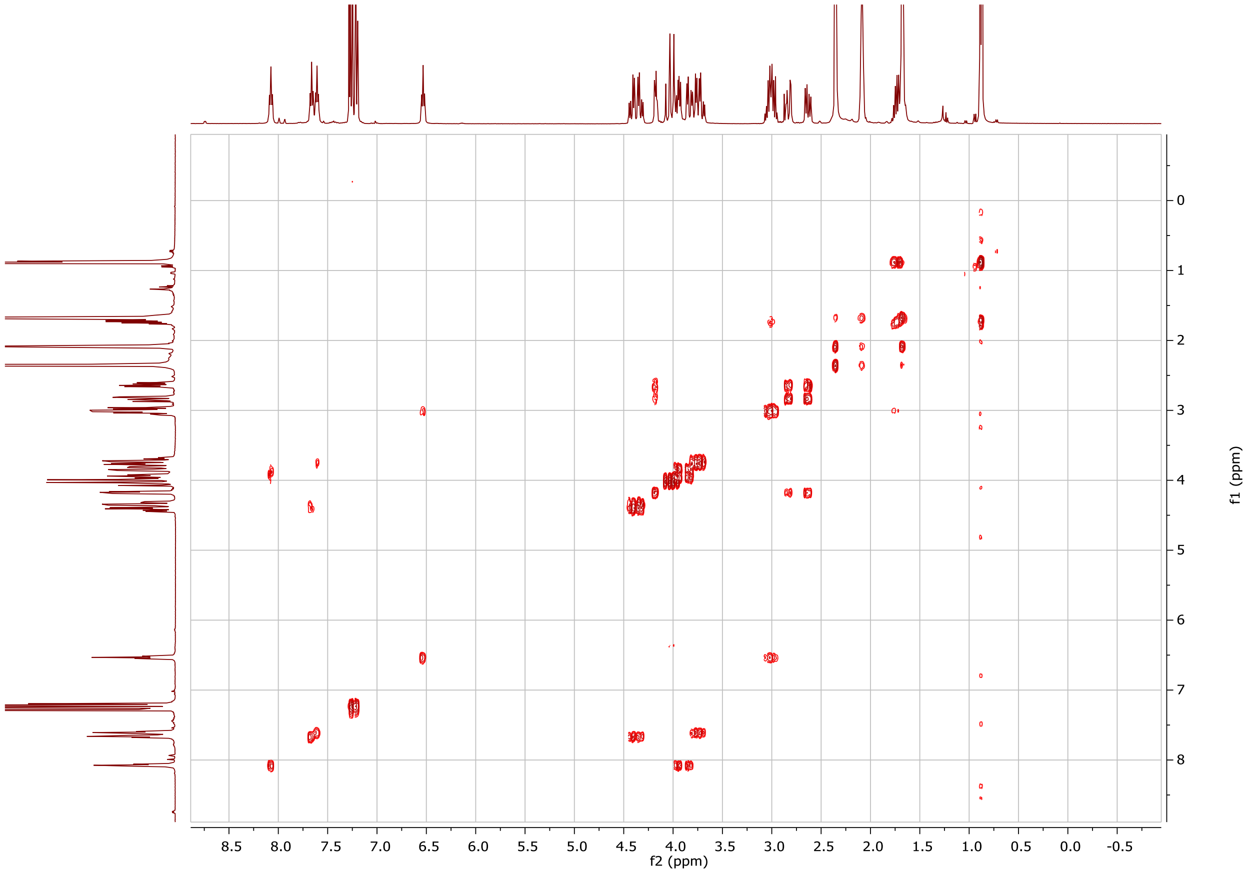

$^{13}C\{^1H\}$ NMR (101 MHz, $CDCl_3$)

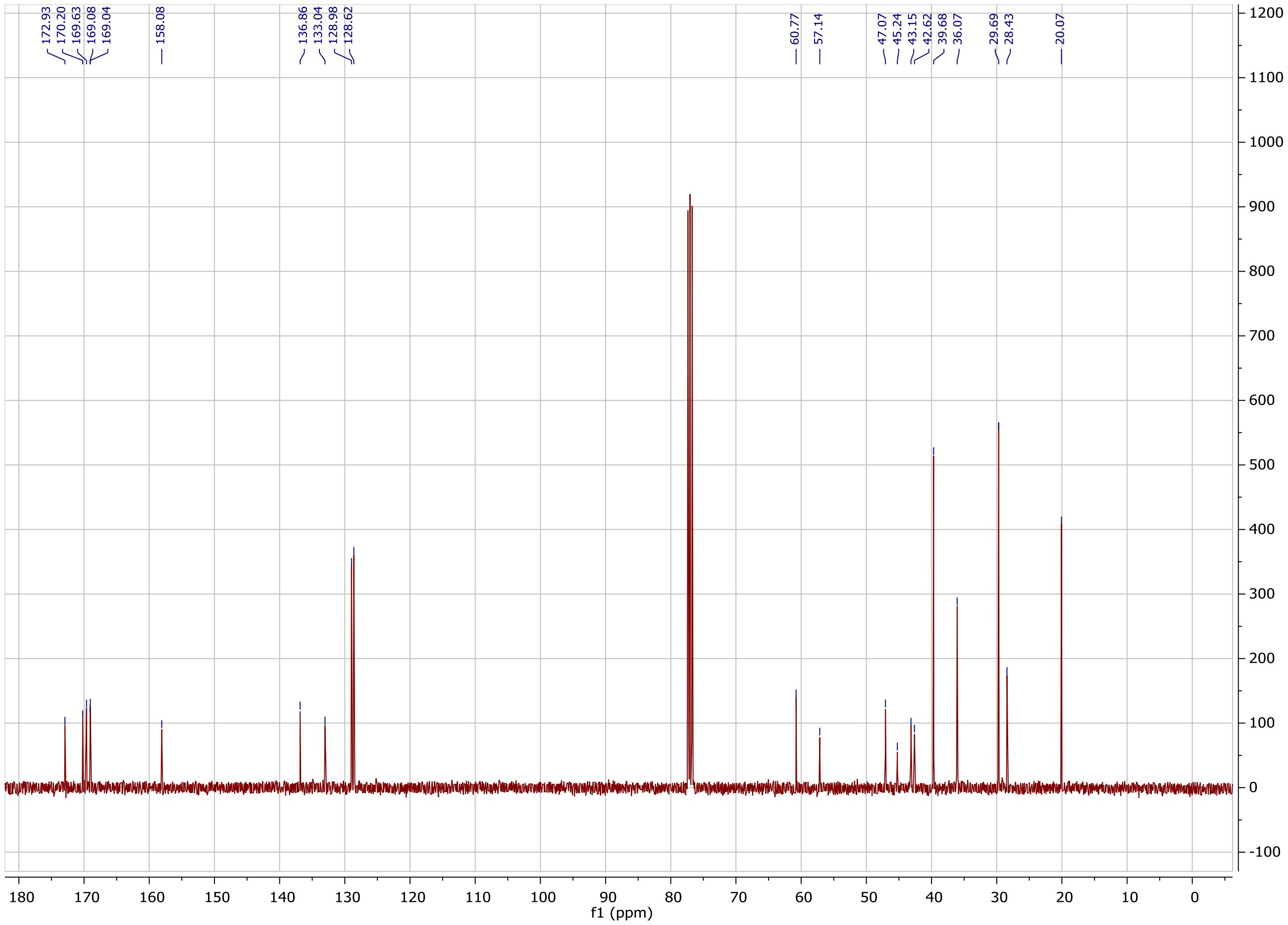

-L.G.S. - Laboratorio Grandi Strumenti - Display Report

Analysis Name	av 2920.d	Acquisition Date	07/02/21 12:03:47	Operator	Walter Panzeri
Sample Name		Method	Copy of $wp_lm.MS	Instrument	esquire3000plus
Comment	1 mg/mL dil 1:100 MeOH<br>Richiedente: Volonterio				

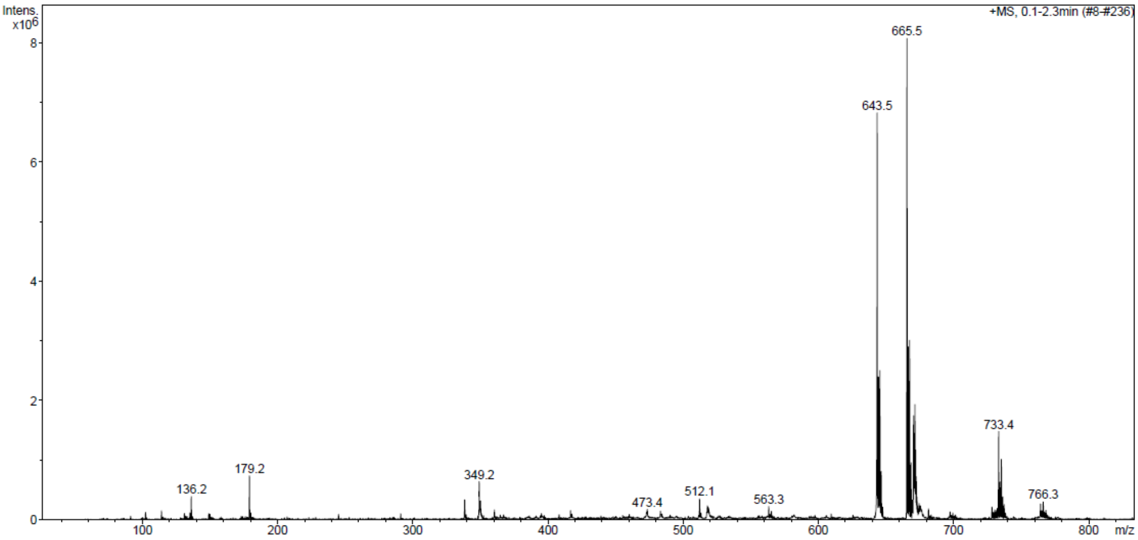

Bruker Daltonics DataAnalysis 3.1 printed: 07/02/21 12:12:10 Page 1 of 1

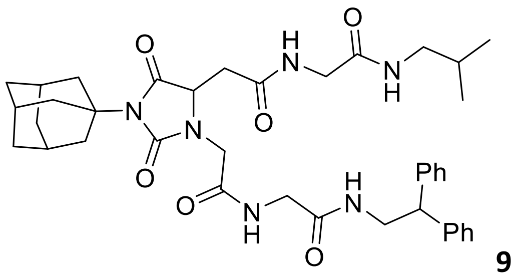

9

^{1}H NMR (400 MHZ, dmso-d$_6$)

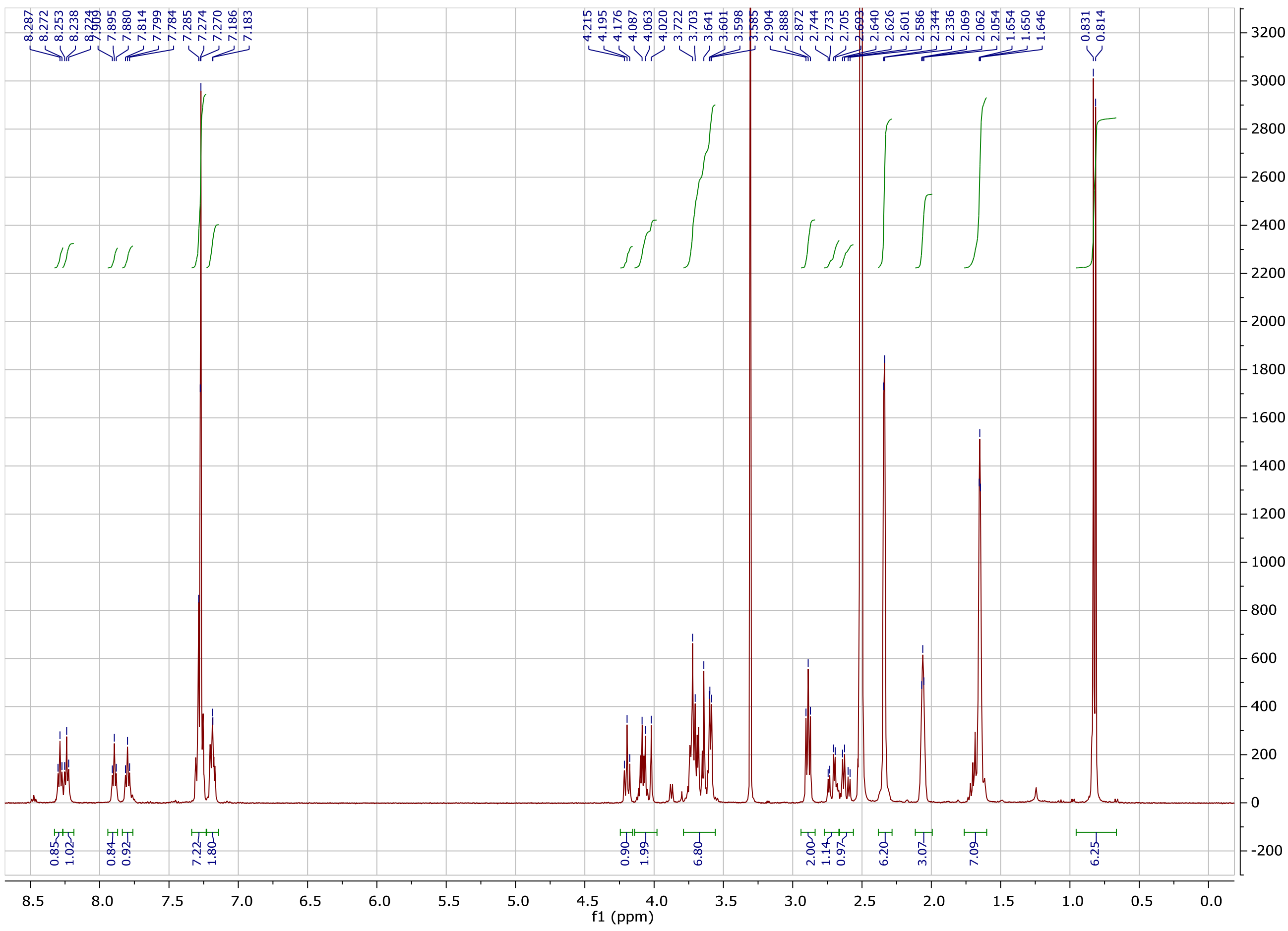

gCOSY NMR (400 MHZ, dmso-d$_6$)

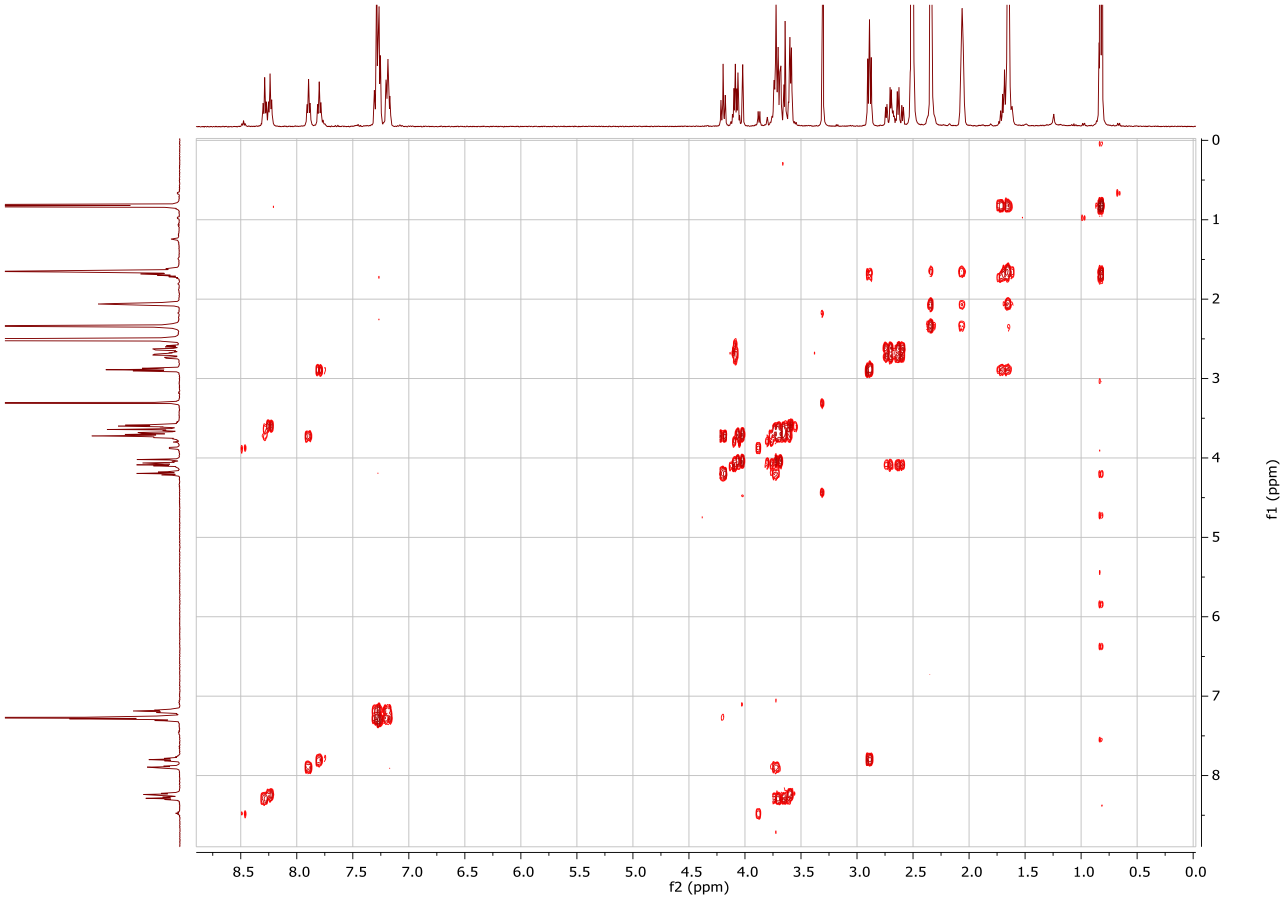

$^{13}C\{^1H\}$ NMR (101 MHz, dmso-d$_6$)

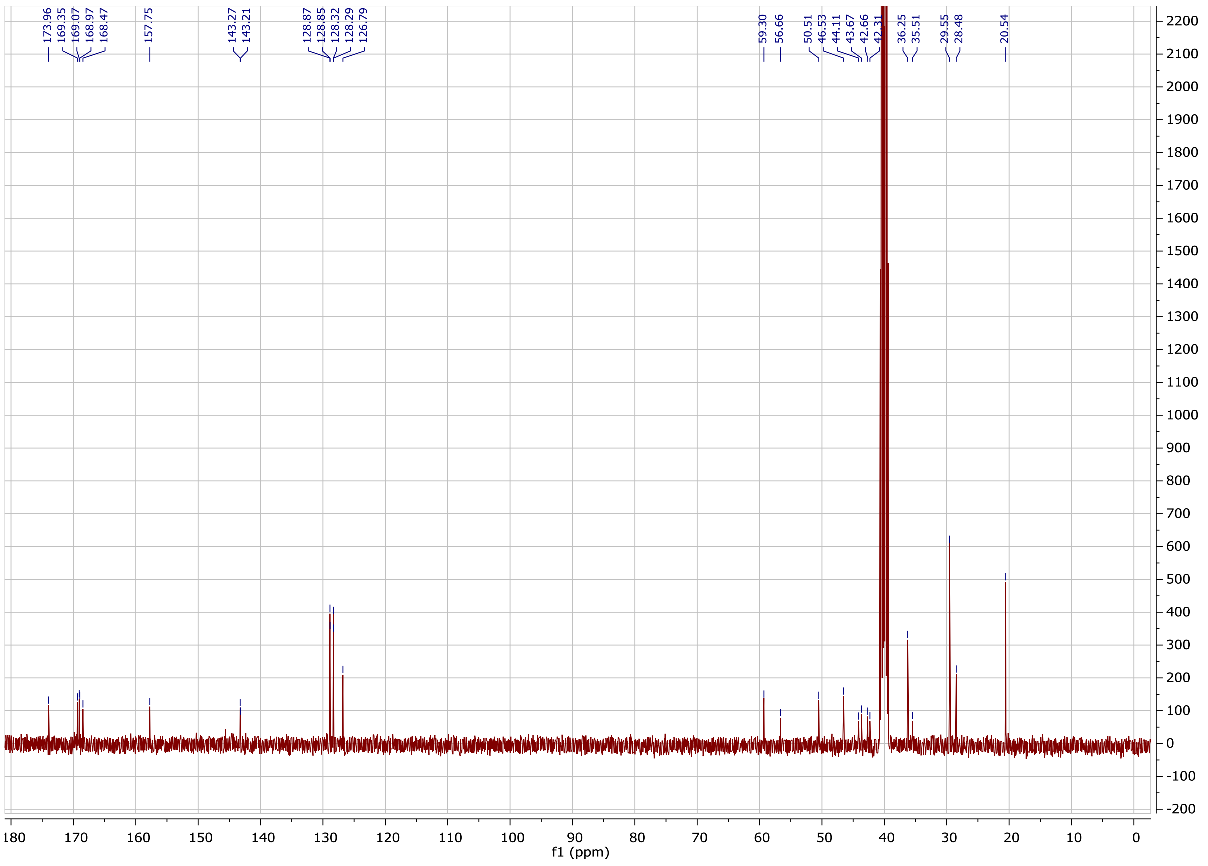

-L.G.S. - Laboratorio Grandi Strumenti - Display Report

Analysis Name	av gc113.d	Acquisition Date	03/06/19 14:49:10	Operator	Walter Panzeri
Sample Name		Method	Copy of _01tmix_posneg lm.MS	Instrument	esquire3000plus
Comment	1 mg/ml dil 1:100 MeOH<br>Richiedente: Cristina				

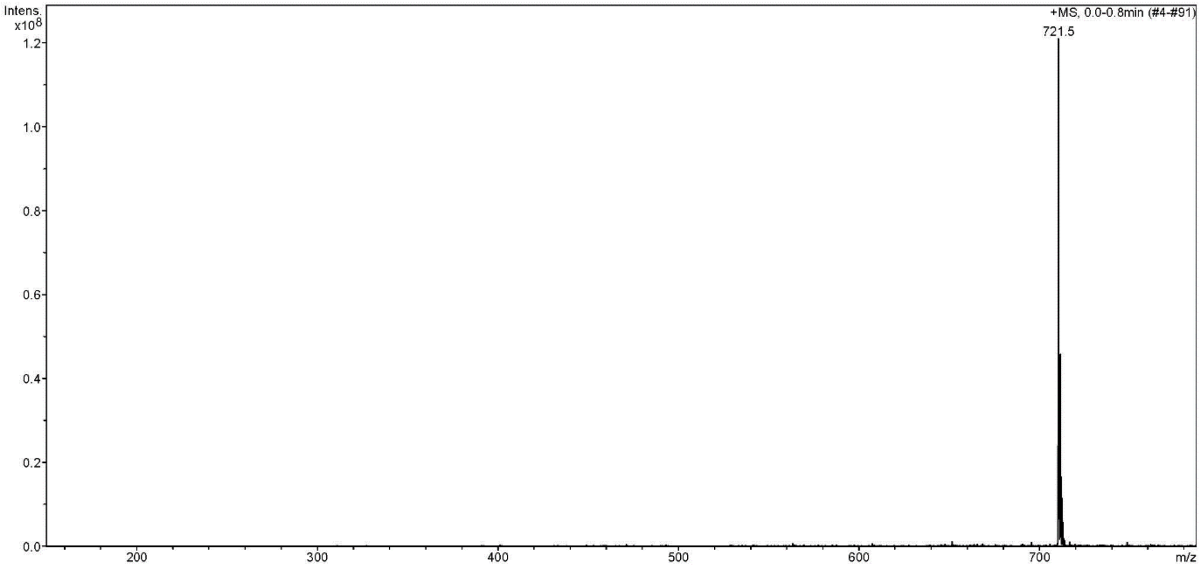

Bruker Daltonics DataAnalysis 3.1 printed: 03/06/19 15:05:46 Page 1 of 1

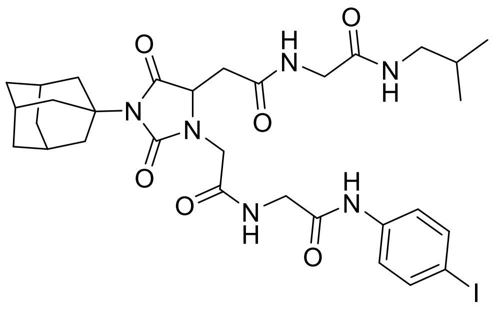

10

^{1}H NMR (400 MHZ, CD_3OD)

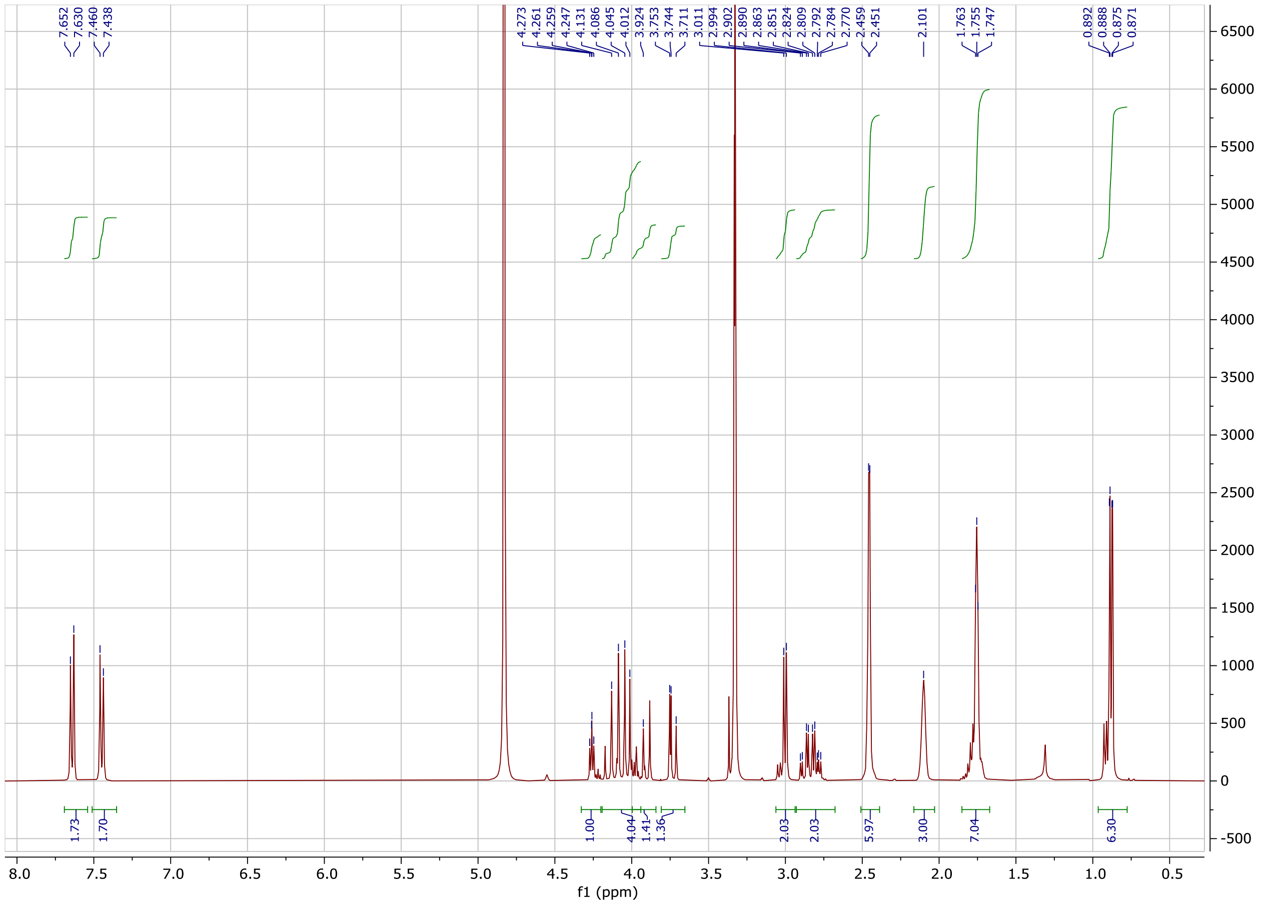

$^{13}C\{^1H\}$ NMR (101 MHz, $CDCl_3$)

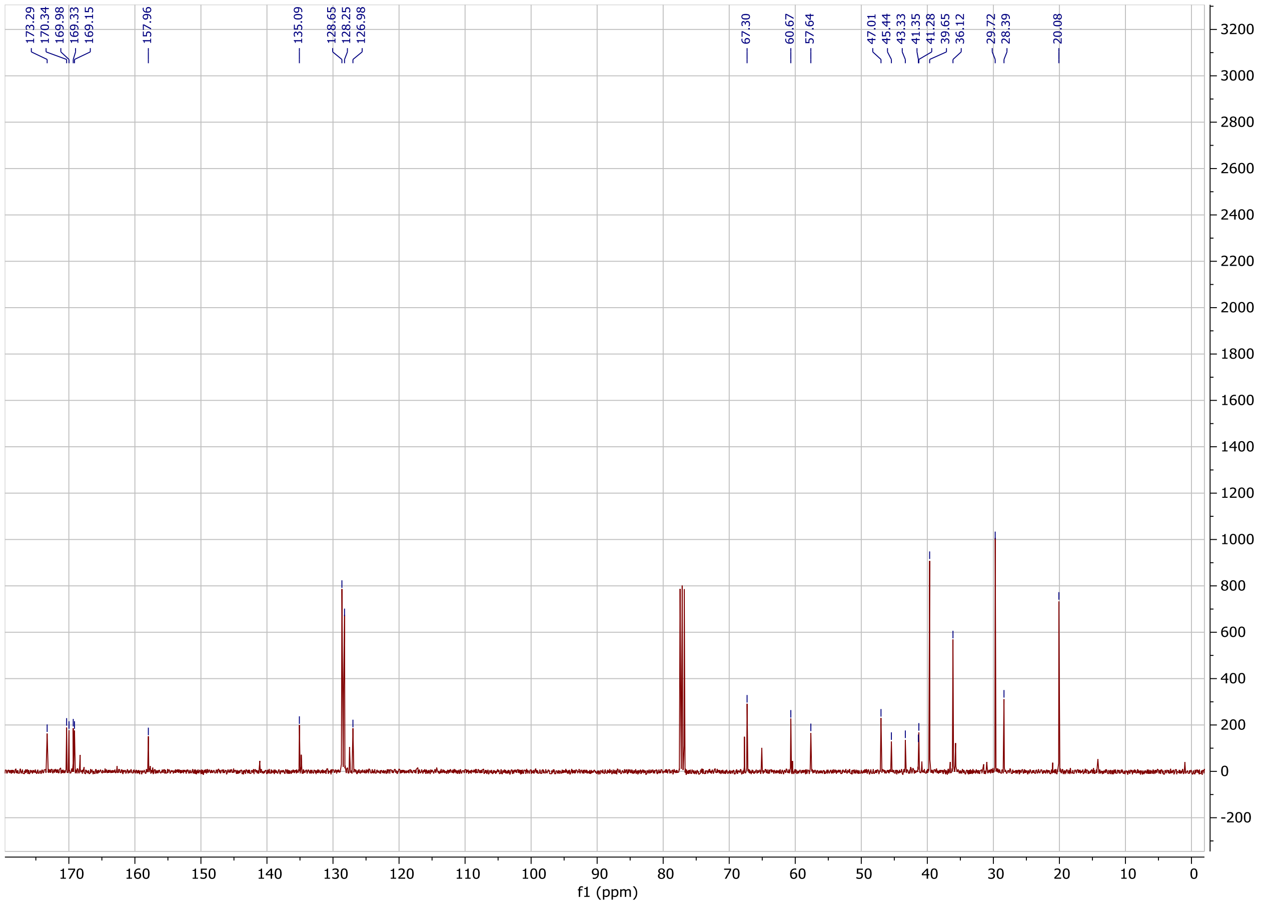

-L.G.S. - Laboratorio Grandi Strumenti - Display Report

Analysis Name	av gc117.d	Acquisition Date	03/06/19 14:49:10	Operator	Walter Panzeri
Sample Name		Method	Copy of _01tmix_posneg lm.MS	Instrument	esquire3000plus
Comment	1 mg/ml dil 1:100 MeOH<br>Richiedente: Cristina				

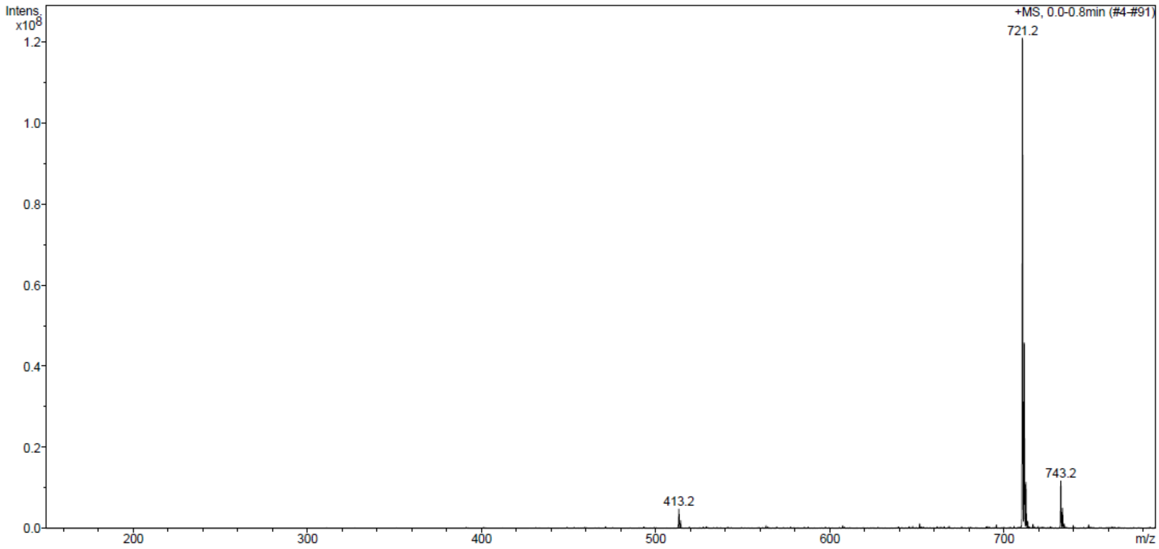

Bruker Daltonics DataAnalysis 3.1 printed: 03/06/19 15:05:46 Page 1 of 1

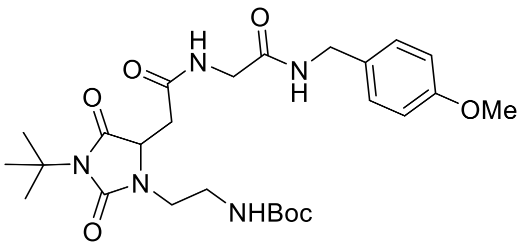

11

^{1}H NMR (400 MHZ, CD_3OD)

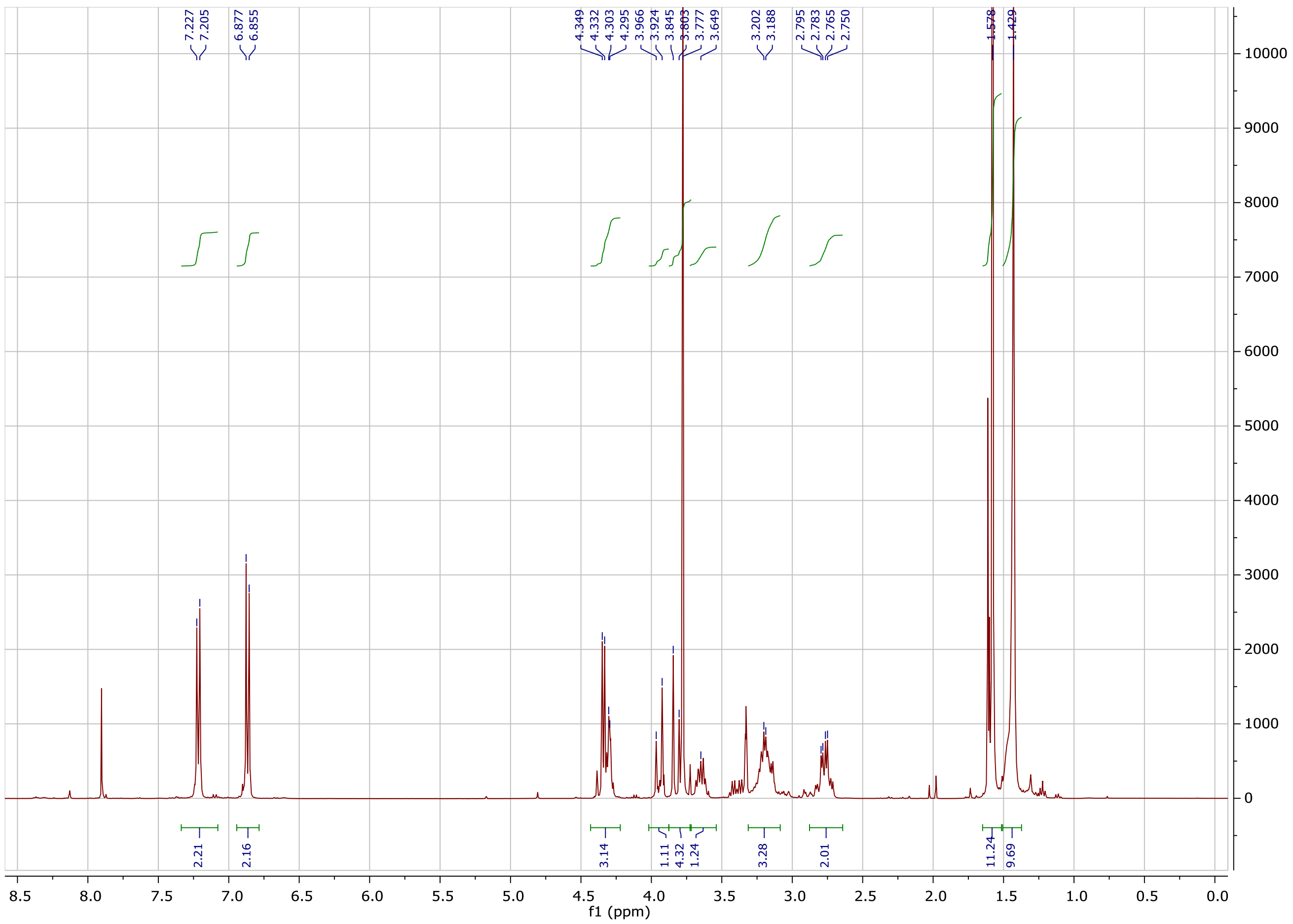

$^{13}C\{^{1}H\}$ NMR (101 MHz, CD_3OD)

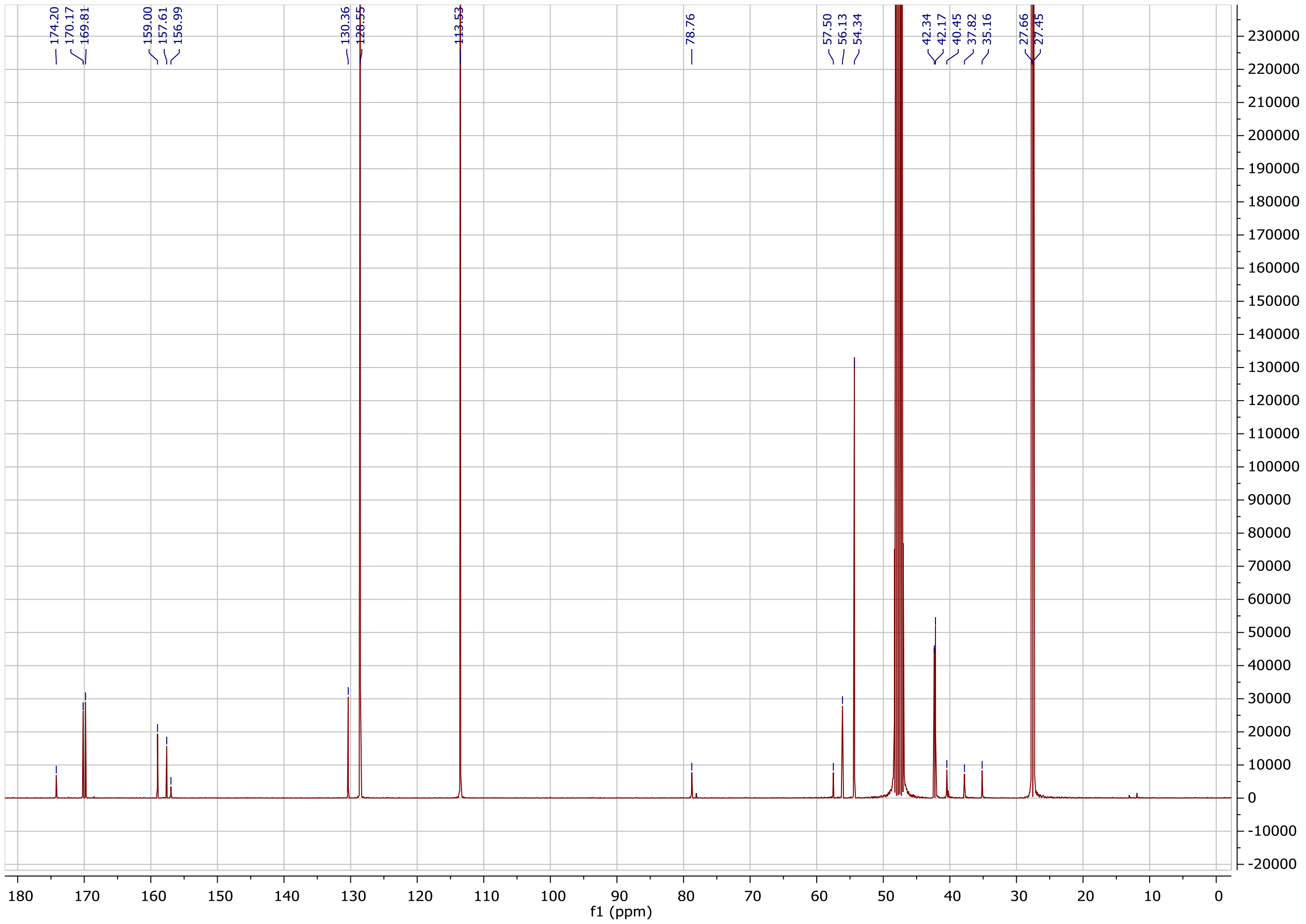

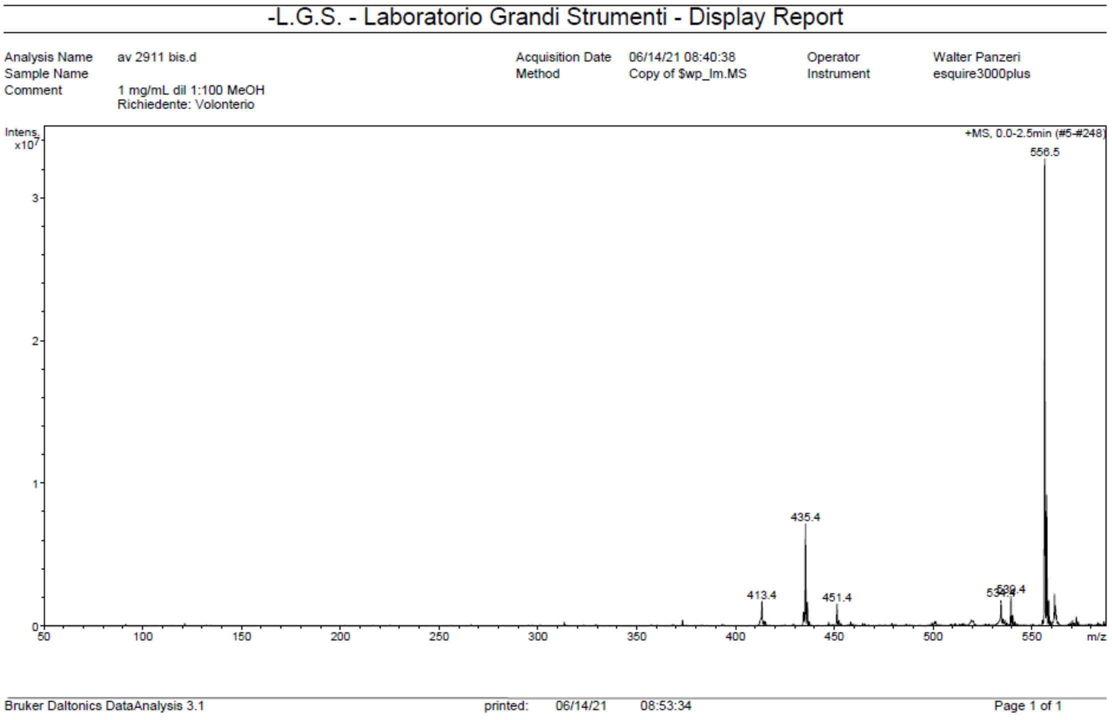

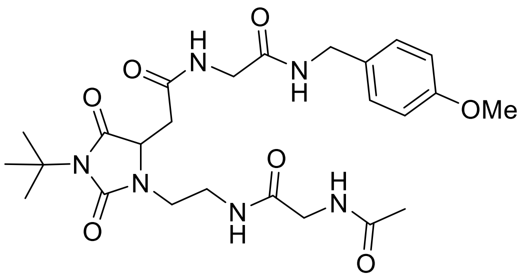

12

^{1}H NMR (400 MHZ, $CDCl_3$)

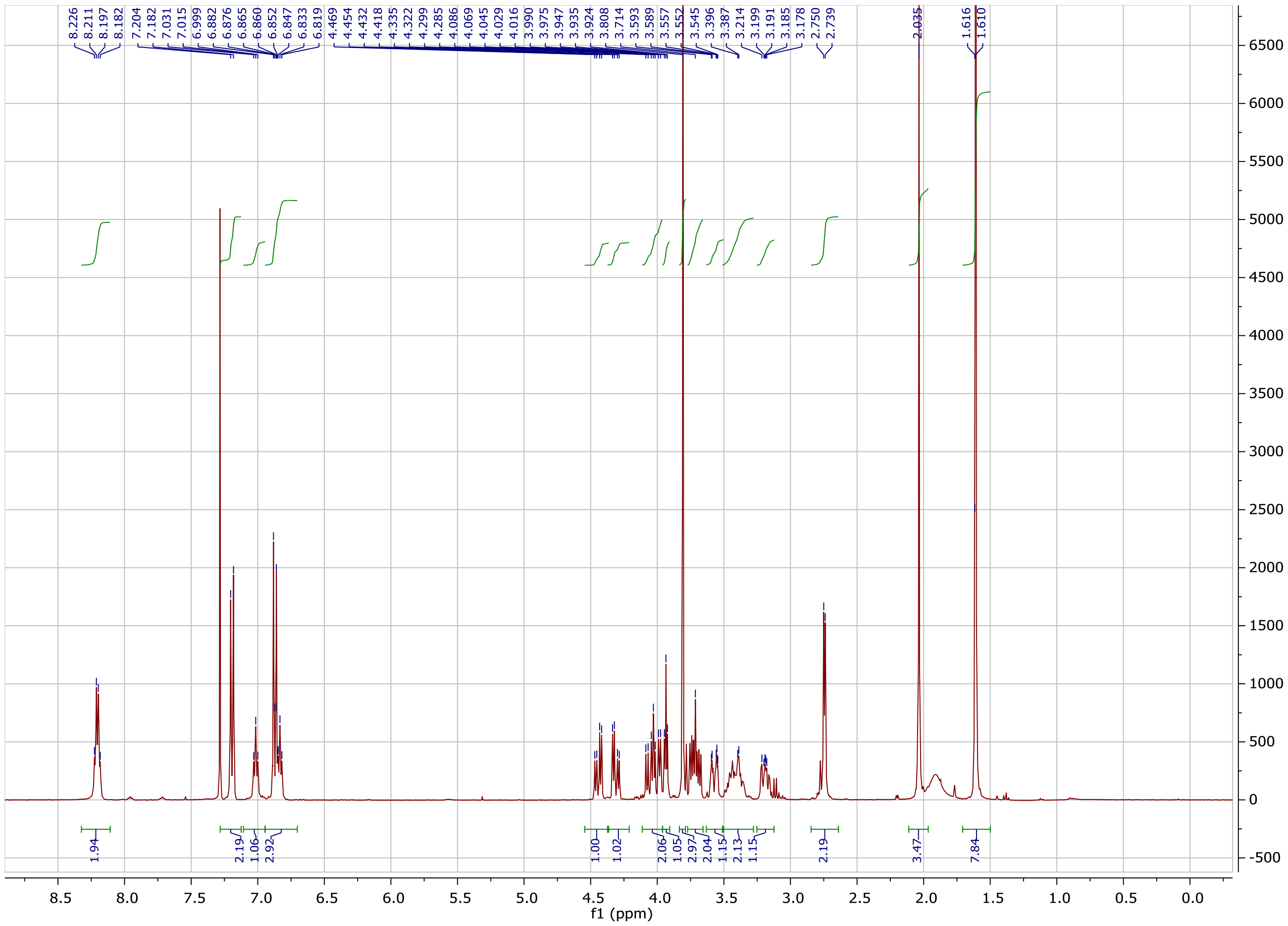

gCOSY NMR (400 MHZ, $CDCl_3$)

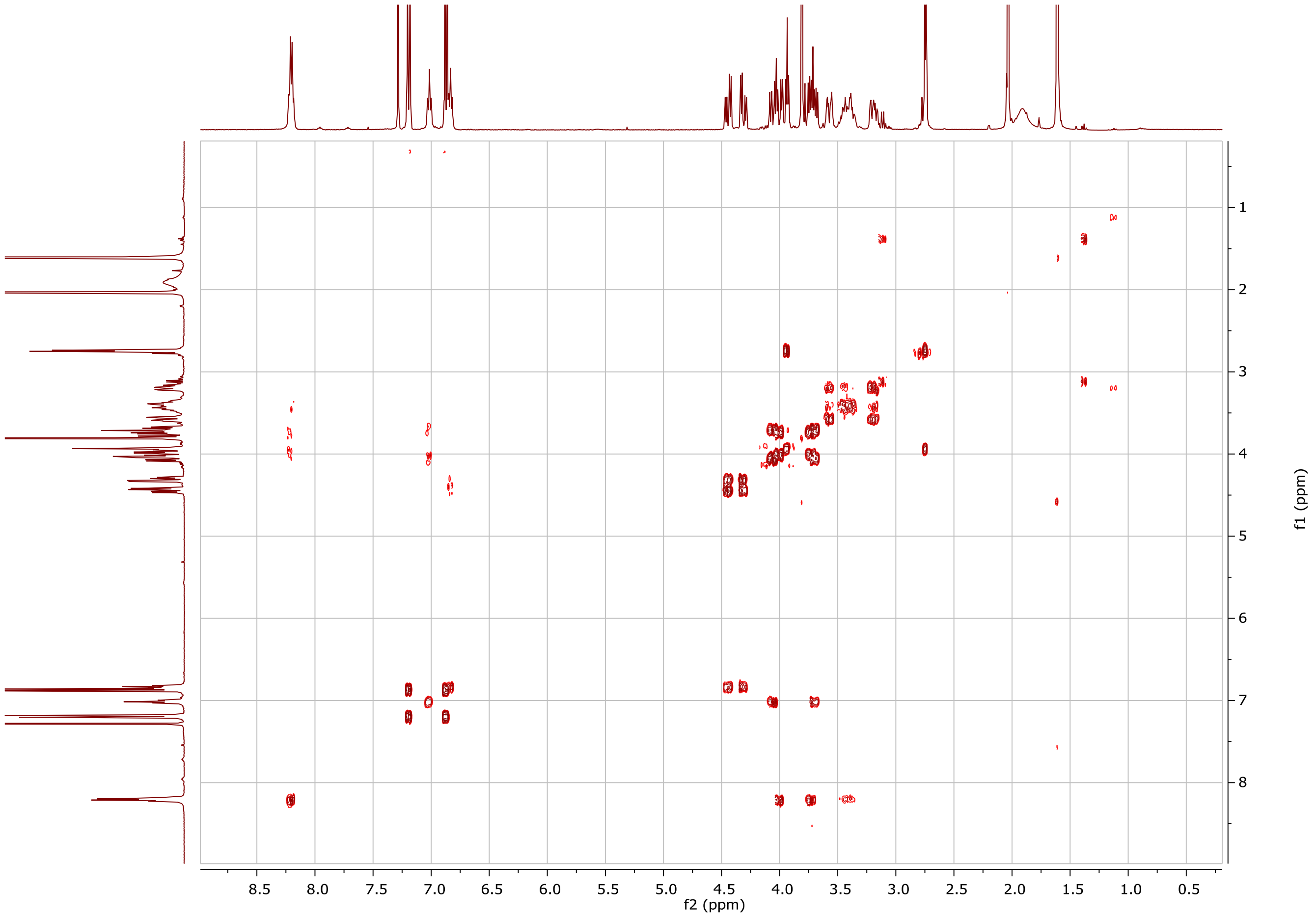

$^{13}C\{^1H\}$ NMR (101 MHz, $CDCl_3$)

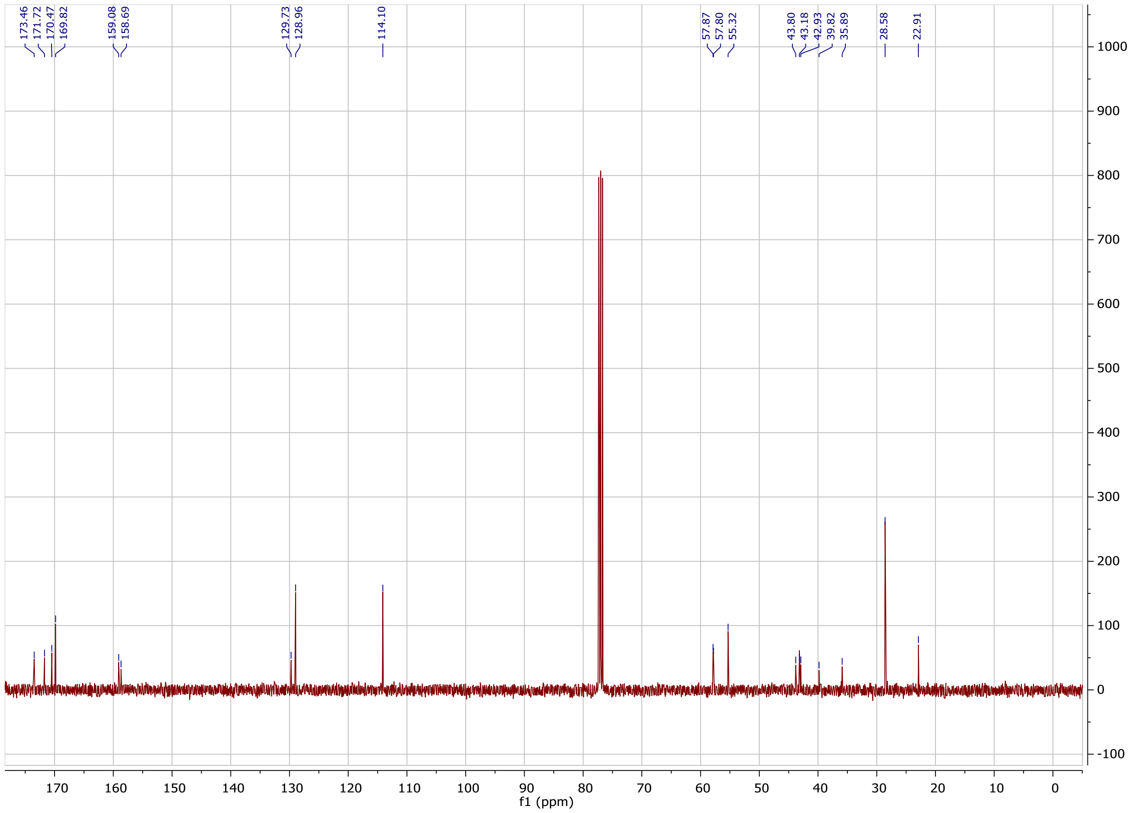

-L.G.S. - Laboratorio Grandi Strumenti - Display Report

Analysis Name	av 2912.d	Acquisition Date	06/11/21 10:10:11	Operator	Walter Panzeri
Sample Name		Method	Copy of $wp_lm.MS	Instrument	esquire3000plus
Comment	1 mg/mL dil 1:100 MeOH<br>Richiedente: Volonterio				

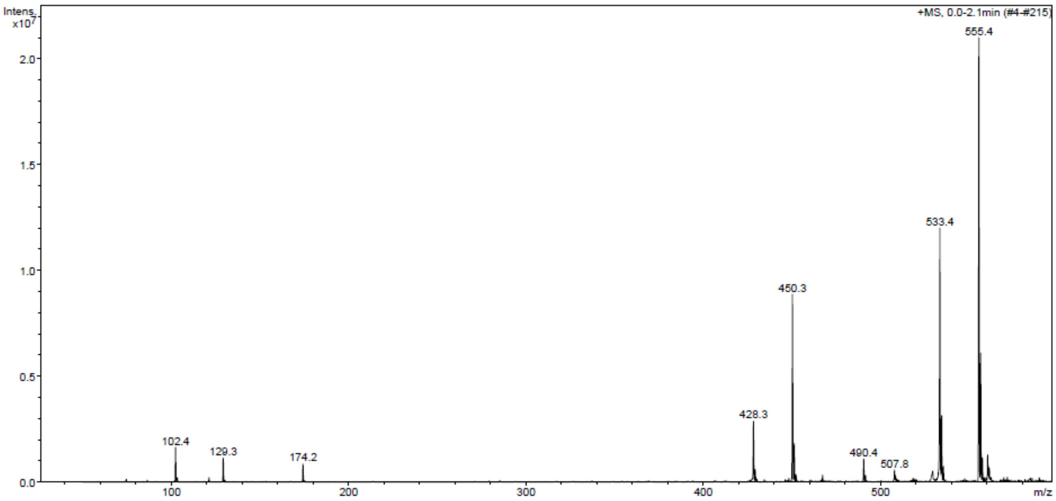

Bruker Daltonics DataAnalysis 3.1 printed: 06/11/21 10:23:30 Page 1 of 1

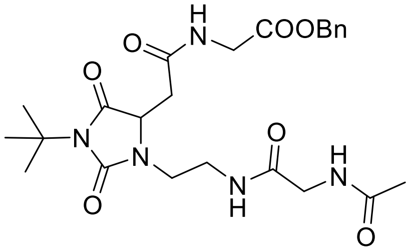

13

^{1}H NMR (400 MHZ, $CDCl_3$)

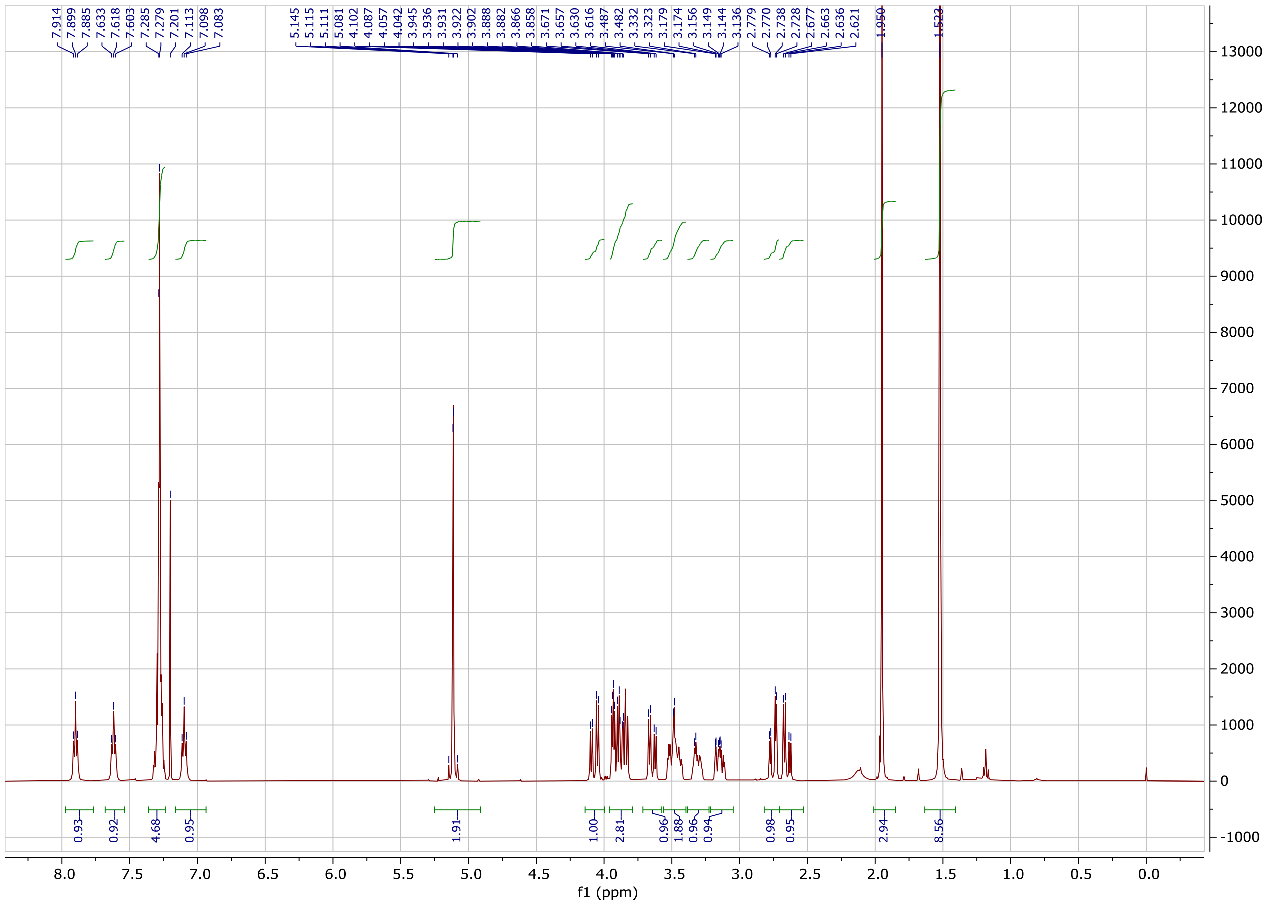

$^{13}C\{^{1}H\}$ NMR (101 MHz, $CDCl_3$)

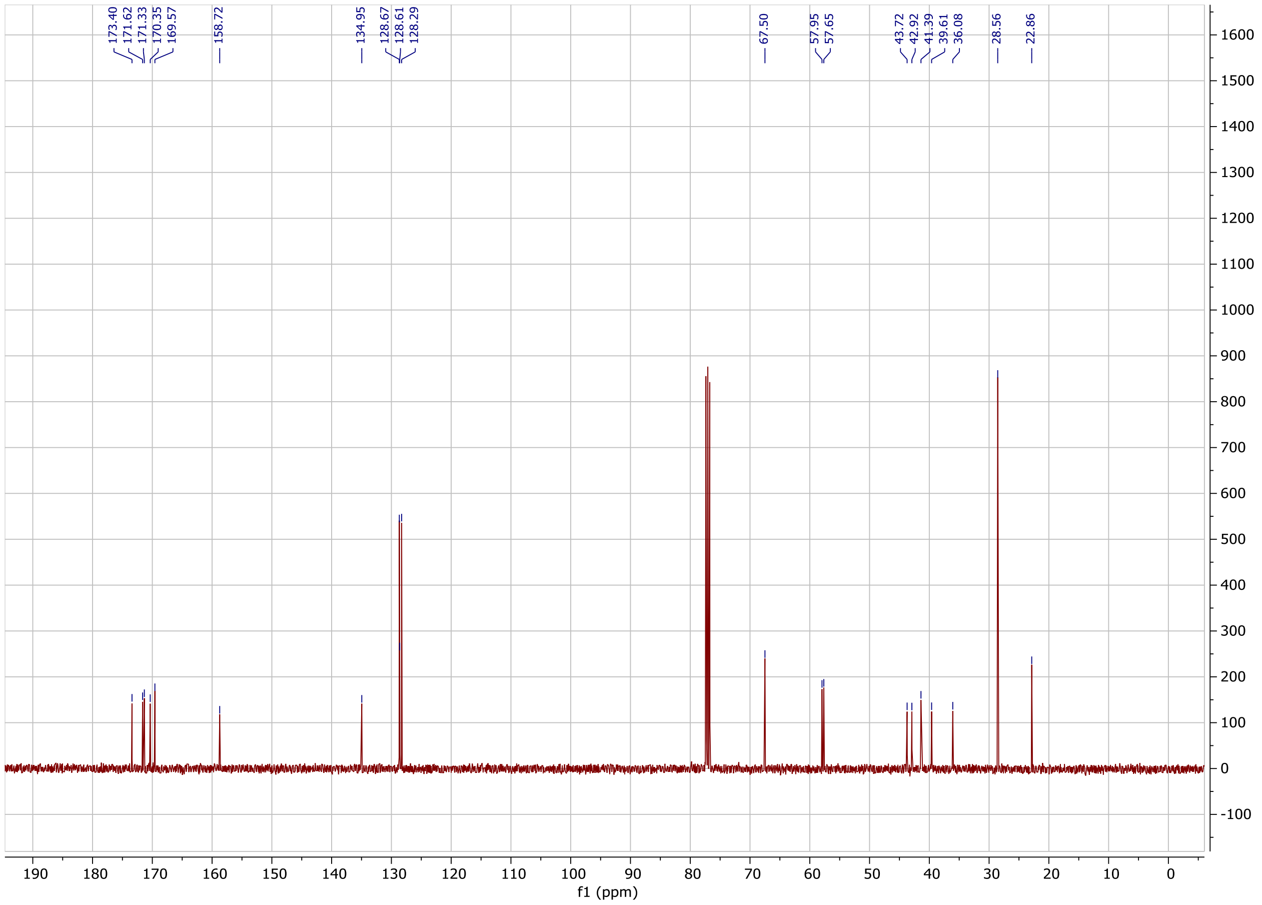

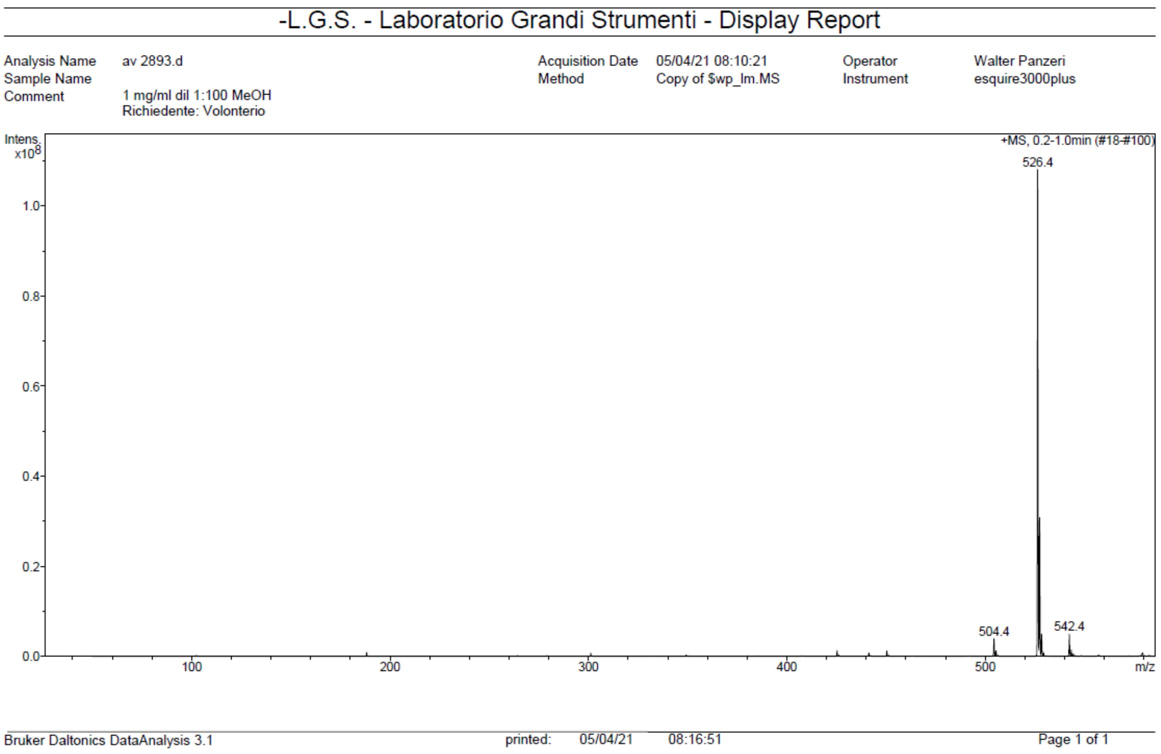

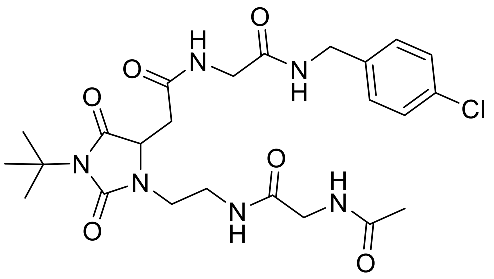

14

^{1}H NMR (400 MHZ, $CDCl_3$)

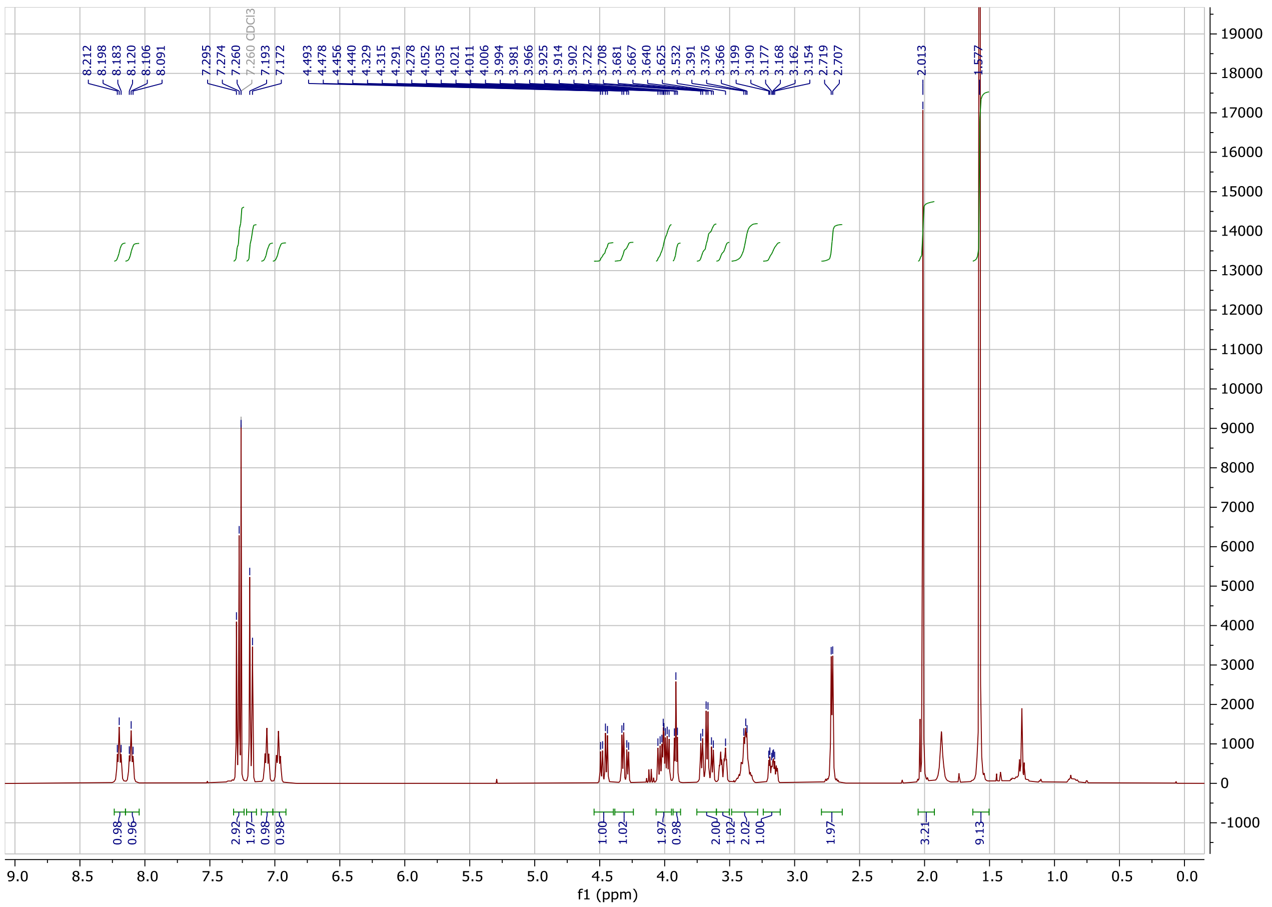

gCOSY NMR (400 MHZ, $CDCl_3$)

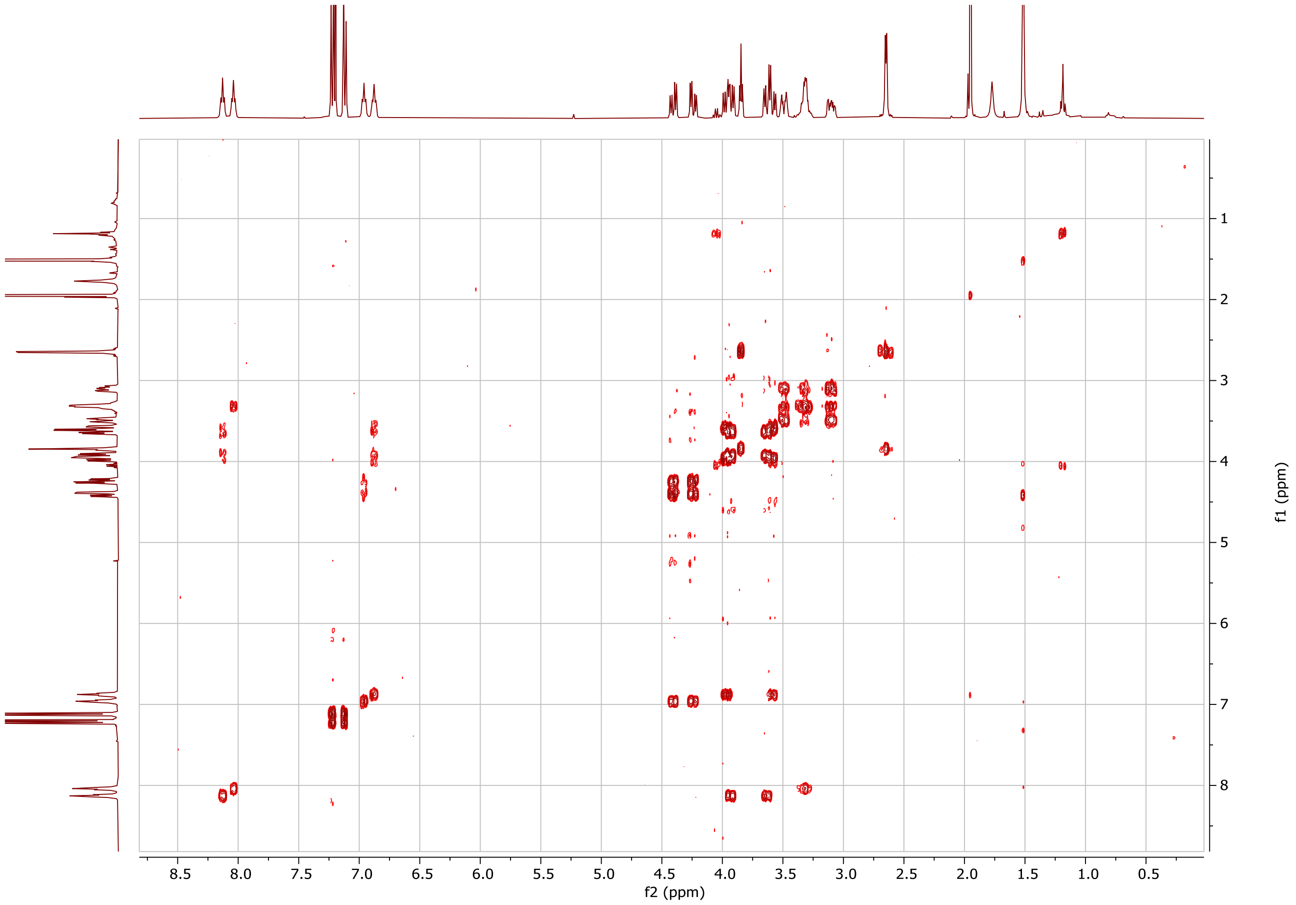

$^{13}C\{^1H\}$ NMR (101 MHz, $CDCl_3$)

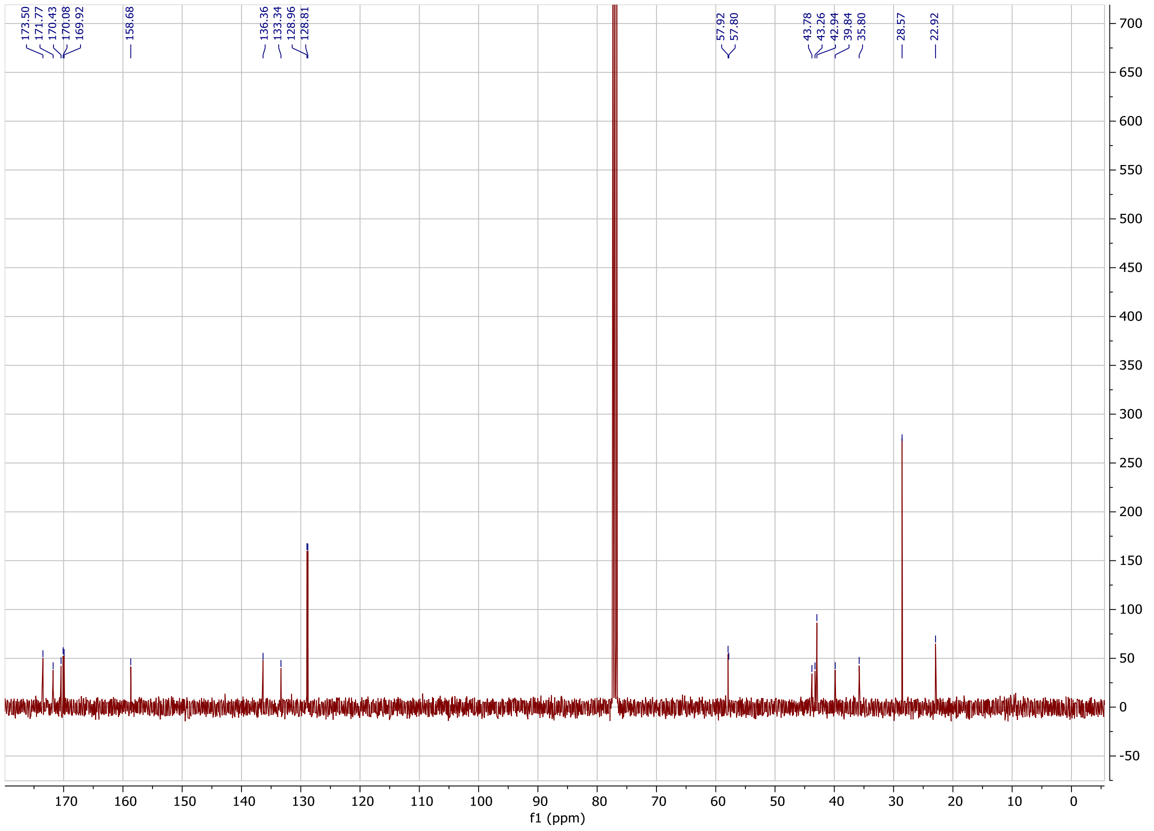

-L.G.S. - Laboratorio Grandi Strumenti - Display Report

Analysis Name	av gc85a.d	Acquisition Date	10/02/18 13:53:06	Operator	Walter Panzeri
Sample Name		Method	Copy of _01tmix_posneg lm.MS	Instrument	esquire3000plus
Comment	1 mg/ml dil 1:100 MeOH Richiedente Cristina				

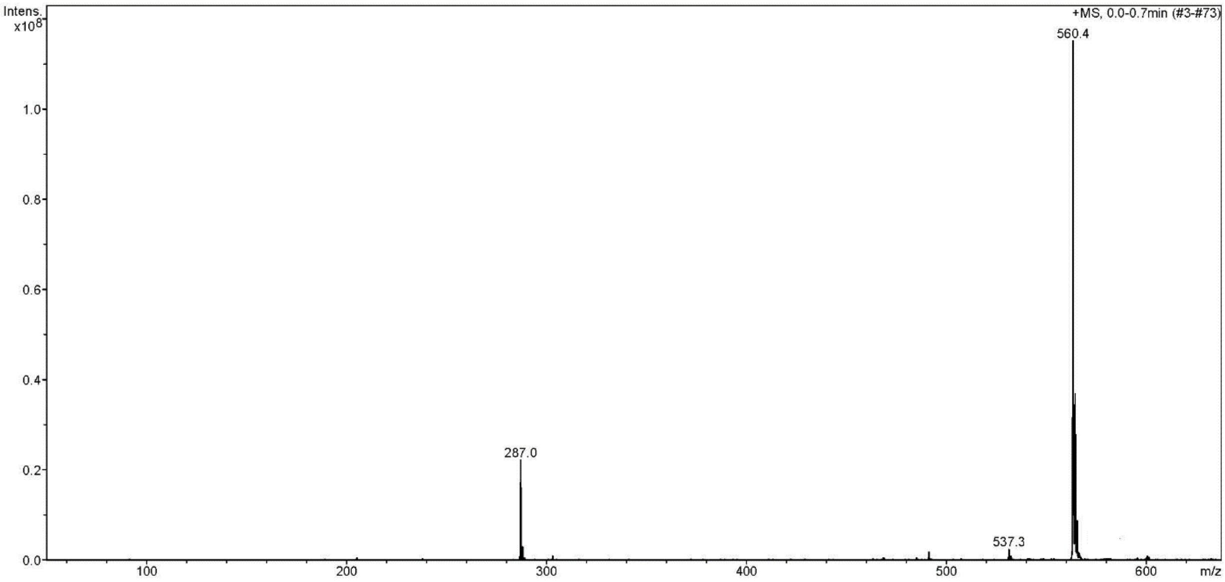

Bruker Daltonics DataAnalysis 3.1 printed: 10/02/18 13:54:50 Page 1 of 1

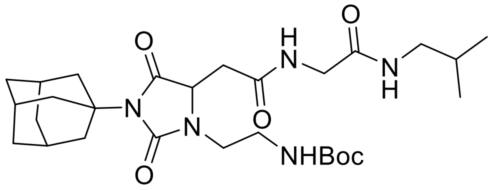

15

^{1}H NMR (400 MHZ, $CDCl_3$)

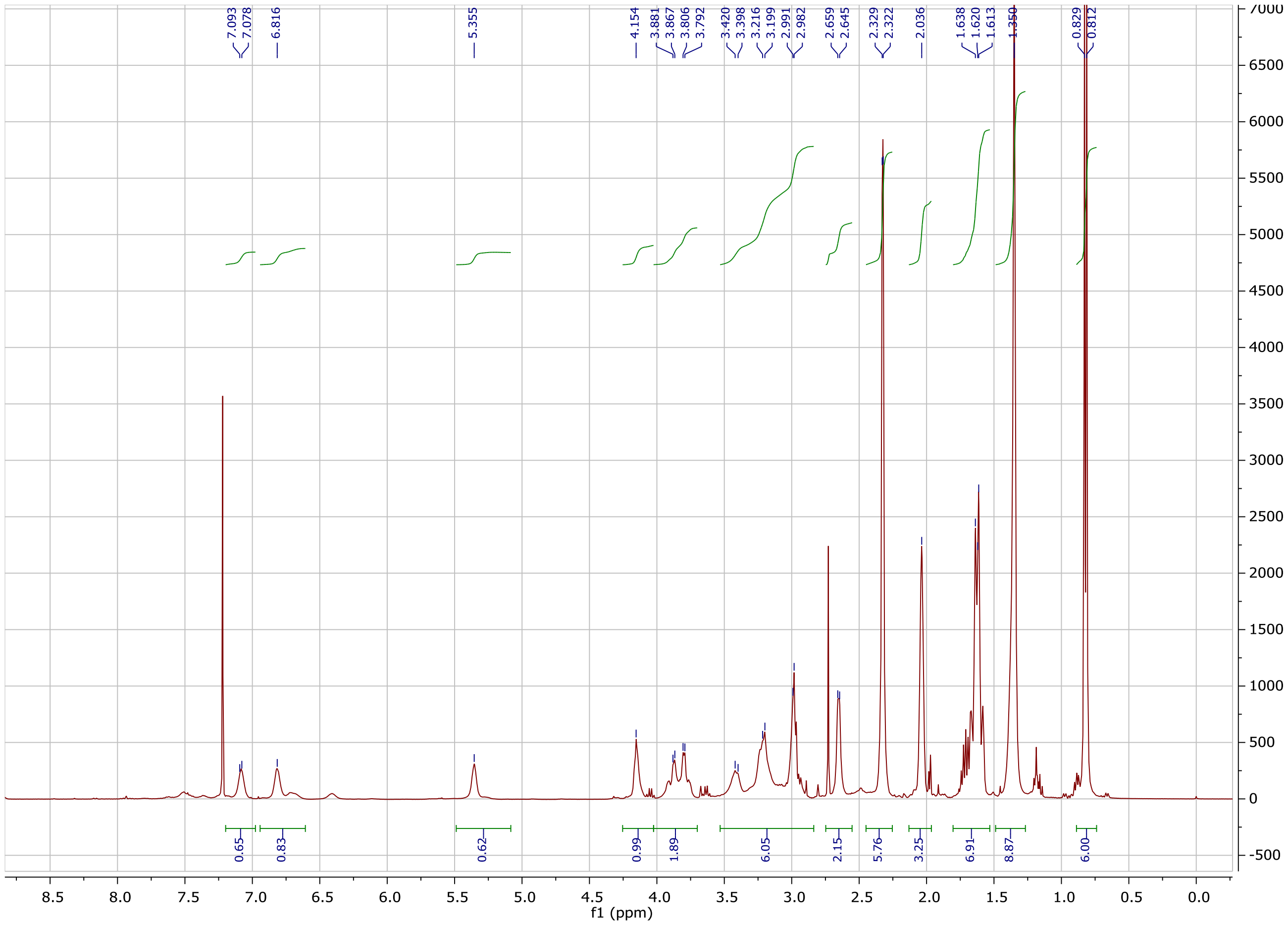

$^{13}C\{^1H\}$ NMR (101 MHz, $CDCl_3$)

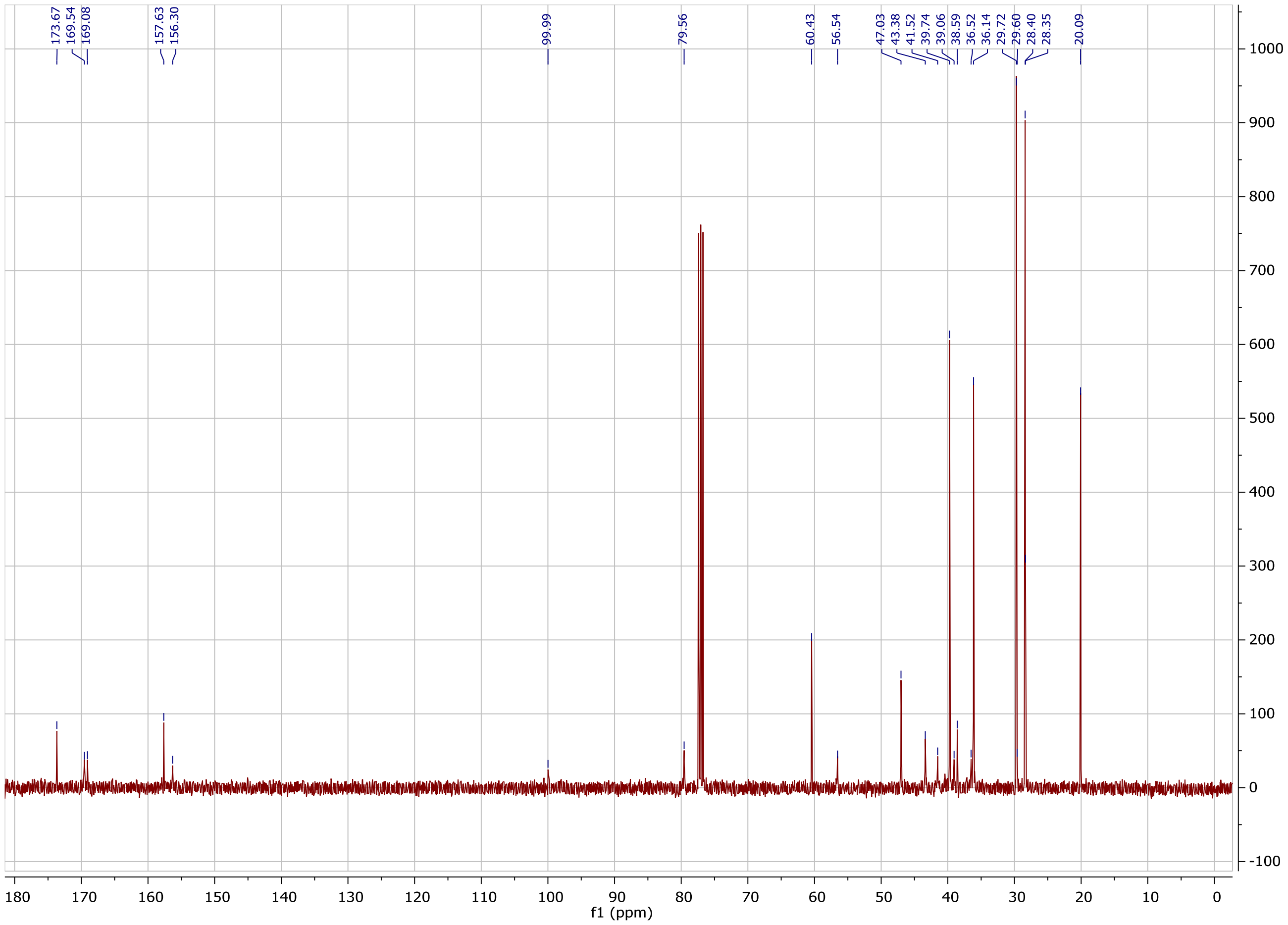

-L.G.S. - Laboratorio Grandi Strumenti - Display Report

Analysis Name	av 2926.d	Acquisition Date	07/20/21 13:58:10	Operator	Walter Panzeri
Sample Name		Method	Copy of $wp_lm.MS	Instrument	esquire3000plus
Comment	1 mg/mL dil 1:100 MeOH<br>Richiedente: Volonterio				

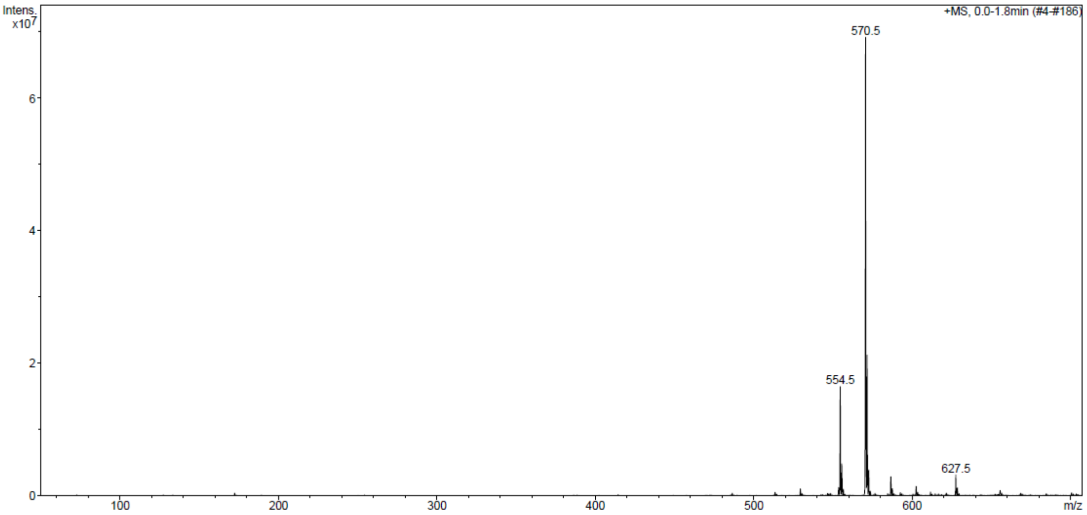

Bruker Daltonics DataAnalysis 3.1 printed: 07/20/21 14:01:44 Page 1 of 1

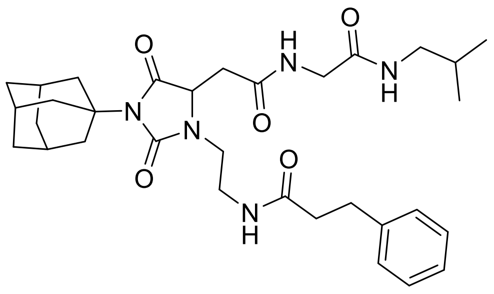

16

^{1}H NMR (400 MHZ, $CDCl_3$)

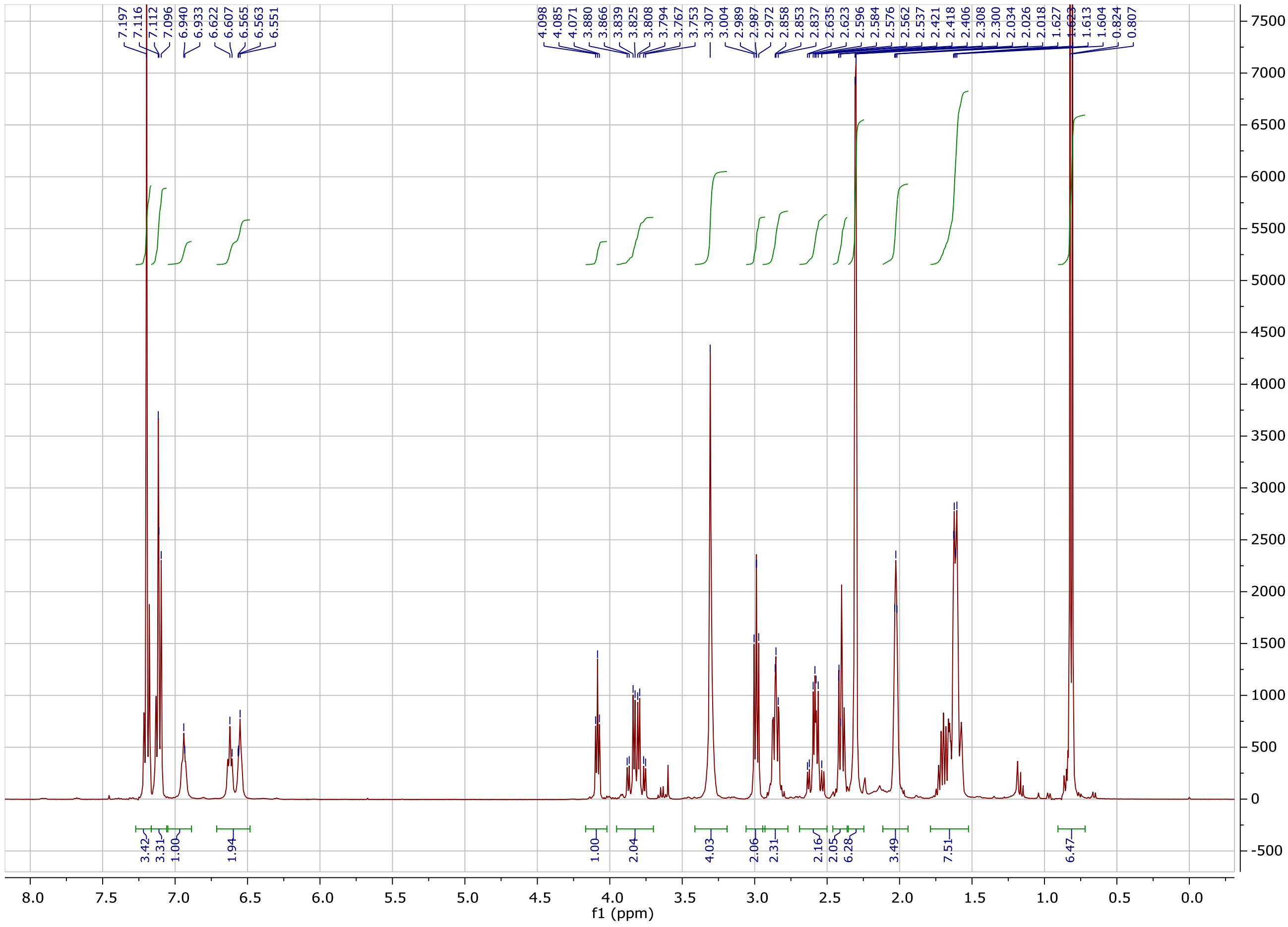

gCOSY NMR (400 MHZ, $CDCl_3$)

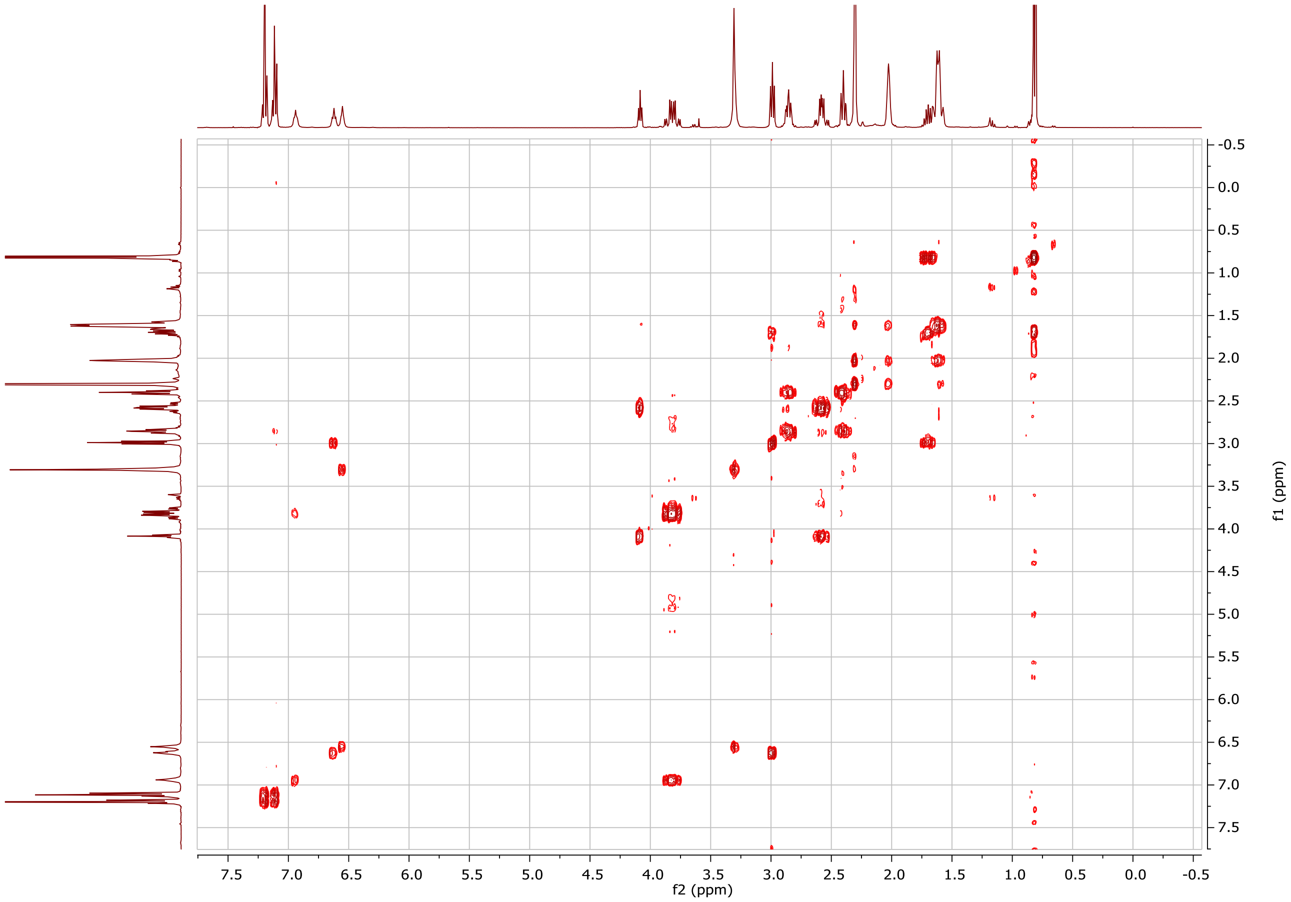

$^{13}C\{^1H\}$ NMR (101 MHz, $CDCl_3$)

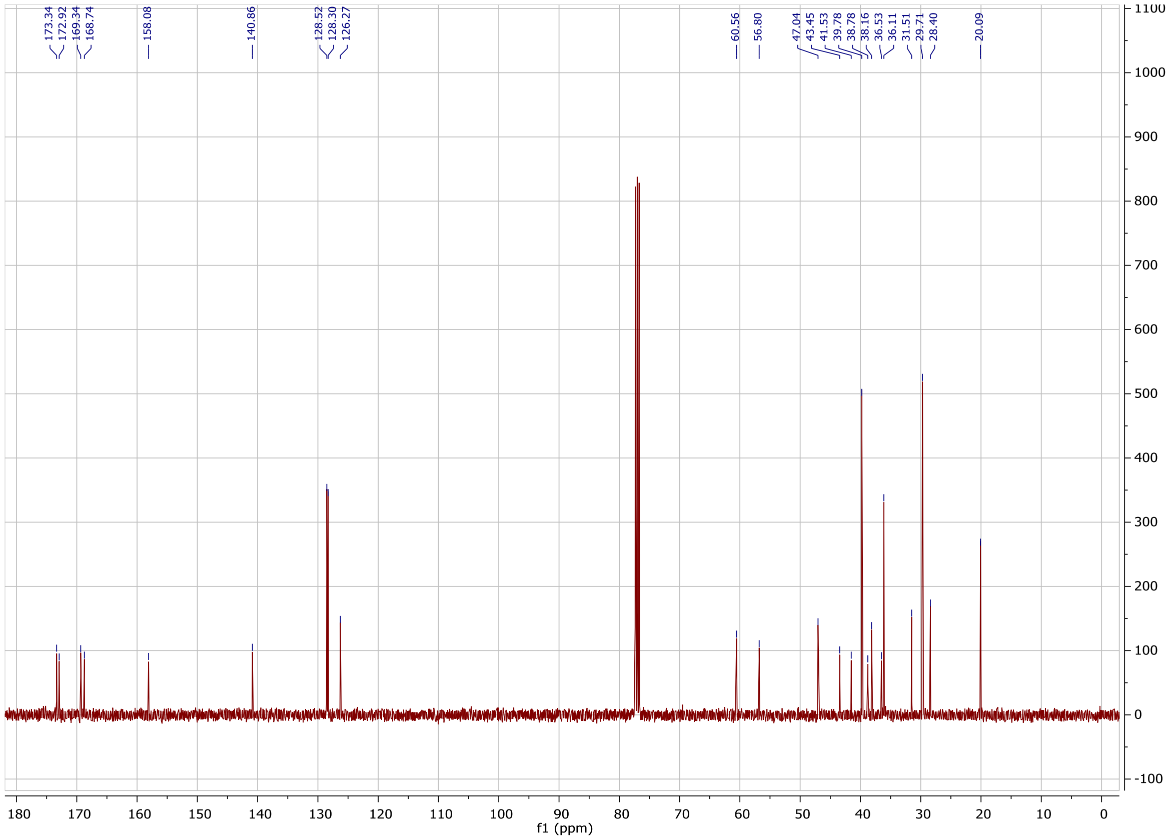

-L.G.S. - Laboratorio Grandi Strumenti - Display Report

Analysis Name	av 2927.d	Acquisition Date	07/20/21 13:33:40	Operator	Walter Panzeri
Sample Name		Method	Copy of $wp_lm.MS	Instrument	esquire3000plus
Comment	1 mg/mL dil 1:100 MeOH<br>Richiedente: Volonterio				

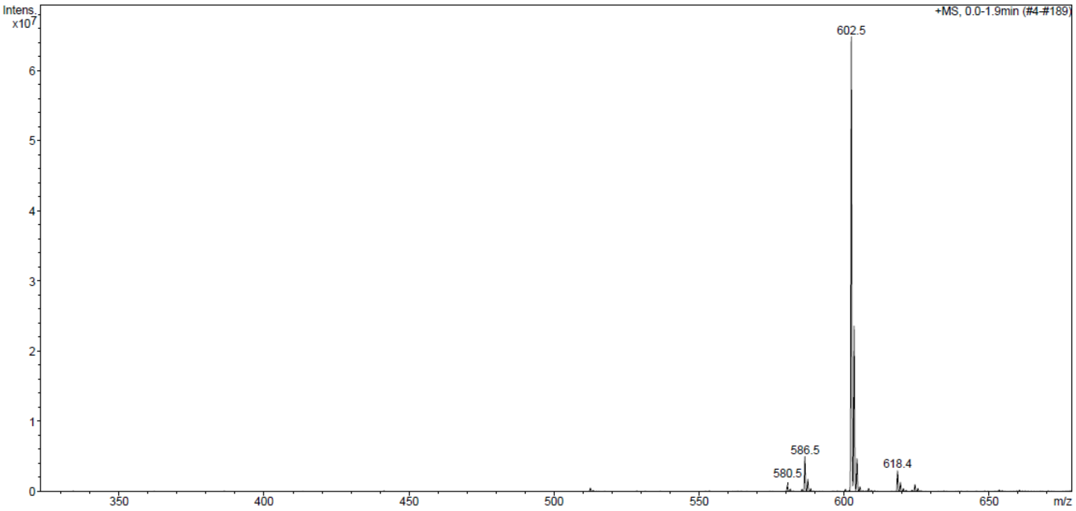

Bruker Daltonics DataAnalysis 3.1 printed: 07/20/21 13:52:31 Page 1 of 1

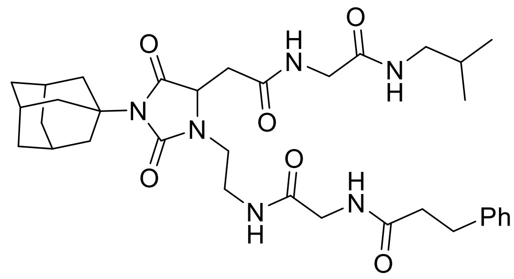

17

^{1}H NMR (400 MHZ, CDCl$_3$)

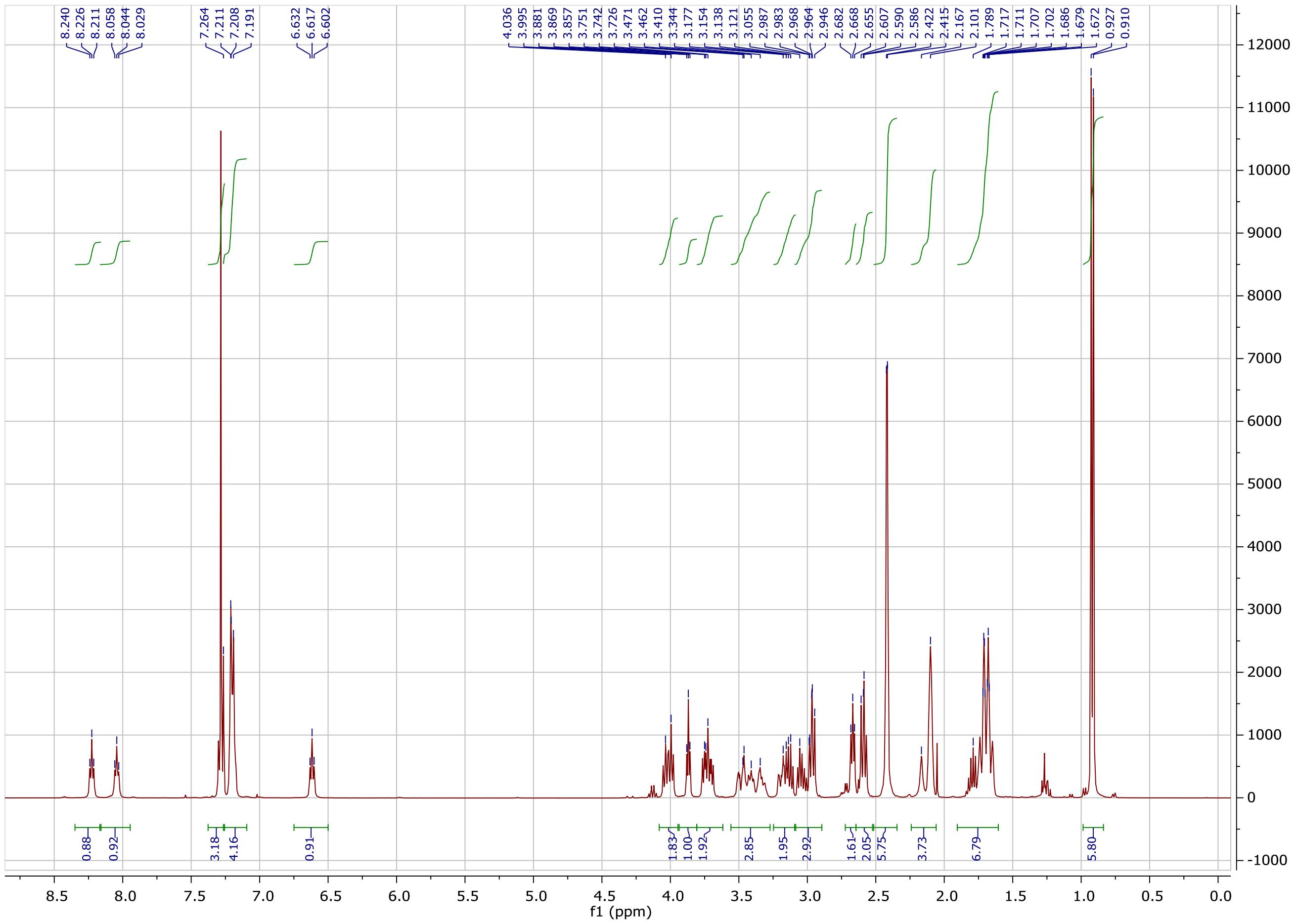

gCOSY NMR (400 MHZ, $CDCl_3$)

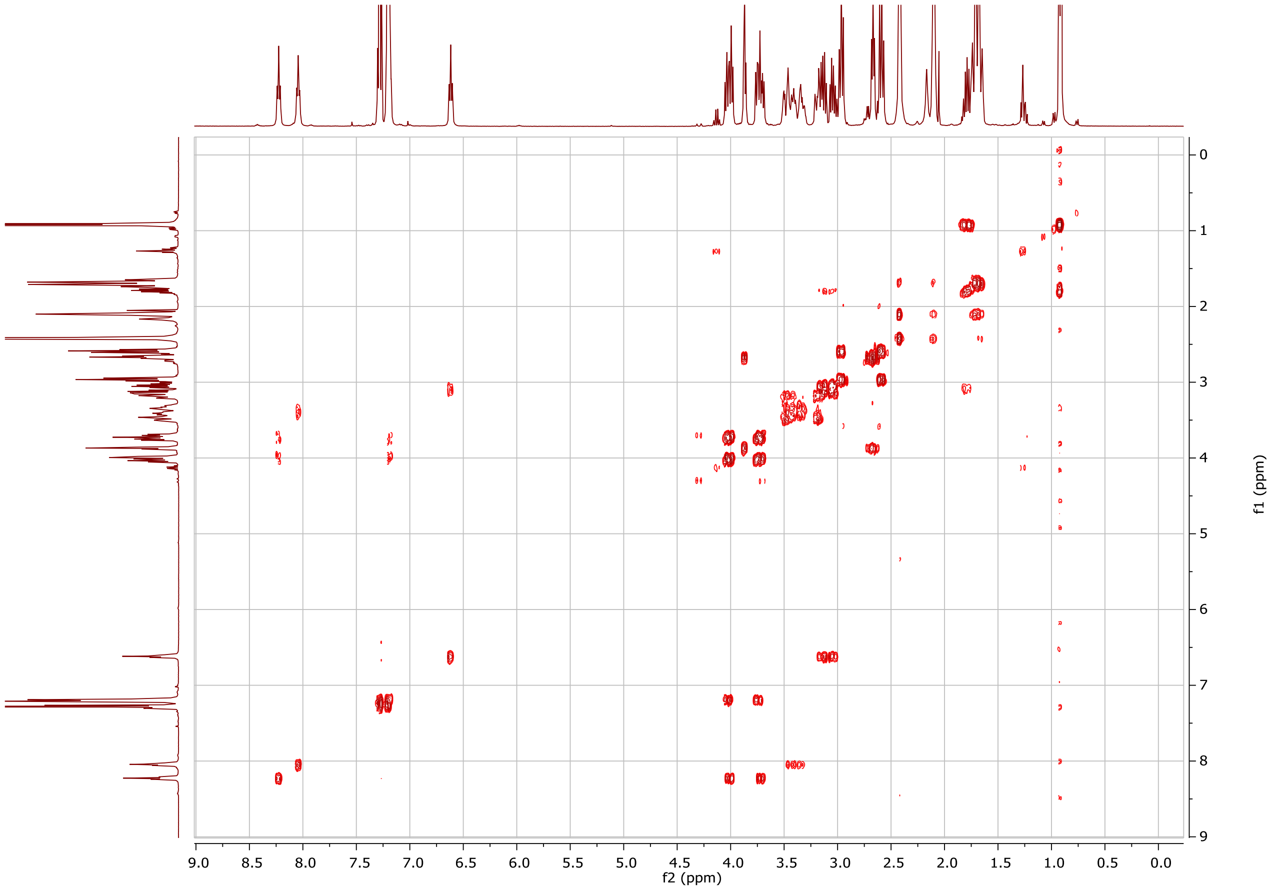

$^{13}C\{^{1}H\}$ NMR (101 MHz, $CDCl_3$)

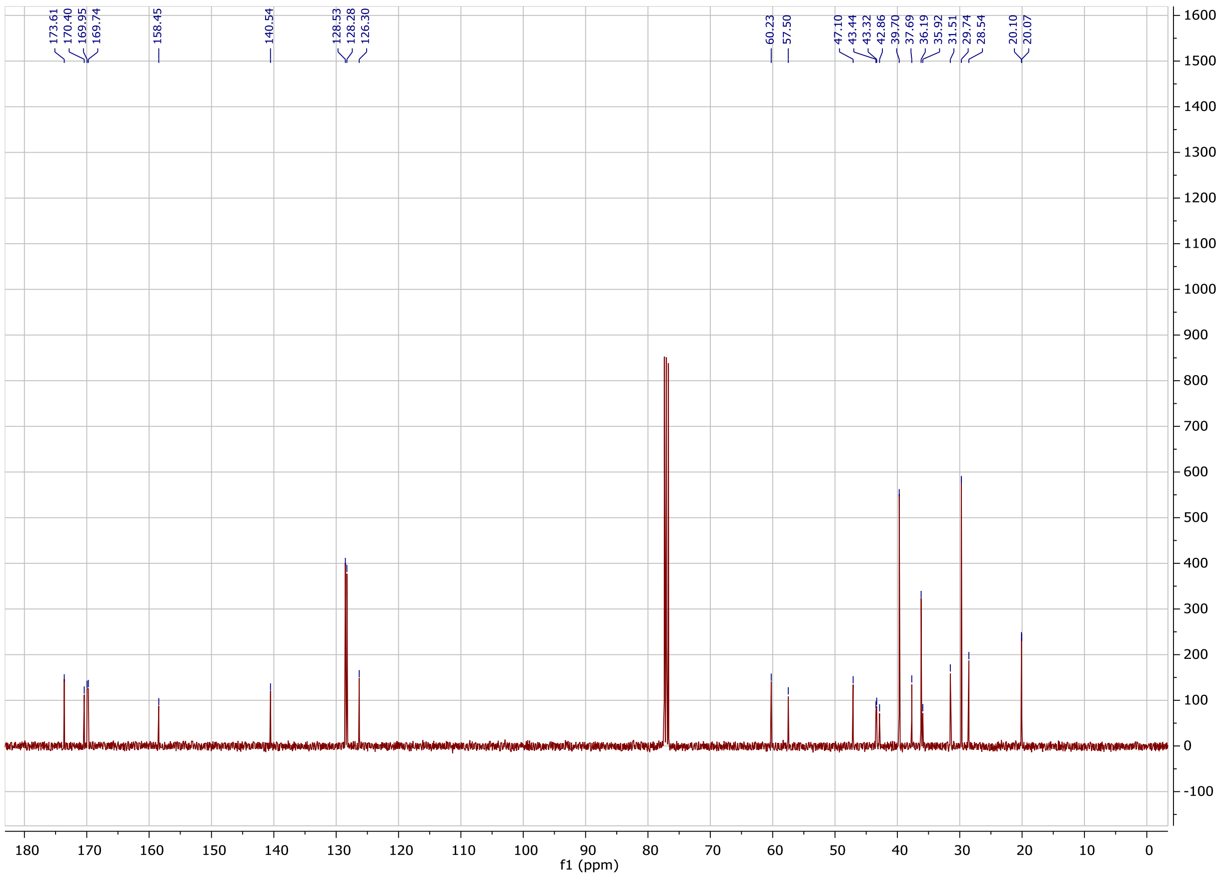

-L.G.S. - Laboratorio Grandi Strumenti - Display Report

Analysis Name	av md20g_f.d	Acquisition Date	04/24/19 14:19:55	Operator	Walter Panzeri
Sample Name		Method	Copy of _01tmix_posneg lm.MS	Instrument	esquire3000plus
Comment	1 mg/ml dil 1:100 CH3CN<br>Richiedente: Viani				

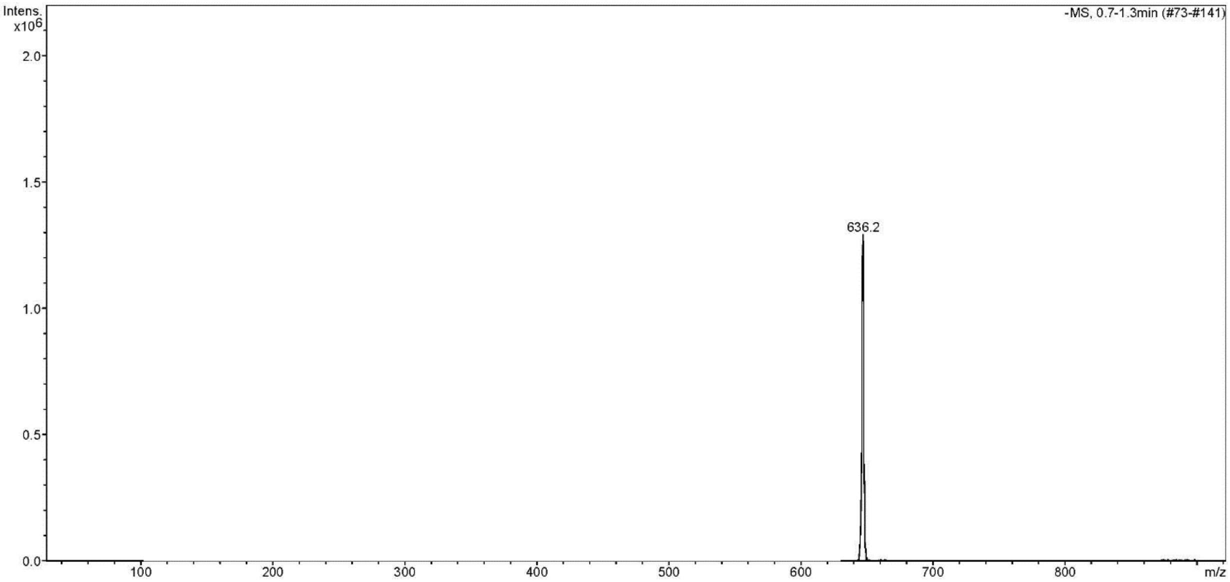

Bruker Daltonics DataAnalysis 3.1 printed: 04/24/19 14:50:09 Page 1 of 1

Figure S1. VT ^{1}H NMR spectra of **6a** in $CDCl_3$ (400 MHz)

From the bottom to the top: a) ^{1}H NMR in $CDCl_3$ at 302 K, b) ^{1}H NMR in $CDCl_3$ at 306 K, c) ^{1}H NMR in $CDCl_3$ at 310 K, d) ^{1}H NMR in $CDCl_3$ at 314 K, e) ^{1}H NMR in $CDCl_3$ at 318 K.

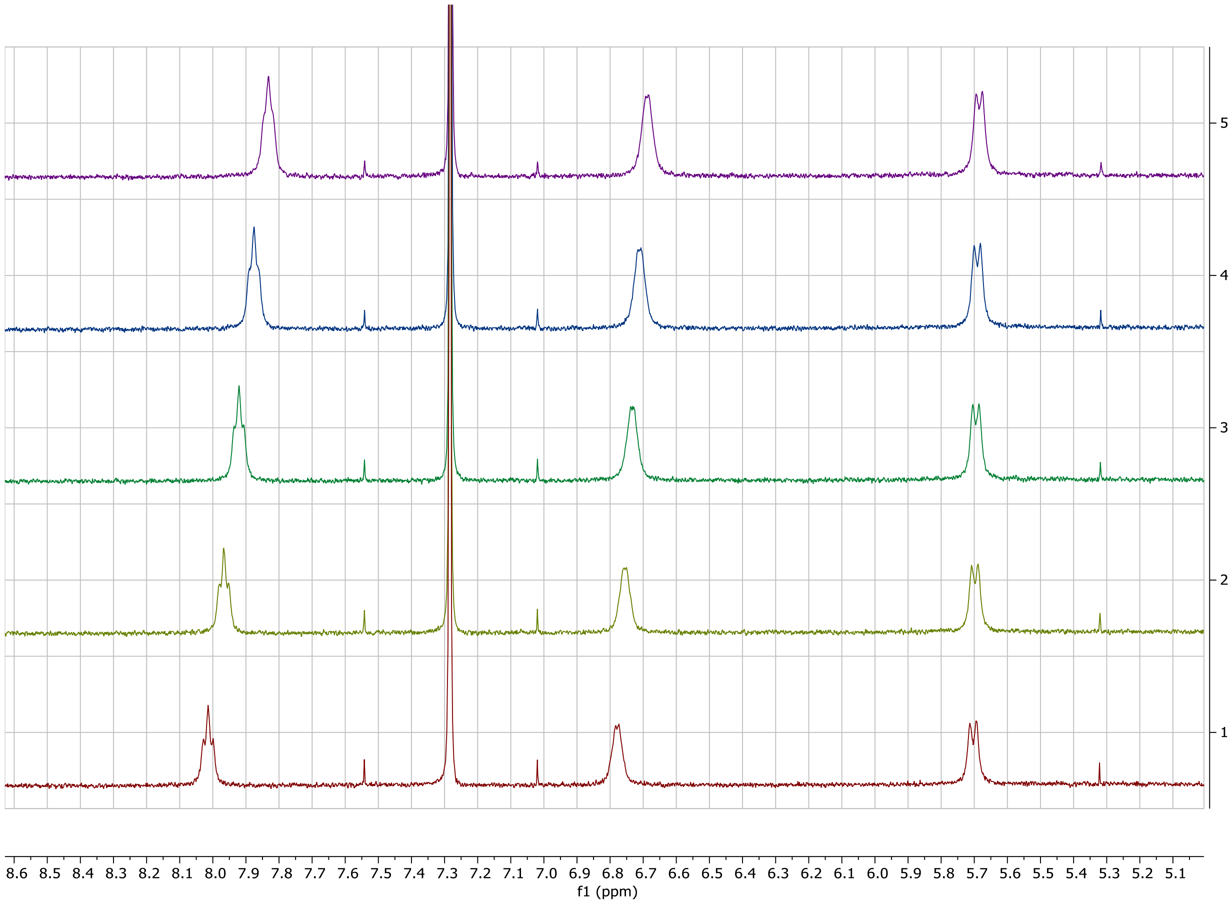

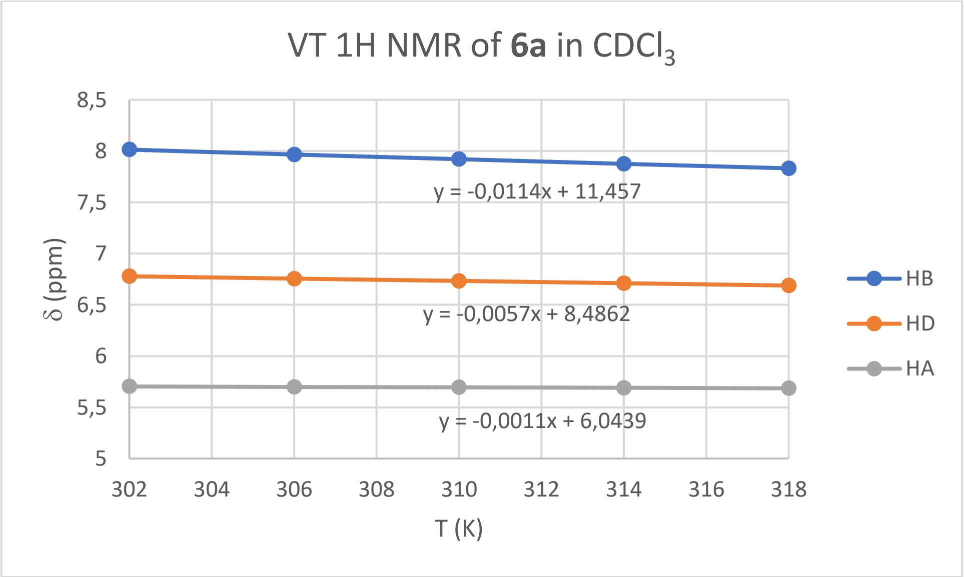

Figure S2. VT ^{1}H NMR spectra of **6a** in dmso-d_6 (400 MHz)

From the bottom to the top: a) ^{1}H NMR in dmso-d_6 at 302 K, b) ^{1}H NMR in dmso-d_6 at 306 K, c) ^{1}H NMR in dmso-d_6 at 310 K, d) ^{1}H NMR in dmso-d_6 at 314 K, e) ^{1}H NMR in dmso-d_6 at 318 K.

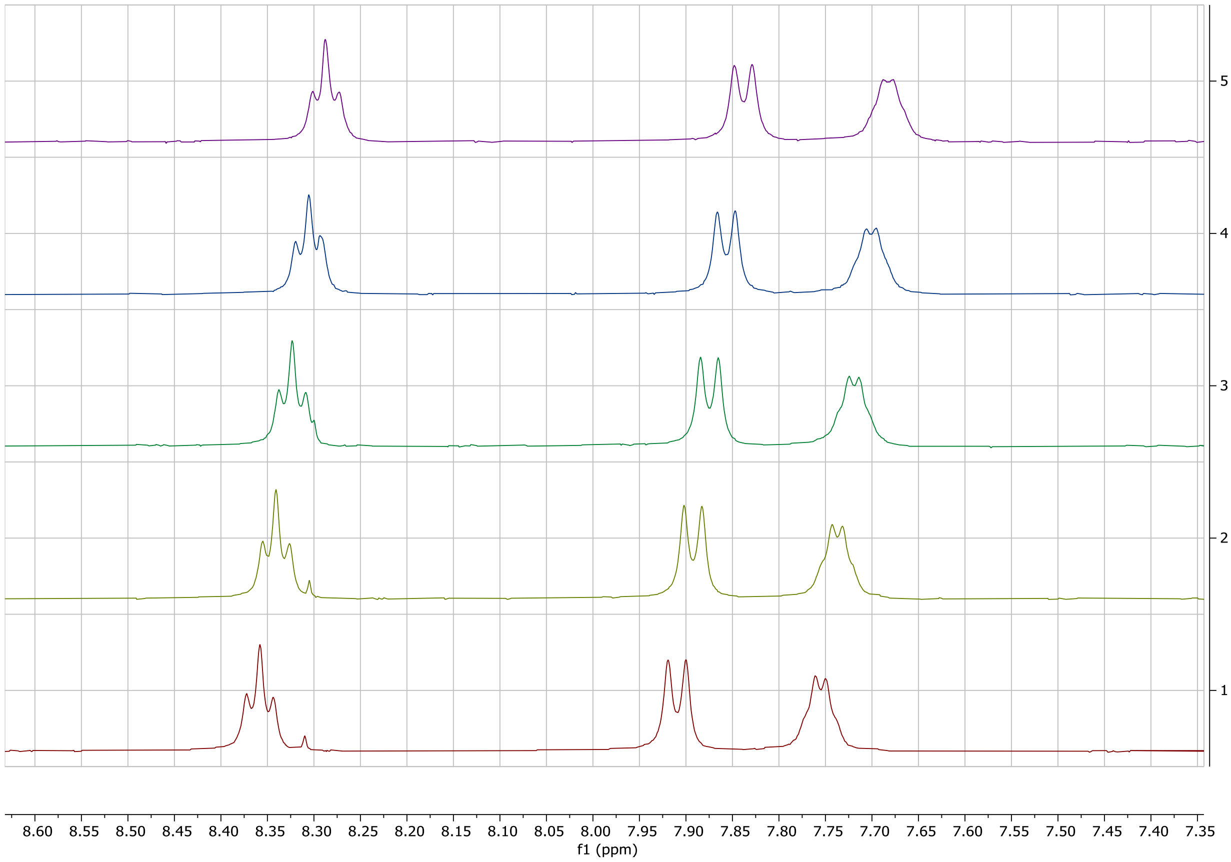

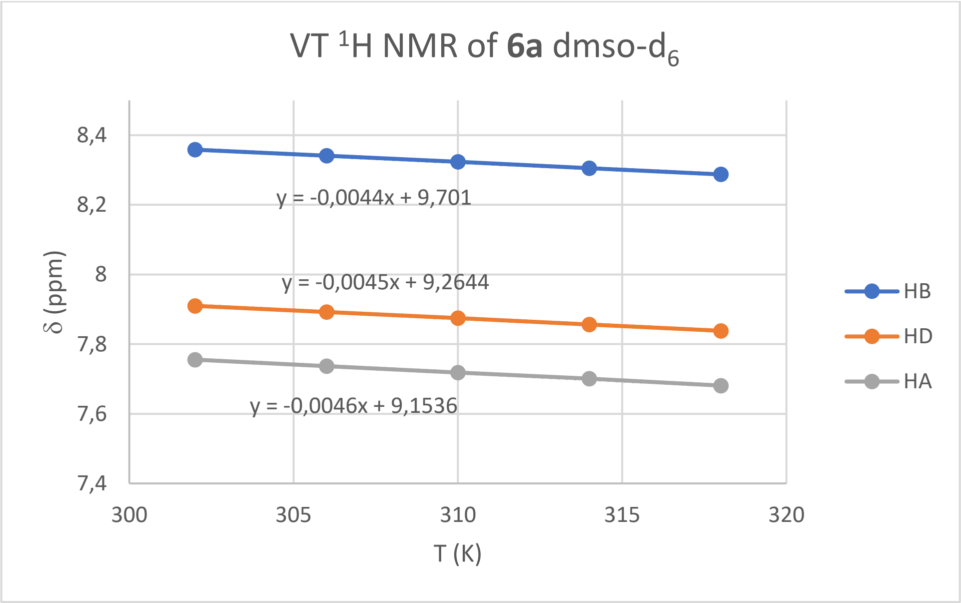

Figure S3. VT ^{1}H NMR spectra of **8** in $CDCl_3$ (400 MHz)

From the bottom to the top: a) ^{1}H NMR in $CDCl_3$ at 302 K, b) ^{1}H NMR in $CDCl_3$ at 306 K, c) ^{1}H NMR in $CDCl_3$ at 310 K, d) ^{1}H NMR in $CDCl_3$ at 314 K, e) ^{1}H NMR in $CDCl_3$ at 318 K.

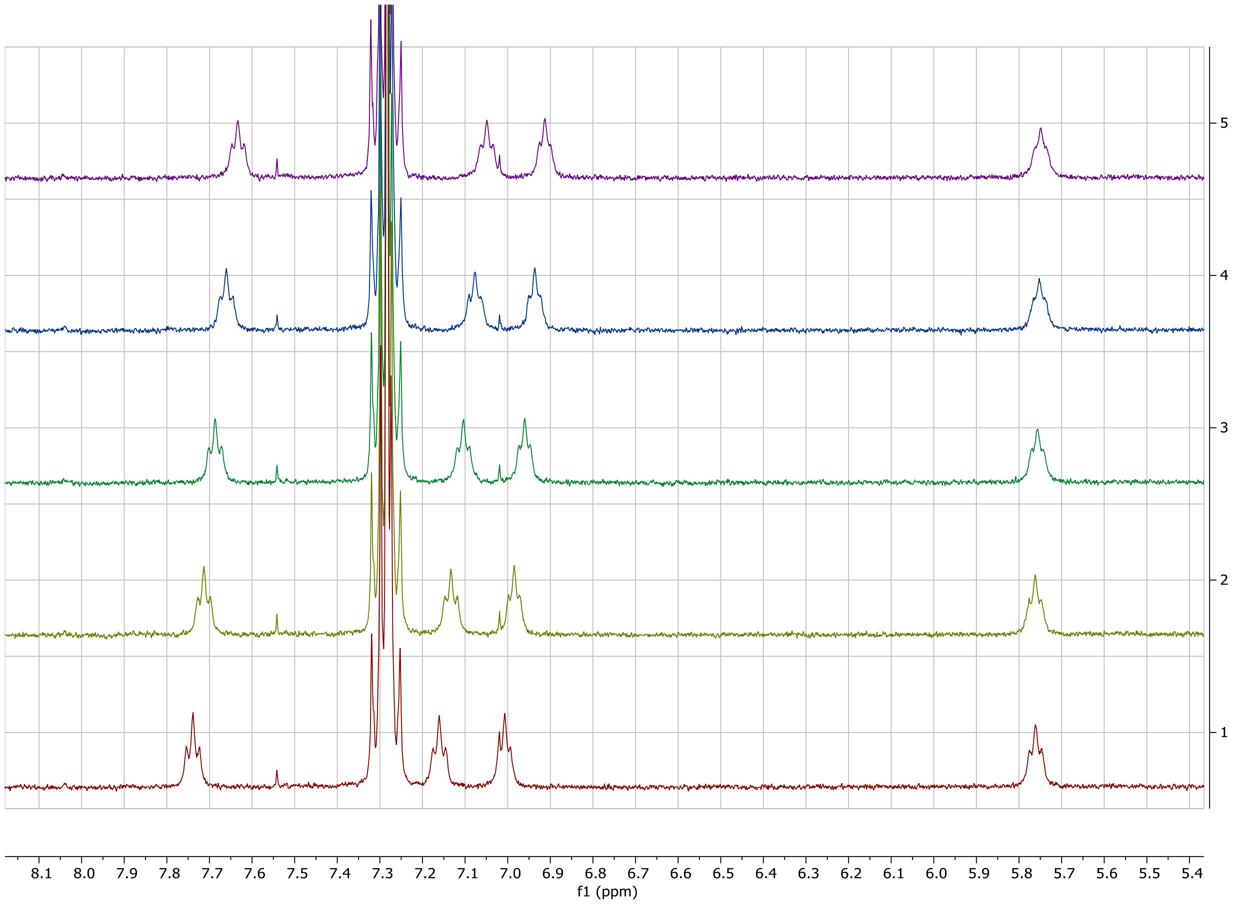

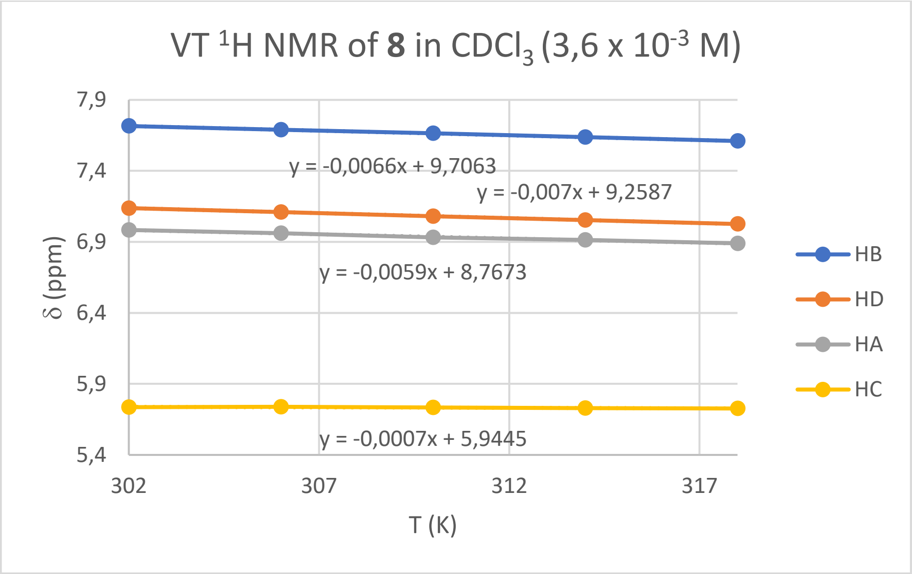

Figure S4. VT ^{1}H NMR spectra of **8** in dmso-d_6 (400 MHz)

From the bottom to the top: a) ^{1}H NMR in dmso-d_6 at 302 K, b) ^{1}H NMR in dmso-d_6 at 306 K, c) ^{1}H NMR in dmso-d_6 at 310 K, d) ^{1}H NMR in dmso-d_6 at 314 K, e) ^{1}H NMR in dmso-d_6 at 318 K.

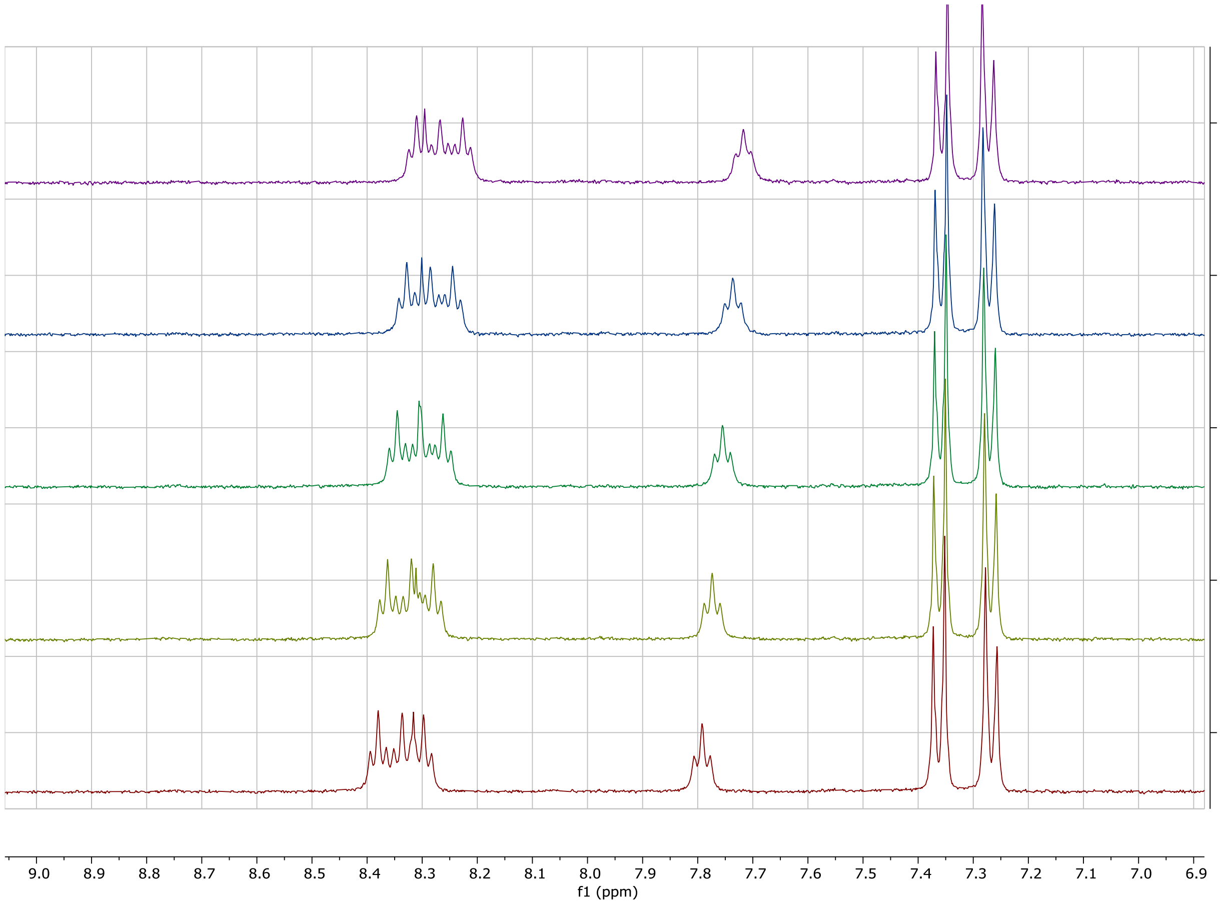

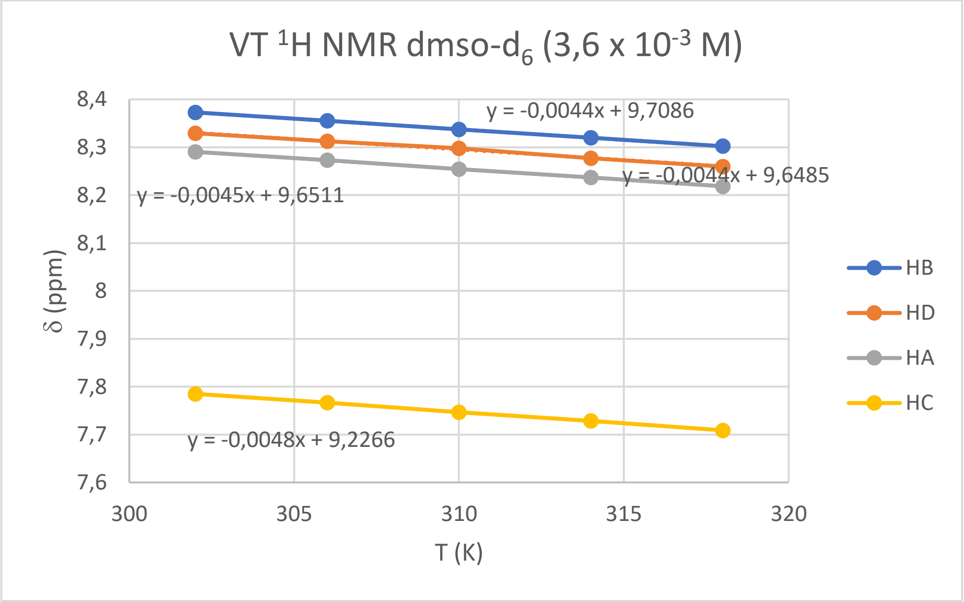

Figure S5. VT ^{1}H NMR spectra of **14** in $CDCl_3$ (400 MHz)

From the bottom to the top: a) ^{1}H NMR in $CDCl_3$ at 302 K, b) ^{1}H NMR in $CDCl_3$ at 306 K, c) ^{1}H NMR in $CDCl_3$ at 310 K, d) ^{1}H NMR in $CDCl_3$ at 314 K, e) ^{1}H NMR in $CDCl_3$ at 318 K.

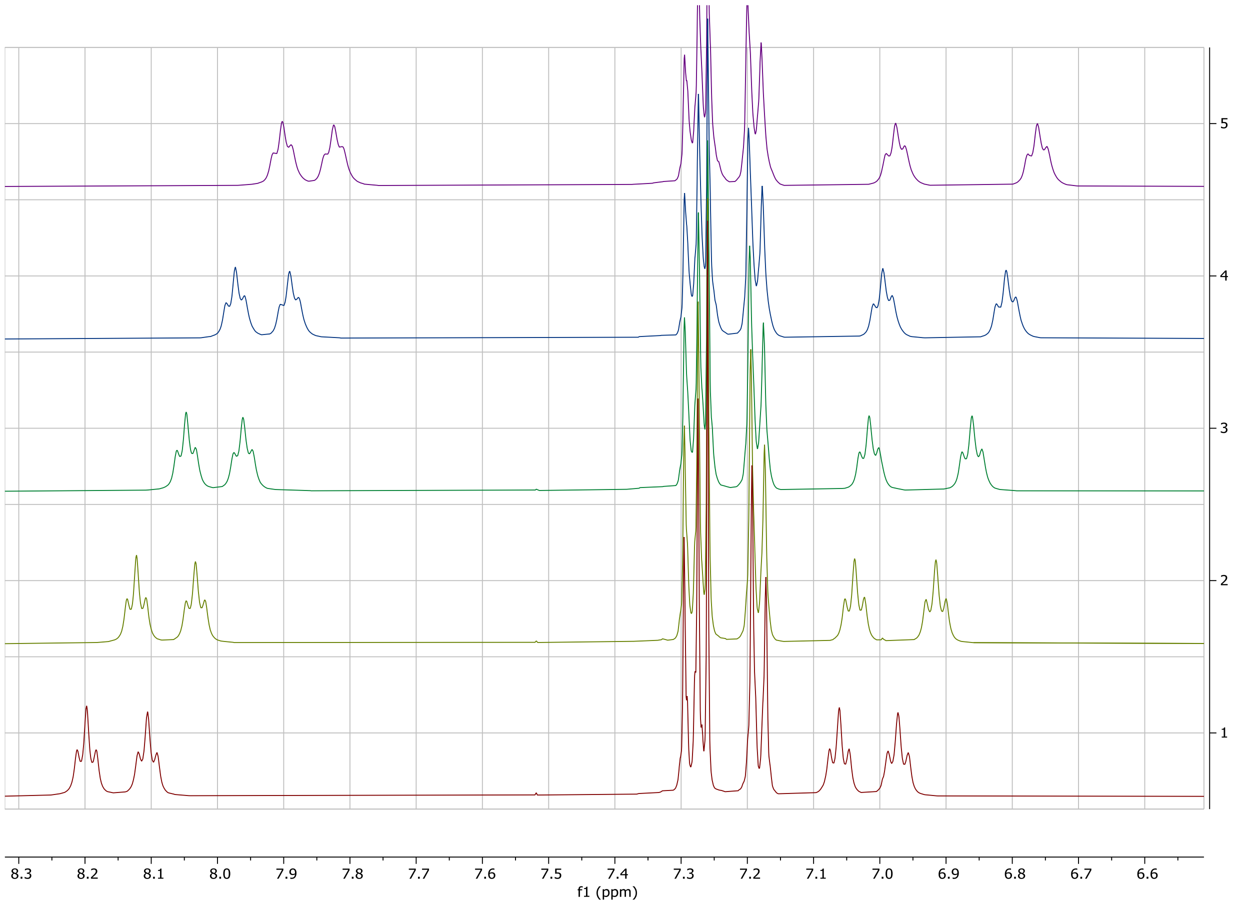

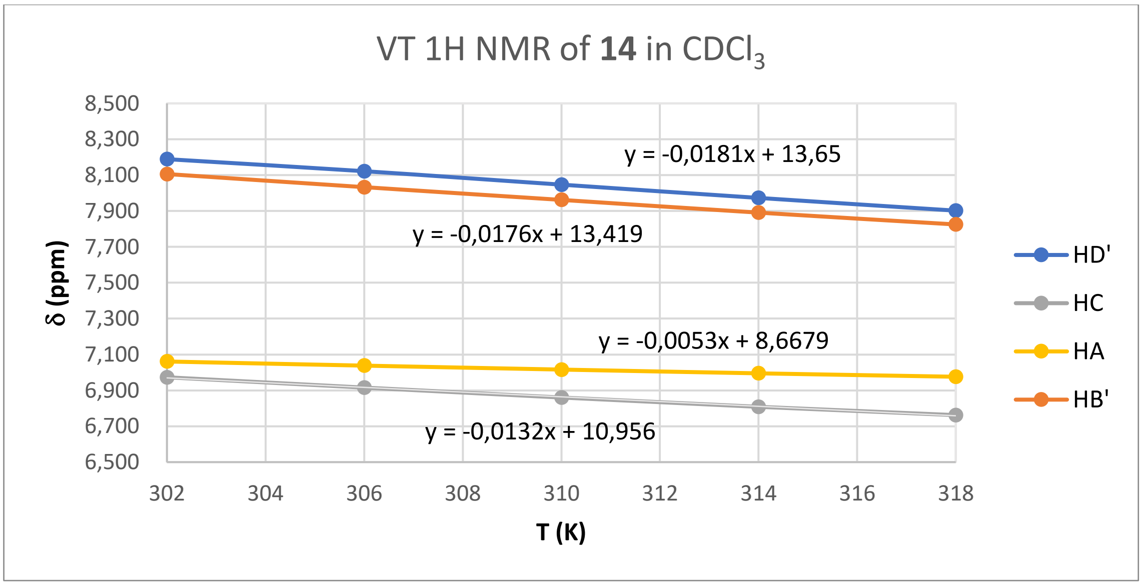

Table S1-S2. Computational results for compound **6a**

Energy : -1432.058360 hartrees; Solvation : -36.22 kJ/mol (SM54/AM1); ZPE: 1420.01 kJ/mol

Cartesian Coordinates (Angstroms)

Atom		X	Y	Z
1	N	-2.2602	0.370861	1.907461
2	C	-2.91586	0.668263	0.7462
3	N	-3.33039	-0.53866	0.140445
4	C	-2.79505	-1.61692	0.835666
5	C	-2.10463	-1.05673	2.089348
6	O	-3.10475	1.805142	0.322451
7	O	-2.85145	-2.80185	0.578852
8	C	-0.67431	-1.58548	2.254131
9	C	0.26232	-1.19275	1.113826
10	N	1.568309	-1.46458	1.327363
11	O	-0.17127	-0.68789	0.071462
12	H	-0.2546	-1.24319	3.206004
13	H	-0.74502	-2.6787	2.296501
14	H	1.837768	-1.79711	2.243059
15	C	2.644019	-1.0861	0.409692
16	C	4.748702	0.291356	0.120658
17	C	4.483113	-1.90593	-1.12273
18	C	5.471741	-0.93074	-0.46648
19	C	3.36522	-2.32084	-0.15472
20	C	3.631432	-0.12332	1.088809
21	H	4.315833	0.885894	-0.69572
22	H	4.031434	-1.4241	-2.00167
23	H	6.015406	-1.4508	0.335345
24	H	3.792258	-2.89955	0.677797
25	H	4.076752	-0.62645	1.961644
26	H	2.149466	-0.56247	-0.41331
27	H	5.460843	0.946782	0.634539
28	H	5.007666	-2.79469	-1.49139
29	H	6.225793	-0.6101	-1.19481
30	H	2.640612	-2.97278	-0.6554
31	H	3.085278	0.74506	1.470566
32	C	-3.99573	-0.54978	-1.213
33	C	-3.00411	0.025305	-2.24118
34	H	-2.08371	-0.56562	-2.24892
35	H	-2.75436	1.062219	-2.01117
36	H	-3.44951	-0.01237	-3.24024
37	C	-5.27572	0.303385	-1.13296
38	H	-5.97778	-0.12543	-0.41051
39	H	-5.7625	0.309268	-2.11312
40	H	-5.05468	1.33113	-0.84697
41	C	-4.38777	-1.97953	-1.619
42	H	-5.0469	-2.44604	-0.8837
43	H	-3.52385	-2.62844	-1.75677
44	H	-4.92858	-1.90894	-2.56794
45	C	-1.66774	1.410734	2.729856
46	H	-1.51719	1.024988	3.739447
47	H	-2.36903	2.249599	2.771053
48	C	-0.29639	1.891469	2.212589
49	O	0.748362	1.54298	2.760258
50	N	-0.36936	2.711354	1.131896
51	H	-1.28561	2.850721	0.715426
52	C	0.792924	3.343377	0.534983
53	H	1.655783	3.069464	1.148325
54	H	0.696764	4.432875	0.564125
55	C	1.098071	2.988738	-0.92807
56	O	1.707752	3.79006	-1.6304
57	N	0.691145	1.7726	-1.36531
58	H	0.28637	1.09156	-0.73094
59	C	0.983519	1.350528	-2.72469
60	H	2.06035	1.224799	-2.8885
61	H	0.62723	2.097182	-3.43956
62	H	0.479859	0.400375	-2.90971
63	H	-2.68404	-1.40018	2.959116

Energy : -1432.050563 hartrees; Solvation : -30.67 kJ/mol (SM54/AM1); ZPE: 1421.87 kJ/mol

Cartesian Coordinates (Angstroms)

Atom		X	Y	Z
1	N	1.077884	0.020703	-1.28245
2	C	1.525858	-1.25644	-1.13833
3	N	0.480551	-2.03506	-0.57603
4	C	-0.61717	-1.23264	-0.32803
5	C	-0.30136	0.176072	-0.85124
6	O	2.646882	-1.65313	-1.44021
7	O	-1.66303	-1.54037	0.223427
8	C	-0.51013	1.294665	0.198419
9	C	-1.90932	1.931908	0.166747
10	N	-2.94092	1.091344	0.441431
11	O	-2.04083	3.12511	-0.08848
12	H	-0.28641	0.907834	1.199154
13	H	0.187479	2.107616	-0.01113
14	H	-2.73135	0.104105	0.555266
15	C	-4.33735	1.531441	0.380843
16	C	-5.19348	-0.32495	-1.1552
17	C	-5.39792	-0.58761	1.361442
18	C	-6.02892	-0.90463	-0.00332
19	C	-5.15324	0.920922	1.53508
20	C	-4.97692	1.18572	-0.98094
21	H	-5.6803	-0.52479	-2.1171
22	H	-6.04012	-0.9519	2.17199
23	H	-6.14516	-1.98816	-0.12391
24	H	-4.651	1.125629	2.487252
25	H	-4.35032	1.590456	-1.78353
26	H	-4.29996	2.618079	0.488971
27	H	-4.22093	-0.8359	-1.18804
28	H	-4.44995	-1.13588	1.458801
29	H	-7.04095	-0.47589	-0.04226
30	H	-6.12171	1.437638	1.569559
31	H	-5.94535	1.701084	-1.04712
32	C	0.685162	-3.49437	-0.23315
33	C	1.787793	-3.58609	0.836823
34	H	1.482669	-3.06455	1.749348
35	H	2.725752	-3.15841	0.480903
36	H	1.961056	-4.63595	1.090787
37	C	1.090468	-4.23595	-1.52143
38	H	0.299997	-4.15773	-2.2749
39	H	1.233051	-5.29605	-1.29166
40	H	2.01752	-3.84381	-1.93876
41	C	-0.60799	-4.12349	0.308507
42	H	-1.43033	-4.05489	-0.40589
43	H	-0.93304	-3.67217	1.245583
44	H	-0.39803	-5.1823	0.487497
45	C	1.866221	1.032151	-1.97505
46	H	1.198847	1.816935	-2.33247
47	H	2.362294	0.561635	-2.83021
48	C	2.906771	1.696956	-1.05633
49	O	2.766486	2.858528	-0.6656
50	N	3.951429	0.906543	-0.72138
51	H	3.9078	-0.07403	-0.98628
52	C	5.022487	1.366206	0.158167
53	H	5.274934	2.393347	-0.12328
54	H	5.892431	0.727234	0.008542
55	C	4.672828	1.307159	1.65934
56	O	5.237916	0.524301	2.414979
57	N	3.712532	2.192758	2.037705
58	H	3.298142	2.772445	1.314083
59	C	3.228809	2.276599	3.403074
60	H	3.366552	3.285078	3.807556
61	H	3.802807	1.569279	4.00247
62	H	2.164838	2.019757	3.467724
63	H	-0.94818	0.366993	-1.7209

Table S3-S4. Computational results for compound **6f**

Energy : -1263.355560 hartrees; Solvation : -15.58 kJ/mol (SM54/AM1); ZPE: 1351.08 kJ/mol

Cartesian Coordinates (Angstroms)

**Atom**		**X**	**Y**	**Z**
1	N	1.856518	-1.21919	0.292473
2	C	2.959749	-0.44785	0.004143
3	N	2.555647	0.926691	-0.01964
4	C	1.194406	1.015867	0.13128
5	C	0.657072	-0.40335	0.324041
6	O	4.087886	-0.85271	-0.20519
7	O	0.484754	2.014451	0.169124
8	C	-0.14946	-0.48008	1.652429
9	C	-1.65737	-0.42114	1.411675
10	N	-2.1196	0.779404	1.002935
11	O	-2.36513	-1.42563	1.562328
12	H	0.067003	-1.41662	2.161609
13	H	0.159286	0.332185	2.318182
14	H	-1.42233	1.492209	0.801643
15	C	3.561828	2.033703	-0.2227
16	C	2.892651	3.414242	-0.13156
17	H	2.427159	3.585843	0.84051
18	H	2.134653	3.560645	-0.90116
19	C	4.187188	1.864252	-1.61963
20	H	4.676118	0.894898	-1.71765
21	H	3.422149	1.960945	-2.39672
22	C	4.630253	1.924094	0.881822
23	H	5.155397	0.97042	0.836544
24	H	4.173086	2.035269	1.870393
25	C	1.87128	-2.63581	-0.10372
26	H	2.90573	-2.96259	0.017593
27	H	1.610716	-2.73756	-1.16471
28	C	0.943338	-3.55938	0.698097
29	H	1.073158	-3.404	1.773059
30	H	1.253117	-4.58298	0.466038
31	N	-0.46847	-3.43496	0.363839
32	H	-1.10913	-2.94067	0.980598
33	C	-0.96448	-4.04336	-0.75701
34	O	-0.24445	-4.65775	-1.53967
35	C	-2.46637	-3.91693	-0.95466
36	H	-2.93275	-4.8841	-0.74073
37	H	-2.66205	-3.68713	-2.00462
38	H	-2.91906	-3.15669	-0.31381
39	C	-3.51978	1.01523	0.632549
40	C	-3.25899	3.495174	0.04543
41	C	-3.09701	1.743904	-1.78431
42	C	-3.53177	3.175035	-1.43283
43	C	-3.77057	0.717	-0.86163
44	C	-3.91763	2.46158	0.975612
45	H	-3.62583	4.497751	0.294034
46	H	-3.33639	1.518248	-2.82989
47	H	-3.01847	3.898506	-2.07676
48	H	-3.43306	-0.29989	-1.08894
49	H	-3.67737	2.670458	2.024265
50	H	-4.10449	0.31489	1.234935
51	H	-2.17335	3.521093	0.214108
52	H	-2.00502	1.664194	-1.69314
53	H	-4.60715	3.283672	-1.63368
54	H	-4.85583	0.732824	-1.03134
55	H	-5.00824	2.53925	0.879023
56	H	4.931872	2.65006	-1.77924
57	H	3.678427	4.162671	-0.27327
58	H	5.358956	2.730036	0.754244
59	H	0.004528	-0.64751	-0.5252

Energy : -1263.351845 hartrees; Solvation : -25.02 kJ/mol (SM54/AM1); ZPE: 1347.44 kJ/mol

Cartesian Coordinates (Angstroms)

Atom		X	Y	Z
1	N	1.856518	-1.21919	0.292473
2	C	2.959749	-0.44785	0.004143
3	N	2.555647	0.926691	-0.01964
4	C	1.194406	1.015867	0.13128
5	C	0.657072	-0.40335	0.324041
6	O	4.087886	-0.85271	-0.20519
7	O	0.484754	2.014451	0.169124
8	C	-0.14946	-0.48008	1.652429
9	C	-1.65737	-0.42114	1.411675
10	N	-2.1196	0.779404	1.002935
11	O	-2.36513	-1.42563	1.562328
12	H	0.067003	-1.41662	2.161609
13	H	0.159286	0.332185	2.318182
14	H	-1.42233	1.492209	0.801643
15	C	3.561828	2.033703	-0.2227
16	C	2.892651	3.414242	-0.13156
17	H	2.427159	3.585843	0.84051
18	H	2.134653	3.560645	-0.90116
19	C	4.187188	1.864252	-1.61963
20	H	4.676118	0.894898	-1.71765
21	H	3.422149	1.960945	-2.39672
22	C	4.630253	1.924094	0.881822
23	H	5.155397	0.97042	0.836544
24	H	4.173086	2.035269	1.870393
25	C	1.87128	-2.63581	-0.10372
26	H	2.90573	-2.96259	0.017593
27	H	1.610716	-2.73756	-1.16471
28	C	0.943338	-3.55938	0.698097
29	H	1.073158	-3.404	1.773059
30	H	1.253117	-4.58298	0.466038
31	N	-0.46847	-3.43496	0.363839
32	H	-1.10913	-2.94067	0.980598
33	C	-0.96448	-4.04336	-0.75701
34	O	-0.24445	-4.65775	-1.53967
35	C	-2.46637	-3.91693	-0.95466
36	H	-2.93275	-4.8841	-0.74073
37	H	-2.66205	-3.68713	-2.00462
38	H	-2.91906	-3.15669	-0.31381
39	C	-3.51978	1.01523	0.632549
40	C	-3.25899	3.495174	0.04543
41	C	-3.09701	1.743904	-1.78431
42	C	-3.53177	3.175035	-1.43283
43	C	-3.77057	0.717	-0.86163
44	C	-3.91763	2.46158	0.975612
45	H	-3.62583	4.497751	0.294034
46	H	-3.33639	1.518248	-2.82989
47	H	-3.01847	3.898506	-2.07676
48	H	-3.43306	-0.29989	-1.08894
49	H	-3.67737	2.670458	2.024265
50	H	-4.10449	0.31489	1.234935
51	H	-2.17335	3.521093	0.214108
52	H	-2.00502	1.664194	-1.69314
53	H	-4.60715	3.283672	-1.63368
54	H	-4.85583	0.732824	-1.03134
55	H	-5.00824	2.53925	0.879023
56	H	4.931872	2.65006	-1.77924
57	H	3.678427	4.162671	-0.27327
58	H	5.358956	2.730036	0.754244
59	H	0.004528	-0.64751	-0.5252

Table S5-S6. Computational results for compound **6g**

Energy : -1720.094152 hartrees; Solvation : -18.37 kJ/mol (SM54/AM1); ZPE: 1587.68 kJ/mol

Cartesian Coordinates (Angstroms)

Atom		X	Y	Z
1	N	-1.66452	1.389682	1.210808
2	C	-2.93635	1.501194	0.728423
3	N	-3.41099	0.182545	0.427254
4	C	-2.41204	-0.73459	0.643803
5	C	-1.1788	0.019347	1.149978
6	O	-3.56695	2.533943	0.574503
7	O	-2.45345	-1.95076	0.484958
8	C	-0.65591	-0.52583	2.505428
9	C	0.436718	-1.58959	2.354052
10	N	0.075073	-2.70411	1.656599
11	O	1.551267	-1.43068	2.842034
12	H	-0.1987	0.289566	3.066971
13	H	-1.48855	-0.9171	3.100632
14	H	-0.83014	-2.69567	1.187943
15	C	1.068626	-3.66389	1.243573
16	C	1.581852	-3.5078	-0.18698
17	H	0.698071	-4.68878	1.337578
18	C	-4.83392	-0.04857	-0.01944
19	C	-5.05167	0.695494	-1.35028
20	H	-4.414	0.273794	-2.13436
21	H	-4.83972	1.75966	-1.24688
22	C	-5.77198	0.493883	1.075986
23	H	-5.60528	-0.03614	2.019327
24	H	-5.6204	1.561443	1.235491
25	C	-5.12068	-1.5447	-0.22554
26	H	-4.98872	-2.12253	0.690291
27	H	-4.49011	-1.98812	-0.99691
28	C	-0.84306	2.575713	1.430839
29	H	-0.14671	2.384992	2.250905
30	H	-1.52201	3.374827	1.737459
31	H	1.930043	-3.55678	1.909713
32	O	2.237447	-4.35283	-0.75431
33	O	1.236255	-2.32245	-0.73437
34	C	1.683736	-2.10056	-2.09294
35	H	1.464153	-2.99943	-2.67833
36	H	2.768564	-1.96279	-2.0946
37	C	0.973113	-0.88593	-2.62986
38	C	-0.35044	1.363023	-3.66979
39	C	1.695497	0.143604	-3.2421
40	C	-0.4221	-0.77775	-2.53957
41	C	-1.07756	0.342512	-3.04995
42	C	1.038272	1.260237	-3.76652
43	H	2.777912	0.07685	-3.30118
44	H	-0.98823	-1.56477	-2.05011
45	H	-2.15759	0.419544	-2.96835
46	H	1.612419	2.050622	-4.24062
47	H	-0.86354	2.231405	-4.072
48	C	-0.04774	3.031532	0.197547
49	H	0.382715	4.014198	0.420679
50	H	-0.72089	3.136482	-0.65725
51	N	1.014124	2.107547	-0.17036
52	H	0.994005	1.638801	-1.06484
53	C	2.198343	2.123869	0.51975
54	O	2.385077	2.81398	1.511324
55	O	3.077952	1.272788	-0.05226
56	C	4.410291	1.052603	0.544228
57	C	4.996467	-0.03393	-0.35995
58	H	6.006718	-0.29382	-0.03093
59	H	5.050031	0.309653	-1.39782
60	H	4.378831	-0.93579	-0.31914
61	C	4.281852	0.545923	1.983556
62	H	3.910285	1.329951	2.642665
63	H	5.266784	0.224599	2.338699
64	H	3.598567	-0.30559	2.040633
65	C	5.228026	2.344091	0.452207
66	H	4.784975	3.125715	1.070392
67	H	5.278748	2.693471	-0.5842
68	H	6.249596	2.158825	0.799856
69	H	-6.81077	0.328151	0.774828
70	H	-6.09233	0.574589	-1.66604
71	H	-6.16556	-1.6336	-0.5392
72	H	-0.38894	-0.07703	0.394805

Energy : -1720.092838 hartrees; Solvation : -24.31 kJ/mol (SM54/AM1); ZPE: 1589.35 kJ/mol

Cartesian Coordinates (Angstroms)

Atom		X	Y	Z
1	N	-1.55376	0.92858	1.56382
2	C	-2.84068	1.238518	1.179987
3	N	-3.4422	0.066335	0.623182
4	C	-2.51051	-0.94004	0.538382
5	C	-1.20557	-0.4115	1.126907
6	O	-3.3794	2.325083	1.28353
7	O	-2.64433	-2.08678	0.128295
8	C	-0.71718	-1.36995	2.256191
9	C	0.422841	-2.24299	1.751398
10	N	0.075762	-3.16679	0.815673
11	O	1.585259	-2.07233	2.124223
12	H	-0.34618	-0.79652	3.104046
13	H	-1.54787	-1.98939	2.608269
14	H	-0.86438	-3.08825	0.428721
15	C	1.11497	-3.76173	0.002516
16	C	1.763045	-2.81516	-1.01422
17	H	0.70293	-4.61288	-0.54773
18	C	-4.88328	0.060301	0.180104
19	C	-5.30349	-1.33502	-0.30927
20	H	-5.20067	-2.09297	0.469111
21	H	-4.72877	-1.66242	-1.17579
22	C	-5.03513	1.069864	-0.97283
23	H	-4.78005	2.080333	-0.65168
24	H	-4.38863	0.790847	-1.81075
25	C	-5.76141	0.450735	1.383939
26	H	-5.51671	1.447813	1.749742
27	H	-5.63641	-0.27013	2.198608
28	C	-0.58441	2.030394	1.657021
29	H	-1.1337	2.869509	2.088614
30	H	-0.25578	2.330831	0.653839
31	H	1.920133	-4.13147	0.639074
32	O	2.812998	-3.05485	-1.56657
33	O	1.007466	-1.72317	-1.24507
34	C	1.551462	-0.75498	-2.188
35	H	1.934918	-1.3032	-3.05301
36	H	2.385509	-0.24763	-1.7001
37	C	0.454258	0.20569	-2.55826
38	C	-1.59747	1.986037	-3.26158
39	C	0.519632	1.550663	-2.17321
40	C	-0.65065	-0.23874	-3.29768
41	C	-1.67098	0.644076	-3.64637
42	C	-0.50112	2.437157	-2.52662
43	H	1.365559	1.911127	-1.5938
44	H	-0.7086	-1.28129	-3.59894
45	H	-2.5192	0.288955	-4.2246
46	H	-0.43675	3.478463	-2.22555
47	H	-2.39006	2.675429	-3.53684
48	C	0.663358	1.754408	2.506521
49	H	0.386542	1.313596	3.468494
50	H	1.12121	2.727624	2.706341
51	N	1.669963	0.912149	1.87744
52	H	1.790127	-0.06388	2.143381
53	C	2.614869	1.463707	1.063662
54	O	2.644836	2.647175	0.746351
55	O	3.48117	0.503953	0.647444
56	C	4.829036	0.855335	0.163494
57	C	5.484764	-0.51609	-0.01572
58	H	6.499988	-0.3957	-0.40563
59	H	4.913484	-1.13501	-0.7134
60	H	5.537997	-1.04426	0.940247
61	C	5.554125	1.676039	1.234669
62	H	5.07536	2.646874	1.374921
63	H	6.593084	1.840512	0.932499
64	H	5.558538	1.138766	2.188114
65	C	4.75732	1.607233	-1.16977
66	H	4.242582	1.008913	-1.9278
67	H	5.772476	1.79653	-1.53389
68	H	4.23859	2.558346	-1.05245
69	H	-6.07142	1.067122	-1.32532
70	H	-6.35846	-1.27449	-0.59308
71	H	-6.81269	0.436921	1.080707
72	H	-0.46173	-0.37726	0.321658

Table S7-S8. Computational results for compound **8**

Energy : -2485.585544 hartrees; ZPE: 1954.66 kJ/mol

Cartesian Coordinates (Angstroms)

Atom		X	Y	Z
1	N	-1.33325	-2.32931	-0.11632
2	C	-2.64391	-1.99505	0.127916
3	N	-2.91655	-0.76962	-0.54911
4	C	-1.7874	-0.33191	-1.21319
5	C	-0.64875	-1.30442	-0.87321
6	O	-3.43631	-2.648	0.781788
7	O	-1.6406	0.627404	-1.94892
8	C	0.079537	-1.77232	-2.15846
9	C	1.508823	-2.20519	-1.86043
10	N	2.44777	-1.26683	-2.09905
11	O	1.777313	-3.30802	-1.36526
12	H	-0.46335	-2.61156	-2.60073
13	H	0.060029	-0.93171	-2.85898
14	H	2.221826	-0.38335	-2.54284
15	C	3.82831	-1.38627	-1.67469
16	C	4.388308	0.033278	-1.54767
17	H	4.431334	-1.92303	-2.42073
18	C	-0.67677	-3.40236	0.601729
19	H	-0.18017	-4.07182	-0.10382
20	H	-1.46104	-3.94642	1.134913
21	H	3.888951	-1.94476	-0.73941
22	O	3.968835	0.928994	-2.28469
23	N	5.360691	0.199565	-0.62331
24	C	2.825982	0.844066	2.248441
25	H	3.276777	1.239589	3.167936
26	C	1.633373	1.689048	1.857254
27	C	-0.60853	3.201346	1.160139
28	C	0.831049	2.274549	2.843345
29	C	1.291438	1.880591	0.511993
30	C	0.170131	2.630224	0.154119
31	C	-0.29006	3.031525	2.506008
32	H	1.084584	2.142036	3.892006
33	H	1.914595	1.459302	-0.27263
34	H	-0.10164	2.757832	-0.88702
35	H	-0.90627	3.485426	3.273863
36	C	0.32187	-2.87233	1.641424
37	N	1.584207	-3.35516	1.504858
38	H	1.845127	-3.71143	0.588419
39	C	2.64892	-2.97993	2.425344
40	H	0.055641	-0.74706	-0.23659
41	H	5.425757	-0.49169	0.117441
42	C	3.25641	-1.59388	2.138233
43	N	2.449155	-0.55683	2.451276
44	Cl	-2.01371	4.168343	0.724575
45	H	2.242803	-3.00249	3.440868
46	H	3.457285	-3.70737	2.344936
47	O	-0.01617	-2.09055	2.533185
48	O	4.389584	-1.48072	1.656488
49	H	3.596115	0.865186	1.474888
50	C	6.028908	1.489783	-0.45193
51	H	6.357862	1.554034	0.591261
52	H	5.293683	2.281192	-0.62932
53	C	7.23024	1.683467	-1.39569
54	H	6.848812	1.565267	-2.41851
55	C	8.3174	0.630019	-1.15314
56	H	7.91751	-0.38407	-1.24902
57	H	8.744422	0.731111	-0.14723
58	H	9.135908	0.737826	-1.87208
59	C	7.7842	3.106702	-1.24987
60	H	8.153434	3.284583	-0.23209
61	H	7.019483	3.860427	-1.46597
62	H	8.62077	3.273057	-1.93603
63	H	1.50865	-0.78308	2.773479
64	C	-4.29337	-0.16625	-0.54659
65	C	-5.75194	1.761113	-1.27824
66	C	-6.70252	-0.56275	-1.20381
67	C	-6.72357	0.779122	-1.96005
68	C	-5.27496	-1.14691	-1.23348
69	C	-4.3213	1.180845	-1.30485
70	H	-6.43309	0.626452	-3.00762
71	H	-4.95125	-1.3075	-2.27002
72	H	-3.99723	1.043729	-2.33903
73	H	-5.74454	2.712776	-1.82378
74	H	-7.38555	-1.27316	-1.68605
75	H	-7.73904	1.195761	-1.96885
76	H	-5.24503	-2.11209	-0.72151
77	H	-3.62212	1.886449	-0.84731
78	C	-4.73762	0.08676	0.917018
79	H	-4.03914	0.793469	1.382671
80	H	-4.69638	-0.8453	1.483386
81	C	-6.19684	1.999024	0.176787
82	H	-5.52715	2.720235	0.662266
83	H	-7.20634	2.42962	0.198412
84	C	-7.13285	-0.33908	0.260119
85	H	-7.13153	-1.29157	0.805146
86	H	-8.15988	0.047064	0.296675
87	C	-6.16965	0.659651	0.934426
88	H	-6.47208	0.815045	1.977529

Energy : -2485.579270 hartrees; ZPE: 1951.02 kJ/mol

Cartesian Coordinates (Angstroms)

Atom		X	Y	Z
1	N	-1.05035	-0.39333	-2.2865
2	C	-1.04308	-1.6495	-1.751
3	N	0.093262	-1.75736	-0.90606
4	C	0.806221	-0.57154	-0.93342
5	C	0.095717	0.394188	-1.87692
6	O	-1.88564	-2.50829	-1.98422
7	O	1.808757	-0.30278	-0.28814
8	C	-0.24342	1.723878	-1.17529
9	C	0.992191	2.624873	-1.19057
10	N	1.424058	3.080374	0.016337
11	O	1.54225	2.900259	-2.25298
12	H	-1.02269	2.230318	-1.75456
13	H	-0.63928	1.542717	-0.17169
14	H	0.989408	2.733128	0.859394
15	C	2.616598	3.901624	0.138062
16	C	3.968991	3.17362	0.245616
17	H	2.524616	4.547178	1.014957
18	C	-1.99243	-0.01647	-3.33167
19	H	-2.11058	-0.85832	-4.02202
20	H	-1.58964	0.839494	-3.87378
21	H	2.681762	4.541858	-0.74434
22	O	4.986726	3.843585	0.39357
23	N	3.948183	1.822016	0.167513
24	C	-5.03372	2.234537	1.030753
25	H	-5.35975	3.262478	0.845416
26	C	-3.65883	2.236794	1.675014
27	C	-1.11663	2.209657	2.827904
28	C	-2.88045	3.396979	1.712292
29	C	-3.14396	1.059618	2.239373
30	C	-1.87462	1.037307	2.814005
31	C	-1.6076	3.395495	2.287713
32	H	-3.26703	4.320179	1.288448
33	H	-3.74951	0.158198	2.223541
34	H	-1.4792	0.128144	3.253368
35	H	-1.011	4.300537	2.318475
36	C	-3.36209	0.399907	-2.76878
37	N	-4.08795	-0.61328	-2.24482
38	H	-3.63445	-1.52001	-2.15343
39	C	-5.42545	-0.41331	-1.69749
40	H	0.747059	0.624297	-2.73126
41	H	3.079402	1.332316	-0.00719
42	C	-5.43441	0.186127	-0.27737
43	N	-5.09261	1.501494	-0.229
44	Cl	0.502514	2.188117	3.532871
45	H	-5.98136	0.239863	-2.37765
46	H	-5.92296	-1.38113	-1.63905
47	O	-3.74254	1.573908	-2.80789
48	O	-5.74458	-0.48304	0.704207
49	H	-5.75765	1.763666	1.700951
50	C	5.181541	1.049053	0.119426
51	H	4.951206	0.037538	0.47508
52	H	5.888936	1.503969	0.818748
53	C	5.822492	0.979877	-1.28207
54	H	6.068055	2.011141	-1.5657
55	C	4.856643	0.407162	-2.32727
56	H	3.957986	1.024686	-2.41574
57	H	4.546853	-0.61123	-2.05919
58	H	5.330135	0.362562	-3.31398
59	C	7.125861	0.173779	-1.21361
60	H	6.93173	-0.86668	-0.92323
61	H	7.82249	0.599552	-0.48312
62	H	7.629502	0.156546	-2.18561
63	H	-4.72713	1.919822	-1.08155
64	C	0.445965	-2.90056	0.011574
65	C	2.276658	-4.52415	0.649456
66	C	0.80334	-3.52991	2.436313
67	C	2.234276	-3.99563	2.097662
68	C	0.394276	-2.3958	1.475597
69	C	1.875047	-3.39567	-0.32366
70	H	2.939666	-3.16358	2.216746
71	H	1.063225	-1.54061	1.597827
72	H	2.580987	-2.565	-0.25152
73	H	3.294902	-4.84992	0.404977
74	H	0.772898	-3.14761	3.463941
75	H	2.548868	-4.78285	2.794526
76	H	-0.62585	-2.05771	1.699483
77	H	1.893189	-3.76137	-1.35781
78	C	-0.53389	-4.08759	-0.13568
79	H	-0.53222	-4.45432	-1.16551
80	H	-1.55515	-3.76591	0.081343
81	C	1.298038	-5.70325	0.500469
82	H	1.333792	-6.0979	-0.52321
83	H	1.586435	-6.52499	1.168488
84	C	-0.17648	-4.70752	2.284097
85	H	-1.19535	-4.38459	2.53435
86	H	0.083734	-5.51386	2.981385
87	C	-0.12591	-5.21918	0.832009
88	H	-0.83599	-6.04552	0.706853

Table S9-S10. Computational results for compound **14**

Energy : -2174.680559 hartrees; ZPE: 1514.23 kJ/mol

Cartesian Coordinates (Angstroms)

Atom		X	Y	Z
1	N	1.555394	-0.71616	-0.29223
2	C	2.716127	-0.6229	-1.01049
3	N	3.808191	-0.69803	-0.10136
4	C	3.338083	-0.85402	1.192764
5	C	1.804738	-0.73966	1.137964
6	O	2.791049	-0.5077	-2.22398
7	O	3.961371	-1.04299	2.21851
8	C	1.117266	-1.86892	1.934349
9	C	-0.20215	-1.41648	2.546209
10	N	-1.32941	-1.967	2.041954
11	O	-0.21134	-0.56964	3.451324
12	H	1.007397	-2.75874	1.308057
13	H	1.778156	-2.10512	2.77452
14	H	-1.29703	-2.57145	1.218283
15	C	-2.65628	-1.64898	2.559148
16	C	-3.19165	-0.28128	2.087501
17	H	-2.62605	-1.66746	3.653607
18	C	5.233446	-0.68207	-0.58963
19	C	6.220432	-0.75498	0.586721
20	H	6.119028	-1.67634	1.160115
21	H	6.102084	0.08348	1.275323
22	C	5.464941	0.636362	-1.35246
23	H	4.79035	0.726255	-2.20368
24	H	5.316056	1.493434	-0.68807
25	C	5.446583	-1.8997	-1.50873
26	H	4.779425	-1.86557	-2.37103
27	H	5.272452	-2.8303	-0.95878
28	C	0.257004	-0.55886	-0.93636
29	H	0.224752	0.387555	-1.48701
30	H	-0.49582	-0.50828	-0.1482
31	H	-3.34732	-2.41322	2.204826
32	O	-4.14141	-0.18423	1.30053
33	N	-2.53959	0.770543	2.627446
34	C	-2.77055	2.155475	2.228047
35	H	-2.82051	2.768251	3.135165
36	H	-3.74719	2.192448	1.743142
37	C	-1.69012	2.687141	1.301206
38	C	0.32027	3.624312	-0.4041
39	C	-1.94585	2.899744	-0.05706
40	C	-0.40365	2.956015	1.789352
41	C	0.604771	3.421597	0.947219
42	C	-0.94994	3.368422	-0.91586
43	H	-2.93728	2.704225	-0.45428
44	H	-0.18011	2.800835	2.84184
45	H	1.595613	3.634834	1.33302
46	H	-1.15696	3.536276	-1.96695
47	C	-0.06753	-1.7406	-1.87969
48	H	0.275553	-2.66825	-1.41665
49	H	0.460554	-1.61365	-2.82577
50	N	-1.49267	-1.88054	-2.13341
51	H	-1.95797	-1.3369	-2.85892
52	C	-2.27969	-2.63139	-1.33233
53	O	-1.8534	-3.29196	-0.37357
54	H	6.496245	0.667509	-1.71772
55	H	7.229534	-0.7147	0.165083
56	H	6.480621	-1.90927	-1.86728
57	H	1.542175	0.22019	1.608479
58	H	-1.71004	0.53988	3.178749
59	C	-4.27883	-0.2829	-2.29099
60	O	-3.70606	-0.4182	-3.37769
61	C	-3.77607	-2.59345	-1.66624
62	H	-3.93159	-2.81669	-2.72502
63	H	-4.26432	-3.35512	-1.05723
64	N	-4.37214	-1.29543	-1.38533
65	H	-4.55982	-1.06188	-0.41113
66	C	-4.97566	1.004467	-1.89702
67	H	-4.46201	1.843671	-2.36893
68	H	-5.00786	1.132633	-0.81226
69	H	-6.00416	0.986167	-2.27317
70	Cl	1.581882	4.218551	-1.47445

Energy : -2174.6655201 hartrees; ZPE: 1510.80 kJ/mol

Cartesian Coordinates (Angstroms)

Atom		X	Y	Z
1	N	-0.76056	-0.17313	0.638546
2	C	-1.4954	0.345538	-0.38671
3	N	-0.76783	0.128814	-1.59946
4	C	0.315906	-0.67725	-1.34044
5	C	0.490324	-0.75183	0.17748
6	O	-2.5844	0.890677	-0.29228
7	O	1.021928	-1.26125	-2.15292
8	C	0.798481	-2.18314	0.681936
9	C	2.302062	-2.45964	0.720284
10	N	2.922059	-2.58461	-0.4772
11	O	2.899555	-2.5311	1.80096
12	H	0.474603	-2.27392	1.717289
13	H	0.250332	-2.91882	0.087747
14	H	2.395068	-2.31174	-1.30638
15	C	4.37382	-2.70419	-0.57438
16	C	5.05055	-1.32659	-0.45381
17	H	4.631304	-3.11858	-1.54917
18	C	-1.30057	0.446749	-2.9727
19	C	-0.12834	0.612106	-3.96012
20	H	0.417698	-0.31373	-4.12318
21	H	0.575261	1.370642	-3.60309
22	C	-2.066	1.783961	-2.95066
23	H	-2.97413	1.741364	-2.35482
24	H	-1.43627	2.584993	-2.55197
25	C	-2.21357	-0.7114	-3.40829
26	H	-3.04108	-0.84397	-2.70523
27	H	-1.6543	-1.64987	-3.45514
28	C	-1.14921	-0.02591	2.034296
29	H	-1.72146	0.901866	2.118393
30	H	-0.23485	0.087815	2.62847
31	H	4.717871	-3.37727	0.215493
32	O	5.514329	-0.73978	-1.42658
33	N	5.036912	-0.81828	0.812577
34	C	5.350653	0.585452	1.066501
35	H	6.104906	0.8787	0.332907
36	H	5.792159	0.669178	2.064016
37	C	4.126379	1.478457	0.955664
38	C	1.793872	3.003757	0.727943
39	C	3.474324	1.962961	2.094323
40	C	3.59191	1.784333	-0.30604
41	C	2.429946	2.544696	-0.42666
42	C	2.3088	2.725796	1.991996
43	H	3.874826	1.740806	3.080258
44	H	4.090079	1.410221	-1.19549
45	H	2.019926	2.779563	-1.4028
46	H	1.807677	3.098417	2.878566
47	C	-1.97822	-1.19636	2.603822
48	H	-2.10976	-1.02391	3.677533
49	H	-1.44228	-2.13992	2.483017
50	N	-3.28956	-1.34398	2.00422
51	H	-4.07172	-0.80051	2.364372
52	C	-3.49177	-2.01253	0.838384
53	O	-2.62459	-2.6443	0.238084
54	H	-2.3248	2.039788	-3.98225
55	H	-0.53614	0.958948	-4.91398
56	H	-2.63142	-0.50905	-4.3995
57	H	1.328257	-0.09031	0.448798
58	H	4.485839	-1.32922	1.499715
59	C	-5.65589	0.38555	0.833895
60	O	-5.78565	0.118656	2.03156
61	C	-4.9361	-1.91347	0.311913
62	H	-5.01562	-2.57744	-0.54957
63	H	-5.638	-2.23969	1.084351
64	N	-5.31607	-0.56257	-0.08756
65	H	-4.95526	-0.22803	-0.96997
66	C	-5.84223	1.787494	0.292919
67	H	-6.52087	2.332124	0.950026
68	H	-6.23012	1.794564	-0.72938
69	H	-4.8687	2.289195	0.295685
70	Cl	0.308157	3.939616	0.583976

Crystal structure of 6a

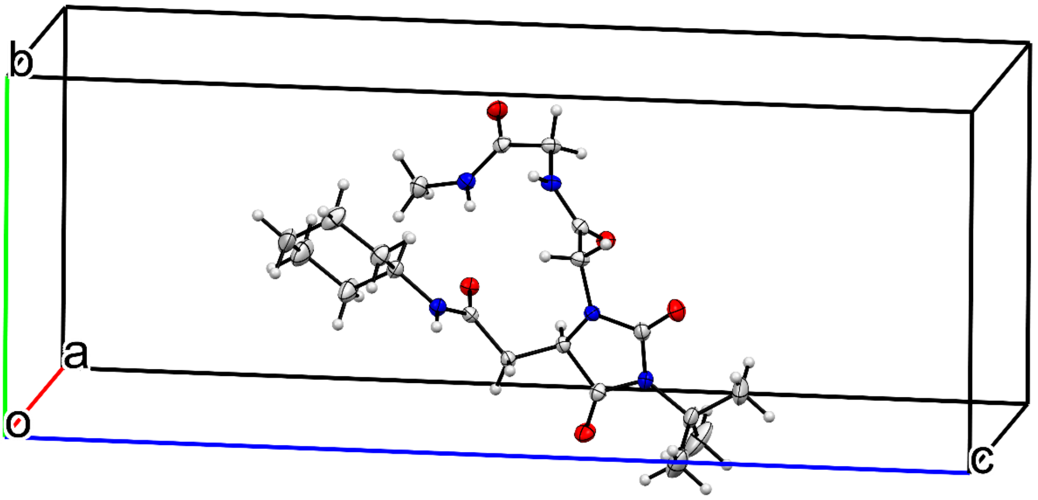

Figure S6. Crystal structure of **6a** showing only the asymmetric unit. ORTEP plot with thermal ellipsoids drawn at the 50 % of probability level.

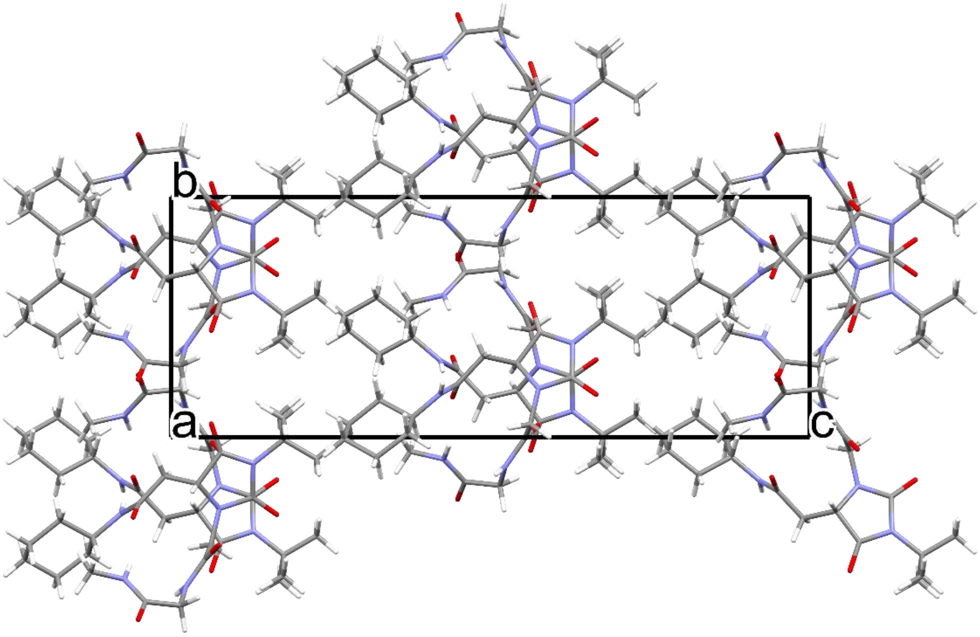

Figure S7. Crystal packing of **6a** viewed along the *a*-axis.

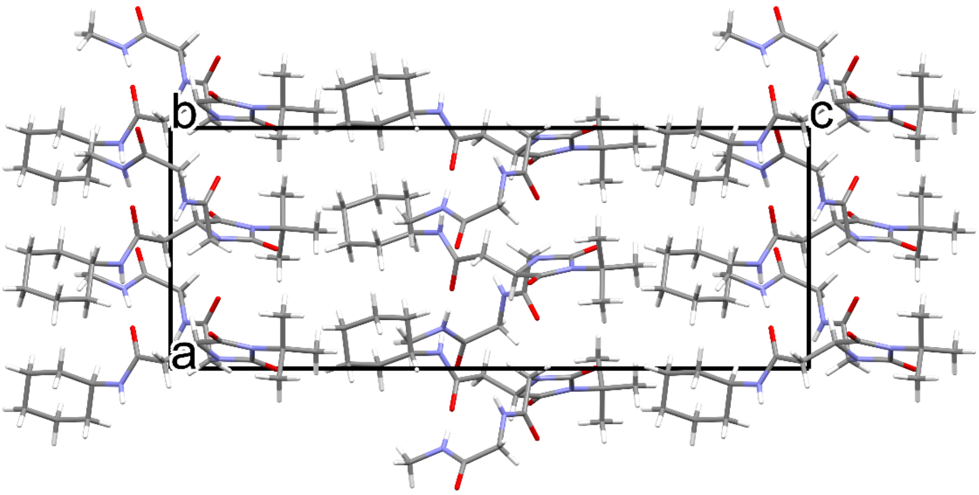

Figure S8. Crystal packing of **6a** viewed along the *b*-axis.

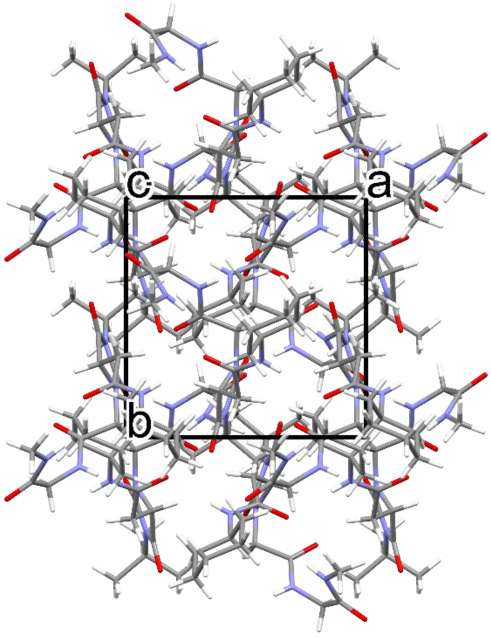

Figure S9. Crystal packing of **6a** viewed along the *c*-axis.

Table S11. Crystal data and structure refinement for **6a**.

Identification code	6a	
Empirical formula	$C_{20}H_{33}N_5O_5$	
Formula weight	423.51	
Temperature	100(2) K	
Wavelength	0.82656 Å	
Crystal system	Orthorhombic	
Space group	$Pna2_1$	
Unit cell dimensions	a = 9.42670(10) Å	α = 90°.
	b = 9.412 Å	β = 90°.
	c = 25.02480(10) Å	γ = 90°.
Volume	2220.30(3) $Å^3$	
Z	4	
Density (calculated)	1.267 Mg/m^3	
Absorption coefficient	0.092 mm^{-1}	
F(000)	912	
Crystal size	0.100 x 0.100 x 0.050 mm^3	
Theta range for data collection	1.893 to 34.187°.	
Index ranges	-10<=h<=10, -12<=k<=11, -31<=l<=31	
Reflections collected	27488	
Independent reflections	5012 [R(int) = 0.1195]	
Completeness to theta = 29.731°	97.7 %	
Absorption correction	"cylinder"	
Max. and min. transmission	0.8454 and 0.8444	
Refinement method	Full-matrix least-squares on F^2	
Data / restraints / parameters	5012 / 1 / 283	
Goodness-of-fit on F^2	1.052	
Final R indices [I>2sigma(I)]	R1 = 0.0664, wR2 = 0.1527	
R indices (all data)	R1 = 0.0698, wR2 = 0.1559	
Absolute structure parameter	0.4(8)	
Extinction coefficient	n/a	
Largest diff. peak and hole	0.615 and -0.593 e.$Å^{-3}$	

Crystal structure of 6f.

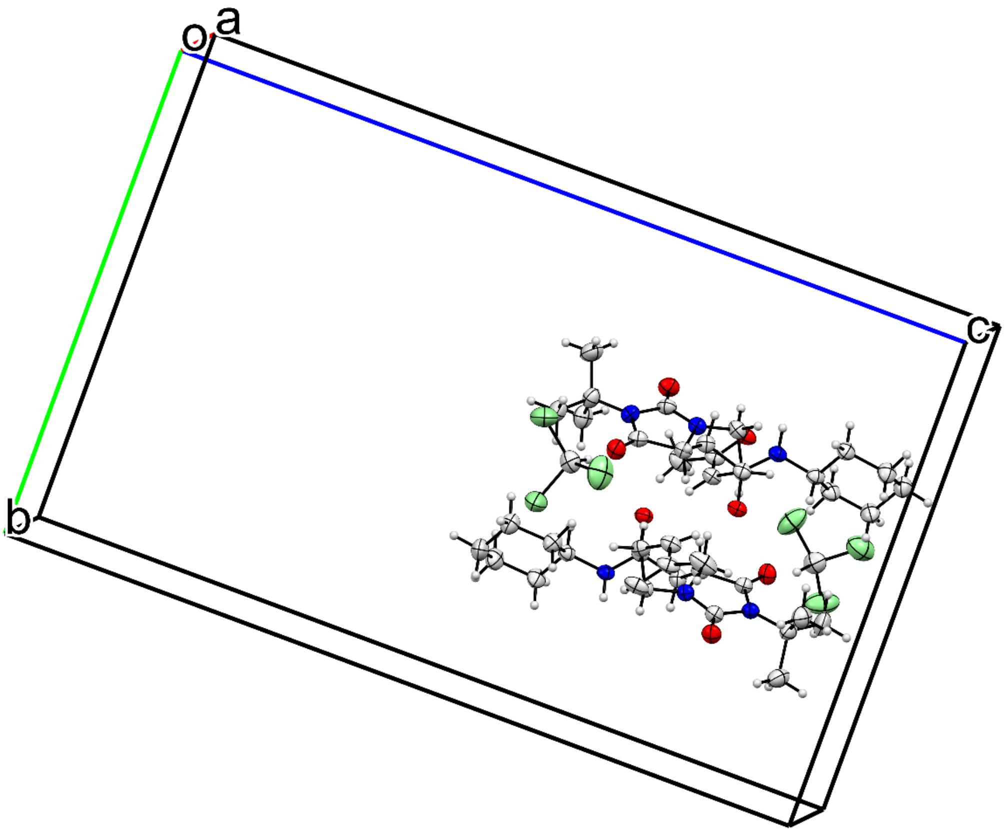

Figure S10. Crystal structure of **6f** showing only the asymmetric unit. ORTEP plot with thermal ellipsoids drawn at the 50 % of probability level.

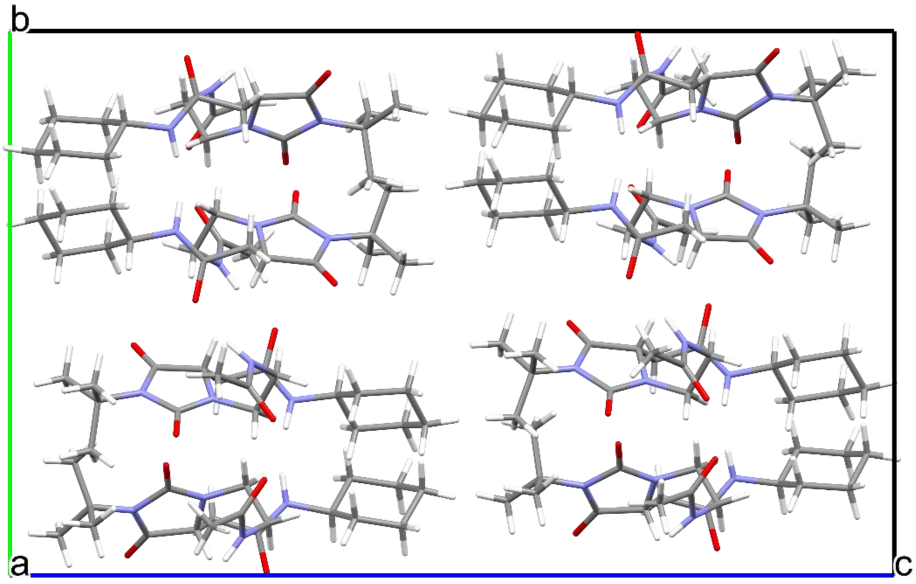

Figure S11. Crystal packing of **6f** viewed along the *a*-axis without $CHCl_3$.

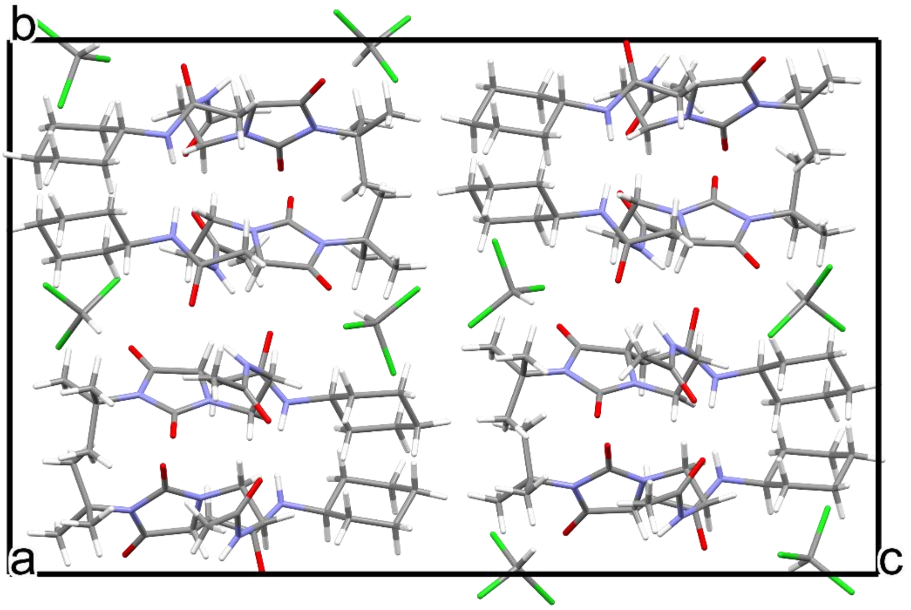

Figure S12. Crystal packing of **6f** viewed along the *a*-axis including solvent $CHCl_3$.

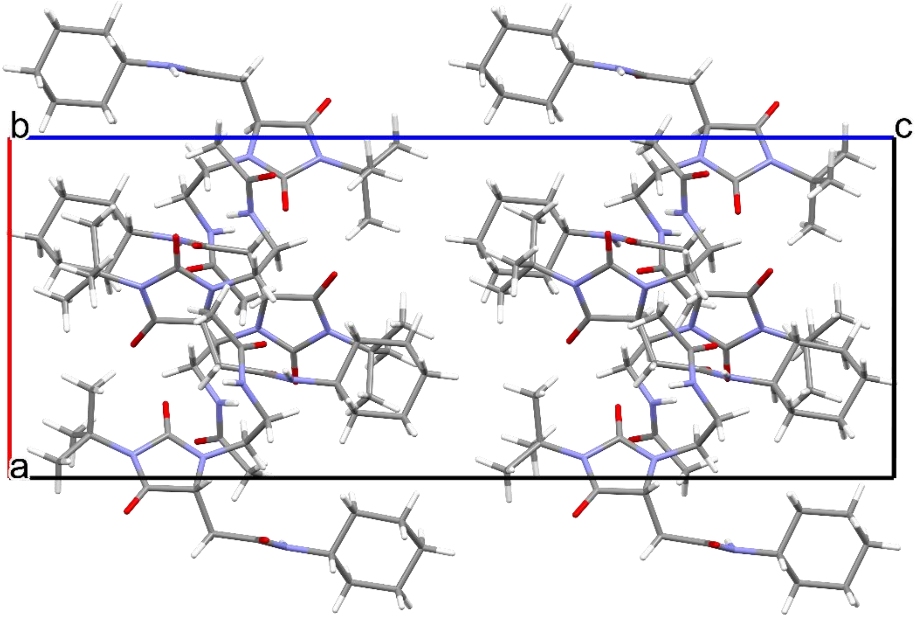

Figure S13. Crystal packing of **6f** viewed along the *b*-axis without $CHCl_3$.

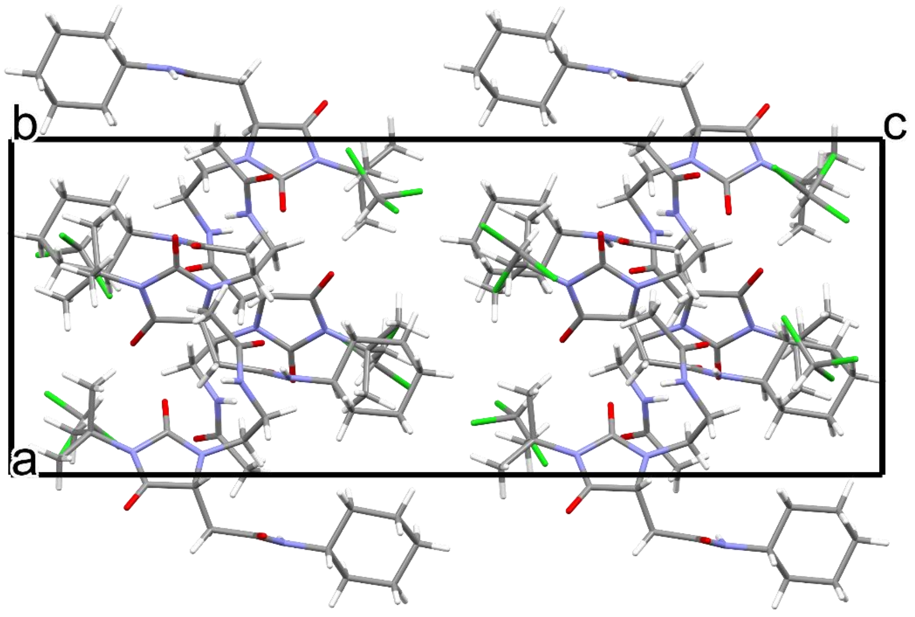

Figure S14. Crystal packing of **6f** viewed along the *b*-axis including solvent $CHCl_3$.

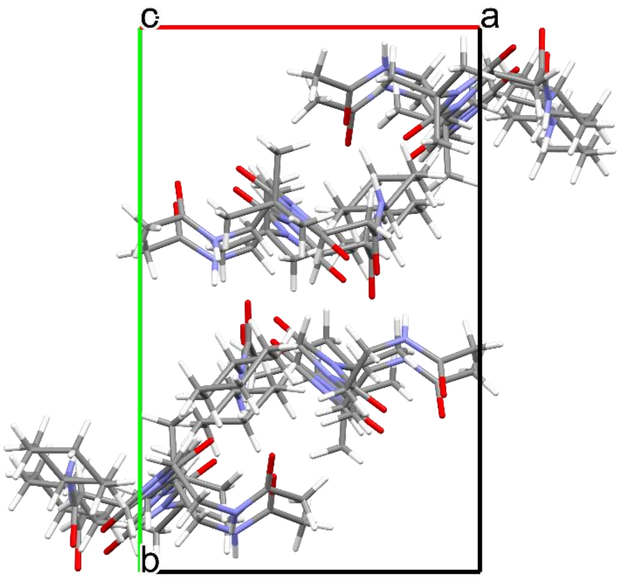

Figure S15. Crystal packing of **6f** viewed along the *c*-axis without $CHCl_3$.

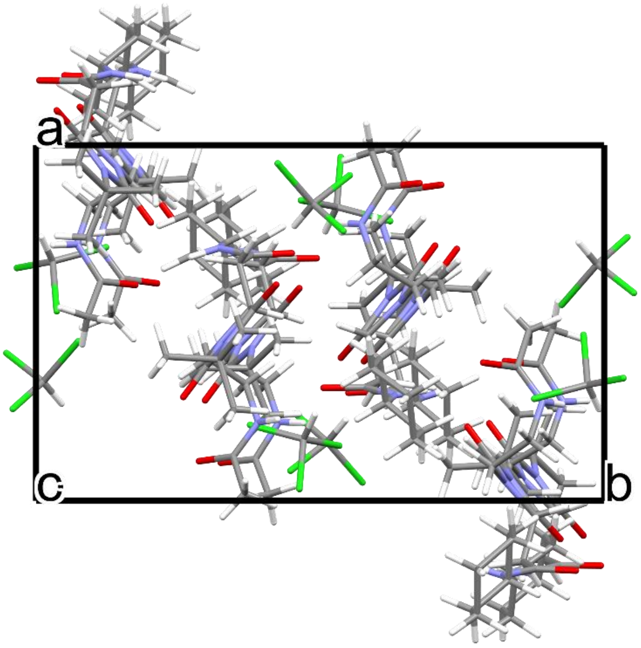

Figure 16. Crystal packing of **6f** viewed along the *c*-axis including solvent $CHCl_3$.

Table S12. Crystal data and structure refinement for **6f**.

Identification code	**6f**	
Empirical formula	$C_{20} H_{33} Cl_3 N_4 O_4$	
Formula weight	499.85	
Temperature	100(2) K	
Wavelength	0.700 Å	
Crystal system	Monoclinic	
Space group	*P* 21/*n*	
Unit cell dimensions	*a* = 10.719(2) Å	α = 90°.
	b = 17.131(3) Å	β = 90.03(3)°.
	c = 27.859(6) Å	γ = 90°.
Volume	5115.7(18) Å^3	
Z	8	
Density (calculated)	1.298 Mg/m^3	
Absorption coefficient	0.390 mm^{-1}	
F(000)	2112	
Crystal size	0.100 x 0.050 x 0.020 mm^3	
Theta range for data collection	1.374 to 29.656°.	
Index ranges	-15<=h<=15, -22<=k<=22, -39<=l<=39	
Reflections collected	92490	
Independent reflections	14882 [R(int) = 0.0624]	
Completeness to theta = 24.835°	99.6 %	
Absorption correction	"sphere"	
Max. and min. transmission	0.8621 and 0.8614	
Refinement method	Full-matrix least-squares on F^2	
Data / restraints / parameters	14882 / 4 / 395	
Goodness-of-fit on F^2	1.071	
Final R indices [I>2sigma(I)]	R1 = 0.1217, wR2 = 0.3700	
R indices (all data)	R1 = 0.1587, wR2 = 0.4002	
Extinction coefficient	n/a	
Largest diff. peak and hole	1.009 and -1.029 e.Å^{-3}	
